# Supplementary material for: Human longevity is influenced by many genetic variants: evidence from 75,000 UK Biobank participants
Source: Aging (Albany NY). 2016 Mar 23;8(3):547–60. doi: 10.18632/aging.100930 (PMC4833145; doi:10.18632/aging.100930)
Supplement: Supplementary file 1 [file aging-08-547-s001.pdf]

SUPPLEMENTARY METHODS

Selection of variants for GRS

Body mass index. We selected 69 of 76 common genetic variants associated with BMI at genome wide significance in the GIANT consortium studies of up to 339,224 individuals [1]. We limited the BMI SNPs to those that were associated with BMI in the analysis of all European ancestry individuals and did not include those that only reached genome-wide levels of statistical confidence in one-sex only, or one-strata only. Variants were also excluded if classified as a secondary signal within a locus. [Three SNPs were excluded due to potential pleiotropy (rs11030104, rs13107325, rs3888190)], 3 SNPs were not in Hardy-Weinberg Equilibrium (HWE) (rs17001654, rs2075650, rs9925964) and one SNP was unavailable (rs2033529) (Supplementary Table 1).

Coronary artery disease. Of 44 genetic variants associated ($p<5x10^{-8}$) with Coronary Artery Disease (CAD) in the recent meta-analysis of up to 185,000 cases and controls [2] one was excluded based on $R^2>0.1$ with another SNP (<1Mb distance) in the GRS, and one other was included as it was not available in the data; leaving 42 eligible SNPs to include (Supplementary Table 1).

Lipids: LDL, HDL and TG. The most recent Global Lipids Consortium meta-analysis [3] (n=188,577) reported the following numbers of variants associated ($p<5x10^{-8}$) with: LDL (55), HDL (67), and TG (37). Three LDL SNPs were not in HWE (rs1800562, rs2902940, rs1250229) so were excluded. No further exclusions were required. Therefore 52 LDL SNPs, 67 HDL SNPs and 37 TG SNPs were included in the respective GRS' (Supplementary Table 1).

Coronary artery disease – independent of lipids. Of the 42 SNPs included in the CAD GRS we excluded 9 where the SNP (or an exact proxy) was present in the LDL meta-analysis and associated with LDL p<0.05, an additional 4 SNPs that were associated p<0.05 with HDL, and 6 SNPs for which no proxies in the lipid GWAS could be identified, to create a "CAD no lipids" GRS including 23 SNPs associated with CAD but not LDL, HDL or TG (Supplementary Table 1).

Systolic blood pressure. We generated GRS for systolic blood pressure (SBP) only, due to the substantial overlap between the SNPs identified for SBP and diastolic blood pressure, based on the results of a meta-analysis on up to 200,000 participants of European ancestry [4]. All 26 SNPs for SBP were present in the UK BioBank imputation data with quality >0.95, and all HWE p-values $>1x10^{-6}$ (Supplementary Table 1).

Stroke. In 2014 a GWAS of 17,970 ischemic stroke cases reported 4 loci associated with stroke (rs17696736, rs2023938, rs12646447, rs10744777) [5]. All four were present in the imputed Biobank genetics data with quality >0.95 and HWE p-values $>1x10^{-6}$ (Supplementary Table 1).

Alzheimer's disease. Results from a meta-analysis including 4,018 Alzheimer's Disease (AD) cases showed 7 loci significantly associated with late-onset AD [6]. All 7 were used to create the GRS in this study (imputation quality =1 for all SNPs, HWE $p>1x10^{-6}$) (Supplementary Table 1).

Breast Cancer. The most recent breast cancer GWAS meta-analysis found 41 new loci, bringing the total to 66 [7]. One SNP (rs614367) was excluded due to HWE $p<1x10^{-6}$, leaving 65 for the GRS (Supplementary Table 1).

Prostate cancer. The most recent GWAS meta-analysis identified 23 new loci for prostate cancer (PC), bringing the total to 87 [8]. We used the results from participants on European descent (35,093 cases, 34,599 controls) to generate the risk score. All SNPs have imputation quality >0.95, however 2 SNPs (rs1775148 and rs1983891) were excluded due to HWE $p<1x10^{-6}$, meaning 85 SNPs were included in the GRS (Supplementary Table 1).

Inflammatory bowel disease. Inflammatory Bowel Disease (IBD) is commonly defined as presence of either Crohn's Disease (CD) or Ulcerative Colitis (UC), and a recent GWAS meta-analysis found significant overlap between CD and UC, but also some genetic divergence [9]. In total 232 unique SNPs are reported with either CD, UC or IBD in the 86,640 individuals of European descent in the study, 142 with CD, 89 with UC, and 159 with IBD. We generated three GRS based on these lists: for IBD 3 SNPs were excluded due to HWE p<1x10-6 (rs4703855, rs224090, rs17622378), for CD the same 3 SNPs were excluded, and for UC 2 SNPs were excluded due to HWE p<1x10-6 (rs17622378 and rs17771967 – the latter is unique to the UC list of significantly associated SNPs). Therefore the following number of SNPs were included: 156 for IBC, 139 for CD and 87 for UC (Supplementary Table 1).

Forced vital capacity. In an analysis of 85,170 participants for Forced Vital Capacity (FVC) 6 loci were significant in the combined meta-analysis [10]. All

were imputed in UK BioBank with high quality (>0.95) and with HWE $p>1x10^{-6}$. Therefore all 6 SNPs were included in the FVC GRS (Supplementary Table 1).

Age at menopause. Fifty four common SNPs were identified or confirmed in the most recent GWAS of age-at-menopause, in addition to 3 low-frequency (MAF >0.5% and <5%) coding variants identified by exome array [11]. One "exome SNP" (rs148126992) was excluded because it is not independent of rs75770066, which is more significant in the meta-analysis. Four of the common SNPs (rs6484478, rs3741604, rs1183272, rs7397861) were excluded because their association with age-at-menopause was only significant ($p<5x10^{-8}$) in joint analyses for regions containing more than one SNP. Therefore 52 SNPs were included in the GRS. Greater values of age-at-menopause GRS equate to earlier menopausal age.

Telomere length. Seven loci were identified to be associated with Telomere Length (TL) in a recent GWAS meta-analysis [12]. All 7 were imputed with high quality and no significant deviation from HWE was observed, therefore all 7 contributed to the GRS. Greater values of TL GRS equate to longer telomeres.

Type-1 diabetes. In total 29 SNPs were used to create the GRS for Type-1 Diabetes (T1D), using the methods as described in [13] (Supplementary Table 1). Two SNPs (rs2187668, rs7454108) were used to determine an individual's haplotype combination (DR3/DR4-DQ8) with each haplotype combination having an associated effect size. 27 out of 30 non-HLA SNPs were utilized in addition to the HLA haplotype-tagging SNPs (3 were excluded due to HWE $p<5x10^{-6}$).

Type-2 diabetes. Of 65 loci confirmed to be associated with Type-2 Diabetes (T2D) in a recent GWAS meta-analysis (34,840 cases) [14] 10 were excluded due to non-specific effects (e.g. primary effect of rs12970134 is on BMI, therefore excluded here) and one was substituted for a proxy (rs11651052 substituted for rs4430796). Therefore 55 SNPs are included in the GRS (see Supplementary Table 1 for details).

SUPPLEMENTARY REFERENCES

1. Locke AE, Kahali B, Berndt SI, Justice AE, Pers TH, Day FR, et al. Genetic studies of body mass index yield new insights for obesity biology. Nature. 2015; 518:197–206.

2. The CARDIoGRAMplusC4D Consortium. A comprehensive 1000 Genomes-based genome-wide association meta-analysis of coronary artery disease. Nat. Genet. 2015; 47.

3. Global Lipids Genetics Consortium, Willer CJ, Schmidt EM, Sengupta S, Peloso GM, Gustafsson S, et al. Discovery and refinement of loci associated with lipid levels. Nat. Genet. 2013; 45:1274–1283.

4. Ehret GB, Munroe PB, Rice KM, Bochud M, Johnson AD, Chasman DI, et al. Genetic variants in novel pathways influence blood pressure and cardiovascular disease risk. Nature. Nature Publishing Group; 2011; 478:103–109.

5. Kilarski LL, Achterberg S, Devan WJ, Traylor M, Malik R, Lindgren A, et al. Meta-analysis in more than 17,900 cases of ischemic stroke reveals a novel association at 12q24.12. Neurology. 2014; 83:678–685.

6. Kamboh MI, Demirci FY, Wang X, Minster RL, Carrasquillo MM, Pankratz VS, et al. Genome-wide association study of Alzheimer's disease. Transl. Psychiatry. 2012; 1–7.

7. Michailidou K, Hall P, Gonzalez-Neira A, Ghoussaini M, Dennis J, Milne RL, et al. Large-scale genotyping identifies 41 new loci associated with breast cancer risk. Nat. Genet. 2013; 45:353–61, 361e1–2.

8. Al Olama AA, Kote-Jarai Z, Berndt SI, Conti D V, Schumacher F, Han Y, et al. A meta-analysis of 87,040 individuals identifies 23 new susceptibility loci for prostate cancer. Nat. Genet. 2014; 46:1103–1109.

9. Liu JZ, van Sommeren S, Huang H, Ng SC, Alberts R, Takahashi A, et al. Association analyses identify 38 susceptibility loci for inflammatory bowel disease and highlight shared genetic risk across populations. Nat. Genet. 2015.

10. Loth DW, Artigas MS, Gharib SA, Wain L V, Franceschini N, Koch B, et al. Genome-wide association analysis identifies six new loci associated with forced vital capacity. Nat. Genet. 2014; 46:669–677.

11. Day FR, Ruth KS, Thompson DJ, Lunetta KL, Pervjakova N, Chasman DI, et al. Large-scale genomic analyses link reproductive aging to hypothalamic signaling, breast cancer susceptibility and BRCA1-mediated DNA repair. Nat. Genet. 2015; 47.

12. Codd V, Nelson CP, Albrecht E, Mangino M, Deelen J, Buxton JL, et al. Identification of seven loci affecting mean telomere length and their association with disease. Nat. Genet. 2013; 45:422–427.

13. Oram RA, Patel K, Hill A, Shields B, McDonald TJ, Jones A, et al. A Type 1 Diabetes Genetic Risk Score Can Aid Discrimination Between Type 1 and Type 2 Diabetes in Young Adults. Diabetes Care. 2015; 1–8.

14. Morris AP, Voight BF, Teslovich TM, Ferreira T, Segrè A V, Steinthorsdottir V, et al. Large-scale association analysis provides insights into the genetic architecture and pathophysiology of type 2 diabetes. Nat. Genet. 2012; 44:981–990.

SUPPLEMENTARY TABLES

Please browse Full text version of this manuscript to see Supplementary Tables:

Supplementary Table 1. Lists the genetic variants included for each genetic risk score.

Supplementary Table 2. Includes the 1,000 most-associated variants from each of the four GWAS performed.

Supplementary Table 3. Includes the full results for all associations between the genetic risk scores and the four parent's age-at-death phenotypes.

Supplementary Table 4. The results from the four GWAS performed for a specific set of variants with *a priori* hypotheses regarding human longevity.

SUPPLEMENTARY FIGURES

Please browse Full text version of this manuscript to see Supplementary Figures:

Supplementary Figure 1. Flow-chart demonstrating the derivation (and number of participants included) for each of the four phenotypes/analyses utilized in the analyses.

Supplementary Figure 2. QQ plot and Manhattan plot for the GWAS of father's age at death.

Supplementary Figure 3. QQ plot and Manhattan plot for the GWAS of mother's age at death.

Supplementary Figure 4. QQ plot and Manhattan plot for the GWAS of combined parents' age at death.

Supplementary Figure 5. QQ plot and Manhattan plot for the GWAS of extreme longevity (at least one parent lived to the top 1% of the age at death distribution in the Biobank respondents).

Supplementary Figure 6. LocusZoom plot shows the chromosomal locations of variants within 250kbp of rs1051730 (x-axis), and the degree of association with father's age at death (y-axis) in the GWAS. The colors reflect the degree of linkage disequilibrium (red = high) with rs1051730 (purple).

Supplementary Figure 7. LocusZoom plot shows the chromosomal locations of variants within 250kbp of rs62227724 (x-axis), and the degree of association with mother's age at death (y-axis) in the GWAS. The colors reflect the degree of linkage disequilibrium (red = high) with rs62227724 (purple).

Supplementary Figure 8. LocusZoom plot shows the chromosomal locations of variants within 250kbp of rs75824829 (x-axis), and the degree of association with extreme longevity (y-axis) in the GWAS (at least one parent lived to the top 1% of age at death distribution in UK Biobank respondents). The colors reflect the degree of linkage disequilibrium (red = high) with rs75824829 (purple).

Supplementary Figure 9. LocusZoom plot shows the chromosomal locations of variants within 250kbp of rs79109928 (x-axis), and the degree of association with extreme longevity (y-axis) in the GWAS (at least one parent lived to the top 1% of age at death distribution in UK Biobank respondents). The colors reflect the degree of linkage disequilibrium (red = high) with rs79109928 (purple).
LocusZoom utilizes information from the Nov 2014. Release of the 1000 genomes panel, which does not appear to include variant rs528161076, the most-associated variant in the GWAS. We searched for proxies for this variant in the UK Biobank respondents and found that rs79109928 (located 1323 bases from rs528161076) is in high linkage disequilibrium (R^2=0.943); we therefore created the LocusZoom plot focused on rs79109928 and have included a "star" symbol to represent the location of rs528161076, and the degree of association with extreme longevity.

Supplementary Figure 10. Forest plot showing the associations between the GRS for CAD, LDL, HDL and AD with and without the APOE variant rs4420638.
