# Supplementary material for: Human longevity is influenced by many genetic variants: evidence from 75,000 UK Biobank participants
Source: Aging (Albany NY). 2016 Mar 23;8(3):547–60. doi: 10.18632/aging.100930 (PMC4833145; doi:10.18632/aging.100930)

1)

UK Biobank participants included in the primary analyses

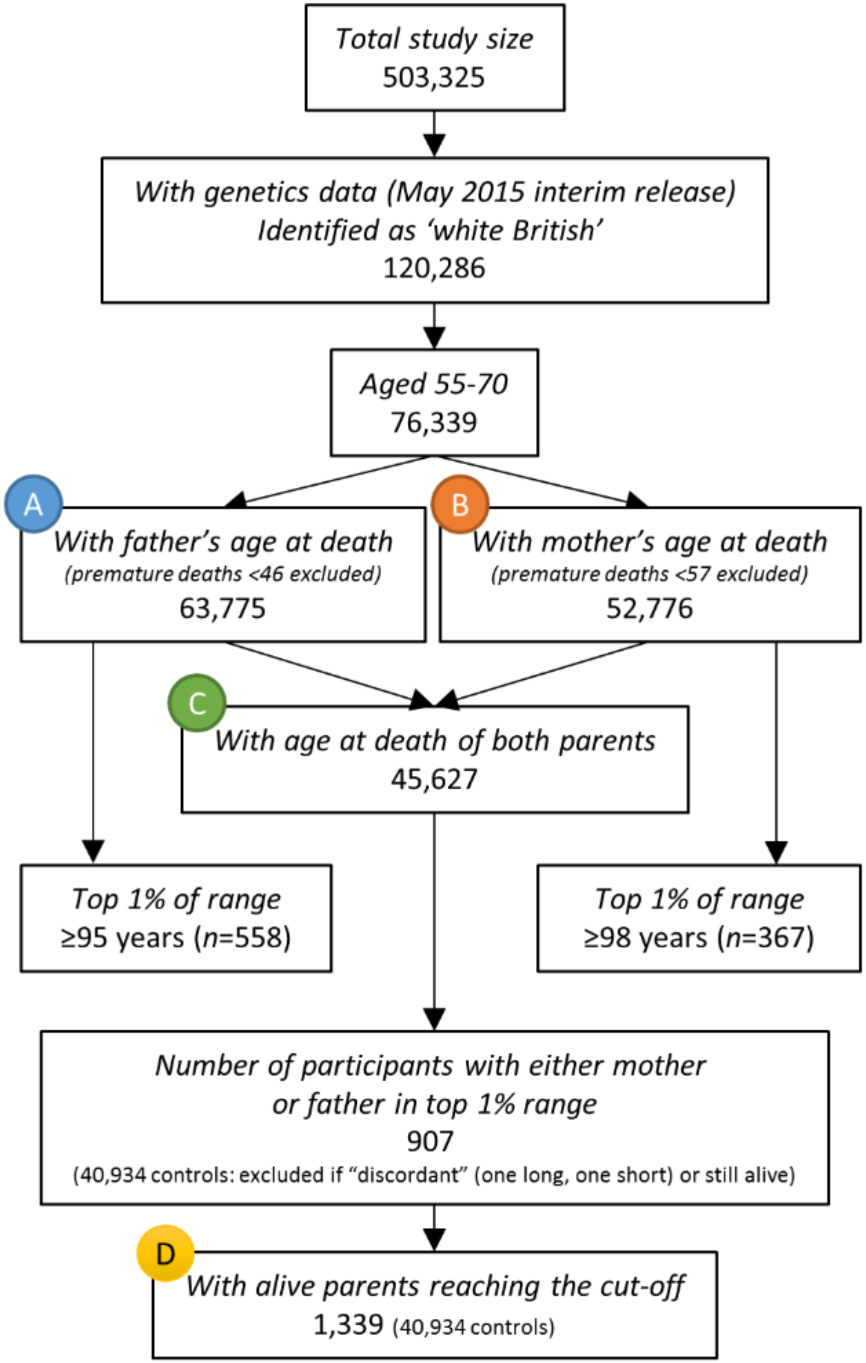

Highlighted here with A-D

A) Father's age at death,
B) Mother's age at death,
C) Combined parental age at death (z-scored),
D) Binary phenotype: at least one parent attained the top 1% cut-off, derived from the mother's/father's ages at death

75,244 participants included in at least one analysis

2) QQ plot for GWAS of father's age at death in 63,775 UK Biobank participants (9million imputed variants)

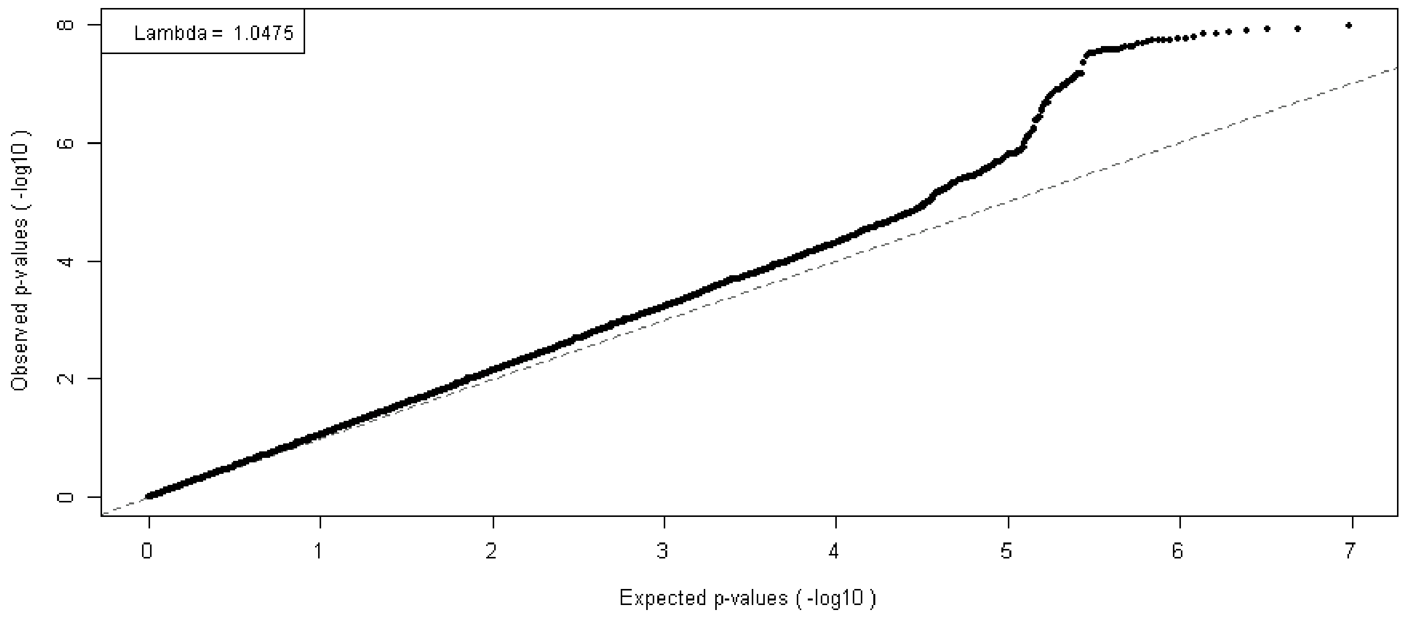

Manhattan plot of above data

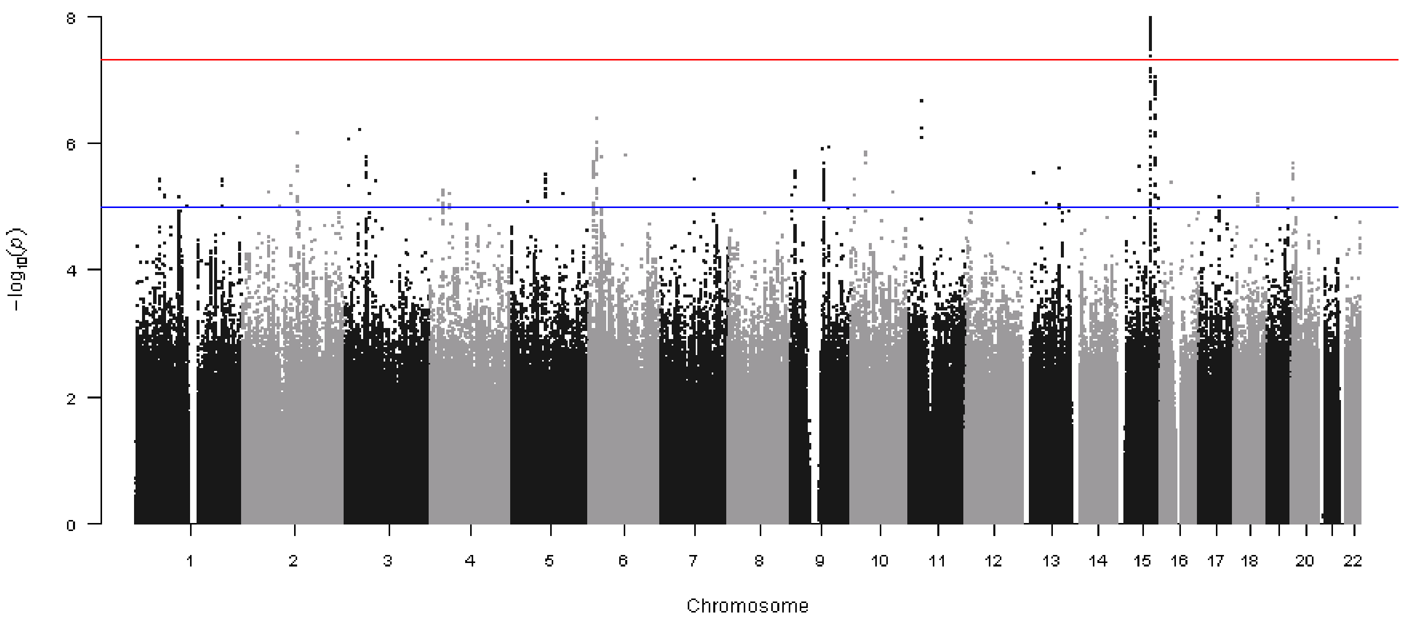

3) QQ plot for GWAS of mother's age at death in 52,776 UK Biobank participants (9million imputed variants)

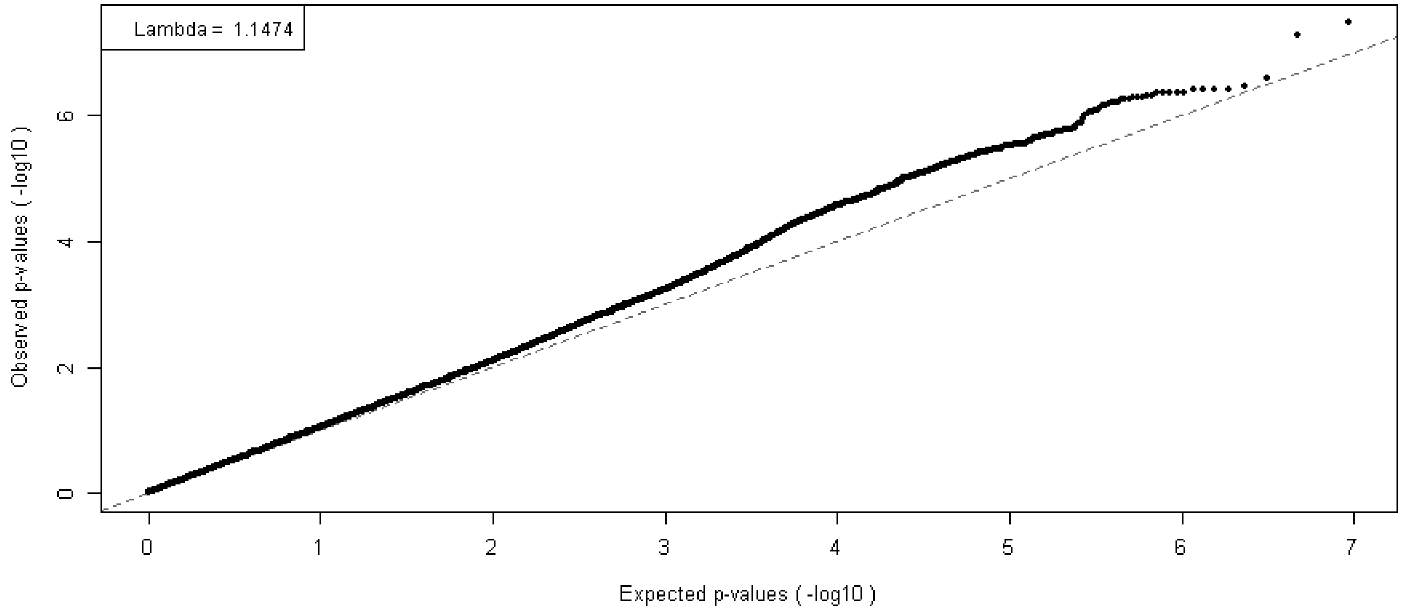

Manhattan plot of above data

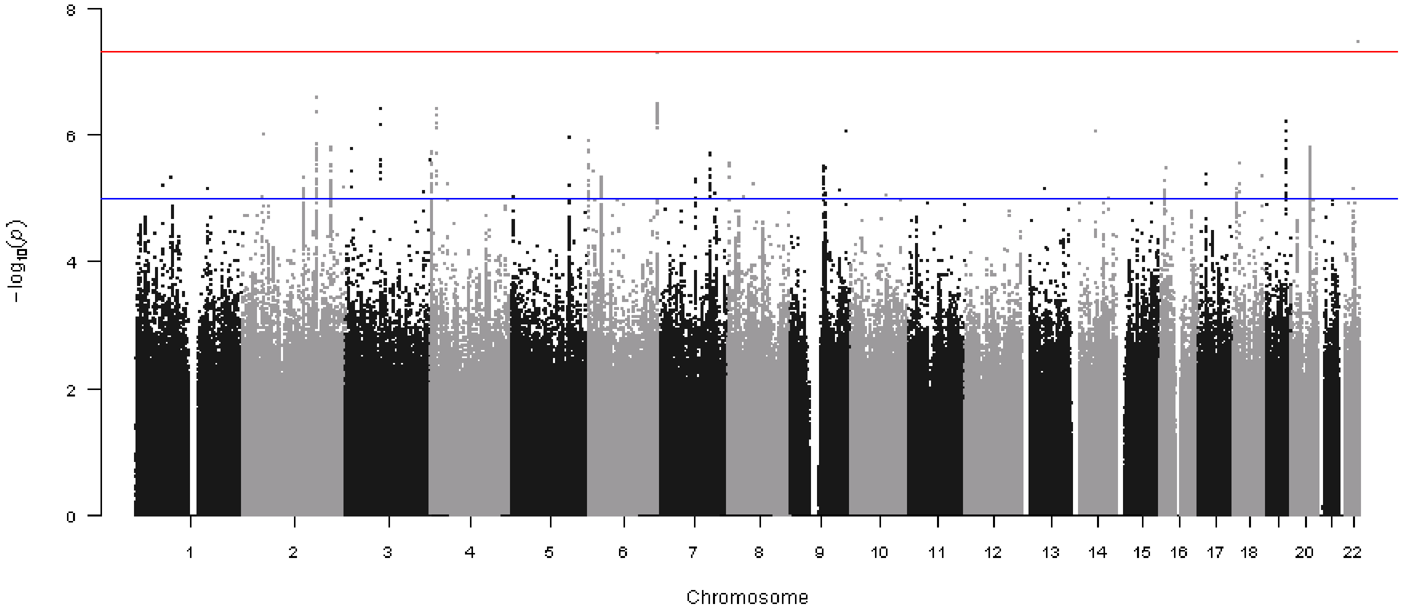

4) QQ plot for GWAS of combined parent's age at death in 45,627 UK Biobank participants (9million imputed variants)

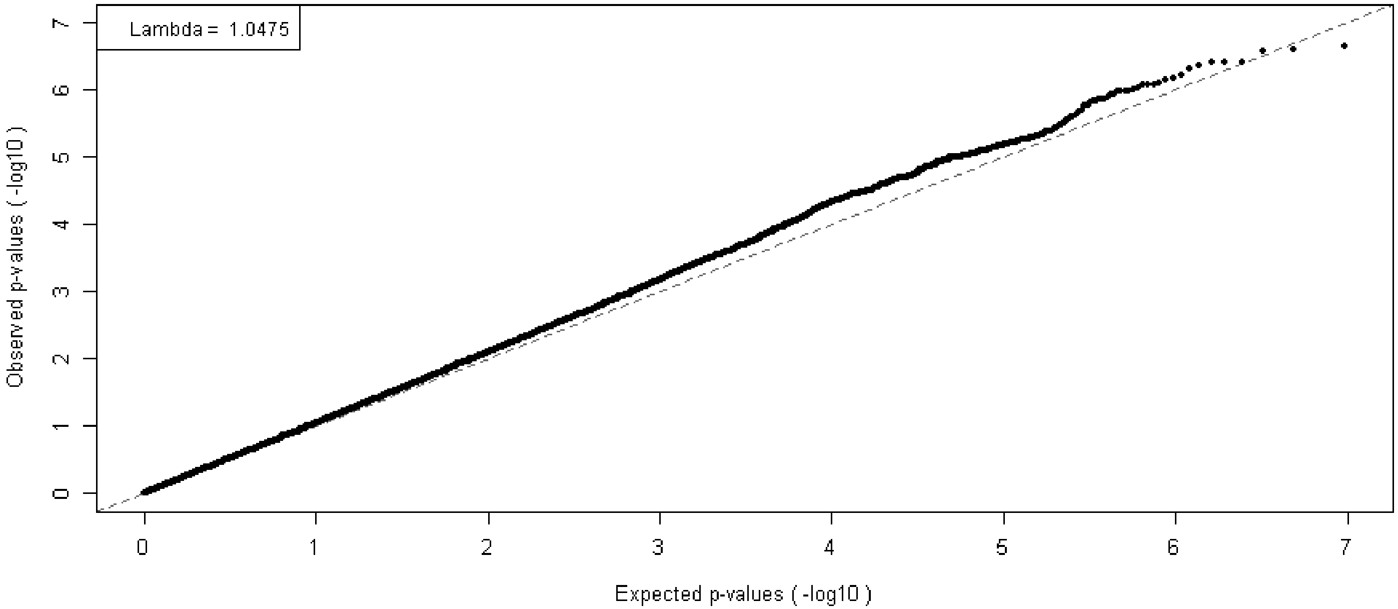

Manhattan plot of above data

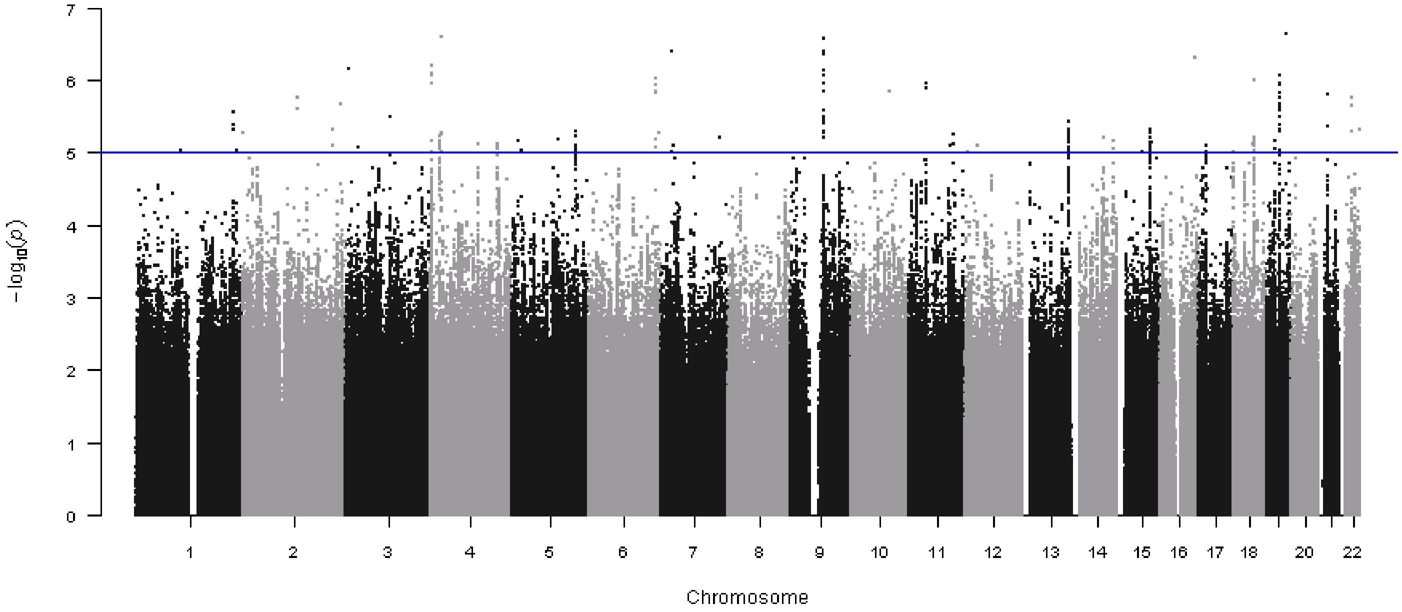

5) QQ plot for GWAS of “top 1% of age at death range” in 42,273 UK Biobank participants (9million imputed variants)

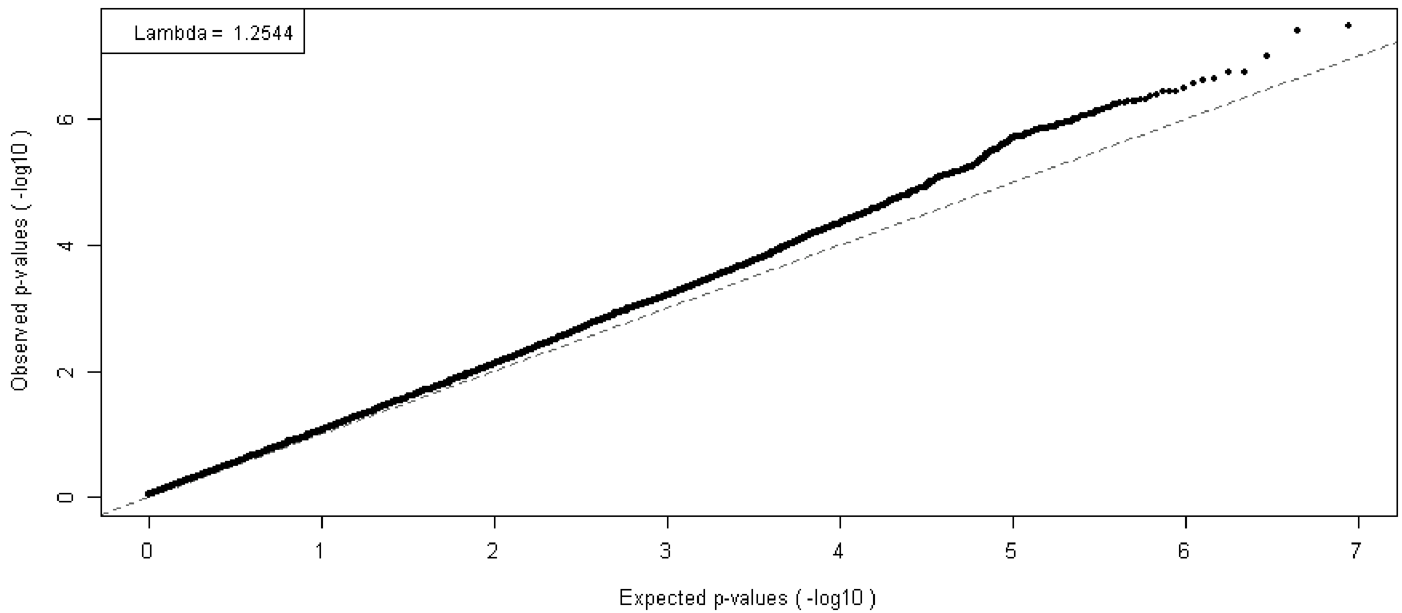

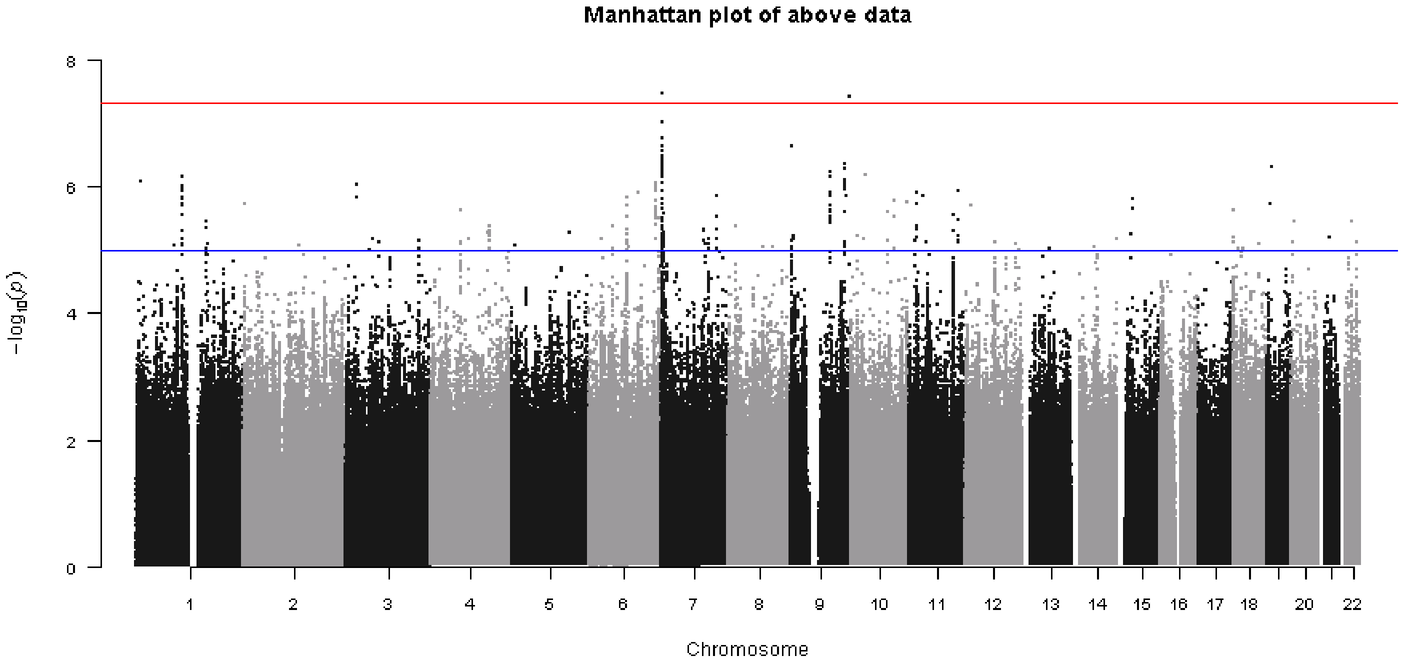

6) Locus Zoom plot displaying variants +/- 250kbp around variant rs1051830
P-values are the association with Father's age at death

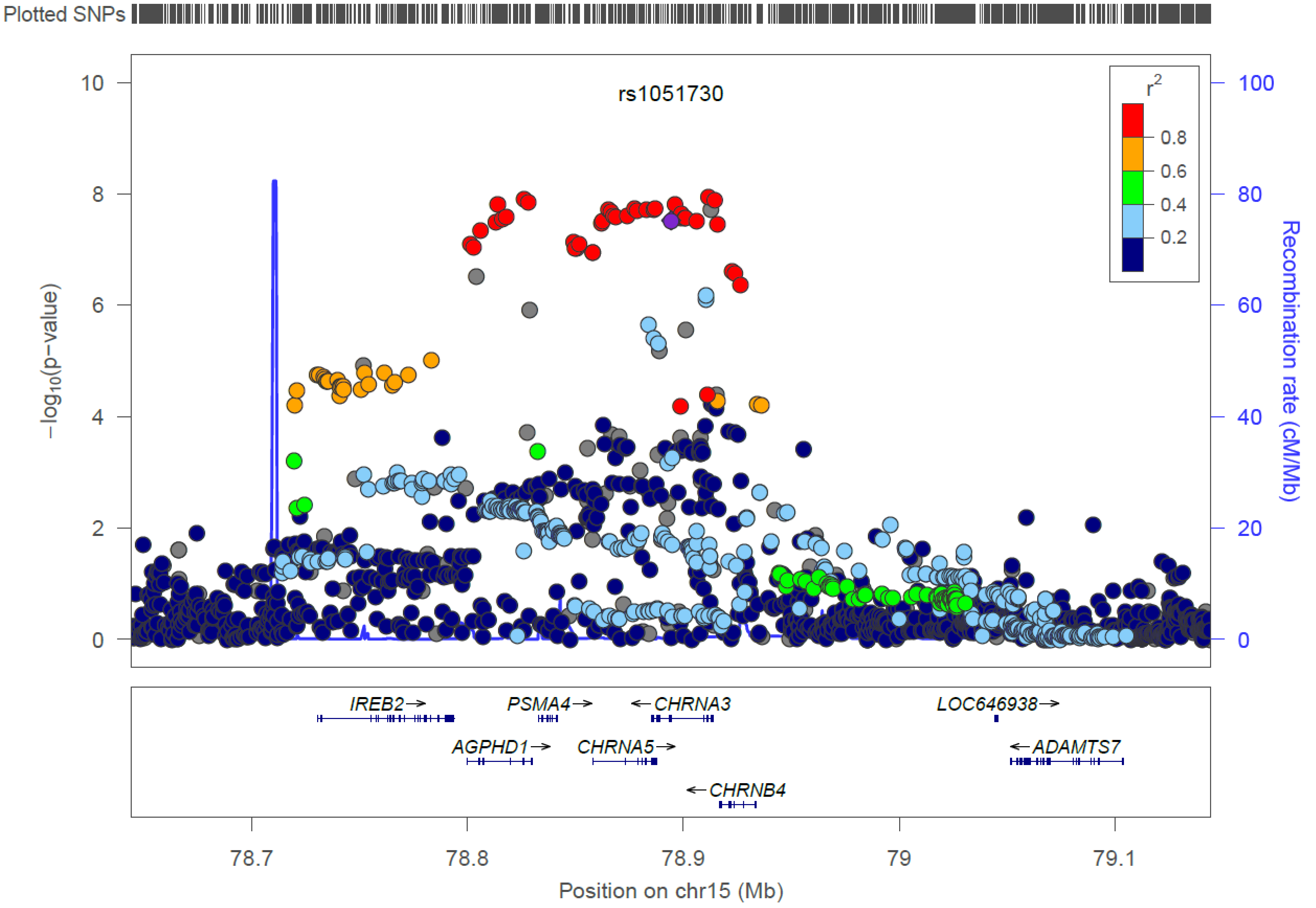

7) Locus Zoom plot displaying variants +/- 250kbp around variant rs62227724
P-values are the association with Mother's age at death

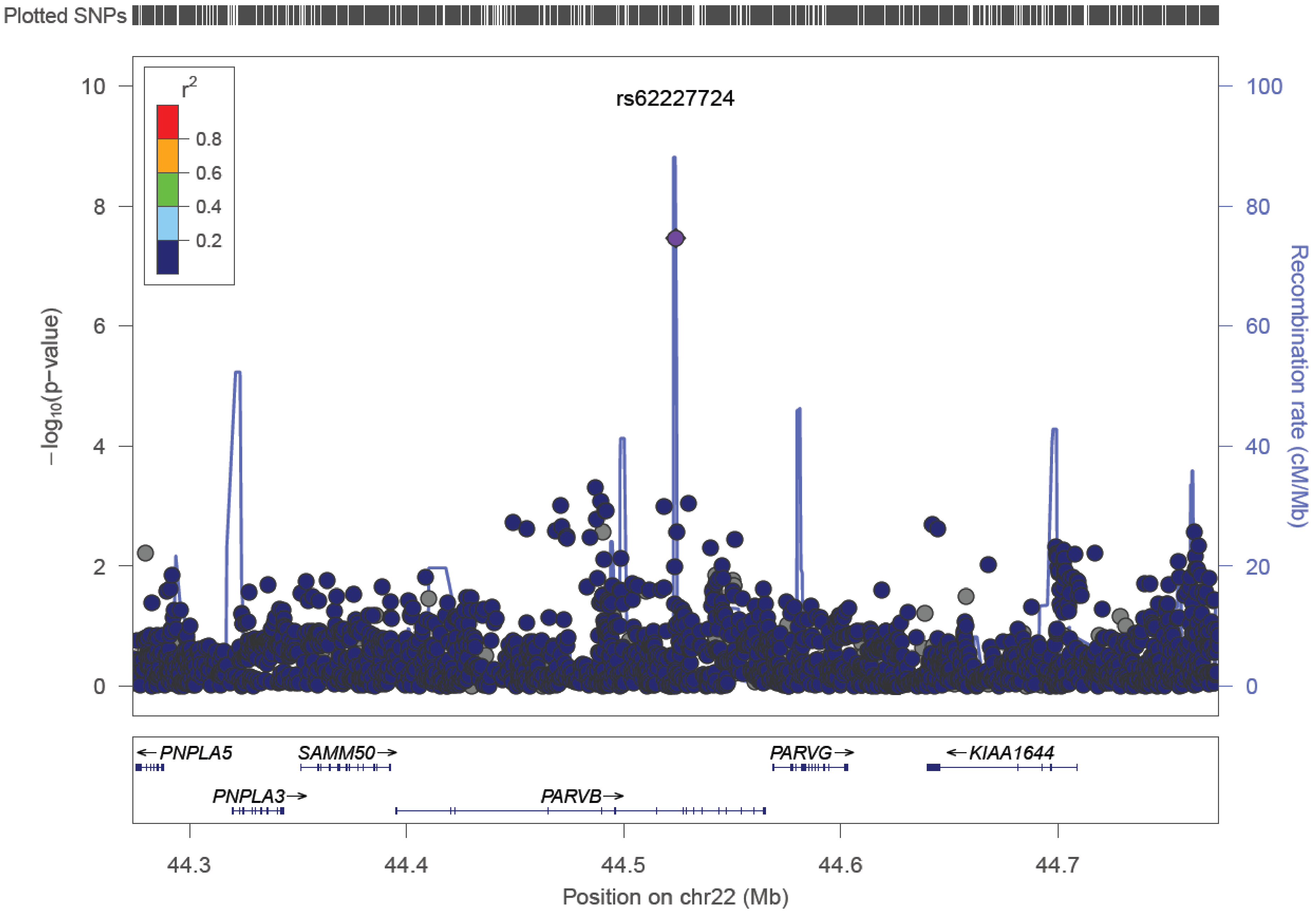

8) Locus Zoom plot displaying variants +/- 250kbp around variant rs75824829

P-values are the association with “top 1% of age at death range”

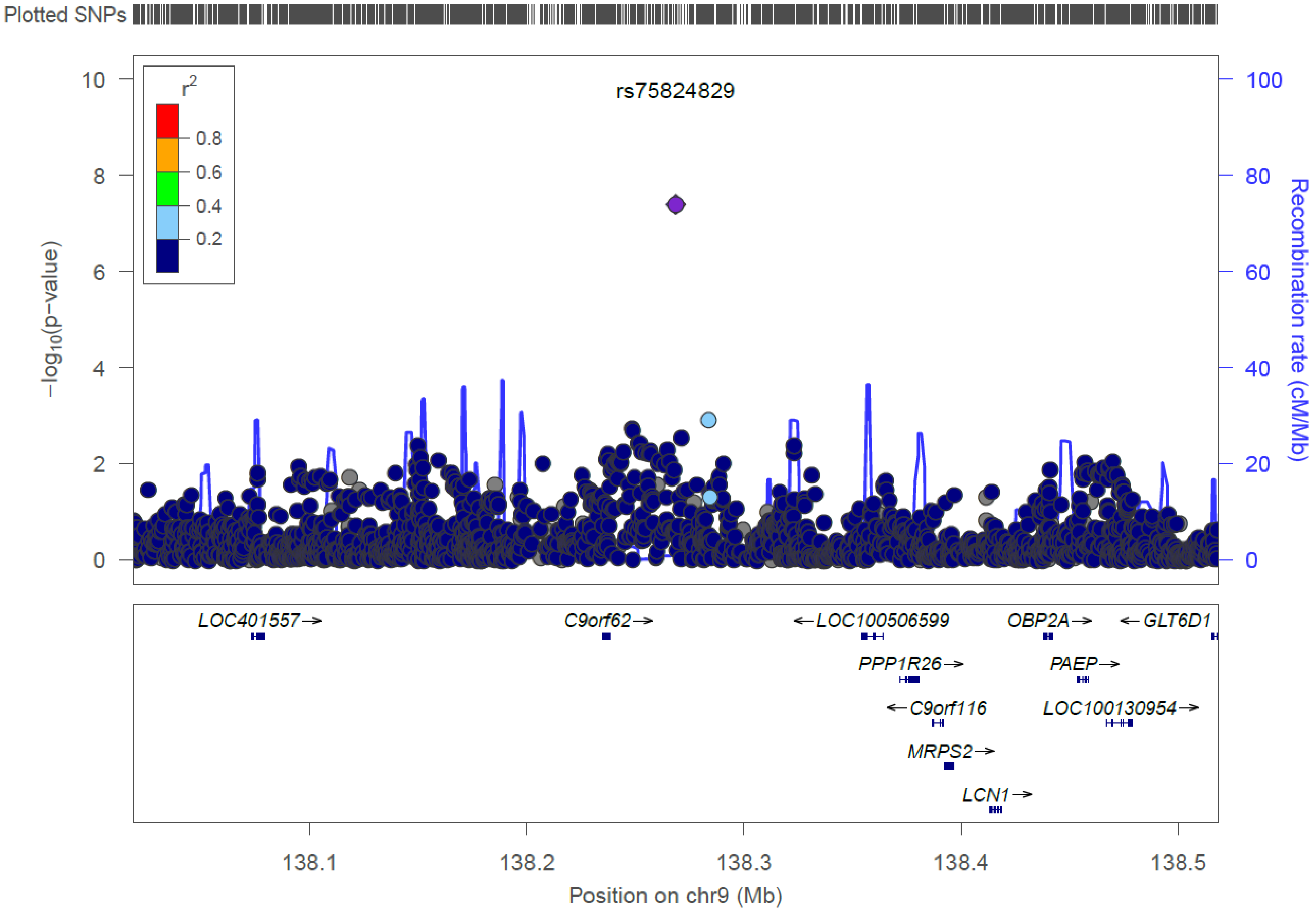

9) Locus Zoom plot displaying variants +/- 250kbp around variant rs528161076

P-values are the association with “top 1% of age at death range”

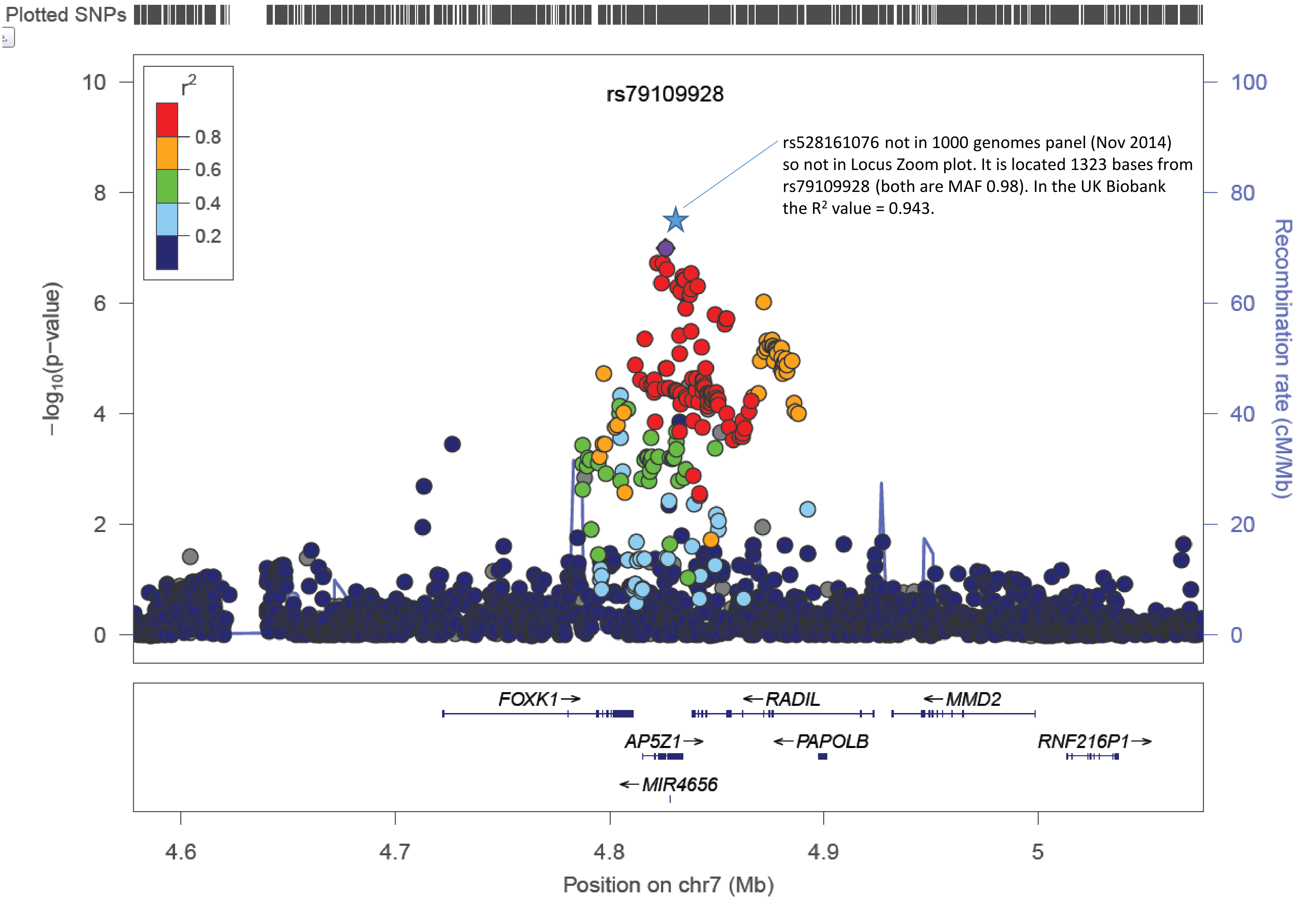

10) Comparing GRS associations with parental age-at-death phenotypes before/after exclusion of APOE variant rs4420638

Parent's age-at-death (combined z-scores) (linear regression models)

GRS (n SNPs)	Coef	95% CI
Coronary Artery Disease (CAD) (42)	-0.031	-0.040 to -0.022
Coronary Artery Disease (CAD) (42) no ApoE	-0.029	-0.038 to -0.020
Low Density Lipoprotein (49)	-0.024	-0.033 to -0.015
Low Density Lipoprotein (49) no ApoE	-0.018	-0.027 to -0.009
High Density Lipoprotein (67)	0.006	-0.003 to 0.015
High Density Lipoprotein (67) no ApoE	0.004	-0.005 to 0.013
Alzheimer's Disease (8)	-0.011	-0.020 to -0.002
Alzheimer's Disease (8) no ApoE	0.002	-0.007 to 0.011

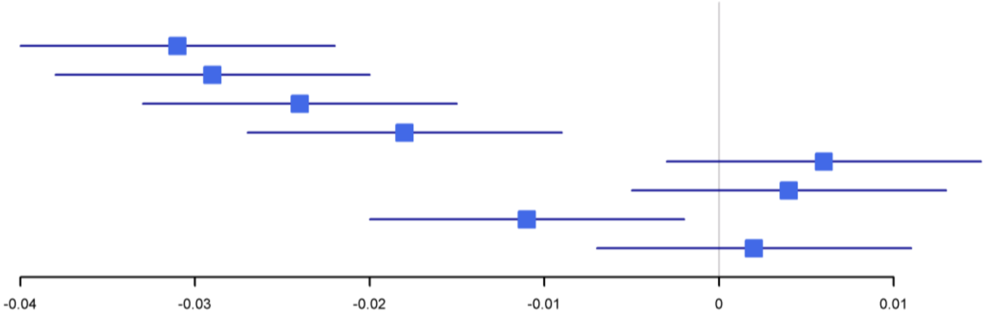

Father's age-at-death (linear regression models)

GRS (n SNPs)	Coef	95% CI
Coronary Artery Disease (CAD) (42)	-0.024	-0.032 to -0.016
Coronary Artery Disease (CAD) (42) no ApoE	-0.024	-0.031 to -0.016
Low Density Lipoprotein (49)	-0.016	-0.024 to -0.008
Low Density Lipoprotein (49) no ApoE	-0.016	-0.023 to -0.008
High Density Lipoprotein (67)	0.007	-0.001 to 0.015
High Density Lipoprotein (67) no ApoE	0.007	-0.001 to 0.014
Alzheimer's Disease (8)	0.000	-0.008 to 0.008
Alzheimer's Disease (8) no ApoE	0.005	-0.003 to 0.013

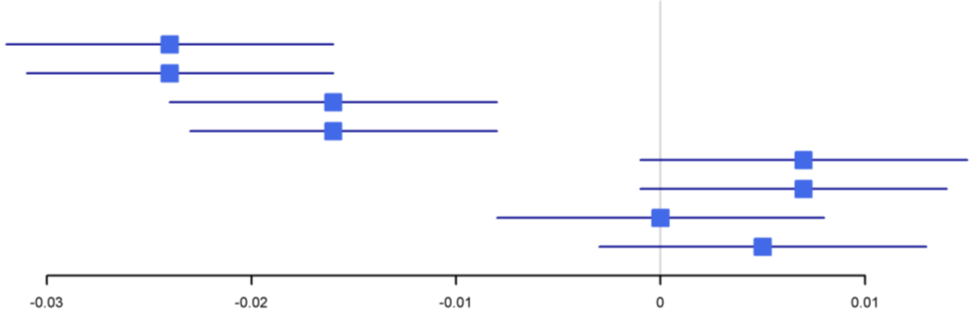

Mother's age-at-death (linear regression models)

GRS (n SNPs)	Coef	95% CI
Coronary Artery Disease (CAD) (42)	-0.020	-0.028 to -0.011
Coronary Artery Disease (CAD) (42) no ApoE	-0.017	-0.026 to -0.009
Low Density Lipoprotein (49)	-0.011	-0.019 to -0.002
Low Density Lipoprotein (49) no ApoE	-0.003	-0.012 to 0.005
High Density Lipoprotein (67)	0.003	-0.006 to 0.011
High Density Lipoprotein (67) no ApoE	0.000	-0.008 to 0.008
Alzheimer's Disease (8)	-0.015	-0.024 to -0.007
Alzheimer's Disease (8) no ApoE	-0.003	-0.012 to 0.005

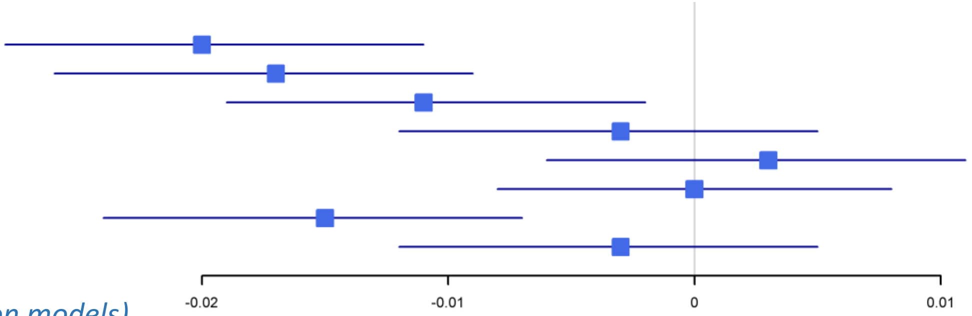

At least one parent reached top 1% (logistic regression models)

GRS (n SNPs)	OR	95% CI
Coronary Artery Disease (CAD) (42)	0.883	0.836 to 0.933
Coronary Artery Disease (CAD) (42) no ApoE	0.875	0.821 to 0.932
Low Density Lipoprotein (49)	0.892	0.844 to 0.942
Low Density Lipoprotein (49) no ApoE	0.899	0.844 to 0.957
High Density Lipoprotein (67)	1.080	1.022 to 1.140
High Density Lipoprotein (67) no ApoE	1.073	1.008 to 1.142
Alzheimer's Disease (8)	0.941	0.890 to 0.995
Alzheimer's Disease (8) no ApoE	1.033	0.970 to 1.100

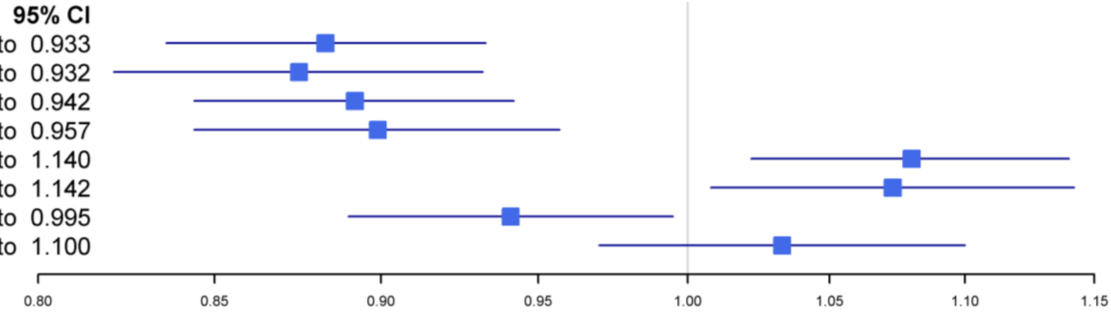

Supplement: Supplementary file 2 [file aging-08-547-s002.pdf]
