# Supplementary material for: Human longevity is influenced by many genetic variants: evidence from 75,000 UK Biobank participants
Source: Aging (Albany NY). 2016 Mar 23;8(3):547–60. doi: 10.18632/aging.100930 (PMC4833145; doi:10.18632/aging.100930)
Supplement: Supplementary file 3 [file aging-08-547-s003.docx]

**Supplementary Table 1**

Variants included in the genetic risk scores.

RA=risk (or trait-raising) allele. OR=odds ratio (or coefficient if continuous trait, e.g. BMI or Age at Menopause)

| **Trait** | **rsID** | **RA** | **OR** | **Excluded? (If text then the SNP was excluded due to reason given)** |
| --- | --- | --- | --- | --- |
| Alzheimer's Disease | rs4420638 | G | 3.45 |  |
| Alzheimer's Disease | rs3748140 | G | 2.43 |  |
| Alzheimer's Disease | rs17817600 | A | 1.33 |  |
| Alzheimer's Disease | rs7561528 | G | 1.25 |  |
| Alzheimer's Disease | rs12808148 | C | 1.23 |  |
| Alzheimer's Disease | rs11738335 | G | 1.2 |  |
| Alzheimer's Disease | rs6856768 | A | 1.18 |  |
| Alzheimer's Disease | rs1357692 | G | 1.16 |  |
| BMI | rs1558902 | A | 0.082 |  |
| BMI | rs17024393 | C | 0.066 |  |
| BMI | rs13021737 | G | 0.06 |  |
| BMI | rs6567160 | C | 0.056 |  |
| BMI | rs11847697 | T | 0.049 |  |
| BMI | rs13107325 | T | 0.048 | Missense Ala/Thr polymorphism located in exon 7 of SLC39A8, which encodes a zinc transporter that also transports cadmium and manganese. It is also associated with BP and HDL levels, and presumably these and the BMI effect are secondary to the metal ion transport variation. |
| BMI | rs16851483 | T | 0.048 |  |
| BMI | rs543874 | G | 0.048 |  |
| BMI | rs1516725 | C | 0.045 |  |
| BMI | rs2207139 | G | 0.045 |  |
| BMI | rs11030104 | A | 0.041 | BMI-raising allele also associated with regular smoking (which itself has a causal effect on BMI in opposite direction) |
| BMI | rs10938397 | G | 0.04 |  |
| BMI | rs12446632 | G | 0.04 |  |
| BMI | rs7899106 | G | 0.04 |  |
| BMI | rs11727676 | T | 0.036 |  |
| BMI | rs2287019 | C | 0.036 |  |
| BMI | rs12429545 | A | 0.033 |  |
| BMI | rs3101336 | C | 0.033 |  |
| BMI | rs2245368 | C | 0.032 |  |
| BMI | rs7138803 | A | 0.032 |  |
| BMI | rs10182181 | G | 0.031 |  |
| BMI | rs11057405 | G | 0.031 |  |
| BMI | rs11191560 | C | 0.031 |  |
| BMI | rs16951275 | T | 0.031 |  |
| BMI | rs17001654 | G | 0.031 | HWE p < 1e-6 |
| BMI | rs3888190 | A | 0.031 | Associated with lots of other traits and is a big haplotype |
| BMI | rs13078960 | G | 0.03 |  |
| BMI | rs9581854 | T | 0.03 |  |
| BMI | rs13191362 | A | 0.028 |  |
| BMI | rs3810291 | A | 0.028 |  |
| BMI | rs2075650 | A | 0.026 | HWE p < 1e-6 |
| BMI | rs2112347 | T | 0.026 |  |
| BMI | rs3817334 | T | 0.026 |  |
| BMI | rs10968576 | G | 0.025 |  |
| BMI | rs17094222 | C | 0.025 |  |
| BMI | rs2121279 | T | 0.025 |  |
| BMI | rs12566985 | G | 0.024 |  |
| BMI | rs7141420 | T | 0.024 |  |
| BMI | rs10132280 | C | 0.023 |  |
| BMI | rs1016287 | T | 0.023 |  |
| BMI | rs657452 | A | 0.023 |  |
| BMI | rs758747 | T | 0.023 |  |
| BMI | rs11165643 | T | 0.022 |  |
| BMI | rs12286929 | G | 0.022 |  |
| BMI | rs17405819 | T | 0.022 |  |
| BMI | rs205262 | G | 0.022 |  |
| BMI | rs7243357 | T | 0.022 |  |
| BMI | rs7599312 | G | 0.022 |  |
| BMI | rs11126666 | A | 0.021 |  |
| BMI | rs12401738 | A | 0.021 |  |
| BMI | rs12885454 | C | 0.021 |  |
| BMI | rs2650492 | A | 0.021 |  |
| BMI | rs4256980 | G | 0.021 |  |
| BMI | rs1167827 | G | 0.02 |  |
| BMI | rs2176598 | T | 0.02 |  |
| BMI | rs2365389 | C | 0.02 |  |
| BMI | rs2820292 | C | 0.02 |  |
| BMI | rs1000940 | G | 0.019 |  |
| BMI | rs17724992 | A | 0.019 |  |
| BMI | rs1928295 | T | 0.019 |  |
| BMI | rs2033529 | G | 0.019 | Not in data |
| BMI | rs2033732 | C | 0.019 |  |
| BMI | rs3849570 | A | 0.019 |  |
| BMI | rs6804842 | G | 0.019 |  |
| BMI | rs9400239 | C | 0.019 |  |
| BMI | rs9925964 | A | 0.019 | HWE p < 1e-6 |
| BMI | rs11583200 | C | 0.018 |  |
| BMI | rs12940622 | G | 0.018 |  |
| BMI | rs1528435 | T | 0.018 |  |
| BMI | rs29941 | G | 0.018 |  |
| BMI | rs3736485 | A | 0.018 |  |
| BMI | rs4740619 | T | 0.018 |  |
| BMI | rs10733682 | A | 0.017 |  |
| BMI | rs11688816 | G | 0.017 |  |
| BMI | rs1808579 | C | 0.017 |  |
| BMI | rs6477694 | C | 0.017 |  |
| Breast cancer | rs11571833 | T | 1.39 |  |
| Breast cancer | rs132390 | C | 1.36 |  |
| Breast cancer | rs11814448 | C | 1.35 |  |
| Breast cancer | rs2981579 | A | 1.35 |  |
| Breast cancer | rs614367 | T | 1.31 | HWE p < 1e-6 |
| Breast cancer | rs3803662 | A | 1.28 |  |
| Breast cancer | rs10771399 | A | 1.22 |  |
| Breast cancer | rs3757318 | A | 1.21 |  |
| Breast cancer | rs13387042 | A | 1.2 |  |
| Breast cancer | rs10995190 | G | 1.19 |  |
| Breast cancer | rs10941679 | G | 1.17 |  |
| Breast cancer | rs2943559 | G | 1.17 |  |
| Breast cancer | rs6001930 | C | 1.17 |  |
| Breast cancer | rs889312 | C | 1.16 |  |
| Breast cancer | rs999737 | C | 1.15 |  |
| Breast cancer | rs11249433 | G | 1.14 |  |
| Breast cancer | rs13329835 | G | 1.14 |  |
| Breast cancer | rs2236007 | G | 1.14 |  |
| Breast cancer | rs4973768 | T | 1.14 |  |
| Breast cancer | rs6472903 | T | 1.14 |  |
| Breast cancer | rs11780156 | T | 1.13 |  |
| Breast cancer | rs17356907 | A | 1.12 |  |
| Breast cancer | rs6828523 | C | 1.12 |  |
| Breast cancer | rs704010 | T | 1.12 |  |
| Breast cancer | rs865686 | T | 1.12 |  |
| Breast cancer | rs12422552 | C | 1.11 |  |
| Breast cancer | rs1353747 | T | 1.11 |  |
| Breast cancer | rs2046210 | A | 1.11 |  |
| Breast cancer | rs4849887 | C | 1.11 |  |
| Breast cancer | rs7072776 | A | 1.11 |  |
| Breast cancer | rs1011970 | T | 1.1 |  |
| Breast cancer | rs1292011 | A | 1.1 |  |
| Breast cancer | rs13281615 | G | 1.1 |  |
| Breast cancer | rs1550623 | A | 1.1 |  |
| Breast cancer | rs527616 | G | 1.1 |  |
| Breast cancer | rs6504950 | G | 1.1 |  |
| Breast cancer | rs16857609 | T | 1.09 |  |
| Breast cancer | rs17530068 | G | 1.09 |  |
| Breast cancer | rs2823093 | G | 1.09 |  |
| Breast cancer | rs3903072 | G | 1.09 |  |
| Breast cancer | rs9790517 | T | 1.09 |  |
| Breast cancer | rs11552449 | T | 1.08 |  |
| Breast cancer | rs11820646 | C | 1.08 |  |
| Breast cancer | rs1436904 | T | 1.08 |  |
| Breast cancer | rs720475 | G | 1.08 |  |
| Breast cancer | rs10759243 | A | 1.07 |  |
| Breast cancer | rs2588809 | T | 1.07 |  |
| Breast cancer | rs9693444 | A | 1.07 |  |
| Breast cancer | rs10472076 | C | 1.06 |  |
| Breast cancer | rs11199914 | C | 1.06 |  |
| Breast cancer | rs1432679 | C | 1.06 |  |
| Breast cancer | rs204247 | G | 1.06 |  |
| Breast cancer | rs3760982 | A | 1.06 |  |
| Breast cancer | rs3817198 | C | 1.06 |  |
| Breast cancer | rs4808801 | A | 1.06 |  |
| Breast cancer | rs616488 | A | 1.06 |  |
| Breast cancer | rs6762644 | G | 1.06 |  |
| Breast cancer | rs7904519 | G | 1.06 |  |
| Breast cancer | rs17817449 | T | 1.05 |  |
| Breast cancer | rs2016394 | G | 1.05 |  |
| Breast cancer | rs2284378 | C | 1.05 |  |
| Breast cancer | rs941764 | G | 1.05 |  |
| Breast cancer | rs10069690 | T | 1.04 |  |
| Breast cancer | rs12493607 | C | 1.04 |  |
| Breast cancer | rs11242675 | T | 1.03 |  |
| Breast cancer | rs8170 | A | 1.03 |  |
| Coronary Artery Disease (CAD) | rs55730499 | T | 1.37 |  |
| Coronary Artery Disease (CAD) | rs4252185 | C | 1.34 | R^2 = 0.65 with rs55730499 |
| Coronary Artery Disease (CAD) | rs2891168 | G | 1.21 |  |
| Coronary Artery Disease (CAD) | rs180803 | G | 1.2 |  |
| Coronary Artery Disease (CAD) | rs577594671 | T | 1.15 | INCLUDED - SNP is proxy for reported indel: chr2:203828796:I -- match within 1bp |
| Coronary Artery Disease (CAD) | rs9349379 | G | 1.14 |  |
| Coronary Artery Disease (CAD) | rs3918226 | T | 1.14 |  |
| Coronary Artery Disease (CAD) | rs56289821 | G | 1.14 |  |
| Coronary Artery Disease (CAD) | rs28451064 | A | 1.14 |  |
| Coronary Artery Disease (CAD) | rs9970807 | C | 1.13 |  |
| Coronary Artery Disease (CAD) | rs7528419 | A | 1.12 |  |
| Coronary Artery Disease (CAD) | rs17678683 | G | 1.1 |  |
| Coronary Artery Disease (CAD) | rs8042271 | G | 1.1 |  |
| Coronary Artery Disease (CAD) | rs4420638 | G | 1.1 |  |
| Coronary Artery Disease (CAD) | rs16986953 | A | 1.09 |  |
| Coronary Artery Disease (CAD) | rs11206510 | T | 1.08 |  |
| Coronary Artery Disease (CAD) | rs67180937 | G | 1.08 |  |
| Coronary Artery Disease (CAD) | rs139016349 | TTTC | 1.08 | INCLUDED - SNP is proxy for reported indel: chr3:138099161:I -- match in dbSNP |
| Coronary Artery Disease (CAD) | rs2107595 | A | 1.08 |  |
| Coronary Artery Disease (CAD) | rs11556924 | C | 1.08 |  |
| Coronary Artery Disease (CAD) | rs2519093 | T | 1.08 |  |
| Coronary Artery Disease (CAD) | rs1870634 | G | 1.08 |  |
| Coronary Artery Disease (CAD) | rs11191416 | T | 1.08 |  |
| Coronary Artery Disease (CAD) | rs2681472 | G | 1.08 |  |
| Coronary Artery Disease (CAD) | rs4468572 | C | 1.08 |  |
| Coronary Artery Disease (CAD) | rs7212798 | C | 1.08 |  |
| Coronary Artery Disease (CAD) | rs34908258 | CT | 1.07 | INCLUDED - SNP is proxy for reported indel: chr2:21378433:D -- 2bp away in Biobank, but only 1 in dbSNP. Probably a match |
| Coronary Artery Disease (CAD) | rs4593108 | C | 1.07 |  |
| Coronary Artery Disease (CAD) | rs72689147 | G | 1.07 |  |
| Coronary Artery Disease (CAD) | rs56336142 | T | 1.07 |  |
| Coronary Artery Disease (CAD) | rs12202017 | A | 1.07 |  |
| Coronary Artery Disease (CAD) | rs1412444 | T | 1.07 |  |
| Coronary Artery Disease (CAD) | rs2128739 | A | 1.07 |  |
| Coronary Artery Disease (CAD) | rs3184504 | T | 1.07 |  |
| Coronary Artery Disease (CAD) | rs11838776 | A | 1.07 |  |
| Coronary Artery Disease (CAD) | rs56062135 | C | 1.07 |  |
| Coronary Artery Disease (CAD) | rs6689306 | A | 1.06 |  |
| Coronary Artery Disease (CAD) |  | I | 1.06 | chr2:44074126:D -- no indels within 3bp of position in dbSNP |
| Coronary Artery Disease (CAD) | rs7568458 | A | 1.06 |  |
| Coronary Artery Disease (CAD) | rs17087335 | T | 1.06 |  |
| Coronary Artery Disease (CAD) | rs2487928 | A | 1.06 |  |
| Coronary Artery Disease (CAD) | rs10840293 | A | 1.06 |  |
| Coronary Artery Disease (CAD) | rs10139550 | G | 1.06 |  |
| Coronary Artery Disease (CAD) | rs663129 | A | 1.06 |  |
| Coronary Artery Disease (CAD) no LDL, HDL or TG | rs2891168 | G | 1.21 |  |
| Coronary Artery Disease (CAD) no LDL, HDL or TG | rs9349379 | G | 1.14 |  |
| Coronary Artery Disease (CAD) no LDL, HDL or TG | rs3918226 | T | 1.14 |  |
| Coronary Artery Disease (CAD) no LDL, HDL or TG | rs9970807 | C | 1.13 |  |
| Coronary Artery Disease (CAD) no LDL, HDL or TG | rs17678683 | G | 1.1 |  |
| Coronary Artery Disease (CAD) no LDL, HDL or TG | rs16986953 | A | 1.09 |  |
| Coronary Artery Disease (CAD) no LDL, HDL or TG | rs11191416 | T | 1.08 |  |
| Coronary Artery Disease (CAD) no LDL, HDL or TG | rs2681472 | G | 1.08 |  |
| Coronary Artery Disease (CAD) no LDL, HDL or TG | rs4468572 | C | 1.08 |  |
| Coronary Artery Disease (CAD) no LDL, HDL or TG | rs7212798 | C | 1.08 |  |
| Coronary Artery Disease (CAD) no LDL, HDL or TG | rs2107595 | A | 1.08 |  |
| Coronary Artery Disease (CAD) no LDL, HDL or TG | rs67180937 | G | 1.08 |  |
| Coronary Artery Disease (CAD) no LDL, HDL or TG | rs72689147 | G | 1.07 |  |
| Coronary Artery Disease (CAD) no LDL, HDL or TG | rs1412444 | T | 1.07 |  |
| Coronary Artery Disease (CAD) no LDL, HDL or TG | rs12202017 | A | 1.07 |  |
| Coronary Artery Disease (CAD) no LDL, HDL or TG | rs56062135 | C | 1.07 |  |
| Coronary Artery Disease (CAD) no LDL, HDL or TG | rs2128739 | A | 1.07 |  |
| Coronary Artery Disease (CAD) no LDL, HDL or TG | rs4593108 | C | 1.07 |  |
| Coronary Artery Disease (CAD) no LDL, HDL or TG | rs56336142 | T | 1.07 |  |
| Coronary Artery Disease (CAD) no LDL, HDL or TG | rs2487928 | A | 1.06 |  |
| Coronary Artery Disease (CAD) no LDL, HDL or TG | rs7568458 | A | 1.06 |  |
| Coronary Artery Disease (CAD) no LDL, HDL or TG | rs17087335 | T | 1.06 |  |
| Coronary Artery Disease (CAD) no LDL, HDL or TG | rs6689306 | A | 1.06 |  |
| Crohn's disease | rs11742570 | G | 1.28 |  |
| Crohn's disease | rs12994997 | A | 1.25 |  |
| Crohn's disease | rs2413583 | G | 1.23 |  |
| Crohn's disease | rs1505992 | A | 1.22 |  |
| Crohn's disease | rs17622378 | G | 1.21 | HWE p < 1e-6 |
| Crohn's disease | rs10761659 | G | 1.2 |  |
| Crohn's disease | rs13126505 | A | 1.2 |  |
| Crohn's disease | rs6679677 | C | 1.2 |  |
| Crohn's disease | rs2155219 | A | 1.19 |  |
| Crohn's disease | rs2361755 | G | 1.19 |  |
| Crohn's disease | rs4409764 | A | 1.19 |  |
| Crohn's disease | rs56167332 | A | 1.19 |  |
| Crohn's disease | rs10781499 | A | 1.18 |  |
| Crohn's disease | rs13300218 | G | 1.18 |  |
| Crohn's disease | rs1893217 | G | 1.18 |  |
| Crohn's disease | rs3024505 | A | 1.18 |  |
| Crohn's disease | rs3197999 | A | 1.17 |  |
| Crohn's disease | rs6556412 | A | 1.17 |  |
| Crohn's disease | rs8005161 | A | 1.17 |  |
| Crohn's disease | rs11554257 | G | 1.16 |  |
| Crohn's disease | rs116392568 | G | 1.16 |  |
| Crohn's disease | rs11681525 | G | 1.16 |  |
| Crohn's disease | rs2024092 | A | 1.16 |  |
| Crohn's disease | rs6651252 | A | 1.16 |  |
| Crohn's disease | rs7554511 | C | 1.16 |  |
| Crohn's disease | rs75900472 | C | 1.16 |  |
| Crohn's disease | rs9313808 | G | 1.16 |  |
| Crohn's disease | rs11743851 | G | 1.15 |  |
| Crohn's disease | rs11879191 | G | 1.15 |  |
| Crohn's disease | rs12720356 | C | 1.15 |  |
| Crohn's disease | rs13204742 | A | 1.15 |  |
| Crohn's disease | rs2823286 | G | 1.15 |  |
| Crohn's disease | rs3091315 | A | 1.15 |  |
| Crohn's disease | rs3764147 | G | 1.15 |  |
| Crohn's disease | rs4246905 | G | 1.15 |  |
| Crohn's disease | rs11010067 | G | 1.14 |  |
| Crohn's disease | rs12722515 | C | 1.14 |  |
| Crohn's disease | rs13300483 | A | 1.14 |  |
| Crohn's disease | rs17293632 | A | 1.14 |  |
| Crohn's disease | rs224090 | A | 1.14 | HWE p < 1e-6 |
| Crohn's disease | rs3851228 | A | 1.14 |  |
| Crohn's disease | rs423674 | C | 1.14 |  |
| Crohn's disease | rs6588248 | C | 1.14 |  |
| Crohn's disease | rs6716753 | G | 1.14 |  |
| Crohn's disease | rs7097656 | G | 1.14 |  |
| Crohn's disease | rs7517810 | A | 1.14 |  |
| Crohn's disease | rs7746082 | C | 1.14 |  |
| Crohn's disease | rs10495903 | A | 1.13 |  |
| Crohn's disease | rs12946510 | A | 1.13 |  |
| Crohn's disease | rs26528 | G | 1.13 |  |
| Crohn's disease | rs4256159 | A | 1.13 |  |
| Crohn's disease | rs7282490 | G | 1.13 |  |
| Crohn's disease | rs7555082 | A | 1.13 |  |
| Crohn's disease | rs7608910 | G | 1.13 |  |
| Crohn's disease | rs1260326 | A | 1.12 |  |
| Crohn's disease | rs12627970 | G | 1.12 |  |
| Crohn's disease | rs13407913 | G | 1.12 |  |
| Crohn's disease | rs17391694 | G | 1.12 |  |
| Crohn's disease | rs1819333 | A | 1.12 |  |
| Crohn's disease | rs2488389 | A | 1.12 |  |
| Crohn's disease | rs2641348 | A | 1.12 |  |
| Crohn's disease | rs2945412 | A | 1.12 |  |
| Crohn's disease | rs4768236 | C | 1.12 |  |
| Crohn's disease | rs516246 | A | 1.12 |  |
| Crohn's disease | rs6708413 | G | 1.12 |  |
| Crohn's disease | rs921720 | G | 1.12 |  |
| Crohn's disease | rs9868809 | A | 1.12 |  |
| Crohn's disease | rs113653754 | C | 1.11 |  |
| Crohn's disease | rs1199103 | A | 1.11 |  |
| Crohn's disease | rs1250546 | A | 1.11 |  |
| Crohn's disease | rs12942547 | A | 1.11 |  |
| Crohn's disease | rs1363907 | A | 1.11 |  |
| Crohn's disease | rs1569328 | G | 1.11 |  |
| Crohn's disease | rs16967103 | G | 1.11 |  |
| Crohn's disease | rs1842076 | A | 1.11 |  |
| Crohn's disease | rs212388 | G | 1.11 |  |
| Crohn's disease | rs2256609 | G | 1.11 |  |
| Crohn's disease | rs2284553 | G | 1.11 |  |
| Crohn's disease | rs2836878 | G | 1.11 |  |
| Crohn's disease | rs3766606 | C | 1.11 |  |
| Crohn's disease | rs6062504 | G | 1.11 |  |
| Crohn's disease | rs6908425 | G | 1.11 |  |
| Crohn's disease | rs925255 | G | 1.11 |  |
| Crohn's disease | rs10051722 | A | 1.1 |  |
| Crohn's disease | rs10065637 | G | 1.1 |  |
| Crohn's disease | rs10486483 | A | 1.1 |  |
| Crohn's disease | rs1292053 | G | 1.1 |  |
| Crohn's disease | rs1456896 | A | 1.1 |  |
| Crohn's disease | rs2227551 | A | 1.1 |  |
| Crohn's disease | rs3742130 | G | 1.1 |  |
| Crohn's disease | rs395157 | A | 1.1 |  |
| Crohn's disease | rs4802307 | C | 1.1 |  |
| Crohn's disease | rs559928 | G | 1.1 |  |
| Crohn's disease | rs6074022 | G | 1.1 |  |
| Crohn's disease | rs6740462 | A | 1.1 |  |
| Crohn's disease | rs6863411 | T | 1.1 |  |
| Crohn's disease | rs71559680 | G | 1.1 |  |
| Crohn's disease | rs7236492 | G | 1.1 |  |
| Crohn's disease | rs727563 | G | 1.1 |  |
| Crohn's disease | rs72810983 | A | 1.1 |  |
| Crohn's disease | rs10061469 | A | 1.09 |  |
| Crohn's disease | rs11230563 | G | 1.09 |  |
| Crohn's disease | rs1517352 | C | 1.09 |  |
| Crohn's disease | rs17119 | A | 1.09 |  |
| Crohn's disease | rs174537 | A | 1.09 |  |
| Crohn's disease | rs1801274 | A | 1.09 |  |
| Crohn's disease | rs1847472 | C | 1.09 |  |
| Crohn's disease | rs2930047 | G | 1.09 |  |
| Crohn's disease | rs35320439 | G | 1.09 |  |
| Crohn's disease | rs3853824 | G | 1.09 |  |
| Crohn's disease | rs568617 | A | 1.09 |  |
| Crohn's disease | rs5763767 | A | 1.09 |  |
| Crohn's disease | rs7015630 | A | 1.09 |  |
| Crohn's disease | rs7438704 | G | 1.09 |  |
| Crohn's disease | rs7657746 | A | 1.09 |  |
| Crohn's disease | rs7773324 | A | 1.09 |  |
| Crohn's disease | rs7954567 | A | 1.09 |  |
| Crohn's disease | rs1042058 | G | 1.08 |  |
| Crohn's disease | rs10798069 | C | 1.08 |  |
| Crohn's disease | rs10865331 | A | 1.08 |  |
| Crohn's disease | rs11641184 | A | 1.08 |  |
| Crohn's disease | rs12718244 | A | 1.08 |  |
| Crohn's disease | rs13204048 | A | 1.08 |  |
| Crohn's disease | rs17694108 | A | 1.08 |  |
| Crohn's disease | rs2538470 | A | 1.08 |  |
| Crohn's disease | rs4703855 | G | 1.08 | HWE p < 1e-6 |
| Crohn's disease | rs670523 | A | 1.08 |  |
| Crohn's disease | rs7758080 | G | 1.08 |  |
| Crohn's disease | rs864745 | A | 1.08 |  |
| Crohn's disease | rs9297145 | C | 1.08 |  |
| Crohn's disease | rs9358372 | G | 1.08 |  |
| Crohn's disease | rs9525625 | A | 1.08 |  |
| Crohn's disease | rs10185424 | A | 1.07 |  |
| Crohn's disease | rs2382817 | A | 1.07 |  |
| Crohn's disease | rs259964 | A | 1.07 |  |
| Crohn's disease | rs314313 | G | 1.07 |  |
| Crohn's disease | rs4976646 | G | 1.07 |  |
| Crohn's disease | rs915286 | A | 1.06 |  |
| Forced Vital Capacity | rs6923462 | T | 30.883 |  |
| Forced Vital Capacity | rs2863171 | C | 23.924 |  |
| Forced Vital Capacity | rs6501431 | T | 23.053 |  |
| Forced Vital Capacity | rs1430193 | A | 21.125 |  |
| Forced Vital Capacity | rs4237643 | G | 16.666 |  |
| Forced Vital Capacity | rs1079572 | G | 16.258 |  |
| HDL | rs3764261 | A | 0.2412 |  |
| HDL | rs12678919 | G | 0.1554 |  |
| HDL | rs1800961 | C | 0.127 |  |
| HDL | rs1532085 | A | 0.1068 |  |
| HDL | rs7241918 | T | 0.0902 |  |
| HDL | rs16942887 | A | 0.0831 |  |
| HDL | rs9987289 | G | 0.0817 |  |
| HDL | rs13107325 | C | 0.0708 |  |
| HDL | rs1883025 | C | 0.0698 |  |
| HDL | rs4420638 | A | 0.0669 |  |
| HDL | rs6065906 | T | 0.0594 |  |
| HDL | rs737337 | T | 0.0565 |  |
| HDL | rs4759375 | T | 0.056 |  |
| HDL | rs3136441 | C | 0.0545 |  |
| HDL | rs12748152 | C | 0.0506 |  |
| HDL | rs838880 | C | 0.0484 |  |
| HDL | rs386000 | C | 0.0479 |  |
| HDL | rs4846914 | A | 0.0479 |  |
| HDL | rs12328675 | C | 0.0447 |  |
| HDL | rs581080 | C | 0.0419 |  |
| HDL | rs17145738 | T | 0.0408 |  |
| HDL | rs2954029 | T | 0.0401 |  |
| HDL | rs174546 | C | 0.0391 |  |
| HDL | rs17173637 | T | 0.0363 |  |
| HDL | rs7134594 | T | 0.0354 |  |
| HDL | rs4660293 | A | 0.0353 |  |
| HDL | rs2925979 | C | 0.0351 |  |
| HDL | rs1689800 | A | 0.0344 |  |
| HDL | rs11246602 | C | 0.034 |  |
| HDL | rs4765127 | T | 0.0324 |  |
| HDL | rs2972146 | G | 0.0323 |  |
| HDL | rs11869286 | C | 0.0319 |  |
| HDL | rs7255436 | A | 0.0316 |  |
| HDL | rs2293889 | G | 0.0312 |  |
| HDL | rs2290547 | G | 0.0297 |  |
| HDL | rs4731702 | T | 0.0294 |  |
| HDL | rs17695224 | G | 0.029 |  |
| HDL | rs13326165 | A | 0.0289 |  |
| HDL | rs2652834 | G | 0.0285 |  |
| HDL | rs11613352 | T | 0.0281 |  |
| HDL | rs605066 | T | 0.0281 |  |
| HDL | rs4148008 | C | 0.028 |  |
| HDL | rs10019888 | A | 0.027 |  |
| HDL | rs1047891 | C | 0.0269 |  |
| HDL | rs4142995 | G | 0.0263 |  |
| HDL | rs499974 | C | 0.0263 |  |
| HDL | rs12967135 | G | 0.0262 |  |
| HDL | rs998584 | C | 0.026 |  |
| HDL | rs970548 | C | 0.0258 |  |
| HDL | rs2923084 | A | 0.0256 |  |
| HDL | rs2013208 | T | 0.0254 |  |
| HDL | rs6450176 | G | 0.0254 |  |
| HDL | rs3822072 | G | 0.0251 |  |
| HDL | rs2606736 | C | 0.0246 |  |
| HDL | rs702485 | G | 0.0243 |  |
| HDL | rs4129767 | A | 0.0237 |  |
| HDL | rs12801636 | A | 0.0235 |  |
| HDL | rs4917014 | G | 0.0222 |  |
| HDL | rs731839 | A | 0.022 |  |
| HDL | rs4650994 | G | 0.021 |  |
| HDL | rs7134375 | A | 0.0207 |  |
| HDL | rs12145743 | G | 0.0203 |  |
| HDL | rs1936800 | C | 0.02 |  |
| HDL | rs6805251 | T | 0.02 |  |
| HDL | rs4983559 | G | 0.0197 |  |
| HDL | rs1121980 | G | 0.0196 |  |
| HDL | rs2602836 | A | 0.0192 |  |
| Inflammatory Bowel Disease | rs80174646 | C | 1.89 |  |
| Inflammatory Bowel Disease | rs12422544 | G | 1.35 |  |
| Inflammatory Bowel Disease | rs7517847 | A | 1.28 |  |
| Inflammatory Bowel Disease | rs113653754 | C | 1.22 |  |
| Inflammatory Bowel Disease | rs11741861 | G | 1.22 |  |
| Inflammatory Bowel Disease | rs3024505 | A | 1.22 |  |
| Inflammatory Bowel Disease | rs34856868 | G | 1.22 |  |
| Inflammatory Bowel Disease | rs11742570 | G | 1.19 |  |
| Inflammatory Bowel Disease | rs2413583 | G | 1.19 |  |
| Inflammatory Bowel Disease | rs6025 | G | 1.19 |  |
| Inflammatory Bowel Disease | rs2836878 | G | 1.18 |  |
| Inflammatory Bowel Disease | rs3197999 | A | 1.18 |  |
| Inflammatory Bowel Disease | rs4409764 | A | 1.18 |  |
| Inflammatory Bowel Disease | rs7554511 | C | 1.18 |  |
| Inflammatory Bowel Disease | rs10781499 | A | 1.17 |  |
| Inflammatory Bowel Disease | rs56167332 | A | 1.17 |  |
| Inflammatory Bowel Disease | rs10761659 | G | 1.16 |  |
| Inflammatory Bowel Disease | rs12720356 | C | 1.16 |  |
| Inflammatory Bowel Disease | rs13300218 | G | 1.16 |  |
| Inflammatory Bowel Disease | rs2155219 | A | 1.16 |  |
| Inflammatory Bowel Disease | rs3851228 | A | 1.16 |  |
| Inflammatory Bowel Disease | rs75900472 | C | 1.16 |  |
| Inflammatory Bowel Disease | rs11554257 | G | 1.15 |  |
| Inflammatory Bowel Disease | rs12994997 | A | 1.15 |  |
| Inflammatory Bowel Disease | rs17061048 | A | 1.15 |  |
| Inflammatory Bowel Disease | rs17622378 | G | 1.15 | HWE p < 1e-6 |
| Inflammatory Bowel Disease | rs1893217 | G | 1.15 |  |
| Inflammatory Bowel Disease | rs2361755 | G | 1.15 |  |
| Inflammatory Bowel Disease | rs8005161 | A | 1.15 |  |
| Inflammatory Bowel Disease | rs9313808 | G | 1.15 |  |
| Inflammatory Bowel Disease | rs9868809 | A | 1.15 |  |
| Inflammatory Bowel Disease | rs12946510 | A | 1.14 |  |
| Inflammatory Bowel Disease | rs13126505 | A | 1.14 |  |
| Inflammatory Bowel Disease | rs1505992 | A | 1.14 |  |
| Inflammatory Bowel Disease | rs1801274 | A | 1.14 |  |
| Inflammatory Bowel Disease | rs4246905 | G | 1.14 |  |
| Inflammatory Bowel Disease | rs4845604 | G | 1.14 |  |
| Inflammatory Bowel Disease | rs6426833 | A | 1.14 |  |
| Inflammatory Bowel Disease | rs6556412 | A | 1.13 |  |
| Inflammatory Bowel Disease | rs7608910 | G | 1.13 |  |
| Inflammatory Bowel Disease | rs11879191 | G | 1.12 |  |
| Inflammatory Bowel Disease | rs12627970 | G | 1.12 |  |
| Inflammatory Bowel Disease | rs2823286 | G | 1.12 |  |
| Inflammatory Bowel Disease | rs3749171 | A | 1.12 |  |
| Inflammatory Bowel Disease | rs3766606 | C | 1.12 |  |
| Inflammatory Bowel Disease | rs7282490 | G | 1.12 |  |
| Inflammatory Bowel Disease | rs10495903 | A | 1.11 |  |
| Inflammatory Bowel Disease | rs11010067 | G | 1.11 |  |
| Inflammatory Bowel Disease | rs11681525 | G | 1.11 |  |
| Inflammatory Bowel Disease | rs11743851 | G | 1.11 |  |
| Inflammatory Bowel Disease | rs17293632 | A | 1.11 |  |
| Inflammatory Bowel Disease | rs2024092 | A | 1.11 |  |
| Inflammatory Bowel Disease | rs3091315 | A | 1.11 |  |
| Inflammatory Bowel Disease | rs6062504 | G | 1.11 |  |
| Inflammatory Bowel Disease | rs7134472 | A | 1.11 |  |
| Inflammatory Bowel Disease | rs7746082 | C | 1.11 |  |
| Inflammatory Bowel Disease | rs113010081 | G | 1.1 |  |
| Inflammatory Bowel Disease | rs116392568 | G | 1.1 |  |
| Inflammatory Bowel Disease | rs12722515 | C | 1.1 |  |
| Inflammatory Bowel Disease | rs12942547 | A | 1.1 |  |
| Inflammatory Bowel Disease | rs13204742 | A | 1.1 |  |
| Inflammatory Bowel Disease | rs13300483 | A | 1.1 |  |
| Inflammatory Bowel Disease | rs13407913 | G | 1.1 |  |
| Inflammatory Bowel Disease | rs224090 | A | 1.1 | HWE p < 1e-6 |
| Inflammatory Bowel Disease | rs2488389 | A | 1.1 |  |
| Inflammatory Bowel Disease | rs26528 | G | 1.1 |  |
| Inflammatory Bowel Disease | rs2816958 | G | 1.1 |  |
| Inflammatory Bowel Disease | rs3764147 | G | 1.1 |  |
| Inflammatory Bowel Disease | rs3806308 | G | 1.1 |  |
| Inflammatory Bowel Disease | rs395157 | A | 1.1 |  |
| Inflammatory Bowel Disease | rs559928 | G | 1.1 |  |
| Inflammatory Bowel Disease | rs6651252 | A | 1.1 |  |
| Inflammatory Bowel Disease | rs6920220 | A | 1.1 |  |
| Inflammatory Bowel Disease | rs7097656 | G | 1.1 |  |
| Inflammatory Bowel Disease | rs7555082 | A | 1.1 |  |
| Inflammatory Bowel Disease | rs10185424 | A | 1.09 |  |
| Inflammatory Bowel Disease | rs11229555 | C | 1.09 |  |
| Inflammatory Bowel Disease | rs11230563 | G | 1.09 |  |
| Inflammatory Bowel Disease | rs1199103 | A | 1.09 |  |
| Inflammatory Bowel Disease | rs12103 | A | 1.09 |  |
| Inflammatory Bowel Disease | rs1250546 | A | 1.09 |  |
| Inflammatory Bowel Disease | rs12568930 | A | 1.09 |  |
| Inflammatory Bowel Disease | rs1456896 | A | 1.09 |  |
| Inflammatory Bowel Disease | rs1569328 | G | 1.09 |  |
| Inflammatory Bowel Disease | rs17085007 | G | 1.09 |  |
| Inflammatory Bowel Disease | rs17119 | A | 1.09 |  |
| Inflammatory Bowel Disease | rs17694108 | A | 1.09 |  |
| Inflammatory Bowel Disease | rs17780256 | A | 1.09 |  |
| Inflammatory Bowel Disease | rs1819333 | A | 1.09 |  |
| Inflammatory Bowel Disease | rs1842076 | A | 1.09 |  |
| Inflammatory Bowel Disease | rs2256609 | G | 1.09 |  |
| Inflammatory Bowel Disease | rs3172494 | A | 1.09 |  |
| Inflammatory Bowel Disease | rs3742130 | G | 1.09 |  |
| Inflammatory Bowel Disease | rs423674 | C | 1.09 |  |
| Inflammatory Bowel Disease | rs4256159 | A | 1.09 |  |
| Inflammatory Bowel Disease | rs6588248 | C | 1.09 |  |
| Inflammatory Bowel Disease | rs6708413 | G | 1.09 |  |
| Inflammatory Bowel Disease | rs6740462 | A | 1.09 |  |
| Inflammatory Bowel Disease | rs6863411 | T | 1.09 |  |
| Inflammatory Bowel Disease | rs6908425 | G | 1.09 |  |
| Inflammatory Bowel Disease | rs7011507 | G | 1.09 |  |
| Inflammatory Bowel Disease | rs7236492 | G | 1.09 |  |
| Inflammatory Bowel Disease | rs7657746 | A | 1.09 |  |
| Inflammatory Bowel Disease | rs921720 | G | 1.09 |  |
| Inflammatory Bowel Disease | rs941823 | G | 1.09 |  |
| Inflammatory Bowel Disease | rs10995235 | A | 1.08 |  |
| Inflammatory Bowel Disease | rs11641184 | A | 1.08 |  |
| Inflammatory Bowel Disease | rs1260326 | A | 1.08 |  |
| Inflammatory Bowel Disease | rs12718244 | A | 1.08 |  |
| Inflammatory Bowel Disease | rs1363907 | A | 1.08 |  |
| Inflammatory Bowel Disease | rs1517352 | C | 1.08 |  |
| Inflammatory Bowel Disease | rs1847472 | C | 1.08 |  |
| Inflammatory Bowel Disease | rs2227551 | A | 1.08 |  |
| Inflammatory Bowel Disease | rs2382817 | A | 1.08 |  |
| Inflammatory Bowel Disease | rs2930047 | G | 1.08 |  |
| Inflammatory Bowel Disease | rs4380874 | A | 1.08 |  |
| Inflammatory Bowel Disease | rs4656958 | G | 1.08 |  |
| Inflammatory Bowel Disease | rs4703855 | G | 1.08 | HWE p < 1e-6 |
| Inflammatory Bowel Disease | rs4768236 | C | 1.08 |  |
| Inflammatory Bowel Disease | rs4976646 | G | 1.08 |  |
| Inflammatory Bowel Disease | rs516246 | A | 1.08 |  |
| Inflammatory Bowel Disease | rs5763767 | A | 1.08 |  |
| Inflammatory Bowel Disease | rs6074022 | G | 1.08 |  |
| Inflammatory Bowel Disease | rs6716753 | G | 1.08 |  |
| Inflammatory Bowel Disease | rs913678 | A | 1.08 |  |
| Inflammatory Bowel Disease | rs925255 | G | 1.08 |  |
| Inflammatory Bowel Disease | rs1292053 | G | 1.07 |  |
| Inflammatory Bowel Disease | rs2538470 | A | 1.07 |  |
| Inflammatory Bowel Disease | rs259964 | A | 1.07 |  |
| Inflammatory Bowel Disease | rs314313 | G | 1.07 |  |
| Inflammatory Bowel Disease | rs9297145 | C | 1.07 |  |
| Inflammatory Bowel Disease | rs10051722 | A | 1.06 |  |
| Inflammatory Bowel Disease | rs1042058 | G | 1.06 |  |
| Inflammatory Bowel Disease | rs111781203 | A | 1.06 |  |
| Inflammatory Bowel Disease | rs1182188 | A | 1.06 |  |
| Inflammatory Bowel Disease | rs174537 | A | 1.06 |  |
| Inflammatory Bowel Disease | rs2111485 | A | 1.06 |  |
| Inflammatory Bowel Disease | rs2284553 | G | 1.06 |  |
| Inflammatory Bowel Disease | rs254560 | A | 1.06 |  |
| Inflammatory Bowel Disease | rs3853824 | G | 1.06 |  |
| Inflammatory Bowel Disease | rs4664304 | A | 1.06 |  |
| Inflammatory Bowel Disease | rs4692386 | G | 1.06 |  |
| Inflammatory Bowel Disease | rs4743820 | A | 1.06 |  |
| Inflammatory Bowel Disease | rs4802307 | C | 1.06 |  |
| Inflammatory Bowel Disease | rs561722 | G | 1.06 |  |
| Inflammatory Bowel Disease | rs653178 | G | 1.06 |  |
| Inflammatory Bowel Disease | rs6724516 | A | 1.06 |  |
| Inflammatory Bowel Disease | rs7015630 | A | 1.06 |  |
| Inflammatory Bowel Disease | rs71559680 | G | 1.06 |  |
| Inflammatory Bowel Disease | rs7240004 | A | 1.06 |  |
| Inflammatory Bowel Disease | rs727088 | G | 1.06 |  |
| Inflammatory Bowel Disease | rs72810983 | A | 1.06 |  |
| Inflammatory Bowel Disease | rs7773324 | A | 1.06 |  |
| Inflammatory Bowel Disease | rs7805114 | A | 1.06 |  |
| Inflammatory Bowel Disease | rs907611 | A | 1.06 |  |
| Inflammatory Bowel Disease | rs915286 | A | 1.06 |  |
| Inflammatory Bowel Disease | rs9358372 | G | 1.06 |  |
| Inflammatory Bowel Disease | rs2945412 | A | 1.05 |  |
| Inflammatory Bowel Disease | rs7404095 | G | 1.05 |  |
| LDL | rs4420638 | G | 0.2251 |  |
| LDL | rs6511720 | G | 0.2209 |  |
| LDL | rs629301 | T | 0.1669 |  |
| LDL | rs1367117 | A | 0.1186 |  |
| LDL | rs10401969 | T | 0.1184 |  |
| LDL | rs1801689 | C | 0.1028 |  |
| LDL | rs964184 | G | 0.0855 |  |
| LDL | rs4299376 | G | 0.0812 |  |
| LDL | rs5763662 | T | 0.0767 |  |
| LDL | rs12916 | C | 0.0733 |  |
| LDL | rs9987289 | G | 0.0714 |  |
| LDL | rs2000999 | A | 0.065 |  |
| LDL | rs2479409 | G | 0.0642 |  |
| LDL | rs1800562 | G | 0.0615 | HWE p < 1e-6 |
| LDL | rs11220462 | A | 0.059 |  |
| LDL | rs2954029 | A | 0.0564 |  |
| LDL | rs3764261 | C | 0.0528 |  |
| LDL | rs174546 | C | 0.0512 |  |
| LDL | rs10490626 | G | 0.0508 |  |
| LDL | rs12748152 | T | 0.0499 |  |
| LDL | rs2131925 | T | 0.0489 |  |
| LDL | rs1564348 | C | 0.0481 |  |
| LDL | rs6882076 | C | 0.0456 |  |
| LDL | rs3177928 | A | 0.0452 |  |
| LDL | rs11136341 | G | 0.0447 |  |
| LDL | rs3780181 | A | 0.0445 |  |
| LDL | rs6029526 | A | 0.0436 |  |
| LDL | rs7640978 | C | 0.0392 |  |
| LDL | rs4722551 | C | 0.0391 |  |
| LDL | rs2072183 | C | 0.0386 |  |
| LDL | rs3757354 | C | 0.0382 |  |
| LDL | rs1169288 | C | 0.0375 |  |
| LDL | rs514230 | T | 0.0364 |  |
| LDL | rs2642442 | T | 0.036 |  |
| LDL | rs11563251 | T | 0.0345 |  |
| LDL | rs12670798 | C | 0.0344 |  |
| LDL | rs17404153 | G | 0.0336 |  |
| LDL | rs267733 | A | 0.0331 |  |
| LDL | rs10102164 | A | 0.0316 |  |
| LDL | rs2081687 | T | 0.0311 |  |
| LDL | rs9488822 | A | 0.0311 | p>5e-8 -- still in this list because replication p=1e-7 |
| LDL | rs8017377 | A | 0.0303 |  |
| LDL | rs12027135 | T | 0.03 |  |
| LDL | rs2255141 | A | 0.0299 |  |
| LDL | rs2328223 | C | 0.0299 |  |
| LDL | rs7206971 | A | 0.0292 | p>5e-8 -- still in this list because replication p=3e-7 |
| LDL | rs4530754 | A | 0.0275 |  |
| LDL | rs2902940 | A | 0.0274 | HWE p < 1e-6 |
| LDL | rs11065987 | A | 0.0269 |  |
| LDL | rs364585 | G | 0.0249 |  |
| LDL | rs1250229 | C | 0.0243 | HWE p < 1e-6 |
| LDL | rs4942486 | T | 0.0243 |  |
| LDL | rs314253 | T | 0.0242 |  |
| LDL | rs2710642 | A | 0.0239 |  |
| LDL | rs2030746 | T | 0.0214 |  |
| Menopause age | rs75770066 | G | 0.9103 |  |
| Menopause age | rs16991615 | G | 0.88 |  |
| Menopause age | rs140267842 | A | 0.8044 |  |
| Menopause age | rs11668344 | G | 0.41 |  |
| Menopause age | rs1183272 | C | 0.31 |  |
| Menopause age | rs2277339 | G | 0.31 |  |
| Menopause age | rs2720044 | A | 0.29 |  |
| Menopause age | rs3741604 | T | 0.29 |  |
| Menopause age | rs2547274 | G | 0.28 |  |
| Menopause age | rs11031006 | G | 0.25 |  |
| Menopause age | rs365132 | G | 0.24 |  |
| Menopause age | rs349306 | G | 0.23 |  |
| Menopause age | rs4246511 | C | 0.22 |  |
| Menopause age | rs6899676 | A | 0.21 |  |
| Menopause age | rs451417 | A | 0.2 |  |
| Menopause age | rs4693089 | A | 0.2 |  |
| Menopause age | rs1054875 | T | 0.19 |  |
| Menopause age | rs1727326 | C | 0.19 |  |
| Menopause age | rs930036 | A | 0.19 |  |
| Menopause age | rs4886238 | G | 0.18 |  |
| Menopause age | rs12461110 | A | 0.17 |  |
| Menopause age | rs1800932 | A | 0.17 |  |
| Menopause age | rs10852344 | T | 0.16 |  |
| Menopause age | rs12196873 | A | 0.16 |  |
| Menopause age | rs13040088 | G | 0.16 |  |
| Menopause age | rs2230365 | C | 0.16 |  |
| Menopause age | rs2236553 | C | 0.16 |  |
| Menopause age | rs5762534 | T | 0.16 |  |
| Menopause age | rs6856693 | A | 0.16 |  |
| Menopause age | rs704795 | A | 0.16 |  |
| Menopause age | rs707938 | G | 0.16 |  |
| Menopause age | rs763121 | G | 0.16 |  |
| Menopause age | rs2236918 | C | 0.15 |  |
| Menopause age | rs8070740 | A | 0.15 |  |
| Menopause age | rs10957156 | A | 0.14 |  |
| Menopause age | rs12824058 | G | 0.14 |  |
| Menopause age | rs16858210 | G | 0.14 |  |
| Menopause age | rs1713460 | G | 0.14 |  |
| Menopause age | rs1799949 | G | 0.14 |  |
| Menopause age | rs2241584 | A | 0.14 |  |
| Menopause age | rs6484478 | G | 0.14 |  |
| Menopause age | rs9393800 | G | 0.14 |  |
| Menopause age | rs12142240 | T | 0.13 |  |
| Menopause age | rs1411478 | A | 0.13 |  |
| Menopause age | rs2941505 | A | 0.13 |  |
| Menopause age | rs427394 | G | 0.13 |  |
| Menopause age | rs551087 | G | 0.13 |  |
| Menopause age | rs7397861 | G | 0.13 |  |
| Menopause age | rs9796 | T | 0.13 |  |
| Menopause age | rs10734411 | A | 0.12 |  |
| Menopause age | rs11738223 | A | 0.12 |  |
| Menopause age | rs12599106 | A | 0.12 |  |
| Menopause age | rs4879656 | A | 0.12 |  |
| Menopause age | rs9039 | C | 0.12 |  |
| Menopause age | rs10905065 | A | 0.11 |  |
| Menopause age | rs7259376 | A | 0.11 |  |
| Prostate cancer | rs16901979 | A | 1.647 |  |
| Prostate cancer | rs1447295 | A | 1.427 |  |
| Prostate cancer | rs12621278 | A | 1.309 |  |
| Prostate cancer | rs6983267 | G | 1.254 |  |
| Prostate cancer | rs10993994 | T | 1.241 |  |
| Prostate cancer | rs4430796 | A | 1.235 |  |
| Prostate cancer | rs7127900 | A | 1.215 |  |
| Prostate cancer | rs1859962 | G | 1.205 |  |
| Prostate cancer | rs2735839 | G | 1.198 |  |
| Prostate cancer | rs7931342 | G | 1.192 |  |
| Prostate cancer | rs2242652 | G | 1.175 |  |
| Prostate cancer | rs620861 | G | 1.159 |  |
| Prostate cancer | rs5759167 | G | 1.157 |  |
| Prostate cancer | rs10086908 | T | 1.15 |  |
| Prostate cancer | rs7679673 | C | 1.15 |  |
| Prostate cancer | rs10486567 | G | 1.147 |  |
| Prostate cancer | rs902774 | A | 1.142 |  |
| Prostate cancer | rs76934034 | T | 1.14 |  |
| Prostate cancer | rs12543663 | C | 1.136 |  |
| Prostate cancer | rs1512268 | T | 1.136 |  |
| Prostate cancer | rs2660753 | T | 1.136 |  |
| Prostate cancer | rs8008270 | C | 1.134 |  |
| Prostate cancer | rs11649743 | G | 1.13 |  |
| Prostate cancer | rs9364554 | T | 1.13 |  |
| Prostate cancer | rs80130819 | A | 1.127 |  |
| Prostate cancer | rs12480328 | T | 1.125 | p>5e-8 -- still in this list because replication p<0.05 |
| Prostate cancer | rs11650494 | A | 1.12 | p>5e-8 -- still in this list because replication p<0.05 |
| Prostate cancer | rs10934853 | A | 1.119 |  |
| Prostate cancer | rs11672691 | G | 1.117 |  |
| Prostate cancer | rs6465657 | C | 1.117 |  |
| Prostate cancer | rs6062509 | T | 1.114 |  |
| Prostate cancer | rs721048 | A | 1.112 |  |
| Prostate cancer | rs10936632 | A | 1.111 |  |
| Prostate cancer | rs12155172 | A | 1.111 |  |
| Prostate cancer | rs11135910 | T | 1.109 |  |
| Prostate cancer | rs17021918 | C | 1.108 |  |
| Prostate cancer | rs1933488 | A | 1.1 |  |
| Prostate cancer | rs10875943 | C | 1.097 |  |
| Prostate cancer | rs17599629 | G | 1.097 |  |
| Prostate cancer | rs339331 | T | 1.096 |  |
| Prostate cancer | rs3771570 | T | 1.096 |  |
| Prostate cancer | rs684232 | C | 1.096 |  |
| Prostate cancer | rs17694493 | G | 1.095 |  |
| Prostate cancer | rs2273669 | G | 1.095 |  |
| Prostate cancer | rs7611694 | A | 1.095 |  |
| Prostate cancer | rs4245739 | A | 1.093 |  |
| Prostate cancer | rs1465618 | T | 1.092 |  |
| Prostate cancer | rs10009409 | T | 1.091 |  |
| Prostate cancer | rs8102476 | C | 1.091 |  |
| Prostate cancer | rs11568818 | T | 1.088 |  |
| Prostate cancer | rs12500426 | A | 1.088 |  |
| Prostate cancer | rs58133635 | T | 1.087 |  |
| Prostate cancer | rs10187424 | T | 1.086 |  |
| Prostate cancer | rs1894292 | G | 1.086 |  |
| Prostate cancer | rs1983891 | T | 1.086 | HWE p < 1e-6 |
| Prostate cancer | rs2238776 | G | 1.085 | p>5e-8 -- still in this list because replication p<0.05 |
| Prostate cancer | rs7153648 | C | 1.085 | p>5e-8 -- still in this list because replication p<0.05 |
| Prostate cancer | rs115457135 | A | 1.084 |  |
| Prostate cancer | rs1270884 | A | 1.084 |  |
| Prostate cancer | rs3850699 | A | 1.084 |  |
| Prostate cancer | rs115306967 | G | 1.081 |  |
| Prostate cancer | rs12653946 | T | 1.081 |  |
| Prostate cancer | rs2292884 | G | 1.08 |  |
| Prostate cancer | rs11214775 | G | 1.079 |  |
| Prostate cancer | rs7241993 | C | 1.077 |  |
| Prostate cancer | rs8014671 | G | 1.073 |  |
| Prostate cancer | rs2121875 | C | 1.072 |  |
| Prostate cancer | rs9443189 | A | 1.072 | p>5e-8 -- still in this list because replication p<0.05 |
| Prostate cancer | rs56232506 | A | 1.071 |  |
| Prostate cancer | rs7141529 | C | 1.071 |  |
| Prostate cancer | rs11902236 | T | 1.07 | p>5e-8 -- still in this list because replication p<0.05 |
| Prostate cancer | rs6869841 | T | 1.07 | p>5e-8 -- still in this list because replication p<0.05 |
| Prostate cancer | rs1218582 | G | 1.067 |  |
| Prostate cancer | rs4962416 | C | 1.067 | p>5e-8 -- still in this list because replication p<0.05 |
| Prostate cancer | rs9287719 | C | 1.067 |  |
| Prostate cancer | rs2427345 | C | 1.066 | p>5e-8 -- still in this list because replication p<0.05 |
| Prostate cancer | rs3096702 | A | 1.066 | p>5e-8 -- still in this list because replication p<0.05 |
| Prostate cancer | rs4713266 | C | 1.065 |  |
| Prostate cancer | rs2928679 | A | 1.064 |  |
| Prostate cancer | rs1041449 | G | 1.063 | p>5e-8 -- still in this list because replication p<0.05 |
| Prostate cancer | rs130067 | C | 1.063 | p>5e-8 -- still in this list because replication p<0.05 |
| Prostate cancer | rs13385191 | G | 1.057 | p>5e-8 -- still in this list because replication p<0.05 |
| Prostate cancer | rs6763931 | A | 1.057 | p>5e-8 -- still in this list because replication p<0.05 |
| Prostate cancer | rs12051443 | A | 1.055 | p>5e-8 -- still in this list because replication p<0.05 |
| Prostate cancer | rs1775148 | C | 1.055 | p>5e-8 -- still in this list because replication p<0.05 |
| Prostate cancer | rs636291 | A | 1.054 | p>5e-8 -- still in this list because replication p<0.05 |
| Prostate cancer | rs1571801 | T | 1.052 | p>5e-8 -- still in this list because replication p<0.05 |
| Stroke | rs17696736 | G | 0.0934 |  |
| Stroke | rs2023938 | C | 0.132 |  |
| Stroke | rs12646447 | C | 0.121 |  |
| Stroke | rs10744777 | T | 0.0925 |  |
| Systolic blood pressure | rs10850411 | T | 0.354 |  |
| Systolic blood pressure | rs11191548 | T | 1.095 |  |
| Systolic blood pressure | rs1173771 | G | 0.504 |  |
| Systolic blood pressure | rs11953630 | C | 0.412 |  |
| Systolic blood pressure | rs12940887 | T | 0.362 |  |
| Systolic blood pressure | rs13107325 | C | 0.981 |  |
| Systolic blood pressure | rs1327235 | G | 0.34 |  |
| Systolic blood pressure | rs1378942 | C | 0.613 |  |
| Systolic blood pressure | rs1458038 | T | 0.706 |  |
| Systolic blood pressure | rs17249754 | G | 0.928 |  |
| Systolic blood pressure | rs17367504 | A | 0.903 |  |
| Systolic blood pressure | rs17608766 | C | 0.556 |  |
| Systolic blood pressure | rs1799945 | G | 0.627 |  |
| Systolic blood pressure | rs1813353 | T | 0.569 |  |
| Systolic blood pressure | rs2521501 | T | 0.65 |  |
| Systolic blood pressure | rs2932538 | G | 0.388 |  |
| Systolic blood pressure | rs3184504 | T | 0.598 |  |
| Systolic blood pressure | rs381815 | T | 0.575 |  |
| Systolic blood pressure | rs419076 | T | 0.409 |  |
| Systolic blood pressure | rs4373814 | C | 0.373 |  |
| Systolic blood pressure | rs4590817 | G | 0.646 |  |
| Systolic blood pressure | rs6015450 | G | 0.896 |  |
| Systolic blood pressure | rs633185 | C | 0.565 |  |
| Systolic blood pressure | rs7129220 | A | 0.619 |  |
| Systolic blood pressure | rs805303 | G | 0.376 |  |
| Systolic blood pressure | rs932764 | G | 0.484 |  |
| Telomere Length | rs10936599 | C | 0.097 |  |
| Telomere Length | rs2736100 | C | 0.078 |  |
| Telomere Length | rs7675998 | G | 0.074 |  |
| Telomere Length | rs9420907 | C | 0.069 |  |
| Telomere Length | rs755017 | G | 0.062 |  |
| Telomere Length | rs11125529 | A | 0.056 |  |
| Telomere Length | rs8105767 | G | 0.048 |  |
| Triglycerides | rs964184 | G | 0.2341 |  |
| Triglycerides | rs12678919 | A | 0.1702 |  |
| Triglycerides | rs10401969 | T | 0.121 |  |
| Triglycerides | rs17145738 | C | 0.1149 |  |
| Triglycerides | rs1260326 | T | 0.1148 |  |
| Triglycerides | rs2412710 | A | 0.0988 |  |
| Triglycerides | rs2954029 | A | 0.0764 |  |
| Triglycerides | rs2929282 | T | 0.0725 |  |
| Triglycerides | rs2131925 | T | 0.0657 |  |
| Triglycerides | rs13238203 | C | 0.0592 | p>5e-8 -- still in this list because replication p=3e-6 |
| Triglycerides | rs6065906 | C | 0.0534 |  |
| Triglycerides | rs174546 | T | 0.0447 |  |
| Triglycerides | rs4846914 | G | 0.0401 |  |
| Triglycerides | rs1495741 | G | 0.0399 |  |
| Triglycerides | rs3764261 | C | 0.0395 |  |
| Triglycerides | rs9686661 | T | 0.0379 |  |
| Triglycerides | rs12748152 | T | 0.0372 |  |
| Triglycerides | rs1832007 | A | 0.0327 |  |
| Triglycerides | rs10761731 | A | 0.0312 |  |
| Triglycerides | rs1532085 | A | 0.031 |  |
| Triglycerides | rs442177 | T | 0.0309 |  |
| Triglycerides | rs645040 | T | 0.0293 |  |
| Triglycerides | rs998584 | A | 0.0293 |  |
| Triglycerides | rs4765127 | G | 0.0286 |  |
| Triglycerides | rs6882076 | C | 0.0286 |  |
| Triglycerides | rs2972146 | T | 0.0281 |  |
| Triglycerides | rs11613352 | C | 0.028 |  |
| Triglycerides | rs11649653 | C | 0.0274 | p>5e-8 -- still in this list because replication p=1e-7 |
| Triglycerides | rs4722551 | T | 0.0267 |  |
| Triglycerides | rs6831256 | G | 0.0258 |  |
| Triglycerides | rs8077889 | C | 0.0252 |  |
| Triglycerides | rs2068888 | G | 0.0241 |  |
| Triglycerides | rs731839 | G | 0.0224 |  |
| Triglycerides | rs7248104 | G | 0.0222 |  |
| Triglycerides | rs11776767 | C | 0.022 |  |
| Triglycerides | rs5756931 | T | 0.0203 |  |
| Triglycerides | rs3198697 | C | 0.0198 |  |
| Triglycerides | rs38855 | A | 0.0187 |  |
| Type 2 diabetes | rs10203174 | C | 0.131 |  |
| Type 2 diabetes | rs10758593 | A | 0.058 |  |
| Type 2 diabetes | rs10811661 | T | 0.166 |  |
| Type 2 diabetes | rs10842994 | C | 0.095 |  |
| Type 2 diabetes | rs10923931 | T | 0.077 |  |
| Type 2 diabetes | rs11063069 | G | 0.077 |  |
| Type 2 diabetes | rs1111875 | C | 0.104 |  |
| Type 2 diabetes | rs11257655 | T | 0.068 |  |
| Type 2 diabetes | rs11634397 | G | 0.049 |  |
| Type 2 diabetes | rs11717195 | T | 0.104 |  |
| Type 2 diabetes | rs12242953 | G | 0.068 |  |
| Type 2 diabetes | rs12427353 | G | 0.077 |  |
| Type 2 diabetes | rs12497268 | G | 0.03 |  |
| Type 2 diabetes | rs12571751 | A | 0.077 |  |
| Type 2 diabetes | rs12899811 | G | 0.077 |  |
| Type 2 diabetes | rs1359790 | G | 0.077 |  |
| Type 2 diabetes | rs1496653 | A | 0.086 |  |
| Type 2 diabetes | rs1552224 | A | 0.104 |  |
| Type 2 diabetes | rs163184 | G | 0.086 |  |
| Type 2 diabetes | rs16927668 | T | 0.039 |  |
| Type 2 diabetes | rs17168486 | T | 0.104 |  |
| Type 2 diabetes | rs17301514 | A | 0.049 |  |
| Type 2 diabetes | rs17791513 | A | 0.113 |  |
| Type 2 diabetes | rs17867832 | T | 0.086 |  |
| Type 2 diabetes | rs1801282 | C | 0.122 |  |
| Type 2 diabetes | rs2007084 | G | 0.02 |  |
| Type 2 diabetes | rs2075423 | G | 0.068 |  |
| Type 2 diabetes | rs2261181 | T | 0.122 |  |
| Type 2 diabetes | rs2334499 | T | 0.039 |  |
| Type 2 diabetes | rs243088 | T | 0.068 |  |
| Type 2 diabetes | rs2447090 | A | 0.039 |  |
| Type 2 diabetes | rs2796441 | G | 0.068 |  |
| Type 2 diabetes | rs3734621 | C | 0.068 |  |
| Type 2 diabetes | rs3802177 | G | 0.131 |  |
| Type 2 diabetes | rs4299828 | A | 0.039 |  |
| Type 2 diabetes | rs4402960 | T | 0.122 |  |
| Type 2 diabetes | rs4430796 | A | 0.095 |  |
| Type 2 diabetes | rs4458523 | G | 0.095 |  |
| Type 2 diabetes | rs4502156 | T | 0.058 |  |
| Type 2 diabetes | rs459193 | G | 0.077 |  |
| Type 2 diabetes | rs4812829 | A | 0.058 |  |
| Type 2 diabetes | rs516946 | C | 0.086 |  |
| Type 2 diabetes | rs5215 | C | 0.068 |  |
| Type 2 diabetes | rs6819243 | T | 0.068 |  |
| Type 2 diabetes | rs6878122 | G | 0.095 |  |
| Type 2 diabetes | rs7177055 | A | 0.077 |  |
| Type 2 diabetes | rs7202877 | T | 0.113 |  |
| Type 2 diabetes | rs7569522 | A | 0.048 |  |
| Type 2 diabetes | rs7756992 | G | 0.157 |  |
| Type 2 diabetes | rs7845219 | T | 0.058 |  |
| Type 2 diabetes | rs7903146 | T | 0.329 |  |
| Type 2 diabetes | rs7955901 | C | 0.068 |  |
| Type 2 diabetes | rs8108269 | G | 0.068 |  |
| Type 2 diabetes | rs8182584 | T | 0.039 |  |
| Type 2 diabetes | rs849135 | G | 0.104 |  |
| Type 2 diabetes | rs10278336 | A |  | Major fasting glucose SNP |
| Type 2 diabetes | rs10401969 | C |  | Associated with LDL, triglycerides and sphingolipids |
| Type 2 diabetes | rs10830963 | G |  | Major fasting glucose SNP |
| Type 2 diabetes | rs11651052 | A |  | SNP unavailable proxy rs4430796 used r2=0.97 |
| Type 2 diabetes | rs12970134 | A |  | Primary effect on BMI |
| Type 2 diabetes | rs13233731 | G |  | Associated with HDL |
| Type 2 diabetes | rs13389219 | C |  | In linkage disequilibrium with SNPs associated with waist hip ratio and triglycerides |
| Type 2 diabetes | rs2943640 | C |  | Associated with HDL, triglycerides and adiposity |
| Type 2 diabetes | rs6795735 | C |  | Associated with waist hip ratio |
| Type 2 diabetes | rs780094 | C |  | Associated with lots of traits |
| Type 2 diabetes | rs9936385 | C |  | Primary effect on BMI |
| Ulcerative colitis | rs80174646 | C | 1.61 |  |
| Ulcerative colitis | rs113653754 | C | 1.35 |  |
| Ulcerative colitis | rs6426833 | A | 1.27 |  |
| Ulcerative colitis | rs2836878 | G | 1.25 |  |
| Ulcerative colitis | rs3024505 | A | 1.25 |  |
| Ulcerative colitis | rs2816958 | G | 1.2 |  |
| Ulcerative colitis | rs3851228 | A | 1.2 |  |
| Ulcerative colitis | rs1801274 | A | 1.19 |  |
| Ulcerative colitis | rs3197999 | A | 1.19 |  |
| Ulcerative colitis | rs3806308 | G | 1.19 |  |
| Ulcerative colitis | rs4845604 | G | 1.18 |  |
| Ulcerative colitis | rs7554511 | C | 1.18 |  |
| Ulcerative colitis | rs12720356 | C | 1.17 |  |
| Ulcerative colitis | rs4409764 | A | 1.17 |  |
| Ulcerative colitis | rs7134472 | A | 1.17 |  |
| Ulcerative colitis | rs6920220 | A | 1.16 |  |
| Ulcerative colitis | rs7517847 | A | 1.16 |  |
| Ulcerative colitis | rs9868809 | A | 1.16 |  |
| Ulcerative colitis | rs13300218 | G | 1.15 |  |
| Ulcerative colitis | rs2413583 | G | 1.15 |  |
| Ulcerative colitis | rs3749171 | A | 1.15 |  |
| Ulcerative colitis | rs3766606 | C | 1.15 |  |
| Ulcerative colitis | rs56167332 | A | 1.15 |  |
| Ulcerative colitis | rs75900472 | C | 1.15 |  |
| Ulcerative colitis | rs10781499 | A | 1.14 |  |
| Ulcerative colitis | rs10799838 | A | 1.14 |  |
| Ulcerative colitis | rs113010081 | G | 1.14 |  |
| Ulcerative colitis | rs12568930 | A | 1.14 |  |
| Ulcerative colitis | rs12946510 | A | 1.14 |  |
| Ulcerative colitis | rs17085007 | G | 1.14 |  |
| Ulcerative colitis | rs4380874 | A | 1.14 |  |
| Ulcerative colitis | rs561722 | G | 1.14 |  |
| Ulcerative colitis | rs6724516 | A | 1.14 |  |
| Ulcerative colitis | rs7608910 | G | 1.14 |  |
| Ulcerative colitis | rs8005161 | A | 1.14 |  |
| Ulcerative colitis | rs9313808 | G | 1.14 |  |
| Ulcerative colitis | rs11554257 | G | 1.13 |  |
| Ulcerative colitis | rs1893217 | G | 1.13 |  |
| Ulcerative colitis | rs2155219 | A | 1.13 |  |
| Ulcerative colitis | rs10761659 | G | 1.12 |  |
| Ulcerative colitis | rs10995235 | A | 1.12 |  |
| Ulcerative colitis | rs17780256 | A | 1.12 |  |
| Ulcerative colitis | rs4246905 | G | 1.12 |  |
| Ulcerative colitis | rs7805114 | A | 1.12 |  |
| Ulcerative colitis | rs1182188 | A | 1.11 |  |
| Ulcerative colitis | rs12627970 | G | 1.11 |  |
| Ulcerative colitis | rs4812833 | A | 1.11 |  |
| Ulcerative colitis | rs7282490 | G | 1.11 |  |
| Ulcerative colitis | rs941823 | G | 1.11 |  |
| Ulcerative colitis | rs10185424 | A | 1.1 |  |
| Ulcerative colitis | rs12103 | A | 1.1 |  |
| Ulcerative colitis | rs17229285 | G | 1.1 |  |
| Ulcerative colitis | rs17694108 | A | 1.1 |  |
| Ulcerative colitis | rs2823286 | G | 1.1 |  |
| Ulcerative colitis | rs3774937 | G | 1.1 |  |
| Ulcerative colitis | rs4728142 | A | 1.1 |  |
| Ulcerative colitis | rs6556412 | A | 1.1 |  |
| Ulcerative colitis | rs7657746 | A | 1.1 |  |
| Ulcerative colitis | rs11229555 | C | 1.09 |  |
| Ulcerative colitis | rs11742570 | G | 1.09 |  |
| Ulcerative colitis | rs12942547 | A | 1.09 |  |
| Ulcerative colitis | rs1405108 | C | 1.09 |  |
| Ulcerative colitis | rs17622378 | G | 1.09 | HWE p < 1e-6 |
| Ulcerative colitis | rs17736589 | G | 1.09 |  |
| Ulcerative colitis | rs2111485 | A | 1.09 |  |
| Ulcerative colitis | rs395157 | A | 1.09 |  |
| Ulcerative colitis | rs4656958 | G | 1.09 |  |
| Ulcerative colitis | rs4743820 | A | 1.09 |  |
| Ulcerative colitis | rs483905 | A | 1.09 |  |
| Ulcerative colitis | rs6062504 | G | 1.09 |  |
| Ulcerative colitis | rs6667605 | G | 1.09 |  |
| Ulcerative colitis | rs7240004 | A | 1.09 |  |
| Ulcerative colitis | rs1077773 | A | 1.08 |  |
| Ulcerative colitis | rs11010067 | G | 1.08 |  |
| Ulcerative colitis | rs11150589 | A | 1.08 |  |
| Ulcerative colitis | rs111781203 | A | 1.08 |  |
| Ulcerative colitis | rs11230563 | G | 1.08 |  |
| Ulcerative colitis | rs11641184 | A | 1.08 |  |
| Ulcerative colitis | rs1517352 | C | 1.08 |  |
| Ulcerative colitis | rs2189234 | A | 1.08 |  |
| Ulcerative colitis | rs254560 | A | 1.08 |  |
| Ulcerative colitis | rs4976646 | G | 1.08 |  |
| Ulcerative colitis | rs7404095 | G | 1.08 |  |
| Ulcerative colitis | rs907611 | A | 1.08 |  |
| Ulcerative colitis | rs913678 | A | 1.08 |  |
| Ulcerative colitis | rs11083840 | C | 1.07 |  |
| Ulcerative colitis | rs12718244 | A | 1.07 |  |
| Ulcerative colitis | rs17771967 | G | 1.07 | HWE p < 1e-6 |
| Ulcerative colitis | rs2382817 | A | 1.07 |  |
