# Supplementary material for: Human longevity is influenced by many genetic variants: evidence from 75,000 UK Biobank participants
Source: Aging (Albany NY). 2016 Mar 23;8(3):547–60. doi: 10.18632/aging.100930 (PMC4833145; doi:10.18632/aging.100930)
Supplement: Supplementary file 4 [file aging-08-547-s004.docx]

**Supplementary Table 2**

The 1,000 most significant variants from each of the 4 GWAS performed

| **Phenotype** | **SNP** | **POS_b37** | **A1FREQ** | **BETA** | **SE** | **P_INF** | **quality** | **hwe_p** |
| --- | --- | --- | --- | --- | --- | --- | --- | --- |
| Combined parent's age at death (z-score) | rs769449 | 19:45410002_G_A | 0.873093 | 0.058581 | 0.011325 | 2.30E-07 | 1 | 0.59227 |
| Combined parent's age at death (z-score) | rs138436261 | 4:25332404_G_T | 0.982163 | 0.156528 | 0.030389 | 2.60E-07 | 0.88175 | 0.531925 |
| Combined parent's age at death (z-score) | rs12345013 | 9:79761711_A_G | 0.92471 | -0.07345 | 0.014284 | 2.70E-07 | 0.98776 | 0.269606 |
| Combined parent's age at death (z-score) | rs140324455 | 9:79896655_GTA_G | 0.927033 | -0.07378 | 0.014556 | 4.00E-07 | 0.98094 | 0.282501 |
| Combined parent's age at death (z-score) | rs569253495 | 9:79896659_A_G | 0.927033 | -0.07378 | 0.014556 | 4.00E-07 | 0.98094 | 0.282501 |
| Combined parent's age at death (z-score) | rs62448936 | 7:24507030_T_C | 0.554349 | 0.038805 | 0.007657 | 4.00E-07 | 0.98646 | 0.056144 |
| Combined parent's age at death (z-score) | rs12349795 | 9:79764473_T_C | 0.923814 | -0.07139 | 0.014139 | 4.40E-07 | 0.99764 | 0.302461 |
| Combined parent's age at death (z-score) | rs17184291 | 16:81161295_A_G | 0.89663 | -0.06192 | 0.012311 | 4.90E-07 | 1 | 0.138131 |
| Combined parent's age at death (z-score) | rs3135174 | 4:3256805_A_G | 0.566134 | -0.03822 | 0.007673 | 6.30E-07 | 0.97963 | 0.109726 |
| Combined parent's age at death (z-score) | rs55841186 | 3:6305909_T_C | 0.973639 | -0.12093 | 0.024377 | 7.00E-07 | 0.92755 | 0.772271 |
| Combined parent's age at death (z-score) | rs7033943 | 9:79720735_T_A | 0.931181 | -0.07427 | 0.015014 | 7.50E-07 | 0.97435 | 0.515905 |
| Combined parent's age at death (z-score) | rs2079642 | 4:3259065_T_C | 0.566513 | -0.03769 | 0.007645 | 8.20E-07 | 0.98894 | 0.045551 |
| Combined parent's age at death (z-score) | rs10410232 | 19:33613708_C_T | 0.955638 | 0.091064 | 0.018496 | 8.50E-07 | 0.9698 | 0.180098 |
| Combined parent's age at death (z-score) | rs11145286 | 9:79732411_C_T | 0.924501 | -0.07018 | 0.014264 | 8.70E-07 | 0.98733 | 0.27024 |
| Combined parent's age at death (z-score) | rs108850 | 4:3258236_C_G | 0.565779 | -0.03773 | 0.007674 | 8.80E-07 | 0.97979 | 0.077786 |
| Combined parent's age at death (z-score) | rs74617384 | 6:160997118_A_T | 0.918161 | 0.06755 | 0.013786 | 9.60E-07 | 1 | 0.133331 |
| Combined parent's age at death (z-score) | rs67163261 | 18:50976490_GATATATAT_G | 0.38315 | 0.039406 | 0.008052 | 9.90E-07 | 0.92515 | 0.349977 |
| Combined parent's age at death (z-score) | rs12421301 | 11:41219414_A_C | 0.847807 | -0.05138 | 0.01053 | 1.10E-06 | 0.99024 | 0.528896 |
| Combined parent's age at death (z-score) | rs79023354 | 9:79714058_A_G | 0.934796 | -0.07458 | 0.015309 | 1.10E-06 | 0.98727 | 0.906087 |
| Combined parent's age at death (z-score) | rs362280 | 4:3260057_A_G | 0.56083 | -0.03721 | 0.007635 | 1.10E-06 | 0.98847 | 0.04891 |
| Combined parent's age at death (z-score) | rs59031438 | 19:33621705_T_TA | 0.898879 | 0.060964 | 0.012493 | 1.10E-06 | 0.99034 | 0.044463 |
| Combined parent's age at death (z-score) | rs79741373 | 19:33619605_TG_T | 0.898853 | 0.060587 | 0.012479 | 1.20E-06 | 0.99247 | 0.046153 |
| Combined parent's age at death (z-score) | rs10455872 | 6:161010118_A_G | 0.91799 | 0.06679 | 0.013775 | 1.20E-06 | 1 | 0.143804 |
| Combined parent's age at death (z-score) | rs2171333 | 11:41216468_C_T | 0.849172 | -0.05096 | 0.010517 | 1.30E-06 | 1 | 0.585208 |
| Combined parent's age at death (z-score) | rs61854888 | 10:90928961_T_A | 0.985744 | -0.1596 | 0.033089 | 1.40E-06 | 0.91696 | 1 |
| Combined parent's age at death (z-score) | rs57389385 | 9:79720177_C_T | 0.934087 | -0.07407 | 0.015357 | 1.40E-06 | 0.97215 | 0.925312 |
| Combined parent's age at death (z-score) | rs55730499 | 6:161005610_C_T | 0.916605 | 0.066126 | 0.0137 | 1.40E-06 | 0.99515 | 0.184544 |
| Combined parent's age at death (z-score) | rs118039278 | 6:160985526_G_A | 0.918049 | 0.066638 | 0.013857 | 1.50E-06 | 0.98866 | 0.172199 |
| Combined parent's age at death (z-score) | rs6510356 | 19:33617583_C_T | 0.899192 | 0.059913 | 0.012451 | 1.50E-06 | 1 | 0.070372 |
| Combined parent's age at death (z-score) | rs11088872 | 21:23033104_G_A | 0.655463 | -0.03827 | 0.00798 | 1.60E-06 | 0.97626 | 0.183168 |
| Combined parent's age at death (z-score) | rs113690530 | 19:33087867_A_G | 0.978477 | 0.136498 | 0.028598 | 1.80E-06 | 0.81249 | 0.529895 |
| Combined parent's age at death (z-score) | rs79959541 | 2:127714851_T_C | 0.983589 | 0.144223 | 0.030215 | 1.80E-06 | 0.95846 | 0.927389 |
| Combined parent's age at death (z-score) | rs67947502 | 22:30487259_T_C | 0.98475 | -0.15824 | 0.033114 | 1.80E-06 | 0.86486 | 0.182974 |
| Combined parent's age at death (z-score) | rs112056118 | 19:33535886_T_C | 0.978121 | 0.122467 | 0.025812 | 2.10E-06 | 0.98677 | 0.639249 |
| Combined parent's age at death (z-score) | rs967006 | 2:231044212_A_G | 0.209687 | 0.044805 | 0.009468 | 2.20E-06 | 0.95053 | 0.638675 |
| Combined parent's age at death (z-score) | rs56205488 | 22:30542265_A_G | 0.988983 | -0.18218 | 0.038548 | 2.30E-06 | 0.87814 | 0.123319 |
| Combined parent's age at death (z-score) | rs111786600 | 19:33612192_TC_T | 0.900081 | 0.059071 | 0.012526 | 2.40E-06 | 0.99505 | 0.062015 |
| Combined parent's age at death (z-score) | rs114374360 | 2:127736061_G_A | 0.983962 | 0.140946 | 0.029945 | 2.50E-06 | 1 | 0.709284 |
| Combined parent's age at death (z-score) | rs75567045 | 9:79742573_AGCT_A | 0.930967 | -0.07031 | 0.014964 | 2.60E-06 | 0.97697 | 0.220658 |
| Combined parent's age at death (z-score) | rs200876032 | 19:33611221_TA_T | 0.899705 | 0.058617 | 0.012502 | 2.70E-06 | 0.99551 | 0.065148 |
| Combined parent's age at death (z-score) | rs143772911 | 1:226095237_G_C | 0.987502 | -0.1653 | 0.035269 | 2.80E-06 | 0.92351 | 0.484362 |
| Combined parent's age at death (z-score) | rs80340606 | 1:226095253_G_C | 0.987505 | -0.16533 | 0.035267 | 2.80E-06 | 0.92376 | 0.484362 |
| Combined parent's age at death (z-score) | rs79194774 | 3:106024685_G_A | 0.950355 | 0.081471 | 0.017484 | 3.20E-06 | 0.97255 | 0.760712 |
| Combined parent's age at death (z-score) | rs182994222 | 9:79697286_A_T | 0.934539 | -0.07128 | 0.015296 | 3.20E-06 | 0.98694 | 0.759277 |
| Combined parent's age at death (z-score) | rs112277911 | 19:33496732_G_C | 0.980716 | 0.132611 | 0.028525 | 3.30E-06 | 0.91552 | 0.940154 |
| Combined parent's age at death (z-score) | rs8113398 | 19:33610060_A_G | 0.896759 | 0.057813 | 0.012433 | 3.30E-06 | 0.98291 | 0.209103 |
| Combined parent's age at death (z-score) | rs201359663 | 9:79697287_G_GGGT | 0.934654 | -0.07096 | 0.015308 | 3.60E-06 | 0.98673 | 0.776998 |
| Combined parent's age at death (z-score) | rs117328225 | 9:79697289_C_T | 0.932576 | -0.06933 | 0.014989 | 3.70E-06 | 1 | 0.552001 |
| Combined parent's age at death (z-score) | rs111859823 | 13:110660190_G_A | 0.974058 | -0.10961 | 0.023695 | 3.70E-06 | 1 | 0.863052 |
| Combined parent's age at death (z-score) | rs62571396 | 9:79711780_A_G | 0.932295 | -0.06922 | 0.015015 | 4.00E-06 | 0.99051 | 1 |
| Combined parent's age at death (z-score) | rs10653116 | 19:33523628_T_TCACCC | 0.944409 | 0.07815 | 0.016957 | 4.10E-06 | 0.93482 | 0.473631 |
| Combined parent's age at death (z-score) | rs200268637 | 19:33523633_AGGATT_A | 0.944409 | 0.07815 | 0.016957 | 4.10E-06 | 0.93482 | 0.473631 |
| Combined parent's age at death (z-score) | rs56170974 | 1:226085362_G_A | 0.985218 | -0.14585 | 0.031682 | 4.20E-06 | 0.96734 | 0.268974 |
| Combined parent's age at death (z-score) | rs202203249 | 21:23070542_T_TC | 0.363264 | 0.037088 | 0.00807 | 4.30E-06 | 0.92793 | 0.726608 |
| Combined parent's age at death (z-score) | rs554233168 | 21:23070545_T_TC | 0.363264 | 0.037088 | 0.00807 | 4.30E-06 | 0.92793 | 0.726608 |
| Combined parent's age at death (z-score) | rs187350755 | 19:33611094_A_G | 0.899171 | 0.057115 | 0.012489 | 4.80E-06 | 0.99297 | 0.068533 |
| Combined parent's age at death (z-score) | rs76611583 | 1:226088652_T_C | 0.985344 | -0.1459 | 0.031889 | 4.80E-06 | 0.96309 | 0.224548 |
| Combined parent's age at death (z-score) | rs146267024 | 22:50722939_A_AC | 0.918637 | 0.064851 | 0.014183 | 4.80E-06 | 0.93698 | 0.846502 |
| Combined parent's age at death (z-score) | rs79150539 | 13:110654691_C_T | 0.969948 | -0.10132 | 0.022159 | 4.80E-06 | 0.99496 | 0.882633 |
| Combined parent's age at death (z-score) | rs34763349 | 13:110656106_T_A | 0.969734 | -0.1008 | 0.022064 | 4.90E-06 | 0.99646 | 0.883287 |
| Combined parent's age at death (z-score) | rs150416243 | 2:212216854_A_C | 0.982851 | -0.13659 | 0.029901 | 4.90E-06 | 0.93087 | 0.220425 |
| Combined parent's age at death (z-score) | rs12592111 | 15:78767346_G_A | 0.385095 | 0.03526 | 0.007716 | 4.90E-06 | 0.99966 | 0.357084 |
| Combined parent's age at death (z-score) | rs71331230 | 22:30609211_C_T | 0.985442 | -0.14595 | 0.03198 | 5.00E-06 | 0.96683 | 0.487776 |
| Combined parent's age at death (z-score) | rs80337237 | 13:110656625_C_T | 0.969716 | -0.10057 | 0.022053 | 5.10E-06 | 0.99699 | 0.883259 |
| Combined parent's age at death (z-score) | rs62571420 | 9:79717402_G_A | 0.933856 | -0.06956 | 0.015252 | 5.10E-06 | 0.98206 | 0.888863 |
| Combined parent's age at death (z-score) | rs6510354 | 19:33614855_G_A | 0.900259 | 0.057226 | 0.012543 | 5.10E-06 | 0.99352 | 0.051523 |
| Combined parent's age at death (z-score) | rs13190655 | 5:152438213_C_T | 0.957252 | -0.08521 | 0.018677 | 5.10E-06 | 1 | 0.409035 |
| Combined parent's age at death (z-score) | rs147142509 | 9:79696790_A_AT | 0.932862 | -0.06831 | 0.015006 | 5.30E-06 | 0.99917 | 0.872222 |
| Combined parent's age at death (z-score) | rs113082832 | 4:25443604_C_G | 0.944373 | 0.074566 | 0.01639 | 5.40E-06 | 1 | 0.486665 |
| Combined parent's age at death (z-score) | rs60771529 | 2:1994509_T_C | 0.895278 | 0.055638 | 0.012232 | 5.40E-06 | 1 | 0.030304 |
| Combined parent's age at death (z-score) | rs4362358 | 15:78796104_C_T | 0.381423 | 0.035363 | 0.007781 | 5.50E-06 | 0.98596 | 0.355361 |
| Combined parent's age at death (z-score) | rs77350958 | 6:165927537_T_C | 0.975495 | -0.11094 | 0.024412 | 5.50E-06 | 1 | 0.041352 |
| Combined parent's age at death (z-score) | rs76721692 | 13:110658467_A_G | 0.96972 | -0.10018 | 0.022055 | 5.60E-06 | 0.9972 | 0.922021 |
| Combined parent's age at death (z-score) | rs75029846 | 13:110658906_A_G | 0.96972 | -0.10018 | 0.022055 | 5.60E-06 | 0.9972 | 0.922021 |
| Combined parent's age at death (z-score) | rs75893981 | 13:110659650_G_C | 0.96972 | -0.10018 | 0.022055 | 5.60E-06 | 0.9972 | 0.922021 |
| Combined parent's age at death (z-score) | rs659722 | 11:106586473_C_G | 0.97866 | 0.118772 | 0.026165 | 5.60E-06 | 0.98605 | 0.459484 |
| Combined parent's age at death (z-score) | rs596819 | 11:106561948_A_G | 0.979467 | 0.120809 | 0.026629 | 5.70E-06 | 0.99214 | 0.781016 |
| Combined parent's age at death (z-score) | rs77262559 | 13:110616403_A_G | 0.969657 | -0.10061 | 0.022181 | 5.70E-06 | 0.9825 | 0.805833 |
| Combined parent's age at death (z-score) | rs141783898 | 4:25240667_G_A | 0.969153 | 0.100556 | 0.022161 | 5.70E-06 | 0.96389 | 0.57248 |
| Combined parent's age at death (z-score) | rs16875255 | 4:24185401_C_T | 0.908232 | 0.059154 | 0.013062 | 5.90E-06 | 0.98806 | 0.488214 |
| Combined parent's age at death (z-score) | rs374343949 | 5:152560334_AGGTGT_A | 0.793755 | -0.04266 | 0.009416 | 5.90E-06 | 0.97162 | 0.33091 |
| Combined parent's age at death (z-score) | rs5809679 | 14:75282873_AT_A | 0.314731 | 0.037788 | 0.008354 | 6.10E-06 | 0.93924 | 0.170876 |
| Combined parent's age at death (z-score) | rs12899351 | 15:78792398_T_C | 0.381685 | 0.035191 | 0.007778 | 6.10E-06 | 0.98643 | 0.346019 |
| Combined parent's age at death (z-score) | rs12594711 | 15:78793921_C_T | 0.38172 | 0.035175 | 0.007777 | 6.10E-06 | 0.98651 | 0.349181 |
| Combined parent's age at death (z-score) | rs12962464 | 18:50967439_G_A | 0.445156 | 0.034238 | 0.007569 | 6.10E-06 | 1 | 0.205017 |
| Combined parent's age at death (z-score) | rs62571393 | 9:79705913_T_G | 0.932625 | -0.06788 | 0.015006 | 6.10E-06 | 0.99575 | 0.945218 |
| Combined parent's age at death (z-score) | rs78682846 | 9:79699494_C_T | 0.9328 | -0.0679 | 0.015035 | 6.30E-06 | 0.99495 | 0.9268 |
| Combined parent's age at death (z-score) | rs181525252 | 7:137803648_G_A | 0.982898 | -0.14264 | 0.031573 | 6.30E-06 | 0.85092 | 0.496021 |
| Combined parent's age at death (z-score) | rs74356282 | 13:110617554_A_G | 0.974383 | -0.1083 | 0.023974 | 6.30E-06 | 0.9879 | 0.815502 |
| Combined parent's age at death (z-score) | rs77870171 | 13:110656020_C_T | 0.96962 | -0.09948 | 0.022038 | 6.40E-06 | 0.99587 | 0.961077 |
| Combined parent's age at death (z-score) | rs62571388 | 9:79700275_C_T | 0.932694 | -0.06767 | 0.015 | 6.40E-06 | 0.99785 | 0.872457 |
| Combined parent's age at death (z-score) | rs10118161 | 9:79763338_C_T | 0.904727 | -0.05752 | 0.012751 | 6.40E-06 | 1 | 0.070291 |
| Combined parent's age at death (z-score) | rs11661326 | 18:50973180_C_T | 0.440088 | 0.034409 | 0.007632 | 6.50E-06 | 0.98259 | 0.851399 |
| Combined parent's age at death (z-score) | rs77823495 | 13:110623067_G_A | 0.974119 | -0.10771 | 0.023883 | 6.50E-06 | 0.98663 | 0.907986 |
| Combined parent's age at death (z-score) | rs74686791 | 13:110623498_C_A | 0.974119 | -0.10771 | 0.023883 | 6.50E-06 | 0.98663 | 0.907986 |
| Combined parent's age at death (z-score) | rs114461222 | 5:112212914_G_C | 0.988198 | -0.16136 | 0.035806 | 6.60E-06 | 0.94448 | 0.102674 |
| Combined parent's age at death (z-score) | rs2315065 | 6:161108144_C_A | 0.893508 | 0.056891 | 0.012637 | 6.70E-06 | 0.93715 | 0.915482 |
| Combined parent's age at death (z-score) | rs12953691 | 18:50969096_A_T | 0.442721 | 0.034075 | 0.007572 | 6.80E-06 | 0.9983 | 0.505033 |
| Combined parent's age at death (z-score) | rs7982954 | 13:110654010_C_T | 0.969412 | -0.0988 | 0.021962 | 6.80E-06 | 0.99445 | 0.772129 |
| Combined parent's age at death (z-score) | rs3135170 | 4:3262323_T_C | 0.583482 | -0.03458 | 0.007685 | 6.80E-06 | 0.99091 | 0.06108 |
| Combined parent's age at death (z-score) | rs35353631 | 4:24183617_T_A | 0.908654 | 0.058774 | 0.013083 | 7.00E-06 | 0.98889 | 0.454402 |
| Combined parent's age at death (z-score) | rs72698726 | 14:101188618_C_T | 0.873869 | -0.05109 | 0.011371 | 7.00E-06 | 0.99091 | 0.164407 |
| Combined parent's age at death (z-score) | rs10421328 | 19:19776814_C_T | 0.786654 | 0.041309 | 0.009195 | 7.00E-06 | 0.99222 | 0.31684 |
| Combined parent's age at death (z-score) | rs74469597 | 5:16484852_A_T | 0.913864 | 0.0613 | 0.013638 | 7.00E-06 | 0.95526 | 0.569705 |
| Combined parent's age at death (z-score) | rs12962394 | 18:50967591_C_A | 0.442905 | 0.034016 | 0.007571 | 7.00E-06 | 0.99879 | 0.493935 |
| Combined parent's age at death (z-score) | 15:78772261_T_TAA | 15:78772261_T_TA | 0.831255 | -0.0461 | 0.010271 | 7.20E-06 | 0.86998 | 0.655436 |
| Combined parent's age at death (z-score) | 15:78772261_T_TAA | 15:78772261_T_TAA | 0.831255 | -0.0461 | 0.010271 | 7.20E-06 | 0.95406 | 0.725922 |
| Combined parent's age at death (z-score) | rs75268262 | 13:110621468_A_T | 0.968929 | -0.09817 | 0.021872 | 7.20E-06 | 0.98911 | 0.923485 |
| Combined parent's age at death (z-score) | rs75914460 | 13:110621807_G_A | 0.968906 | -0.09818 | 0.021873 | 7.20E-06 | 0.9884 | 0.885483 |
| Combined parent's age at death (z-score) | rs78570879 | 13:110624029_C_G | 0.968934 | -0.09809 | 0.021876 | 7.30E-06 | 0.98885 | 0.92343 |
| Combined parent's age at death (z-score) | rs55966621 | 15:81137076_C_A | 0.931779 | 0.066907 | 0.014919 | 7.30E-06 | 1 | 0.873188 |
| Combined parent's age at death (z-score) | rs12960505 | 18:50972182_G_T | 0.438369 | 0.033993 | 0.007583 | 7.40E-06 | 0.99706 | 0.747223 |
| Combined parent's age at death (z-score) | rs16875258 | 4:24185518_T_C | 0.908398 | 0.058565 | 0.013069 | 7.40E-06 | 0.98813 | 0.476782 |
| Combined parent's age at death (z-score) | rs11929834 | 4:114072904_G_A | 0.649165 | 0.035426 | 0.007911 | 7.50E-06 | 0.99369 | 0.24363 |
| Combined parent's age at death (z-score) | rs4446368 | 4:157523922_C_T | 0.275599 | -0.03787 | 0.008453 | 7.50E-06 | 0.98907 | 0.90805 |
| Combined parent's age at death (z-score) | rs4302505 | 4:157524087_A_G | 0.275448 | -0.03785 | 0.008452 | 7.50E-06 | 0.98973 | 0.834111 |
| Combined parent's age at death (z-score) | rs667287 | 11:106552130_T_C | 0.979462 | 0.11921 | 0.026618 | 7.50E-06 | 0.99302 | 0.780657 |
| Combined parent's age at death (z-score) | rs144081173 | 18:47245225_G_T | 0.965374 | 0.09376 | 0.020951 | 7.60E-06 | 0.95781 | 0.49908 |
| Combined parent's age at death (z-score) | rs11664788 | 18:50973186_T_G | 0.439683 | 0.034142 | 0.007637 | 7.80E-06 | 0.98178 | 0.814813 |
| Combined parent's age at death (z-score) | rs2033794 | 12:29884553_T_C | 0.696817 | 0.037198 | 0.008325 | 7.90E-06 | 0.96848 | 0.503709 |
| Combined parent's age at death (z-score) | rs1870861 | 5:152564649_T_G | 0.787433 | -0.04103 | 0.009186 | 7.90E-06 | 0.99834 | 0.345541 |
| Combined parent's age at death (z-score) | rs57601701 | 18:50970438_G_T | 0.443592 | 0.033844 | 0.007582 | 8.00E-06 | 0.99596 | 0.472183 |
| Combined parent's age at death (z-score) | rs11535739 | 11:100527039_T_C | 0.959918 | -0.09322 | 0.020882 | 8.00E-06 | 0.83851 | 0.286187 |
| Combined parent's age at death (z-score) | rs11537179 | 11:100527040_G_C | 0.959918 | -0.09322 | 0.020882 | 8.00E-06 | 0.83851 | 0.286187 |
| Combined parent's age at death (z-score) | rs71610809 | 4:24179748_C_T | 0.904459 | 0.057625 | 0.012903 | 8.00E-06 | 0.97708 | 0.375465 |
| Combined parent's age at death (z-score) | rs7665195 | 4:24183971_T_C | 0.0919747 | -0.05829 | 0.013054 | 8.00E-06 | 0.98759 | 0.533556 |
| Combined parent's age at death (z-score) | rs71610808 | 4:24179732_T_C | 0.908496 | 0.058335 | 0.013073 | 8.10E-06 | 0.98852 | 0.465608 |
| Combined parent's age at death (z-score) | rs56291226 | 2:212061885_C_T | 0.978085 | -0.11462 | 0.025684 | 8.10E-06 | 1 | 0.946318 |
| Combined parent's age at death (z-score) | rs10279833 | 7:28901965_T_C | 0.871001 | 0.049891 | 0.011185 | 8.20E-06 | 1 | 0.486206 |
| Combined parent's age at death (z-score) | rs965604 | 15:78789223_G_A | 0.382087 | 0.034437 | 0.007722 | 8.20E-06 | 0.99992 | 0.312838 |
| Combined parent's age at death (z-score) | rs8076336 | 17:18212614_G_T | 0.567523 | 0.033873 | 0.007593 | 8.20E-06 | 0.99878 | 0.426943 |
| Combined parent's age at death (z-score) | rs71610810 | 4:24180167_C_T | 0.90859 | 0.0583 | 0.013078 | 8.30E-06 | 0.98884 | 0.465243 |
| Combined parent's age at death (z-score) | rs4252185 | 6:161123451_T_C | 0.895165 | 0.057045 | 0.012801 | 8.30E-06 | 0.9257 | 0.782042 |
| Combined parent's age at death (z-score) | rs35623800 | 3:30118478_AT_A | 0.691576 | -0.03841 | 0.008624 | 8.40E-06 | 0.89278 | 0.519182 |
| Combined parent's age at death (z-score) | rs6846259 | 4:24177422_C_T | 0.908667 | 0.05832 | 0.013093 | 8.40E-06 | 0.98734 | 0.433551 |
| Combined parent's age at death (z-score) | rs112718018 | 4:157521850_A_G | 0.2755 | -0.03764 | 0.008447 | 8.40E-06 | 0.99033 | 0.822911 |
| Combined parent's age at death (z-score) | rs9588066 | 13:110661287_G_A | 0.969366 | -0.09812 | 0.022051 | 8.60E-06 | 0.98511 | 0.664661 |
| Combined parent's age at death (z-score) | rs35915724 | 4:24192006_C_T | 0.908684 | 0.058116 | 0.013069 | 8.70E-06 | 0.99116 | 0.508358 |
| Combined parent's age at death (z-score) | rs116069046 | 5:152572037_C_A | 0.78736 | -0.0409 | 0.0092 | 8.70E-06 | 0.99361 | 0.27609 |
| Combined parent's age at death (z-score) | rs13157574 | 5:152547025_G_A | 0.787169 | -0.04085 | 0.00919 | 8.80E-06 | 0.99657 | 0.386919 |
| Combined parent's age at death (z-score) | rs13180 | 15:78789488_C_T | 0.382129 | 0.034326 | 0.007722 | 8.80E-06 | 1 | 0.315813 |
| Combined parent's age at death (z-score) | rs8042260 | 15:78774374_A_G | 0.381721 | 0.034315 | 0.007725 | 8.90E-06 | 0.99956 | 0.265424 |
| Combined parent's age at death (z-score) | rs1964678 | 15:78754000_A_G | 0.379966 | 0.03434 | 0.00773 | 8.90E-06 | 1 | 0.317644 |
| Combined parent's age at death (z-score) | rs12522365 | 5:152561963_G_A | 0.78659 | -0.04078 | 0.009179 | 8.90E-06 | 0.99634 | 0.321329 |
| Combined parent's age at death (z-score) | rs55831814 | 19:19777644_C_T | 0.787915 | 0.04087 | 0.009202 | 8.90E-06 | 0.99574 | 0.371578 |
| Combined parent's age at death (z-score) | rs77158908 | 4:24180667_TA_T | 0.908405 | 0.058071 | 0.013072 | 8.90E-06 | 0.988 | 0.4246 |
| Combined parent's age at death (z-score) | rs67022228 | 14:101191007_A_T | 0.727231 | -0.03846 | 0.008663 | 9.00E-06 | 0.95435 | 0.656115 |
| Combined parent's age at death (z-score) | rs4691372 | 4:157593703_C_T | 0.222236 | -0.04016 | 0.009048 | 9.00E-06 | 1 | 0.593827 |
| Combined parent's age at death (z-score) | rs11874018 | 18:50976688_T_C | 0.437033 | 0.033646 | 0.007586 | 9.20E-06 | 0.99653 | 0.769361 |
| Combined parent's age at death (z-score) | rs71588079 | 5:152571659_G_A | 0.787049 | -0.04075 | 0.009187 | 9.20E-06 | 0.99513 | 0.26911 |
| Combined parent's age at death (z-score) | rs564885564 | 5:25438388_G_GT | 0.987526 | -0.16262 | 0.036693 | 9.30E-06 | 0.86171 | 0.29651 |
| Combined parent's age at death (z-score) | rs7994376 | 13:110660745_T_A | 0.96946 | -0.09782 | 0.022061 | 9.30E-06 | 0.98716 | 0.698924 |
| Combined parent's age at death (z-score) | rs1502225 | 18:50978637_G_A | 0.438037 | 0.033602 | 0.007581 | 9.30E-06 | 0.99729 | 0.911337 |
| Combined parent's age at death (z-score) | rs12957920 | 18:50975543_A_T | 0.437387 | 0.033591 | 0.007581 | 9.40E-06 | 0.99741 | 0.810032 |
| Combined parent's age at death (z-score) | rs6510330 | 19:33520073_C_T | 0.946432 | 0.076569 | 0.01728 | 9.40E-06 | 0.93205 | 0.33282 |
| Combined parent's age at death (z-score) | rs553626126 | 1:234914694_C_T | 0.986408 | 0.176503 | 0.039829 | 9.40E-06 | 0.64807 | 1 |
| Combined parent's age at death (z-score) | rs556991919 | 1:104827489_C_G | 0.9848 | -0.1767 | 0.039894 | 9.50E-06 | 0.60391 | 0.298992 |
| Combined parent's age at death (z-score) | rs12428468 | 13:110636631_A_G | 0.969707 | -0.09751 | 0.022023 | 9.50E-06 | 0.99977 | 1 |
| Combined parent's age at death (z-score) | rs11661305 | 18:50973169_A_G | 0.440044 | 0.033678 | 0.007604 | 9.50E-06 | 0.9898 | 0.869775 |
| Combined parent's age at death (z-score) | rs12604130 | 18:50974215_T_C | 0.43737 | 0.033571 | 0.007581 | 9.50E-06 | 0.9974 | 0.814577 |
| Combined parent's age at death (z-score) | rs869225 | 18:50977732_G_T | 0.437386 | 0.033583 | 0.007582 | 9.50E-06 | 0.99729 | 0.80549 |
| Combined parent's age at death (z-score) | rs36146269 | 15:78779510_T_A | 0.382021 | 0.034193 | 0.007723 | 9.50E-06 | 0.99981 | 0.321692 |
| Combined parent's age at death (z-score) | rs8042238 | 15:78774271_C_T | 0.38156 | 0.034185 | 0.007723 | 9.60E-06 | 1 | 0.275975 |
| Combined parent's age at death (z-score) | rs113549776 | 13:110622108_G_A | 0.968844 | -0.09668 | 0.021843 | 9.60E-06 | 0.9883 | 0.77382 |
| Combined parent's age at death (z-score) | rs74892655 | 13:110650730_G_C | 0.969846 | -0.09786 | 0.022111 | 9.60E-06 | 0.99623 | 0.9608 |
| Combined parent's age at death (z-score) | rs13168691 | 5:152566077_G_C | 0.787208 | -0.04063 | 0.009179 | 9.60E-06 | 0.99865 | 0.359062 |
| Combined parent's age at death (z-score) | rs34586652 | 18:50967184_TA_T | 0.445841 | 0.033533 | 0.007581 | 9.70E-06 | 0.99482 | 0.341232 |
| Combined parent's age at death (z-score) | rs12429445 | 13:110650457_A_C | 0.969722 | -0.09748 | 0.022043 | 9.80E-06 | 0.99871 | 1 |
| Combined parent's age at death (z-score) | rs10505728 | 12:4320185_T_A | 0.965406 | -0.09141 | 0.020675 | 9.80E-06 | 1 | 0.117948 |
| Combined parent's age at death (z-score) | rs12428472 | 13:110636706_A_G | 0.969919 | -0.09781 | 0.022131 | 9.90E-06 | 0.99689 | 1 |
| Combined parent's age at death (z-score) | rs1559112 | 5:152580137_G_T | 0.786716 | -0.04058 | 0.009184 | 9.90E-06 | 0.99494 | 0.258666 |
| Combined parent's age at death (z-score) | rs4887059 | 15:78782095_C_T | 0.382191 | 0.034125 | 0.007722 | 9.90E-06 | 0.9997 | 0.318787 |
| Combined parent's age at death (z-score) | rs540443223 | 15:61194493_A_T | 0.976805 | -0.11514 | 0.02605 | 9.90E-06 | 0.92384 | 0.846327 |
| Combined parent's age at death (z-score) | rs7219628 | 17:18189249_T_C | 0.567361 | 0.033575 | 0.007599 | 9.90E-06 | 0.99787 | 0.437321 |
| Combined parent's age at death (z-score) | rs8066387 | 17:18187621_T_C | 0.567816 | 0.033602 | 0.007606 | 1.00E-05 | 0.99633 | 0.440687 |
| Combined parent's age at death (z-score) | rs8084696 | 18:1819543_G_T | 0.852255 | 0.04729 | 0.010727 | 1.00E-05 | 0.97341 | 0.026375 |
| Combined parent's age at death (z-score) | rs1504549 | 15:78766629_C_T | 0.382179 | 0.034075 | 0.007723 | 1.00E-05 | 0.99948 | 0.333864 |
| Combined parent's age at death (z-score) | rs12903295 | 15:78778972_A_G | 0.381363 | 0.034125 | 0.007725 | 1.00E-05 | 0.9998 | 0.289581 |
| Combined parent's age at death (z-score) | rs12904234 | 15:78779384_C_T | 0.381714 | 0.034069 | 0.007725 | 1.00E-05 | 0.99975 | 0.34291 |
| Combined parent's age at death (z-score) | rs1062980 | 15:78792527_T_C | 0.618778 | -0.03436 | 0.00778 | 1.00E-05 | 0.98643 | 0.381338 |
| Combined parent's age at death (z-score) | rs71852607 | 15:78799060_ACT_A | 0.381789 | 0.034327 | 0.007785 | 1.00E-05 | 0.98486 | 0.358765 |
| Combined parent's age at death (z-score) | rs4299116 | 15:78766194_T_A | 0.382006 | 0.034032 | 0.007722 | 1.00E-05 | 0.99979 | 0.315748 |
| Combined parent's age at death (z-score) | rs11082998 | 18:50964031_G_A | 0.445565 | 0.03346 | 0.007581 | 1.00E-05 | 0.99511 | 0.341169 |
| Combined parent's age at death (z-score) | rs11082999 | 18:50964095_T_C | 0.44562 | 0.033469 | 0.00758 | 1.00E-05 | 0.99534 | 0.341185 |
| Combined parent's age at death (z-score) | rs10502977 | 18:50964372_G_C | 0.445621 | 0.03347 | 0.00758 | 1.00E-05 | 0.99534 | 0.341177 |
| Combined parent's age at death (z-score) | rs16956735 | 18:50978986_A_G | 0.436961 | 0.033487 | 0.007584 | 1.00E-05 | 0.99706 | 0.888078 |
| Combined parent's age at death (z-score) | rs74517256 | 13:110638471_A_G | 0.969722 | -0.09724 | 0.022031 | 1.00E-05 | 0.99955 | 1 |
| Combined parent's age at death (z-score) | rs75246465 | 13:110638710_C_G | 0.969722 | -0.09724 | 0.022031 | 1.00E-05 | 0.99955 | 1 |
| Combined parent's age at death (z-score) | rs139225128 | 13:110644108_T_C | 0.969743 | -0.09718 | 0.022048 | 1.00E-05 | 0.99881 | 1 |
| Combined parent's age at death (z-score) | rs79635430 | 13:110646192_G_A | 0.969743 | -0.09718 | 0.022048 | 1.00E-05 | 0.9988 | 1 |
| Combined parent's age at death (z-score) | rs78424303 | 13:110648577_G_A | 0.969743 | -0.09718 | 0.022048 | 1.00E-05 | 0.9988 | 1 |
| Combined parent's age at death (z-score) | rs149272296 | 4:25225428_T_C | 0.969328 | 0.098495 | 0.022293 | 1.00E-05 | 0.95798 | 0.602837 |
| Combined parent's age at death (z-score) | rs7659144 | 4:3098321_C_G | 0.649388 | -0.03496 | 0.007927 | 1.00E-05 | 0.99042 | 0.143061 |
| Combined parent's age at death (z-score) | rs13157119 | 5:152546935_C_T | 0.787006 | -0.04052 | 0.009191 | 1.00E-05 | 0.99585 | 0.368706 |
| Combined parent's age at death (z-score) | rs34611901 | 5:152548305_C_G | 0.787013 | -0.04047 | 0.009182 | 1.00E-05 | 0.99762 | 0.377859 |
| Combined parent's age at death (z-score) | rs13176510 | 5:152548490_A_T | 0.78702 | -0.04048 | 0.009182 | 1.00E-05 | 0.99759 | 0.373267 |
| Combined parent's age at death (z-score) | rs148684043 | 5:152562989_G_A | 0.786627 | -0.04064 | 0.009198 | 1.00E-05 | 0.99191 | 0.329807 |
| Combined parent's age at death (z-score) | rs34165048 | 5:152583317_T_A | 0.786558 | -0.0406 | 0.009193 | 1.00E-05 | 0.99266 | 0.266238 |
| Combined parent's age at death (z-score) | rs36114345 | 7:24509702_G_GT | 0.583443 | 0.033955 | 0.007697 | 1.00E-05 | 0.98492 | 0.369922 |
| Combined parent's age at death (z-score) | rs34605934 | 5:152512454_C_A | 0.78659 | -0.04037 | 0.009199 | 1.10E-05 | 0.99418 | 0.467509 |
| Combined parent's age at death (z-score) | rs13156645 | 5:152546657_C_T | 0.786952 | -0.04037 | 0.009182 | 1.10E-05 | 0.99739 | 0.373345 |
| Combined parent's age at death (z-score) | rs12518199 | 5:152550924_G_T | 0.787089 | -0.04038 | 0.009184 | 1.10E-05 | 0.99742 | 0.363982 |
| Combined parent's age at death (z-score) | rs137925282 | 5:152561451_AG_A | 0.787664 | -0.04048 | 0.009207 | 1.10E-05 | 0.99453 | 0.372229 |
| Combined parent's age at death (z-score) | rs114167462 | 3:106053542_C_T | 0.951175 | 0.076491 | 0.017366 | 1.10E-05 | 1 | 0.386865 |
| Combined parent's age at death (z-score) | rs112092968 | 19:33518065_C_T | 0.947052 | 0.076203 | 0.017332 | 1.10E-05 | 0.93687 | 0.387695 |
| Combined parent's age at death (z-score) | rs12593229 | 15:78765290_T_G | 0.381331 | 0.033975 | 0.007724 | 1.10E-05 | 0.99978 | 0.260034 |
| Combined parent's age at death (z-score) | rs12910910 | 15:78767850_C_T | 0.381979 | 0.033983 | 0.007721 | 1.10E-05 | 0.9999 | 0.306959 |
| Combined parent's age at death (z-score) | rs8043227 | 15:78768871_C_G | 0.381957 | 0.034023 | 0.007722 | 1.10E-05 | 0.99985 | 0.312781 |
| Combined parent's age at death (z-score) | rs12903285 | 15:78778953_A_G | 0.386293 | 0.034116 | 0.007747 | 1.10E-05 | 0.98912 | 0.297598 |
| Combined parent's age at death (z-score) | rs112236733 | 15:78783832_GTATGTATTTATT_G | 0.381243 | 0.034001 | 0.007728 | 1.10E-05 | 0.99884 | 0.278593 |
| Combined parent's age at death (z-score) | rs28880370 | 17:18182720_A_G | 0.567902 | 0.033447 | 0.007594 | 1.10E-05 | 0.99908 | 0.476343 |
| Combined parent's age at death (z-score) | rs4925159 | 17:18185510_G_A | 0.567751 | 0.033431 | 0.00759 | 1.10E-05 | 1 | 0.513582 |
| Combined parent's age at death (z-score) | rs7207666 | 17:18192614_T_C | 0.567892 | 0.033449 | 0.007593 | 1.10E-05 | 0.9996 | 0.4372 |
| Combined parent's age at death (z-score) | rs2168781 | 17:18240746_C_G | 0.56876 | 0.033455 | 0.007605 | 1.10E-05 | 0.99839 | 0.352103 |
| Combined parent's age at death (z-score) | rs75967541 | 13:110643583_C_T | 0.969718 | -0.09667 | 0.022024 | 1.10E-05 | 1 | 1 |
| Combined parent's age at death (z-score) | rs369557327 | 4:157524158_C_CT | 0.275596 | -0.03717 | 0.008452 | 1.10E-05 | 0.99032 | 0.806315 |
| Combined parent's age at death (z-score) | rs76381506 | 4:24176976_T_G | 0.909484 | 0.057555 | 0.013171 | 1.20E-05 | 0.98335 | 0.380925 |
| Combined parent's age at death (z-score) | rs189187135 | 2:14558241_A_T | 0.988061 | -0.15867 | 0.036187 | 1.20E-05 | 0.89532 | 0.165554 |
| Combined parent's age at death (z-score) | rs12429247 | 13:110644729_A_G | 0.969896 | -0.0968 | 0.022117 | 1.20E-05 | 0.99729 | 0.960742 |
| Combined parent's age at death (z-score) | rs62191945 | 20:11560425_A_T | 0.91062 | 0.057825 | 0.013232 | 1.20E-05 | 0.99618 | 0.141778 |
| Combined parent's age at death (z-score) | rs13154170 | 5:152523263_G_A | 0.786857 | -0.04022 | 0.009197 | 1.20E-05 | 0.99477 | 0.456657 |
| Combined parent's age at death (z-score) | rs34244108 | 5:152528318_A_G | 0.786781 | -0.0403 | 0.009195 | 1.20E-05 | 0.99494 | 0.44655 |
| Combined parent's age at death (z-score) | rs12516389 | 5:152532584_G_A | 0.786879 | -0.04018 | 0.009195 | 1.20E-05 | 0.99525 | 0.416141 |
| Combined parent's age at death (z-score) | rs12517903 | 5:152534905_G_A | 0.786691 | -0.04019 | 0.009197 | 1.20E-05 | 0.99435 | 0.411529 |
| Combined parent's age at death (z-score) | rs80197494 | 5:152539791_C_T | 0.786916 | -0.04023 | 0.009183 | 1.20E-05 | 0.99743 | 0.421357 |
| Combined parent's age at death (z-score) | rs13190158 | 5:152559879_T_G | 0.7875 | -0.04024 | 0.009198 | 1.20E-05 | 0.99564 | 0.358872 |
| Combined parent's age at death (z-score) | rs13156828 | 5:152576916_A_G | 0.785799 | -0.04016 | 0.009173 | 1.20E-05 | 0.99353 | 0.232434 |
| Combined parent's age at death (z-score) | rs78675401 | 7:33054114_CTT_C | 0.907948 | 0.057103 | 0.013069 | 1.20E-05 | 0.99801 | 0.130972 |
| Combined parent's age at death (z-score) | rs1975821 | 17:18244814_T_C | 0.5686 | 0.033321 | 0.007608 | 1.20E-05 | 0.99696 | 0.373844 |
| Combined parent's age at death (z-score) | rs11637656 | 15:78751961_C_T | 0.380666 | 0.033877 | 0.007739 | 1.20E-05 | 0.99667 | 0.28372 |
| Combined parent's age at death (z-score) | rs8033999 | 15:92169857_T_C | 0.466516 | 0.033085 | 0.007552 | 1.20E-05 | 1 | 0.316636 |
| Combined parent's age at death (z-score) | rs8093161 | 18:50976512_T_C | 0.41833 | 0.033668 | 0.007678 | 1.20E-05 | 0.98319 | 0.525836 |
| Combined parent's age at death (z-score) | rs2088949 | 18:50978121_G_C | 0.432259 | 0.033556 | 0.007656 | 1.20E-05 | 0.98142 | 0.92038 |
| Combined parent's age at death (z-score) | rs185465898 | 9:6463044_G_C | 0.982985 | 0.135157 | 0.030887 | 1.20E-05 | 0.88072 | 0.027265 |
| Combined parent's age at death (z-score) | rs9644831 | 9:33514389_G_C | 0.971827 | -0.10302 | 0.0235 | 1.20E-05 | 0.93058 | 0.493401 |
| Combined parent's age at death (z-score) | rs7221807 | 17:18221799_T_C | 0.567975 | 0.033129 | 0.007594 | 1.30E-05 | 0.99897 | 0.46186 |
| Combined parent's age at death (z-score) | rs1975822 | 17:18244770_C_T | 0.568881 | 0.033127 | 0.00761 | 1.30E-05 | 0.99683 | 0.412914 |
| Combined parent's age at death (z-score) | rs2826965 | 21:23086597_G_A | 0.642278 | -0.03438 | 0.007876 | 1.30E-05 | 0.98597 | 0.76768 |
| Combined parent's age at death (z-score) | rs1487080 | 11:41224281_T_C | 0.842316 | -0.0453 | 0.010373 | 1.30E-05 | 0.99142 | 0.658825 |
| Combined parent's age at death (z-score) | rs2169485 | 11:41079587_A_G | 0.82703 | 0.043528 | 0.009986 | 1.30E-05 | 0.99169 | 0.991915 |
| Combined parent's age at death (z-score) | rs11872815 | 18:50966547_G_A | 0.445271 | 0.03308 | 0.007581 | 1.30E-05 | 0.99498 | 0.368443 |
| Combined parent's age at death (z-score) | rs1502231 | 18:50993642_T_A | 0.44348 | 0.03318 | 0.007613 | 1.30E-05 | 0.9856 | 0.874593 |
| Combined parent's age at death (z-score) | rs4887057 | 15:78760918_G_A | 0.619048 | -0.03372 | 0.007728 | 1.30E-05 | 0.99983 | 0.275766 |
| Combined parent's age at death (z-score) | rs12916801 | 15:78769130_A_G | 0.382134 | 0.033703 | 0.007722 | 1.30E-05 | 0.99966 | 0.32774 |
| Combined parent's age at death (z-score) | rs2667279 | 4:24156458_T_A | 0.889469 | 0.052305 | 0.01201 | 1.30E-05 | 0.99266 | 0.289101 |
| Combined parent's age at death (z-score) | rs2798282 | 4:3029916_A_G | 0.297081 | 0.036303 | 0.008329 | 1.30E-05 | 0.97193 | 0.021757 |
| Combined parent's age at death (z-score) | rs35708928 | 5:152516410_C_T | 0.787034 | -0.04012 | 0.0092 | 1.30E-05 | 0.99495 | 0.456478 |
| Combined parent's age at death (z-score) | rs12518081 | 5:152522954_A_G | 0.78683 | -0.04002 | 0.009194 | 1.30E-05 | 0.99519 | 0.426256 |
| Combined parent's age at death (z-score) | rs71588075 | 5:152529330_C_T | 0.786742 | -0.04012 | 0.009194 | 1.30E-05 | 0.99514 | 0.451679 |
| Combined parent's age at death (z-score) | rs34316705 | 5:152534727_C_A | 0.786581 | -0.04018 | 0.009199 | 1.30E-05 | 0.99349 | 0.42157 |
| Combined parent's age at death (z-score) | rs71588077 | 5:152535916_C_G | 0.786322 | -0.04003 | 0.009192 | 1.30E-05 | 0.99486 | 0.489035 |
| Combined parent's age at death (z-score) | rs80094630 | 5:152564226_C_A | 0.786975 | -0.04012 | 0.009186 | 1.30E-05 | 0.99627 | 0.342066 |
| Combined parent's age at death (z-score) | rs71588081 | 5:152583794_C_G | 0.785486 | -0.03999 | 0.009182 | 1.30E-05 | 0.99169 | 0.271775 |
| Combined parent's age at death (z-score) | rs919778 | 19:19772722_G_A | 0.800248 | 0.041025 | 0.009435 | 1.40E-05 | 0.99048 | 0.389108 |
| Combined parent's age at death (z-score) | rs539530657 | 19:19798954_ATATT_A | 0.950606 | 0.080135 | 0.018439 | 1.40E-05 | 0.88706 | 0.467251 |
| Combined parent's age at death (z-score) | rs329745 | 19:53540541_A_G | 0.626809 | 0.036658 | 0.008435 | 1.40E-05 | 0.84516 | 0.858266 |
| Combined parent's age at death (z-score) | rs71588074 | 5:152513524_C_A | 0.787592 | -0.04 | 0.009221 | 1.40E-05 | 0.99268 | 0.466069 |
| Combined parent's age at death (z-score) | rs77305357 | 5:152514374_G_T | 0.786683 | -0.03995 | 0.009197 | 1.40E-05 | 0.99466 | 0.467337 |
| Combined parent's age at death (z-score) | rs7443245 | 5:152523960_C_A | 0.787442 | -0.03998 | 0.009217 | 1.40E-05 | 0.99208 | 0.435281 |
| Combined parent's age at death (z-score) | rs141826741 | 13:19201462_T_C | 0.97913 | 0.126047 | 0.028985 | 1.40E-05 | 0.82353 | 0.302266 |
| Combined parent's age at death (z-score) | rs304780 | 7:80014145_C_A | 0.359813 | -0.03481 | 0.008007 | 1.40E-05 | 0.96445 | 0.960068 |
| Combined parent's age at death (z-score) | rs9870308 | 3:116649571_G_T | 0.914198 | 0.072809 | 0.016742 | 1.40E-05 | 0.64521 | 0.839421 |
| Combined parent's age at death (z-score) | rs9672607 | 15:78797460_T_A | 0.161803 | 0.045514 | 0.010459 | 1.40E-05 | 0.95014 | 0.693027 |
| Combined parent's age at death (z-score) | rs55822928 | 18:36965785_C_T | 0.866516 | 0.048157 | 0.011101 | 1.40E-05 | 0.98904 | 0.950257 |
| Combined parent's age at death (z-score) | rs9646485 | 18:1823698_G_A | 0.852012 | 0.046042 | 0.010605 | 1.40E-05 | 0.99383 | 0.013633 |
| Combined parent's age at death (z-score) | rs141395978 | 9:133310333_C_CT | 0.728461 | 0.036972 | 0.008509 | 1.40E-05 | 0.98313 | 0.524723 |
| Combined parent's age at death (z-score) | rs12949119 | 17:18229452_T_A | 0.568629 | 0.033044 | 0.007597 | 1.40E-05 | 0.99934 | 0.454527 |
| Combined parent's age at death (z-score) | rs117692605 | 11:106545714_A_G | 0.979775 | 0.11677 | 0.026835 | 1.40E-05 | 0.99102 | 0.832505 |
| Combined parent's age at death (z-score) | rs1632765 | 11:106526656_T_C | 0.979742 | 0.116563 | 0.026819 | 1.40E-05 | 0.99044 | 0.778807 |
| Combined parent's age at death (z-score) | rs56946152 | 10:56623884_A_G | 0.888481 | 0.052062 | 0.011972 | 1.40E-05 | 0.99889 | 0.942905 |
| Combined parent's age at death (z-score) | rs75076682 | 4:24137170_G_A | 0.890383 | 0.052201 | 0.012018 | 1.40E-05 | 1 | 0.32762 |
| Combined parent's age at death (z-score) | rs4697438 | 4:24173783_A_T | 0.0987159 | -0.05499 | 0.012649 | 1.40E-05 | 0.9867 | 0.111926 |
| Combined parent's age at death (z-score) | rs75806555 | 14:101189448_C_T | 0.877981 | -0.05031 | 0.011587 | 1.40E-05 | 0.98101 | 0.220419 |
| Combined parent's age at death (z-score) | rs16866299 | 2:179231888_A_G | 0.98 | -0.11684 | 0.027028 | 1.50E-05 | 0.99117 | 0.088859 |
| Combined parent's age at death (z-score) | rs146745360 | 13:19203442_A_G | 0.980126 | 0.125931 | 0.029084 | 1.50E-05 | 0.85675 | 0.386216 |
| Combined parent's age at death (z-score) | rs35029802 | 5:152533739_G_T | 0.787272 | -0.03992 | 0.009206 | 1.50E-05 | 0.99435 | 0.456037 |
| Combined parent's age at death (z-score) | rs201760910 | 19:33597067_TAA_T | 0.978868 | 0.114193 | 0.026357 | 1.50E-05 | 0.97795 | 0.628232 |
| Combined parent's age at death (z-score) | rs191481640 | 21:41820767_T_A | 0.987175 | -0.15101 | 0.034914 | 1.50E-05 | 0.91616 | 0.909377 |
| Combined parent's age at death (z-score) | rs1502230 | 18:50993648_G_A | 0.442382 | 0.032964 | 0.007614 | 1.50E-05 | 0.98568 | 0.87914 |
| Combined parent's age at death (z-score) | 11:41187579_CA_C | 11:41187579_C_G | 0.770227 | -0.0417 | 0.009618 | 1.50E-05 | 0.9166 | 0.355631 |
| Combined parent's age at death (z-score) | 11:41187579_CA_C | 11:41187579_CA_C | 0.770227 | -0.0417 | 0.009618 | 1.50E-05 | 0.85976 | 0.107103 |
| Combined parent's age at death (z-score) | rs117364744 | 10:48392217_C_T | 0.952922 | -0.07989 | 0.018507 | 1.60E-05 | 0.92366 | 0.290583 |
| Combined parent's age at death (z-score) | rs12956641 | 18:50966139_C_T | 0.444448 | 0.032785 | 0.007596 | 1.60E-05 | 0.99098 | 0.365166 |
| Combined parent's age at death (z-score) | rs56079252 | 18:1821017_A_T | 0.851885 | 0.045903 | 0.01063 | 1.60E-05 | 0.98809 | 0.013204 |
| Combined parent's age at death (z-score) | rs72865571 | 18:1823283_A_G | 0.852096 | 0.045828 | 0.010606 | 1.60E-05 | 0.99339 | 0.011929 |
| Combined parent's age at death (z-score) | rs6145642 | 15:78784704_C_CAAAAATTACCAAGATTACCA | 0.618344 | -0.03334 | 0.007723 | 1.60E-05 | 0.99978 | 0.306817 |
| Combined parent's age at death (z-score) | rs11334803 | 17:9720744_CT_C | 0.531072 | 0.033296 | 0.007723 | 1.60E-05 | 0.94504 | 0.630972 |
| Combined parent's age at death (z-score) | rs9902444 | 17:66986162_A_C | 0.0113286 | -0.15836 | 0.036655 | 1.60E-05 | 0.94354 | 0.600081 |
| Combined parent's age at death (z-score) | rs74754074 | 13:110600302_C_T | 0.96886 | -0.0941 | 0.0218 | 1.60E-05 | 0.99453 | 0.961703 |
| Combined parent's age at death (z-score) | rs6832969 | 4:157540844_G_A | 0.739791 | 0.037026 | 0.008576 | 1.60E-05 | 0.99654 | 0.863295 |
| Combined parent's age at death (z-score) | rs576842 | 4:111804004_A_G | 0.226747 | -0.03866 | 0.008972 | 1.60E-05 | 1 | 0.456478 |
| Combined parent's age at death (z-score) | rs12896360 | 14:75278181_A_C | 0.319992 | 0.034764 | 0.008066 | 1.60E-05 | 0.99968 | 0.099816 |
| Combined parent's age at death (z-score) | rs2367812 | 2:33867194_G_T | 0.279418 | 0.03682 | 0.008524 | 1.60E-05 | 0.96389 | 0.70419 |
| Combined parent's age at death (z-score) | rs1267019 | 4:24149452_A_G | 0.889252 | 0.051828 | 0.011999 | 1.60E-05 | 0.99341 | 0.296456 |
| Combined parent's age at death (z-score) | rs1267017 | 4:24151452_T_G | 0.889035 | 0.051708 | 0.011979 | 1.60E-05 | 0.9948 | 0.3111 |
| Combined parent's age at death (z-score) | rs12652843 | 5:98078980_C_T | 0.690803 | -0.0351 | 0.008126 | 1.60E-05 | 0.99711 | 0.978552 |
| Combined parent's age at death (z-score) | rs10559149 | 3:63510527_GCA_G | 0.639511 | -0.03695 | 0.008565 | 1.60E-05 | 0.8294 | 0.066742 |
| Combined parent's age at death (z-score) | rs7642066 | 3:178974671_A_T | 0.456191 | -0.03278 | 0.007605 | 1.60E-05 | 0.99658 | 0.456899 |
| Combined parent's age at death (z-score) | rs7429685 | 3:178978604_T_G | 0.455894 | -0.0328 | 0.007626 | 1.70E-05 | 0.99119 | 0.526344 |
| Combined parent's age at death (z-score) | rs12152961 | 5:152566888_G_A | 0.788636 | -0.03951 | 0.009183 | 1.70E-05 | 1 | 0.051725 |
| Combined parent's age at death (z-score) | rs113516951 | 19:33196436_T_A | 0.980225 | 0.123359 | 0.028658 | 1.70E-05 | 0.88159 | 0.940979 |
| Combined parent's age at death (z-score) | rs79071221 | 6:72128076_G_A | 0.95736 | 0.082368 | 0.019151 | 1.70E-05 | 0.94826 | 0.915516 |
| Combined parent's age at death (z-score) | rs2119745 | 3:77306471_T_C | 0.396927 | -0.03344 | 0.007787 | 1.70E-05 | 0.98084 | 0.12074 |
| Combined parent's age at death (z-score) | rs4741436 | 9:14827373_A_G | 0.759961 | -0.03816 | 0.008863 | 1.70E-05 | 0.98952 | 0.618472 |
| Combined parent's age at death (z-score) | rs2055447 | 18:50974407_A_G | 0.403487 | 0.032953 | 0.007666 | 1.70E-05 | 0.99584 | 0.589631 |
| Combined parent's age at death (z-score) | rs869224 | 18:50977798_A_G | 0.438614 | 0.032608 | 0.007582 | 1.70E-05 | 0.99646 | 0.814682 |
| Combined parent's age at death (z-score) | rs16875229 | 4:24175273_G_A | 0.0985871 | -0.0545 | 0.012688 | 1.70E-05 | 0.98272 | 0.152933 |
| Combined parent's age at death (z-score) | rs2269477 | 4:3184464_G_T | 0.681589 | -0.03486 | 0.008095 | 1.70E-05 | 0.9942 | 0.07449 |
| Combined parent's age at death (z-score) | rs362287 | 4:3257593_C_T | 0.618951 | -0.0337 | 0.007827 | 1.70E-05 | 0.981 | 0.048306 |
| Combined parent's age at death (z-score) | rs78213779 | 13:110587759_T_C | 0.96889 | -0.0942 | 0.021875 | 1.70E-05 | 0.98802 | 0.700455 |
| Combined parent's age at death (z-score) | rs34994073 | 13:110603871_CAA_C | 0.968253 | -0.09339 | 0.021717 | 1.70E-05 | 0.9808 | 0.814746 |
| Combined parent's age at death (z-score) | rs2367813 | 2:33867197_G_T | 0.279898 | 0.036609 | 0.008522 | 1.70E-05 | 0.96339 | 0.715171 |
| Combined parent's age at death (z-score) | rs73919730 | 2:23738368_C_T | 0.90716 | -0.05622 | 0.01307 | 1.70E-05 | 0.97477 | 0.824368 |
| Combined parent's age at death (z-score) | rs17045504 | 2:23733781_A_G | 0.907271 | -0.0558 | 0.013029 | 1.80E-05 | 0.98199 | 0.823979 |
| Combined parent's age at death (z-score) | rs57802314 | 2:23739103_C_CT | 0.906009 | -0.05582 | 0.013016 | 1.80E-05 | 0.97259 | 0.543688 |
| Combined parent's age at death (z-score) | rs3135173 | 4:3257373_T_C | 0.618351 | -0.03354 | 0.007825 | 1.80E-05 | 0.98065 | 0.061589 |
| Combined parent's age at death (z-score) | rs3129320 | 4:3265130_G_C | 0.57501 | -0.03275 | 0.007643 | 1.80E-05 | 0.99416 | 0.100397 |
| Combined parent's age at death (z-score) | rs7695509 | 4:24148456_G_A | 0.890378 | 0.051572 | 0.012027 | 1.80E-05 | 0.99638 | 0.197061 |
| Combined parent's age at death (z-score) | rs79117294 | 13:110582457_G_A | 0.968684 | -0.09343 | 0.021768 | 1.80E-05 | 0.99213 | 0.66799 |
| Combined parent's age at death (z-score) | rs144495348 | 13:110605737_TAC_T | 0.967521 | -0.0923 | 0.02154 | 1.80E-05 | 0.9792 | 0.817325 |
| Combined parent's age at death (z-score) | rs57279878 | 14:75261302_C_CT | 0.315431 | 0.034968 | 0.008148 | 1.80E-05 | 0.98633 | 0.173149 |
| Combined parent's age at death (z-score) | rs1441952 | 3:77307272_A_G | 0.395879 | -0.03344 | 0.007791 | 1.80E-05 | 0.98062 | 0.145158 |
| Combined parent's age at death (z-score) | rs540065432 | 19:53547789_C_CAAAA | 0.591251 | 0.0348 | 0.00812 | 1.80E-05 | 0.88452 | 0.18978 |
| Combined parent's age at death (z-score) | rs143789864 | 18:1816783_GTTGT_G | 0.956441 | 0.084218 | 0.019661 | 1.80E-05 | 0.87306 | 0.097163 |
| Combined parent's age at death (z-score) | rs12789803 | 11:130581865_C_T | 0.759943 | 0.038134 | 0.008898 | 1.80E-05 | 0.96761 | 0.000623 |
| Combined parent's age at death (z-score) | rs11792010 | 9:14816478_A_G | 0.758614 | -0.038 | 0.008848 | 1.80E-05 | 0.98963 | 0.670579 |
| Combined parent's age at death (z-score) | rs192869790 | 9:21559565_C_A | 0.980694 | -0.13268 | 0.031066 | 1.90E-05 | 0.77613 | 0.650309 |
| Combined parent's age at death (z-score) | rs10817261 | 9:114846494_G_A | 0.675457 | -0.03457 | 0.00809 | 1.90E-05 | 0.99337 | 0.973608 |
| Combined parent's age at death (z-score) | rs2134297 | 18:50982831_C_T | 0.438108 | 0.032399 | 0.007579 | 1.90E-05 | 0.99701 | 0.981297 |
| Combined parent's age at death (z-score) | rs71175703 | 18:27624055_AT_A | 0.209305 | 0.040092 | 0.009383 | 1.90E-05 | 0.97551 | 0.155446 |
| Combined parent's age at death (z-score) | rs1386404 | 11:41195670_A_G | 0.84268 | -0.04445 | 0.010392 | 1.90E-05 | 0.99076 | 0.131299 |
| Combined parent's age at death (z-score) | rs10768672 | 11:41231808_C_T | 0.842593 | -0.04445 | 0.010393 | 1.90E-05 | 0.98942 | 0.55321 |
| Combined parent's age at death (z-score) | rs2270431 | 14:75270635_T_C | 0.320118 | 0.034513 | 0.008066 | 1.90E-05 | 0.99941 | 0.098514 |
| Combined parent's age at death (z-score) | rs60143650 | 14:75272572_AT_A | 0.320065 | 0.0345 | 0.008067 | 1.90E-05 | 0.99924 | 0.093175 |
| Combined parent's age at death (z-score) | rs113632539 | 13:110543336_A_G | 0.975139 | -0.10467 | 0.024489 | 1.90E-05 | 0.9748 | 0.631204 |
| Combined parent's age at death (z-score) | rs78989240 | 13:110586235_A_T | 0.968643 | -0.09305 | 0.021747 | 1.90E-05 | 0.99267 | 0.668236 |
| Combined parent's age at death (z-score) | rs76500907 | 13:110589387_G_A | 0.974058 | -0.1018 | 0.023807 | 1.90E-05 | 0.99091 | 0.564794 |
| Combined parent's age at death (z-score) | rs12429912 | 13:110631039_T_C | 0.969376 | -0.09408 | 0.02201 | 1.90E-05 | 0.98926 | 0.961203 |
| Combined parent's age at death (z-score) | rs576838 | 4:111804002_T_C | 0.22656 | -0.0384 | 0.008981 | 1.90E-05 | 0.9988 | 0.501938 |
| Combined parent's age at death (z-score) | rs5861029 | 4:111804051_CA_C | 0.226742 | -0.03838 | 0.008976 | 1.90E-05 | 0.99947 | 0.502158 |
| Combined parent's age at death (z-score) | rs6832283 | 4:157540718_C_T | 0.740516 | 0.036722 | 0.008585 | 1.90E-05 | 0.99684 | 0.952158 |
| Combined parent's age at death (z-score) | rs16875107 | 4:24138261_G_A | 0.890669 | 0.051449 | 0.012035 | 1.90E-05 | 0.99828 | 0.211753 |
| Combined parent's age at death (z-score) | rs11341818;rs368076643 | 4:24156745_GT_G | 0.893319 | 0.052649 | 0.0123 | 1.90E-05 | 0.97501 | 0.386607 |
| Combined parent's age at death (z-score) | rs10023809 | 4:24160980_T_C | 0.8904 | 0.051526 | 0.012051 | 1.90E-05 | 0.99262 | 0.241313 |
| Combined parent's age at death (z-score) | rs362282 | 4:3259671_G_A | 0.618612 | -0.03333 | 0.007794 | 1.90E-05 | 0.9906 | 0.02774 |
| Combined parent's age at death (z-score) | rs10511056 | 3:77309810_A_C | 0.406787 | -0.03316 | 0.007747 | 1.90E-05 | 0.981 | 0.406902 |
| Combined parent's age at death (z-score) | rs12489410 | 3:77311914_G_C | 0.406841 | -0.03312 | 0.007742 | 1.90E-05 | 0.98229 | 0.417138 |
| Combined parent's age at death (z-score) | rs33959041 | 3:77304986_T_G | 0.406856 | -0.03299 | 0.007744 | 2.00E-05 | 0.9818 | 0.406912 |
| Combined parent's age at death (z-score) | rs112291080 | 19:33254757_C_G | 0.981593 | 0.129935 | 0.030434 | 2.00E-05 | 0.83588 | 0.936464 |
| Combined parent's age at death (z-score) | rs2864412 | 3:178981341_T_A | 0.522948 | -0.03255 | 0.007637 | 2.00E-05 | 0.98433 | 0.839706 |
| Combined parent's age at death (z-score) | rs74643186 | 6:72133319_C_T | 0.956714 | 0.080637 | 0.018906 | 2.00E-05 | 0.95947 | 0.834315 |
| Combined parent's age at death (z-score) | rs77334025 | 6:72134226_T_C | 0.956688 | 0.080684 | 0.018899 | 2.00E-05 | 0.95951 | 0.83434 |
| Combined parent's age at death (z-score) | rs140332428 | 6:72135659_TATTTG_T | 0.956436 | 0.080014 | 0.01875 | 2.00E-05 | 0.96889 | 0.808632 |
| Combined parent's age at death (z-score) | rs576414456 | 6:44625918_C_A | 0.980557 | 0.140994 | 0.033023 | 2.00E-05 | 0.67102 | 0.485534 |
| Combined parent's age at death (z-score) | rs12964168 | 18:27592030_A_T | 0.212133 | 0.039455 | 0.009257 | 2.00E-05 | 0.99369 | 0.128603 |
| Combined parent's age at death (z-score) | rs35882434 | 18:50979580_C_T | 0.439165 | 0.032366 | 0.007582 | 2.00E-05 | 0.99674 | 0.865107 |
| Combined parent's age at death (z-score) | rs10502978 | 18:50981440_C_A | 0.437544 | 0.032402 | 0.00759 | 2.00E-05 | 0.99522 | 0.883466 |
| Combined parent's age at death (z-score) | rs10961728 | 9:14817302_G_A | 0.759601 | -0.03765 | 0.008836 | 2.00E-05 | 0.99532 | 0.62984 |
| Combined parent's age at death (z-score) | rs66526375 | 10:125696485_G_A | 0.85863 | 0.046345 | 0.010871 | 2.00E-05 | 0.9815 | 0.465608 |
| Combined parent's age at death (z-score) | rs182439088 | 8:68051368_C_T | 0.97957 | -0.11885 | 0.027875 | 2.00E-05 | 0.9037 | 0.556429 |
| Combined parent's age at death (z-score) | rs60256324 | 10:56623898_T_G | 0.888177 | 0.051005 | 0.011963 | 2.00E-05 | 1 | 0.577006 |
| Combined parent's age at death (z-score) | rs16875183 | 4:24157489_A_G | 0.890411 | 0.051388 | 0.012052 | 2.00E-05 | 0.99244 | 0.207195 |
| Combined parent's age at death (z-score) | rs61507117 | 2:23733446_C_T | 0.907321 | -0.05553 | 0.013021 | 2.00E-05 | 0.98371 | 0.797343 |
| Combined parent's age at death (z-score) | rs492291 | 4:111804548_T_A | 0.226856 | -0.03829 | 0.008985 | 2.00E-05 | 0.99727 | 0.518024 |
| Combined parent's age at death (z-score) | rs79200751 | 13:110592560_A_G | 0.968793 | -0.09292 | 0.02178 | 2.00E-05 | 0.9941 | 0.810726 |
| Combined parent's age at death (z-score) | rs554348642 | 22:38220132_G_GT | 0.450664 | 0.032603 | 0.007649 | 2.00E-05 | 0.97066 | 0.045677 |
| Combined parent's age at death (z-score) | rs78105305 | 13:110586327_G_A | 0.96895 | -0.09311 | 0.021871 | 2.10E-05 | 0.99011 | 1 |
| Combined parent's age at death (z-score) | rs12429915 | 13:110631086_T_G | 0.969384 | -0.09365 | 0.022015 | 2.10E-05 | 0.98956 | 1 |
| Combined parent's age at death (z-score) | rs112296580 | 13:110637926_G_C | 0.970483 | -0.09511 | 0.022382 | 2.10E-05 | 0.99107 | 0.762746 |
| Combined parent's age at death (z-score) | rs13110084 | 4:24150414_C_T | 0.890266 | 0.051123 | 0.012018 | 2.10E-05 | 0.99666 | 0.182306 |
| Combined parent's age at death (z-score) | rs137988645 | 4:24155845_CTGTGTTCTACCACAG_C | 0.891751 | 0.051622 | 0.012149 | 2.10E-05 | 0.98721 | 0.254836 |
| Combined parent's age at death (z-score) | rs7656884 | 4:24158029_T_C | 0.890188 | 0.051168 | 0.012034 | 2.10E-05 | 0.99352 | 0.213486 |
| Combined parent's age at death (z-score) | rs1705096 | 4:24163223_C_G | 0.890446 | 0.051262 | 0.012056 | 2.10E-05 | 0.99255 | 0.265717 |
| Combined parent's age at death (z-score) | rs1267026 | 4:24164708_T_G | 0.889951 | 0.051266 | 0.012043 | 2.10E-05 | 0.99095 | 0.287535 |
| Combined parent's age at death (z-score) | rs11173833 | 12:61615275_C_A | 0.109411 | 0.051508 | 0.012094 | 2.10E-05 | 0.98874 | 0.604603 |
| Combined parent's age at death (z-score) | rs28454578 | 4:157529918_C_A | 0.74074 | 0.036563 | 0.008596 | 2.10E-05 | 0.9948 | 0.952138 |
| Combined parent's age at death (z-score) | rs13434539 | 4:157530542_T_C | 0.740583 | 0.03659 | 0.008594 | 2.10E-05 | 0.99517 | 0.958133 |
| Combined parent's age at death (z-score) | rs2367816 | 2:33873296_A_G | 0.239778 | 0.037577 | 0.008834 | 2.10E-05 | 0.98778 | 0.721639 |
| Combined parent's age at death (z-score) | rs114112366 | 5:112000558_G_A | 0.987918 | -0.14642 | 0.034416 | 2.10E-05 | 1 | 0.620426 |
| Combined parent's age at death (z-score) | rs79798970 | 6:72127707_T_A | 0.956789 | 0.080565 | 0.018925 | 2.10E-05 | 0.95936 | 0.753425 |
| Combined parent's age at death (z-score) | rs79333840 | 6:72128264_C_T | 0.956783 | 0.080559 | 0.018924 | 2.10E-05 | 0.95929 | 0.807027 |
| Combined parent's age at death (z-score) | rs11874751 | 18:50986210_G_T | 0.438159 | 0.032265 | 0.007578 | 2.10E-05 | 0.99729 | 0.985972 |
| Combined parent's age at death (z-score) | rs16956779 | 18:50988070_C_T | 0.438176 | 0.032256 | 0.007578 | 2.10E-05 | 0.99722 | 0.957935 |
| Combined parent's age at death (z-score) | rs2036415 | 18:50989175_G_C | 0.437944 | 0.032258 | 0.007578 | 2.10E-05 | 0.99776 | 0.957928 |
| Combined parent's age at death (z-score) | rs1964667 | 18:50992956_C_T | 0.438998 | 0.03222 | 0.007581 | 2.10E-05 | 0.99614 | 0.971959 |
| Combined parent's age at death (z-score) | rs8090013 | 18:27610212_T_C | 0.212172 | 0.039416 | 0.009273 | 2.10E-05 | 0.99039 | 0.118386 |
| Combined parent's age at death (z-score) | rs71322070 | 21:23089628_ATACTT_A | 0.645084 | -0.03385 | 0.007952 | 2.10E-05 | 0.96964 | 0.874694 |
| Combined parent's age at death (z-score) | rs7184856 | 16:86614217_G_C | 0.888343 | -0.05257 | 0.012348 | 2.10E-05 | 0.9312 | 0.129796 |
| Combined parent's age at death (z-score) | rs10961729 | 9:14817420_A_G | 0.759494 | -0.0376 | 0.008832 | 2.10E-05 | 0.99568 | 0.652665 |
| Combined parent's age at death (z-score) | rs7023244 | 9:14819370_G_T | 0.761 | -0.0376 | 0.008833 | 2.10E-05 | 1 | 0.589685 |
| Combined parent's age at death (z-score) | rs62573226 | 9:79929722_C_T | 0.955601 | -0.07763 | 0.018242 | 2.10E-05 | 1 | 0.374145 |
| Combined parent's age at death (z-score) | rs187771847 | 9:110509903_G_C | 0.988655 | -0.16342 | 0.038381 | 2.10E-05 | 0.85873 | 0.521771 |
| Combined parent's age at death (z-score) | rs201149181 | 17:9719921_GTGTGTGTC_G | 0.566126 | 0.032789 | 0.0077 | 2.10E-05 | 0.96453 | 0.837331 |
| Combined parent's age at death (z-score) | rs7210822 | 17:17689473_T_C | 0.019899 | 0.118458 | 0.027845 | 2.10E-05 | 0.92701 | 0.125591 |
| Combined parent's age at death (z-score) | rs1975833 | 17:9422035_A_G | 0.217734 | -0.0392 | 0.009229 | 2.20E-05 | 0.9771 | 0.710624 |
| Combined parent's age at death (z-score) | rs147696620 | 17:78427832_G_GA | 0.914883 | -0.06083 | 0.014339 | 2.20E-05 | 0.88035 | 0.043003 |
| Combined parent's age at death (z-score) | rs4318281 | 18:27606712_G_C | 0.214119 | 0.039062 | 0.009208 | 2.20E-05 | 0.9977 | 0.122586 |
| Combined parent's age at death (z-score) | rs3215140 | 15:78790735_TG_T | 0.147531 | 0.046886 | 0.011059 | 2.20E-05 | 0.92163 | 0.712262 |
| Combined parent's age at death (z-score) | rs12605017 | 18:50979790_C_T | 0.438212 | 0.032209 | 0.007583 | 2.20E-05 | 0.99642 | 0.911337 |
| Combined parent's age at death (z-score) | rs16956772 | 18:50985078_A_G | 0.438232 | 0.032142 | 0.007579 | 2.20E-05 | 0.99717 | 0.97195 |
| Combined parent's age at death (z-score) | rs11083001 | 18:50987446_C_G | 0.438048 | 0.03218 | 0.007578 | 2.20E-05 | 0.99764 | 0.981296 |
| Combined parent's age at death (z-score) | rs11659924 | 18:50987648_T_G | 0.438047 | 0.032166 | 0.007577 | 2.20E-05 | 0.99765 | 0.995324 |
| Combined parent's age at death (z-score) | rs62098320 | 18:50991321_C_T | 0.438958 | 0.032172 | 0.007581 | 2.20E-05 | 0.99596 | 0.985978 |
| Combined parent's age at death (z-score) | rs1814001 | 18:50992215_A_G | 0.438985 | 0.03218 | 0.007581 | 2.20E-05 | 0.99611 | 0.976632 |
| Combined parent's age at death (z-score) | rs755473 | 18:1823877_A_G | 0.852749 | 0.045149 | 0.010643 | 2.20E-05 | 0.99022 | 0.012354 |
| Combined parent's age at death (z-score) | rs72867237 | 18:1837628_A_T | 0.863146 | 0.046399 | 0.010941 | 2.20E-05 | 0.99047 | 0.013817 |
| Combined parent's age at death (z-score) | rs2168109 | 10:56607303_T_G | 0.886708 | 0.050599 | 0.011924 | 2.20E-05 | 0.99547 | 0.364998 |
| Combined parent's age at death (z-score) | rs10810249 | 9:14806848_C_A | 0.760128 | -0.03787 | 0.008923 | 2.20E-05 | 0.97735 | 0.657582 |
| Combined parent's age at death (z-score) | rs10756614 | 9:14807218_T_G | 0.759165 | -0.03786 | 0.008914 | 2.20E-05 | 0.97704 | 0.635975 |
| Combined parent's age at death (z-score) | rs7186936 | 16:86614222_A_C | 0.88817 | -0.05231 | 0.012338 | 2.20E-05 | 0.93079 | 0.112613 |
| Combined parent's age at death (z-score) | rs76884865 | 16:12778817_G_A | 0.976634 | -0.10521 | 0.024815 | 2.20E-05 | 1 | 0.092961 |
| Combined parent's age at death (z-score) | rs3135172 | 4:3258735_A_G | 0.618597 | -0.03312 | 0.007794 | 2.20E-05 | 0.99041 | 0.031405 |
| Combined parent's age at death (z-score) | rs113660805 | 13:110608372_G_T | 0.97405 | -0.10062 | 0.023737 | 2.20E-05 | 0.99618 | 0.908073 |
| Combined parent's age at death (z-score) | rs75098925 | 13:110661885_C_CT | 0.97018 | -0.09544 | 0.022506 | 2.20E-05 | 0.97017 | 0.34724 |
| Combined parent's age at death (z-score) | rs117732233 | 22:22766730_G_A | 0.989068 | -0.17013 | 0.040133 | 2.20E-05 | 0.80886 | 0.898322 |
| Combined parent's age at death (z-score) | rs577781 | 4:111804131_A_G | 0.226777 | -0.03812 | 0.008981 | 2.20E-05 | 0.99827 | 0.512637 |
| Combined parent's age at death (z-score) | rs11441508 | 14:101185421_T_TA | 0.809013 | -0.04088 | 0.009643 | 2.20E-05 | 0.98683 | 0.700664 |
| Combined parent's age at death (z-score) | rs2324724 | 3:77306695_T_C | 0.406742 | -0.03286 | 0.007745 | 2.20E-05 | 0.98172 | 0.437925 |
| Combined parent's age at death (z-score) | rs304778 | 7:80012026_A_T | 0.360009 | -0.03399 | 0.008012 | 2.20E-05 | 0.96323 | 0.870773 |
| Combined parent's age at death (z-score) | rs4683988 | 3:77300562_G_A | 0.410041 | -0.03279 | 0.007741 | 2.30E-05 | 0.97991 | 0.464109 |
| Combined parent's age at death (z-score) | rs34090148 | 18:50979916_GT_G | 0.43832 | 0.032124 | 0.007581 | 2.30E-05 | 0.9967 | 0.883523 |
| Combined parent's age at death (z-score) | rs16956767 | 18:50984634_T_C | 0.438106 | 0.03208 | 0.007578 | 2.30E-05 | 0.9975 | 0.985972 |
| Combined parent's age at death (z-score) | rs11083002 | 18:50988841_A_C | 0.439069 | 0.032111 | 0.007586 | 2.30E-05 | 0.99437 | 0.990652 |
| Combined parent's age at death (z-score) | rs1502232 | 18:50990508_A_G | 0.438976 | 0.032072 | 0.00758 | 2.30E-05 | 0.99611 | 0.976632 |
| Combined parent's age at death (z-score) | rs10853632 | 18:50993998_C_T | 0.438034 | 0.03213 | 0.007588 | 2.30E-05 | 0.99469 | 0.95793 |
| Combined parent's age at death (z-score) | rs10502979 | 18:51047606_C_G | 0.53503 | 0.031949 | 0.007555 | 2.30E-05 | 0.99284 | 0.598246 |
| Combined parent's age at death (z-score) | rs62573187 | 9:79839447_A_G | 0.947259 | -0.07137 | 0.016862 | 2.30E-05 | 0.99275 | 0.467552 |
| Combined parent's age at death (z-score) | rs2287400 | 14:75182937_G_A | 0.336813 | 0.033773 | 0.007981 | 2.30E-05 | 0.99469 | 0.321403 |
| Combined parent's age at death (z-score) | rs362289 | 4:3256378_G_A | 0.619299 | -0.03312 | 0.007827 | 2.30E-05 | 0.9812 | 0.053297 |
| Combined parent's age at death (z-score) | rs362283 | 4:3259558_T_C | 0.618446 | -0.03302 | 0.007794 | 2.30E-05 | 0.99037 | 0.030026 |
| Combined parent's age at death (z-score) | rs10213261 | 4:24156429_A_G | 0.890269 | 0.050957 | 0.012039 | 2.30E-05 | 0.99345 | 0.21327 |
| Combined parent's age at death (z-score) | rs7696209 | 4:24156758_T_C | 0.890346 | 0.051004 | 0.012039 | 2.30E-05 | 0.99397 | 0.197106 |
| Combined parent's age at death (z-score) | rs6448240 | 4:24156842_A_T | 0.89022 | 0.051014 | 0.012037 | 2.30E-05 | 0.99324 | 0.213486 |
| Combined parent's age at death (z-score) | rs536719489 | 4:182633981_A_T | 0.98843 | 0.186845 | 0.044159 | 2.30E-05 | 0.62312 | 0.007941 |
| Combined parent's age at death (z-score) | rs13435651 | 4:157530143_C_T | 0.740248 | 0.036416 | 0.008599 | 2.30E-05 | 0.9935 | 0.964149 |
| Combined parent's age at death (z-score) | rs950505 | 4:157539967_G_A | 0.740188 | 0.036379 | 0.008583 | 2.30E-05 | 0.99643 | 0.952195 |
| Combined parent's age at death (z-score) | rs4690870 | 4:157540404_G_A | 0.740151 | 0.036309 | 0.008583 | 2.30E-05 | 0.99632 | 0.922397 |
| Combined parent's age at death (z-score) | rs139962365 | 13:110537982_AT_A | 0.972734 | -0.09852 | 0.02328 | 2.30E-05 | 0.98564 | 0.303564 |
| Combined parent's age at death (z-score) | rs113662974 | 13:110587467_G_A | 0.968597 | -0.09204 | 0.021748 | 2.30E-05 | 0.99132 | 0.739061 |
| Combined parent's age at death (z-score) | rs111924867 | 13:110595691_C_A | 0.968753 | -0.09222 | 0.021759 | 2.30E-05 | 0.99457 | 0.848254 |
| Combined parent's age at death (z-score) | rs112552193 | 13:110597723_C_A | 0.974073 | -0.10074 | 0.02377 | 2.30E-05 | 0.99424 | 0.817325 |
| Combined parent's age at death (z-score) | rs113937309 | 13:110607200_C_T | 0.968792 | -0.092 | 0.021736 | 2.30E-05 | 0.99783 | 1 |
| Combined parent's age at death (z-score) | rs1333986 | 13:110582003_G_A | 0.974167 | -0.10114 | 0.023937 | 2.40E-05 | 0.98366 | 0.488562 |
| Combined parent's age at death (z-score) | rs113293725 | 13:110604175_G_A | 0.968787 | -0.09199 | 0.021758 | 2.40E-05 | 0.99566 | 0.923653 |
| Combined parent's age at death (z-score) | rs111950804 | 13:110609347_A_C | 0.9688 | -0.09188 | 0.021736 | 2.40E-05 | 0.99796 | 0.961796 |
| Combined parent's age at death (z-score) | rs77074125 | 13:110613635_A_G | 0.969073 | -0.09224 | 0.02185 | 2.40E-05 | 0.99534 | 1 |
| Combined parent's age at death (z-score) | rs55962025 | 4:3112109_A_C | 0.639242 | -0.03321 | 0.007864 | 2.40E-05 | 0.99317 | 0.103748 |
| Combined parent's age at death (z-score) | rs67950027 | 4:3152786_A_C | 0.669823 | -0.03428 | 0.008107 | 2.40E-05 | 0.97591 | 0.02293 |
| Combined parent's age at death (z-score) | rs2798226 | 4:3255292_C_T | 0.603186 | -0.03347 | 0.00793 | 2.40E-05 | 0.93588 | 0.220029 |
| Combined parent's age at death (z-score) | rs4683968 | 3:77310112_G_A | 0.4065 | -0.03268 | 0.007744 | 2.40E-05 | 0.98207 | 0.466563 |
| Combined parent's age at death (z-score) | rs77155088 | 5:152552273_A_T | 0.786108 | -0.03885 | 0.009189 | 2.40E-05 | 0.99224 | 0.274524 |
| Combined parent's age at death (z-score) | rs62573189 | 9:79849874_C_G | 0.94777 | -0.07147 | 0.016926 | 2.40E-05 | 0.99401 | 0.395039 |
| Combined parent's age at death (z-score) | rs2776113 | 21:23069208_G_A | 0.646551 | 0.033194 | 0.007851 | 2.40E-05 | 0.99387 | 0.479027 |
| Combined parent's age at death (z-score) | rs143828186 | 17:6628183_T_C | 0.989853 | -0.17551 | 0.041552 | 2.40E-05 | 0.80605 | 0.543839 |
| Combined parent's age at death (z-score) | rs35409749 | 18:50981654_A_C | 0.442058 | 0.032068 | 0.007594 | 2.40E-05 | 0.99256 | 0.930086 |
| Combined parent's age at death (z-score) | rs2036414 | 18:50994661_A_G | 0.439313 | 0.032034 | 0.007586 | 2.40E-05 | 0.99467 | 0.939299 |
| Combined parent's age at death (z-score) | rs2036410 | 18:50995585_T_C | 0.438815 | 0.032056 | 0.007592 | 2.40E-05 | 0.99361 | 0.90672 |
| Combined parent's age at death (z-score) | rs193133088 | 18:1818373_C_T | 0.938903 | 0.072484 | 0.017157 | 2.40E-05 | 0.8447 | 0.433651 |
| Combined parent's age at death (z-score) | rs1371273 | 18:1837016_G_A | 0.864371 | 0.046396 | 0.010985 | 2.40E-05 | 0.98817 | 0.008247 |
| Combined parent's age at death (z-score) | rs16906592 | 10:56606168_T_C | 0.887042 | 0.050447 | 0.011943 | 2.40E-05 | 0.99514 | 0.371672 |
| Combined parent's age at death (z-score) | rs12376167 | 9:14817042_C_T | 0.759842 | -0.03728 | 0.00884 | 2.50E-05 | 0.99491 | 0.652334 |
| Combined parent's age at death (z-score) | rs10961731 | 9:14820782_C_T | 0.759853 | -0.03725 | 0.008835 | 2.50E-05 | 0.99461 | 0.580003 |
| Combined parent's age at death (z-score) | rs12364931 | 11:41195359_G_A | 0.842228 | -0.04371 | 0.01036 | 2.50E-05 | 0.99415 | 0.165345 |
| Combined parent's age at death (z-score) | rs77443602 | 11:9026361_A_G | 0.920428 | -0.05886 | 0.013952 | 2.50E-05 | 0.99005 | 0.692405 |
| Combined parent's age at death (z-score) | rs117158759 | 11:108957523_C_T | 0.977374 | -0.10645 | 0.02527 | 2.50E-05 | 1 | 0.430229 |
| Combined parent's age at death (z-score) | rs1371274 | 18:1837007_A_C | 0.864314 | 0.046281 | 0.01098 | 2.50E-05 | 0.98856 | 0.007413 |
| Combined parent's age at death (z-score) | rs12607295 | 18:50981685_A_G | 0.44096 | 0.031992 | 0.007595 | 2.50E-05 | 0.99247 | 0.920751 |
| Combined parent's age at death (z-score) | rs11875490 | 18:50987044_C_T | 0.438325 | 0.031931 | 0.007576 | 2.50E-05 | 0.99771 | 0.985973 |
| Combined parent's age at death (z-score) | rs11662896 | 18:50987590_A_G | 0.438307 | 0.031933 | 0.007576 | 2.50E-05 | 0.99777 | 0.985973 |
| Combined parent's age at death (z-score) | rs34876540 | 18:50987754_A_G | 0.438307 | 0.031933 | 0.007576 | 2.50E-05 | 0.99777 | 0.985973 |
| Combined parent's age at death (z-score) | rs2879567 | 18:51021360_A_C | 0.533937 | 0.031938 | 0.007574 | 2.50E-05 | 0.98656 | 0.834916 |
| Combined parent's age at death (z-score) | rs147758611 | 10:105300191_C_T | 0.984085 | 0.129988 | 0.03085 | 2.50E-05 | 0.94348 | 0.359724 |
| Combined parent's age at death (z-score) | rs59613878 | 4:3139152_C_T | 0.692373 | -0.0344 | 0.008162 | 2.50E-05 | 0.99541 | 0.142771 |
| Combined parent's age at death (z-score) | rs1006798 | 4:3258373_A_G | 0.618685 | -0.03286 | 0.007793 | 2.50E-05 | 0.9909 | 0.030437 |
| Combined parent's age at death (z-score) | rs74933693 | 13:110597520_C_T | 0.968942 | -0.09212 | 0.021839 | 2.50E-05 | 0.99257 | 0.885183 |
| Combined parent's age at death (z-score) | rs73615283 | 13:110625342_G_T | 0.957859 | -0.07954 | 0.018887 | 2.50E-05 | 0.98316 | 0.94347 |
| Combined parent's age at death (z-score) | rs73615290 | 13:110628599_C_T | 0.958306 | -0.07996 | 0.018972 | 2.50E-05 | 0.98372 | 0.886115 |
| Combined parent's age at death (z-score) | rs7303934 | 12:61612281_A_C | 0.112544 | 0.050167 | 0.011914 | 2.50E-05 | 0.99384 | 0.696498 |
| Combined parent's age at death (z-score) | rs7959424 | 12:61612899_T_C | 0.112544 | 0.050169 | 0.011915 | 2.50E-05 | 0.99371 | 0.739673 |
| Combined parent's age at death (z-score) | rs148534114 | 5:179031286_CAG_C | 0.982145 | -0.12115 | 0.028747 | 2.50E-05 | 0.97787 | 0.571216 |
| Combined parent's age at death (z-score) | rs9811457 | 3:77307669_G_A | 0.407261 | -0.03261 | 0.007743 | 2.50E-05 | 0.98172 | 0.383849 |
| Combined parent's age at death (z-score) | rs200407828 | 3:13690069_TG_T | 0.982854 | -0.12958 | 0.030836 | 2.60E-05 | 0.88336 | 0.559899 |
| Combined parent's age at death (z-score) | rs11794906 | 9:14814484_C_A | 0.759488 | -0.03723 | 0.008852 | 2.60E-05 | 0.99094 | 0.624367 |
| Combined parent's age at death (z-score) | rs7019481 | 9:14818797_G_T | 0.759836 | -0.03711 | 0.008825 | 2.60E-05 | 0.99833 | 0.629695 |
| Combined parent's age at death (z-score) | rs557918194 | 9:114848843_AGAG_A | 0.676321 | -0.03405 | 0.008089 | 2.60E-05 | 0.99634 | 0.936645 |
| Combined parent's age at death (z-score) | rs565453041 | 9:4257570_G_A | 0.982051 | 0.123486 | 0.029362 | 2.60E-05 | 0.93013 | 0.411529 |
| Combined parent's age at death (z-score) | rs8090945 | 18:50967252_C_T | 0.407827 | 0.032208 | 0.007663 | 2.60E-05 | 0.99455 | 0.129265 |
| Combined parent's age at death (z-score) | rs12709772 | 18:50993187_T_C | 0.438152 | 0.031893 | 0.007582 | 2.60E-05 | 0.99606 | 0.985972 |
| Combined parent's age at death (z-score) | rs4636995 | 18:27608394_A_T | 0.214033 | 0.038755 | 0.009206 | 2.60E-05 | 0.99858 | 0.124655 |
| Combined parent's age at death (z-score) | rs79395477 | 18:1827189_C_T | 0.854288 | 0.045015 | 0.010704 | 2.60E-05 | 0.98598 | 0.00777 |
| Combined parent's age at death (z-score) | rs538642611 | 11:32003830_G_GCAACTCAGCCCCACTCCACTTGT | 0.967395 | -0.09087 | 0.02162 | 2.60E-05 | 0.95186 | 0.714019 |
| Combined parent's age at death (z-score) | rs77848559 | 11:9028592_A_G | 0.920608 | -0.05873 | 0.013951 | 2.60E-05 | 0.99261 | 0.676488 |
| Combined parent's age at death (z-score) | rs10877587 | 12:61602555_C_T | 0.112659 | 0.050111 | 0.011912 | 2.60E-05 | 0.99305 | 0.750844 |
| Combined parent's age at death (z-score) | rs7155375 | 14:101188731_C_T | 0.649124 | -0.03373 | 0.008016 | 2.60E-05 | 0.96866 | 0.297955 |
| Combined parent's age at death (z-score) | rs74356828 | 13:110560889_T_C | 0.973834 | -0.09957 | 0.023675 | 2.60E-05 | 0.99272 | 0.36509 |
| Combined parent's age at death (z-score) | rs363066 | 4:3135953_T_G | 0.692585 | -0.03433 | 0.008167 | 2.60E-05 | 0.99422 | 0.144398 |
| Combined parent's age at death (z-score) | rs363064 | 4:3141410_C_T | 0.693145 | -0.03439 | 0.00817 | 2.60E-05 | 0.99556 | 0.165863 |
| Combined parent's age at death (z-score) | rs28406606 | 4:25454045_A_G | 0.944843 | 0.070865 | 0.016856 | 2.60E-05 | 0.95338 | 0.267683 |
| Combined parent's age at death (z-score) | rs16875102 | 4:24136683_A_G | 0.890512 | 0.050841 | 0.012123 | 2.70E-05 | 0.98113 | 0.235099 |
| Combined parent's age at death (z-score) | rs34541506 | 4:24144076_G_A | 0.88997 | 0.050436 | 0.01202 | 2.70E-05 | 0.99513 | 0.193428 |
| Combined parent's age at death (z-score) | rs11445574 | 4:3212336_T_TG | 0.662877 | -0.03356 | 0.00799 | 2.70E-05 | 0.99326 | 0.048117 |
| Combined parent's age at death (z-score) | rs1395537 | 12:61592056_G_A | 0.112599 | 0.050036 | 0.011913 | 2.70E-05 | 0.99335 | 0.728954 |
| Combined parent's age at death (z-score) | rs1839157 | 2:33869228_T_C | 0.22924 | 0.037678 | 0.008979 | 2.70E-05 | 0.98804 | 0.863764 |
| Combined parent's age at death (z-score) | rs72848089 | 2:23728727_C_G | 0.905794 | -0.05398 | 0.012861 | 2.70E-05 | 0.9933 | 0.660131 |
| Combined parent's age at death (z-score) | rs967007 | 2:231043864_T_G | 0.20053 | 0.040205 | 0.009584 | 2.70E-05 | 0.95728 | 0.512991 |
| Combined parent's age at death (z-score) | rs75581297 | 13:110620205_C_A | 0.959226 | -0.08037 | 0.01915 | 2.70E-05 | 0.9845 | 0.912414 |
| Combined parent's age at death (z-score) | rs10144845 | 14:75237770_C_T | 0.320149 | 0.033822 | 0.008063 | 2.70E-05 | 0.99961 | 0.093261 |
| Combined parent's age at death (z-score) | rs4420638 | 19:45422946_A_G | 0.809825 | 0.040144 | 0.009566 | 2.70E-05 | 1 | 0.388669 |
| Combined parent's age at death (z-score) | rs111360232 | 19:33597074_A_T | 0.978762 | 0.11096 | 0.026437 | 2.70E-05 | 0.96673 | 0.629738 |
| Combined parent's age at death (z-score) | rs13235271 | 7:28903158_G_A | 0.877799 | 0.048555 | 0.011558 | 2.70E-05 | 0.98309 | 0.587057 |
| Combined parent's age at death (z-score) | rs11036286 | 11:41226338_A_G | 0.838131 | -0.04308 | 0.010268 | 2.70E-05 | 0.98929 | 0.428894 |
| Combined parent's age at death (z-score) | rs72151649 | 17:18176002_A_AT | 0.577715 | 0.032535 | 0.007754 | 2.70E-05 | 0.96546 | 0.294524 |
| Combined parent's age at death (z-score) | rs116942258 | 18:1820319_A_G | 0.854688 | 0.045307 | 0.010801 | 2.70E-05 | 0.97058 | 0.014605 |
| Combined parent's age at death (z-score) | rs1371272 | 18:1837204_G_A | 0.863036 | 0.045845 | 0.010922 | 2.70E-05 | 0.9929 | 0.010144 |
| Combined parent's age at death (z-score) | rs5013791 | 18:50988821_G_A | 0.439349 | 0.031853 | 0.007584 | 2.70E-05 | 0.99447 | 0.967297 |
| Combined parent's age at death (z-score) | rs4799361 | 18:27618328_C_T | 0.212857 | 0.038804 | 0.009248 | 2.70E-05 | 0.99385 | 0.105694 |
| Combined parent's age at death (z-score) | rs4509408 | 9:79688654_A_G | 0.933036 | -0.06307 | 0.015037 | 2.70E-05 | 0.99769 | 0.98161 |
| Combined parent's age at death (z-score) | rs577685194 | 9:114848847_AG_A | 0.676212 | -0.03389 | 0.00809 | 2.80E-05 | 0.99611 | 0.968302 |
| Combined parent's age at death (z-score) | rs76060406 | 11:9026949_T_C | 0.921249 | -0.05843 | 0.013961 | 2.80E-05 | 1 | 0.496547 |
| Combined parent's age at death (z-score) | rs58712598 | 11:21150941_G_A | 0.805568 | -0.0401 | 0.009568 | 2.80E-05 | 0.99301 | 0.112909 |
| Combined parent's age at death (z-score) | rs11661419 | 18:27595439_T_G | 0.212297 | 0.038657 | 0.009228 | 2.80E-05 | 0.9989 | 0.149534 |
| Combined parent's age at death (z-score) | rs497110 | 1:51727687_C_T | 0.961625 | -0.08216 | 0.019599 | 2.80E-05 | 0.9954 | 0.410488 |
| Combined parent's age at death (z-score) | rs6819230 | 4:76180227_G_C | 0.468342 | 0.031745 | 0.007582 | 2.80E-05 | 0.98909 | 0.816859 |
| Combined parent's age at death (z-score) | rs4758807 | 12:61614536_G_C | 0.110024 | 0.05047 | 0.012042 | 2.80E-05 | 0.99267 | 0.658597 |
| Combined parent's age at death (z-score) | rs7322474 | 13:110628276_A_C | 0.958168 | -0.07933 | 0.018921 | 2.80E-05 | 0.98606 | 0.943122 |
| Combined parent's age at death (z-score) | rs73252111 | 14:48966412_G_T | 0.920793 | 0.05819 | 0.013903 | 2.80E-05 | 1 | 0.246723 |
| Combined parent's age at death (z-score) | rs9876344 | 3:77305891_G_A | 0.40722 | -0.03242 | 0.007742 | 2.80E-05 | 0.98205 | 0.393704 |
| Combined parent's age at death (z-score) | rs1372423 | 3:77310364_G_A | 0.406391 | -0.03247 | 0.007745 | 2.80E-05 | 0.98186 | 0.423867 |
| Combined parent's age at death (z-score) | rs146779760 | 6:72138303_ATC_A | 0.956068 | 0.078399 | 0.018719 | 2.80E-05 | 0.96374 | 0.863567 |
| Combined parent's age at death (z-score) | rs56098745 | 20:11554375_C_G | 0.90931 | 0.055012 | 0.013127 | 2.80E-05 | 1 | 0.05503 |
| Combined parent's age at death (z-score) | rs6508634 | 18:27593867_G_A | 0.213058 | 0.038555 | 0.009215 | 2.90E-05 | 1 | 0.1277 |
| Combined parent's age at death (z-score) | rs9960821 | 18:27594909_C_T | 0.212888 | 0.03855 | 0.009227 | 2.90E-05 | 0.99831 | 0.12745 |
| Combined parent's age at death (z-score) | rs236043 | 21:22922109_G_A | 0.467757 | 0.031638 | 0.007561 | 2.90E-05 | 0.99062 | 0.875657 |
| Combined parent's age at death (z-score) | rs140242140 | 11:32000515_G_A | 0.966695 | -0.08913 | 0.021302 | 2.90E-05 | 0.96192 | 0.787771 |
| Combined parent's age at death (z-score) | rs143417053 | 17:18075768_T_C | 0.978537 | -0.11851 | 0.028356 | 2.90E-05 | 0.82322 | 0.06139 |
| Combined parent's age at death (z-score) | rs145978079 | 10:105376021_A_G | 0.983325 | 0.130028 | 0.031093 | 2.90E-05 | 0.89101 | 1 |
| Combined parent's age at death (z-score) | rs8004084 | 14:75220107_T_C | 0.334301 | 0.033417 | 0.007996 | 2.90E-05 | 0.99672 | 0.328943 |
| Combined parent's age at death (z-score) | rs55982413 | 14:75254820_CT_C | 0.304246 | 0.034462 | 0.008239 | 2.90E-05 | 0.98417 | 0.13724 |
| Combined parent's age at death (z-score) | rs57405531 | 13:110556735_C_T | 0.96859 | -0.09111 | 0.021797 | 2.90E-05 | 0.98685 | 0.479479 |
| Combined parent's age at death (z-score) | rs112563030 | 13:110612363_G_A | 0.974214 | -0.09955 | 0.023788 | 2.90E-05 | 1 | 0.953738 |
| Combined parent's age at death (z-score) | rs75206536 | 13:110617464_T_C | 0.958579 | -0.07934 | 0.018984 | 2.90E-05 | 0.98788 | 0.885536 |
| Combined parent's age at death (z-score) | rs111960721 | 13:110626992_CT_C | 0.959151 | -0.08026 | 0.019198 | 2.90E-05 | 0.97838 | 0.941979 |
| Combined parent's age at death (z-score) | rs73919718 | 2:23724746_C_T | 0.906785 | -0.05388 | 0.012915 | 3.00E-05 | 0.994 | 0.720112 |
| Combined parent's age at death (z-score) | rs7561863 | 2:33880014_G_A | 0.232112 | 0.037354 | 0.008949 | 3.00E-05 | 0.9874 | 0.89049 |
| Combined parent's age at death (z-score) | rs10011326 | 4:3218739_A_G | 0.600825 | -0.03212 | 0.007695 | 3.00E-05 | 0.99757 | 0.015937 |
| Combined parent's age at death (z-score) | rs1441953 | 3:77307368_G_A | 0.407338 | -0.03233 | 0.007742 | 3.00E-05 | 0.98209 | 0.383893 |
| Combined parent's age at death (z-score) | rs4799730 | 18:27591327_A_G | 0.212861 | 0.03853 | 0.009236 | 3.00E-05 | 0.99617 | 0.123177 |
| Combined parent's age at death (z-score) | rs34548165 | 18:1820286_TATAA_T | 0.862652 | 0.04676 | 0.011204 | 3.00E-05 | 0.94447 | 0.018387 |
| Combined parent's age at death (z-score) | rs3743079 | 15:78791061_C_T | 0.836122 | -0.04265 | 0.01022 | 3.00E-05 | 0.98416 | 0.46579 |
| Combined parent's age at death (z-score) | rs7870432 | 9:114847102_G_C | 0.675628 | -0.03374 | 0.008089 | 3.00E-05 | 0.99501 | 0.863422 |
| Combined parent's age at death (z-score) | rs62571385 | 9:79687297_C_A | 0.932774 | -0.06262 | 0.015017 | 3.00E-05 | 0.99595 | 0.981659 |
| Combined parent's age at death (z-score) | rs11702549 | 21:22915336_A_T | 0.467982 | 0.031526 | 0.00755 | 3.00E-05 | 0.99483 | 0.710739 |
| Combined parent's age at death (z-score) | rs2100750 | 21:22920847_G_C | 0.467974 | 0.031492 | 0.00754 | 3.00E-05 | 0.99632 | 0.843805 |
| Combined parent's age at death (z-score) | rs72782128 | 16:50309171_C_T | 0.987967 | 0.151442 | 0.036283 | 3.00E-05 | 0.90048 | 0.320044 |
| Combined parent's age at death (z-score) | rs55958997 | 15:78915872_C_A | 0.647698 | 0.033413 | 0.008025 | 3.10E-05 | 0.97566 | 0.241379 |
| Combined parent's age at death (z-score) | 15:78747915_AAAAAAAG_A | 15:78747915_AAAAAAAG_A | 0.388151 | 0.032422 | 0.007786 | 3.10E-05 | 0.98004 | 0.38468 |
| Combined parent's age at death (z-score) | rs141502139 | 15:78747916_AAAAAAG_A | 0.387954 | 0.032417 | 0.007779 | 3.10E-05 | 0.98195 | 0.371433 |
| Combined parent's age at death (z-score) | rs12968346 | 18:27593479_G_A | 0.213067 | 0.038465 | 0.009233 | 3.10E-05 | 0.99613 | 0.123498 |
| Combined parent's age at death (z-score) | rs4799359 | 18:27609743_T_C | 0.212743 | 0.038437 | 0.009228 | 3.10E-05 | 0.99874 | 0.111099 |
| Combined parent's age at death (z-score) | rs66908082 | 18:26730715_TC_T | 0.0522125 | -0.09787 | 0.0235 | 3.10E-05 | 0.51209 | 0.792355 |
| Combined parent's age at death (z-score) | rs138309619 | 17:21170435_C_T | 0.982716 | 0.125915 | 0.030199 | 3.10E-05 | 0.90994 | 0.801999 |
| Combined parent's age at death (z-score) | rs62573250 | 9:79973120_C_T | 0.955752 | -0.07624 | 0.018292 | 3.10E-05 | 0.99715 | 0.372709 |
| Combined parent's age at death (z-score) | rs7870068 | 9:114846866_G_C | 0.675437 | -0.03369 | 0.008085 | 3.10E-05 | 0.99592 | 0.920967 |
| Combined parent's age at death (z-score) | rs2782931 | 9:114850190_C_T | 0.676173 | -0.03366 | 0.008075 | 3.10E-05 | 1 | 0.878935 |
| Combined parent's age at death (z-score) | rs12337699 | 9:125554327_T_A | 0.935699 | 0.075011 | 0.018013 | 3.10E-05 | 0.72361 | 0.721656 |
| Combined parent's age at death (z-score) | rs11036278 | 11:41206243_C_A | 0.842721 | -0.04317 | 0.01036 | 3.10E-05 | 0.99624 | 0.13699 |
| Combined parent's age at death (z-score) | rs201785861 | 4:3135251_TAAAA_T | 0.693171 | -0.03405 | 0.008174 | 3.10E-05 | 0.99466 | 0.159669 |
| Combined parent's age at death (z-score) | rs138984874 | 4:24174961_CAA_C | 0.0953202 | -0.05586 | 0.013419 | 3.10E-05 | 0.90664 | 0.788243 |
| Combined parent's age at death (z-score) | rs565820988 | 2:107015904_G_C | 0.985266 | -0.14013 | 0.033634 | 3.10E-05 | 0.85038 | 0.914273 |
| Combined parent's age at death (z-score) | rs7673919 | 4:162720411_G_A | 0.236722 | 0.036856 | 0.008854 | 3.10E-05 | 0.99585 | 0.541103 |
| Combined parent's age at death (z-score) | rs73615281 | 13:110624743_T_C | 0.958102 | -0.07877 | 0.018909 | 3.10E-05 | 0.98609 | 0.971576 |
| Combined parent's age at death (z-score) | rs8002682 | 13:110626731_T_C | 0.958125 | -0.0788 | 0.01891 | 3.10E-05 | 0.98636 | 0.971561 |
| Combined parent's age at death (z-score) | rs2285179 | 22:38211954_G_A | 0.449468 | 0.031541 | 0.007577 | 3.10E-05 | 0.98936 | 0.017132 |
| Combined parent's age at death (z-score) | rs3825708 | 14:75200697_G_A | 0.335018 | 0.033268 | 0.007985 | 3.10E-05 | 0.99658 | 0.269265 |
| Combined parent's age at death (z-score) | rs10220422 | 14:75222337_C_G | 0.33383 | 0.03333 | 0.007999 | 3.10E-05 | 0.99668 | 0.368791 |
| Combined parent's age at death (z-score) | rs8003660 | 14:75227098_T_C | 0.333732 | 0.033295 | 0.007992 | 3.10E-05 | 1 | 0.544641 |
| Combined parent's age at death (z-score) | rs7146395 | 14:75228647_A_C | 0.329139 | 0.033401 | 0.008021 | 3.10E-05 | 0.99873 | 0.516297 |
| Combined parent's age at death (z-score) | rs1506925 | 12:61585448_G_C | 0.112469 | 0.049696 | 0.011922 | 3.10E-05 | 0.99237 | 0.707115 |
| Combined parent's age at death (z-score) | rs4024187 | 12:61603140_C_CCTTGT | 0.112467 | 0.049653 | 0.011919 | 3.10E-05 | 0.9932 | 0.707066 |
| Combined parent's age at death (z-score) | rs78562044 | 6:72125948_A_T | 0.955788 | 0.077324 | 0.018546 | 3.10E-05 | 0.97969 | 0.633257 |
| Combined parent's age at death (z-score) | rs112511417 | 3:177011681_C_T | 0.958184 | -0.08039 | 0.019294 | 3.10E-05 | 0.94456 | 0.35182 |
| Combined parent's age at death (z-score) | rs75173836 | 6:72140954_A_G | 0.956419 | 0.077624 | 0.018672 | 3.20E-05 | 0.97556 | 0.755388 |
| Combined parent's age at death (z-score) | rs35721825 | 5:152507693_T_C | 0.780717 | -0.03808 | 0.009164 | 3.20E-05 | 0.97928 | 0.2266 |
| Combined parent's age at death (z-score) | rs9811345 | 3:70590738_G_A | 0.761621 | 0.036787 | 0.00885 | 3.20E-05 | 0.99265 | 0.623017 |
| Combined parent's age at death (z-score) | rs10933845 | 3:106091140_G_A | 0.071892 | -0.06061 | 0.014571 | 3.20E-05 | 0.99244 | 0.292294 |
| Combined parent's age at death (z-score) | rs16935437 | 11:41226389_G_A | 0.838409 | -0.04272 | 0.010277 | 3.20E-05 | 0.98947 | 0.453503 |
| Combined parent's age at death (z-score) | rs2919039 | 11:96589219_G_A | 0.426582 | 0.032144 | 0.007726 | 3.20E-05 | 0.95828 | 0.222439 |
| Combined parent's age at death (z-score) | rs7938558 | 11:21149632_G_T | 0.805326 | -0.03979 | 0.009566 | 3.20E-05 | 0.99252 | 0.115305 |
| Combined parent's age at death (z-score) | rs56045627 | 18:27592146_A_AGTAT | 0.214459 | 0.038448 | 0.009243 | 3.20E-05 | 0.98948 | 0.125314 |
| Combined parent's age at death (z-score) | rs33967016 | 18:27600208_G_GA | 0.212774 | 0.03835 | 0.009225 | 3.20E-05 | 0.99897 | 0.121024 |
| Combined parent's age at death (z-score) | rs8086825 | 18:27610573_A_G | 0.212505 | 0.038455 | 0.009247 | 3.20E-05 | 0.99455 | 0.133693 |
| Combined parent's age at death (z-score) | rs4799747 | 18:27620381_G_A | 0.212948 | 0.038435 | 0.009244 | 3.20E-05 | 0.99421 | 0.102146 |
| Combined parent's age at death (z-score) | rs16969906 | 15:78793109_T_C | 0.835473 | -0.04242 | 0.010202 | 3.20E-05 | 0.9848 | 0.506839 |
| Combined parent's age at death (z-score) | rs11637193 | 15:78794235_C_T | 0.835473 | -0.04242 | 0.010202 | 3.20E-05 | 0.9848 | 0.513594 |
| Combined parent's age at death (z-score) | rs7025001 | 9:14821333_T_C | 0.759826 | -0.03669 | 0.008822 | 3.20E-05 | 0.99724 | 0.629695 |
| Combined parent's age at death (z-score) | rs74746968 | 9:137655046_T_G | 0.980509 | 0.116559 | 0.02805 | 3.20E-05 | 0.93611 | 0.411159 |
| Combined parent's age at death (z-score) | rs28541694 | 8:27462008_C_G | 0.733471 | -0.03536 | 0.008503 | 3.20E-05 | 0.99923 | 0.02355 |
| Combined parent's age at death (z-score) | rs73231005 | 8:27470778_C_G | 0.719618 | -0.0349 | 0.008393 | 3.20E-05 | 0.98423 | 2.29E-05 |
| Combined parent's age at death (z-score) | rs59788584 | 17:9719724_T_C | 0.557997 | 0.03177 | 0.007633 | 3.20E-05 | 0.97597 | 0.704061 |
| Combined parent's age at death (z-score) | rs4024186 | 12:61603220_C_A | 0.112417 | 0.049541 | 0.01192 | 3.20E-05 | 0.99328 | 0.706985 |
| Combined parent's age at death (z-score) | rs113982913 | 22:50885244_TTC_T | 0.798324 | 0.040442 | 0.009718 | 3.20E-05 | 0.94623 | 0.725905 |
| Combined parent's age at death (z-score) | rs7673807 | 4:162720528_C_T | 0.236539 | 0.036799 | 0.008854 | 3.20E-05 | 0.99645 | 0.572756 |
| Combined parent's age at death (z-score) | rs13143298 | 4:157524250_A_G | 0.322911 | -0.03487 | 0.008379 | 3.20E-05 | 0.92011 | 0.68259 |
| Combined parent's age at death (z-score) | rs13143462 | 4:157524251_C_T | 0.322895 | -0.03487 | 0.008379 | 3.20E-05 | 0.92011 | 0.672946 |
| Combined parent's age at death (z-score) | rs2342141 | 4:157548539_C_A | 0.747567 | 0.036064 | 0.008673 | 3.20E-05 | 0.99407 | 0.8786 |
| Combined parent's age at death (z-score) | rs72698429 | 4:157551164_C_T | 0.747457 | 0.036054 | 0.008676 | 3.20E-05 | 0.9932 | 0.872626 |
| Combined parent's age at death (z-score) | rs1521726 | 4:157552408_T_A | 0.747528 | 0.03604 | 0.008673 | 3.20E-05 | 0.9939 | 0.884637 |
| Combined parent's age at death (z-score) | rs6543735 | 2:33896721_G_A | 0.232337 | 0.037208 | 0.008946 | 3.20E-05 | 0.98763 | 0.865032 |
| Combined parent's age at death (z-score) | rs524406 | 1:51744478_C_T | 0.961379 | -0.08103 | 0.019491 | 3.20E-05 | 1 | 0.458955 |
| Combined parent's age at death (z-score) | rs112646314 | 13:110623028_C_T | 0.958216 | -0.07864 | 0.01892 | 3.20E-05 | 0.98724 | 0.943079 |
| Combined parent's age at death (z-score) | rs13408535 | 2:152966710_A_C | 0.983137 | -0.12084 | 0.029038 | 3.20E-05 | 1 | 0.082186 |
| Combined parent's age at death (z-score) | rs1849068 | 2:195967027_A_G | 0.44492 | -0.03507 | 0.00844 | 3.30E-05 | 0.79725 | 0.487315 |
| Combined parent's age at death (z-score) | rs34137238 | 4:24165930_AG_A | 0.891507 | 0.050246 | 0.012107 | 3.30E-05 | 0.99101 | 0.164486 |
| Combined parent's age at death (z-score) | rs1267025 | 4:24167431_C_T | 0.891317 | 0.050384 | 0.012135 | 3.30E-05 | 0.98558 | 0.169684 |
| Combined parent's age at death (z-score) | rs74906516 | 4:25215520_T_C | 0.962502 | 0.083547 | 0.020119 | 3.30E-05 | 0.96596 | 0.664737 |
| Combined parent's age at death (z-score) | rs2285087 | 4:3088211_A_G | 0.638102 | -0.03271 | 0.007874 | 3.30E-05 | 0.98985 | 0.086836 |
| Combined parent's age at death (z-score) | rs362279 | 4:3260072_G_A | 0.613398 | -0.03228 | 0.007776 | 3.30E-05 | 0.99021 | 0.027791 |
| Combined parent's age at death (z-score) | rs7978225 | 12:61592841_T_C | 0.112421 | 0.04946 | 0.011919 | 3.30E-05 | 0.99344 | 0.706985 |
| Combined parent's age at death (z-score) | rs10784181 | 12:61593776_A_C | 0.112421 | 0.049459 | 0.011919 | 3.30E-05 | 0.99344 | 0.706985 |
| Combined parent's age at death (z-score) | rs6492234 | 13:110618465_C_G | 0.958199 | -0.07851 | 0.018921 | 3.30E-05 | 0.98607 | 0.886501 |
| Combined parent's age at death (z-score) | rs67787562 | 22:29618395_CT_C | 0.238244 | -0.04332 | 0.010442 | 3.30E-05 | 0.71267 | 0.911834 |
| Combined parent's age at death (z-score) | rs6715333 | 2:33887146_T_C | 0.232071 | 0.037142 | 0.008939 | 3.30E-05 | 0.98944 | 0.858561 |
| Combined parent's age at death (z-score) | rs57475156 | 2:15051742_G_A | 0.98853 | -0.14624 | 0.035234 | 3.30E-05 | 1 | 0.526502 |
| Combined parent's age at death (z-score) | rs115961910 | 3:89227100_A_G | 0.978327 | -0.10683 | 0.025752 | 3.30E-05 | 1 | 0.44858 |
| Combined parent's age at death (z-score) | rs73171597 | 3:171064963_G_T | 0.987144 | 0.157414 | 0.037929 | 3.30E-05 | 0.7789 | 0.200498 |
| Combined parent's age at death (z-score) | rs80165059 | 6:72120015_C_G | 0.956042 | 0.077181 | 0.018599 | 3.30E-05 | 0.98042 | 0.583324 |
| Combined parent's age at death (z-score) | rs76268794 | 6:72122942_A_T | 0.955774 | 0.076953 | 0.018526 | 3.30E-05 | 0.98147 | 0.633301 |
| Combined parent's age at death (z-score) | rs8067349 | 17:9721208_A_G | 0.552852 | 0.031288 | 0.007541 | 3.30E-05 | 1 | 0.934945 |
| Combined parent's age at death (z-score) | rs66958558 | 17:12645033_TAA_T | 0.923248 | 0.072766 | 0.017514 | 3.30E-05 | 0.64879 | 0.760659 |
| Combined parent's age at death (z-score) | rs67254666 | 17:18171119_ACACTTGGCTGAGGTCCATTCCAAT_A | 0.585366 | 0.032408 | 0.007804 | 3.30E-05 | 0.95449 | 0.971512 |
| Combined parent's age at death (z-score) | rs72876784 | 11:18570791_G_A | 0.88255 | 0.048588 | 0.011698 | 3.30E-05 | 0.99607 | 0.116115 |
| Combined parent's age at death (z-score) | rs143522174 | 11:18600636_G_A | 0.882795 | 0.048603 | 0.011711 | 3.30E-05 | 0.99594 | 0.128795 |
| Combined parent's age at death (z-score) | rs7928755 | 11:130581854_C_T | 0.757639 | 0.036843 | 0.008878 | 3.30E-05 | 0.96481 | 0.000402 |
| Combined parent's age at death (z-score) | rs7281895 | 21:22926491_A_G | 0.467097 | 0.031444 | 0.007571 | 3.30E-05 | 0.98839 | 0.884781 |
| Combined parent's age at death (z-score) | rs1460091 | 21:22927241_C_T | 0.467691 | 0.031402 | 0.007565 | 3.30E-05 | 0.9896 | 0.921518 |
| Combined parent's age at death (z-score) | rs11326453 | 8:134176951_AT_A | 0.606375 | 0.032177 | 0.007752 | 3.30E-05 | 0.9907 | 0.23135 |
| Combined parent's age at death (z-score) | rs2739121 | 8:134177852_T_C | 0.606862 | 0.032134 | 0.00774 | 3.30E-05 | 0.9935 | 0.235863 |
| Combined parent's age at death (z-score) | rs12604756 | 18:27598483_C_G | 0.2135 | 0.038279 | 0.009224 | 3.30E-05 | 0.99715 | 0.124039 |
| Combined parent's age at death (z-score) | rs8085609 | 18:27610765_G_C | 0.21297 | 0.03829 | 0.009223 | 3.30E-05 | 0.99891 | 0.115255 |
| Combined parent's age at death (z-score) | rs8085862 | 18:27620646_A_C | 0.216899 | 0.03823 | 0.009213 | 3.30E-05 | 0.98822 | 0.139949 |
| Combined parent's age at death (z-score) | rs13289400 | 9:14825142_G_A | 0.760261 | -0.03681 | 0.008864 | 3.30E-05 | 0.9905 | 0.579522 |
| Combined parent's age at death (z-score) | rs7856534 | 9:114847175_T_C | 0.67558 | -0.03356 | 0.008086 | 3.30E-05 | 0.9958 | 0.910454 |
| Combined parent's age at death (z-score) | rs111718413 | 9:114851068_A_AAAAC | 0.678582 | -0.03367 | 0.008128 | 3.40E-05 | 0.98941 | 1 |
| Combined parent's age at death (z-score) | rs2779523 | 9:101334945_G_T | 0.618611 | 0.03204 | 0.007736 | 3.40E-05 | 1 | 0.732285 |
| Combined parent's age at death (z-score) | rs11188983 | 10:98762853_G_A | 0.929198 | -0.06096 | 0.014718 | 3.40E-05 | 0.99448 | 0.877193 |
| Combined parent's age at death (z-score) | rs985294 | 11:41201969_A_G | 0.845355 | -0.04332 | 0.010457 | 3.40E-05 | 0.99125 | 0.136814 |
| Combined parent's age at death (z-score) | rs143034675 | 11:18559926_C_G | 0.882462 | 0.048451 | 0.011691 | 3.40E-05 | 0.99619 | 0.129682 |
| Combined parent's age at death (z-score) | rs8086089 | 18:27594391_A_G | 0.212819 | 0.038219 | 0.009225 | 3.40E-05 | 0.99892 | 0.119116 |
| Combined parent's age at death (z-score) | rs4564654 | 18:27602954_A_G | 0.21295 | 0.038196 | 0.009221 | 3.40E-05 | 0.99936 | 0.117233 |
| Combined parent's age at death (z-score) | rs4334372 | 18:27608336_T_C | 0.212788 | 0.038244 | 0.009222 | 3.40E-05 | 0.99918 | 0.115064 |
| Combined parent's age at death (z-score) | rs8085168 | 18:27610423_G_A | 0.212905 | 0.038214 | 0.009225 | 3.40E-05 | 0.99866 | 0.119187 |
| Combined parent's age at death (z-score) | rs4359526 | 18:27618005_A_G | 0.213023 | 0.03834 | 0.009243 | 3.40E-05 | 0.99415 | 0.104033 |
| Combined parent's age at death (z-score) | rs9331905 | 8:27464244_T_C | 0.732889 | -0.03527 | 0.008504 | 3.40E-05 | 0.99892 | 0.048328 |
| Combined parent's age at death (z-score) | rs11124329 | 2:33888502_G_A | 0.231695 | 0.037083 | 0.008948 | 3.40E-05 | 0.98926 | 0.852026 |
| Combined parent's age at death (z-score) | rs7665529 | 4:162699899_T_C | 0.235741 | 0.036668 | 0.008851 | 3.40E-05 | 1 | 0.886045 |
| Combined parent's age at death (z-score) | rs4823082 | 22:30600105_G_C | 0.978088 | -0.10701 | 0.025819 | 3.40E-05 | 0.99751 | 0.42157 |
| Combined parent's age at death (z-score) | rs5753046 | 22:30602246_C_A | 0.978105 | -0.10753 | 0.025938 | 3.40E-05 | 0.98875 | 0.421114 |
| Combined parent's age at death (z-score) | rs9668675 | 12:61601038_A_C | 0.112436 | 0.049405 | 0.01192 | 3.40E-05 | 0.99332 | 0.707034 |
| Combined parent's age at death (z-score) | rs10735889 | 12:61612829_G_A | 0.112173 | 0.049498 | 0.011943 | 3.40E-05 | 0.99137 | 0.70635 |
| Combined parent's age at death (z-score) | rs72638547 | 1:7001078_C_T | 0.762786 | -0.03741 | 0.009032 | 3.40E-05 | 0.95847 | 0.929949 |
| Combined parent's age at death (z-score) | rs574141985 | 14:62594501_TTTTA_T | 0.984483 | -0.13476 | 0.032533 | 3.40E-05 | 0.87208 | 0.039673 |
| Combined parent's age at death (z-score) | rs374071816 | 6:161103805_T_A | 0.904096 | 0.055646 | 0.013432 | 3.40E-05 | 0.90738 | 0.933712 |
| Combined parent's age at death (z-score) | rs115762864 | 19:38397285_G_A | 0.987206 | 0.21139 | 0.051034 | 3.40E-05 | 0.43347 | 0.729726 |
| Combined parent's age at death (z-score) | rs146043658 | 5:112166989_A_G | 0.9889 | -0.15491 | 0.037396 | 3.40E-05 | 0.9188 | 0.004378 |
| Combined parent's age at death (z-score) | rs187608901 | 13:19203578_T_C | 0.980787 | 0.120694 | 0.029181 | 3.50E-05 | 0.87744 | 0.456962 |
| Combined parent's age at death (z-score) | rs76237238 | 6:72109907_G_A | 0.955772 | 0.076602 | 0.018521 | 3.50E-05 | 0.98213 | 0.585585 |
| Combined parent's age at death (z-score) | rs145005923 | 6:72111148_AT_A | 0.955772 | 0.076601 | 0.018521 | 3.50E-05 | 0.98212 | 0.585585 |
| Combined parent's age at death (z-score) | rs148432834 | 6:72113489_GA_G | 0.955772 | 0.076602 | 0.018521 | 3.50E-05 | 0.98213 | 0.585585 |
| Combined parent's age at death (z-score) | rs77668034 | 6:72115305_C_T | 0.955772 | 0.076602 | 0.018521 | 3.50E-05 | 0.98213 | 0.585585 |
| Combined parent's age at death (z-score) | rs75889129 | 6:72117483_C_G | 0.955788 | 0.076702 | 0.018527 | 3.50E-05 | 0.98195 | 0.585531 |
| Combined parent's age at death (z-score) | rs76297264 | 6:72118369_G_A | 0.955772 | 0.076602 | 0.018521 | 3.50E-05 | 0.98213 | 0.585585 |
| Combined parent's age at death (z-score) | rs77994134 | 6:72148366_A_C | 0.956331 | 0.077301 | 0.01869 | 3.50E-05 | 0.97197 | 0.703915 |
| Combined parent's age at death (z-score) | rs113702148 | 6:72150044_T_C | 0.956334 | 0.077347 | 0.018691 | 3.50E-05 | 0.97191 | 0.755718 |
| Combined parent's age at death (z-score) | rs112981596 | 6:72150768_T_G | 0.956331 | 0.077301 | 0.01869 | 3.50E-05 | 0.97197 | 0.703915 |
| Combined parent's age at death (z-score) | rs13302958 | 3:195307794_T_C | 0.627196 | 0.032077 | 0.00775 | 3.50E-05 | 0.99737 | 0.204574 |
| Combined parent's age at death (z-score) | rs62256321 | 3:70590619_A_G | 0.759497 | 0.036534 | 0.008835 | 3.50E-05 | 0.98949 | 0.647649 |
| Combined parent's age at death (z-score) | rs34109053 | 8:27470597_G_A | 0.719947 | -0.03471 | 0.008383 | 3.50E-05 | 0.98715 | 9.42E-06 |
| Combined parent's age at death (z-score) | rs11655549 | 17:9719158_C_T | 0.554797 | 0.03148 | 0.007603 | 3.50E-05 | 0.98319 | 0.810913 |
| Combined parent's age at death (z-score) | rs111141414 | 17:9719762_CGT_C | 0.554212 | 0.031491 | 0.007605 | 3.50E-05 | 0.98266 | 0.902495 |
| Combined parent's age at death (z-score) | rs4791875 | 17:9720546_C_T | 0.553856 | 0.031436 | 0.007591 | 3.50E-05 | 0.987 | 0.902514 |
| Combined parent's age at death (z-score) | rs8071852 | 17:9721479_T_A | 0.553596 | 0.031531 | 0.00762 | 3.50E-05 | 0.98021 | 0.953494 |
| Combined parent's age at death (z-score) | rs850794 | 15:23935812_G_T | 0.0923177 | -0.05636 | 0.013623 | 3.50E-05 | 0.91521 | 0.145141 |
| Combined parent's age at death (z-score) | rs62573228 | 9:79942685_G_C | 0.947661 | -0.06992 | 0.016891 | 3.50E-05 | 0.99606 | 0.363312 |
| Combined parent's age at death (z-score) | rs6508635 | 18:27594028_A_C | 0.21294 | 0.038177 | 0.009221 | 3.50E-05 | 0.99933 | 0.119239 |
| Combined parent's age at death (z-score) | rs9959426 | 18:27597756_T_C | 0.212952 | 0.038184 | 0.009221 | 3.50E-05 | 0.99936 | 0.117233 |
| Combined parent's age at death (z-score) | rs4461154 | 18:27599024_T_G | 0.21295 | 0.038185 | 0.009221 | 3.50E-05 | 0.99937 | 0.117233 |
| Combined parent's age at death (z-score) | rs4630626 | 18:27599269_C_T | 0.212949 | 0.038184 | 0.009221 | 3.50E-05 | 0.99936 | 0.117233 |
| Combined parent's age at death (z-score) | rs4577173 | 18:27599452_A_G | 0.21295 | 0.038185 | 0.009221 | 3.50E-05 | 0.99937 | 0.117233 |
| Combined parent's age at death (z-score) | rs9952615 | 18:27601575_A_T | 0.21295 | 0.038185 | 0.009221 | 3.50E-05 | 0.99937 | 0.117233 |
| Combined parent's age at death (z-score) | rs202172833 | 18:27605814_G_A | 0.21295 | 0.038185 | 0.009221 | 3.50E-05 | 0.99937 | 0.117233 |
| Combined parent's age at death (z-score) | rs8090651 | 18:27610660_T_C | 0.212534 | 0.038205 | 0.009236 | 3.50E-05 | 0.99758 | 0.125012 |
| Combined parent's age at death (z-score) | rs12960372 | 18:27613378_T_C | 0.213448 | 0.038134 | 0.009219 | 3.50E-05 | 0.99796 | 0.09723 |
| Combined parent's age at death (z-score) | rs4632198 | 18:27615372_G_A | 0.212228 | 0.038246 | 0.009238 | 3.50E-05 | 0.99744 | 0.140055 |
| Combined parent's age at death (z-score) | rs8097494 | 18:27621461_G_A | 0.213035 | 0.038271 | 0.00925 | 3.50E-05 | 0.9927 | 0.09528 |
| Combined parent's age at death (z-score) | rs12953529 | 18:50968973_A_G | 0.549913 | 0.031477 | 0.007606 | 3.50E-05 | 0.99305 | 0.762202 |
| Combined parent's age at death (z-score) | rs10219146 | 11:18590005_C_T | 0.118611 | -0.04809 | 0.011629 | 3.50E-05 | 1 | 0.044412 |
| Combined parent's age at death (z-score) | rs3019729 | 11:96590356_A_G | 0.425264 | 0.031992 | 0.007727 | 3.50E-05 | 0.95921 | 0.222044 |
| Combined parent's age at death (z-score) | rs1374616 | 11:30584613_C_A | 0.847823 | -0.04329 | 0.010464 | 3.50E-05 | 0.9957 | 0.991154 |
| Combined parent's age at death (z-score) | rs17300755 | 10:6507212_A_G | 0.979238 | -0.10867 | 0.026279 | 3.50E-05 | 1 | 0.04102 |
| Combined parent's age at death (z-score) | rs1839156 | 2:33887560_G_A | 0.232266 | 0.037012 | 0.008939 | 3.50E-05 | 0.98895 | 0.884156 |
| Combined parent's age at death (z-score) | rs6543733 | 2:33896461_T_C | 0.232246 | 0.037016 | 0.008947 | 3.50E-05 | 0.98777 | 0.833257 |
| Combined parent's age at death (z-score) | rs35997970 | 4:3255741_C_T | 0.627184 | -0.03299 | 0.007972 | 3.50E-05 | 0.95335 | 0.085448 |
| Combined parent's age at death (z-score) | rs6746331 | 2:234909799_G_A | 0.799906 | -0.039 | 0.00943 | 3.50E-05 | 0.99155 | 0.858335 |
| Combined parent's age at death (z-score) | rs78045855 | 13:110537722_C_G | 0.974055 | -0.09876 | 0.023856 | 3.50E-05 | 0.98517 | 0.42417 |
| Combined parent's age at death (z-score) | 22:38309883_C_CTT | 22:38309883_C_CTT | 0.614301 | -0.03262 | 0.00788 | 3.50E-05 | 0.9584 | 0.055144 |
| Combined parent's age at death (z-score) | rs556545813 | 22:38223861_C_CA | 0.495442 | 0.032284 | 0.007818 | 3.60E-05 | 0.92334 | 0.07663 |
| Combined parent's age at death (z-score) | rs112439573 | 13:110616860_GA_G | 0.95853 | -0.07834 | 0.018972 | 3.60E-05 | 0.98838 | 0.857405 |
| Combined parent's age at death (z-score) | rs78470266 | 13:110619623_G_A | 0.958521 | -0.0784 | 0.018972 | 3.60E-05 | 0.98822 | 0.85746 |
| Combined parent's age at death (z-score) | rs12585031 | 13:110626112_T_G | 0.957929 | -0.07809 | 0.018892 | 3.60E-05 | 0.98396 | 0.915189 |
| Combined parent's age at death (z-score) | rs2106430 | 22:30597135_C_T | 0.978154 | -0.10704 | 0.02591 | 3.60E-05 | 0.99562 | 0.501326 |
| Combined parent's age at death (z-score) | rs5012284 | 4:58242733_T_A | 0.948869 | 0.094569 | 0.022906 | 3.60E-05 | 0.54772 | 0.744081 |
| Combined parent's age at death (z-score) | rs190922696 | 2:17541497_G_A | 0.985274 | -0.13486 | 0.032639 | 3.60E-05 | 0.9124 | 0.036233 |
| Combined parent's age at death (z-score) | rs3866825 | 4:111784832_G_A | 0.226265 | -0.03717 | 0.008994 | 3.60E-05 | 0.99816 | 0.44088 |
| Combined parent's age at death (z-score) | rs7638039 | 3:70588939_C_T | 0.753726 | 0.036334 | 0.008794 | 3.60E-05 | 0.9866 | 0.143886 |
| Combined parent's age at death (z-score) | rs11667828 | 19:19768474_T_G | 0.745237 | 0.035685 | 0.008638 | 3.60E-05 | 0.9934 | 0.819942 |
| Combined parent's age at death (z-score) | rs12983292 | 19:19787767_G_A | 0.744887 | 0.035667 | 0.008636 | 3.60E-05 | 0.99296 | 0.773304 |
| Combined parent's age at death (z-score) | rs375046492 | 10:56613126_CAT_C | 0.887484 | 0.049516 | 0.011986 | 3.60E-05 | 0.98793 | 0.512543 |
| Combined parent's age at death (z-score) | rs34362932 | 10:71967822_TG_T | 0.940152 | 0.067422 | 0.016323 | 3.60E-05 | 0.9351 | 0.549263 |
| Combined parent's age at death (z-score) | rs11817476 | 10:71967913_T_C | 0.940152 | 0.067422 | 0.016323 | 3.60E-05 | 0.9351 | 0.549263 |
| Combined parent's age at death (z-score) | rs11651190 | 17:9719619_A_T | 0.554338 | 0.031385 | 0.007601 | 3.60E-05 | 0.98385 | 0.838203 |
| Combined parent's age at death (z-score) | rs16958408 | 17:9422618_T_G | 0.397074 | -0.03257 | 0.007879 | 3.60E-05 | 0.94275 | 0.005599 |
| Combined parent's age at death (z-score) | rs10768669 | 11:41180155_A_G | 0.84349 | -0.04293 | 0.010385 | 3.60E-05 | 0.99496 | 0.197651 |
| Combined parent's age at death (z-score) | rs10500891 | 11:21131784_C_G | 0.821664 | -0.04115 | 0.009955 | 3.60E-05 | 0.98054 | 0.191346 |
| Combined parent's age at death (z-score) | rs62573225 | 9:79928172_G_A | 0.946884 | -0.06928 | 0.016762 | 3.60E-05 | 0.99683 | 0.386492 |
| Combined parent's age at death (z-score) | rs2782946 | 9:114847318_G_A | 0.672558 | -0.0332 | 0.00804 | 3.60E-05 | 1 | 0.128535 |
| Combined parent's age at death (z-score) | rs7233476 | 18:27607298_A_G | 0.212615 | 0.038173 | 0.009232 | 3.60E-05 | 0.9976 | 0.127198 |
| Combined parent's age at death (z-score) | rs8094255 | 18:27608881_C_T | 0.212948 | 0.038078 | 0.009221 | 3.60E-05 | 0.99924 | 0.121319 |
| Combined parent's age at death (z-score) | rs9957896 | 18:27609462_T_C | 0.212602 | 0.038181 | 0.009234 | 3.60E-05 | 0.99745 | 0.122956 |
| Combined parent's age at death (z-score) | rs6508637 | 18:27612900_C_T | 0.212972 | 0.038094 | 0.009223 | 3.60E-05 | 0.99866 | 0.111406 |
| Combined parent's age at death (z-score) | rs8085663 | 18:27620874_C_G | 0.213044 | 0.038181 | 0.009248 | 3.60E-05 | 0.99323 | 0.104057 |
| Combined parent's age at death (z-score) | rs8098435 | 18:27621045_A_G | 0.21304 | 0.038237 | 0.00925 | 3.60E-05 | 0.99273 | 0.098719 |
| Combined parent's age at death (z-score) | rs35220224 | 18:27598275_TA_T | 0.212976 | 0.038053 | 0.00922 | 3.70E-05 | 0.99931 | 0.121342 |
| Combined parent's age at death (z-score) | rs9960022 | 18:27598527_A_G | 0.21266 | 0.038093 | 0.009227 | 3.70E-05 | 0.99917 | 0.136047 |
| Combined parent's age at death (z-score) | rs182261127 | 18:27606324_T_C | 0.212976 | 0.038055 | 0.00922 | 3.70E-05 | 0.99931 | 0.121342 |
| Combined parent's age at death (z-score) | rs4358017 | 18:27606877_G_T | 0.212976 | 0.038055 | 0.00922 | 3.70E-05 | 0.99931 | 0.121342 |
| Combined parent's age at death (z-score) | rs12960258 | 18:27607816_C_G | 0.212976 | 0.038055 | 0.00922 | 3.70E-05 | 0.99931 | 0.121342 |
| Combined parent's age at death (z-score) | rs4410158 | 18:27613813_G_C | 0.21291 | 0.038051 | 0.009225 | 3.70E-05 | 0.99851 | 0.117184 |
| Combined parent's age at death (z-score) | rs4318283 | 18:27622162_C_T | 0.212909 | 0.038208 | 0.009265 | 3.70E-05 | 0.98969 | 0.103963 |
| Combined parent's age at death (z-score) | rs58766815 | 15:78796221_C_T | 0.835724 | -0.04216 | 0.010215 | 3.70E-05 | 0.98382 | 0.526793 |
| Combined parent's age at death (z-score) | rs541910703 | 15:78721238_G_GT | 0.8466 | -0.04415 | 0.010704 | 3.70E-05 | 0.94429 | 0.616311 |
| Combined parent's age at death (z-score) | rs7193971 | 16:25895486_A_G | 0.938325 | 0.06527 | 0.015812 | 3.70E-05 | 0.97661 | 0.302956 |
| Combined parent's age at death (z-score) | rs13288945 | 9:114845493_C_G | 0.676097 | -0.03339 | 0.00809 | 3.70E-05 | 0.99568 | 0.899899 |
| Combined parent's age at death (z-score) | rs144676527 | 9:6301951_C_T | 0.988928 | 0.15465 | 0.037466 | 3.70E-05 | 0.91578 | 0.115838 |
| Combined parent's age at death (z-score) | rs62571386 | 9:79687706_T_C | 0.93269 | -0.06182 | 0.014982 | 3.70E-05 | 1 | 0.945081 |
| Combined parent's age at death (z-score) | rs11655535 | 17:9719103_C_A | 0.555844 | 0.031429 | 0.007614 | 3.70E-05 | 0.98081 | 0.770371 |
| Combined parent's age at death (z-score) | rs74660900 | 17:9720080_CTG_C | 0.5592 | 0.031554 | 0.007653 | 3.70E-05 | 0.97438 | 0.869981 |
| Combined parent's age at death (z-score) | rs75096320 | 11:9026021_C_A | 0.921703 | -0.05784 | 0.014012 | 3.70E-05 | 0.99687 | 0.506991 |
| Combined parent's age at death (z-score) | rs76301237 | 11:106622105_C_T | 0.977542 | 0.107075 | 0.025946 | 3.70E-05 | 0.95637 | 0.477035 |
| Combined parent's age at death (z-score) | rs6821330 | 4:162701376_A_G | 0.234505 | 0.036604 | 0.008873 | 3.70E-05 | 0.99752 | 0.609214 |
| Combined parent's age at death (z-score) | rs6847250 | 4:162701397_T_A | 0.234505 | 0.036604 | 0.008873 | 3.70E-05 | 0.99752 | 0.609214 |
| Combined parent's age at death (z-score) | rs1975227 | 4:162706059_C_A | 0.252677 | 0.036048 | 0.008738 | 3.70E-05 | 0.97704 | 0.808037 |
| Combined parent's age at death (z-score) | rs11933304 | 4:162708879_C_T | 0.234788 | 0.036557 | 0.00886 | 3.70E-05 | 0.99925 | 0.533654 |
| Combined parent's age at death (z-score) | rs13152153 | 4:162723341_C_T | 0.236251 | 0.036524 | 0.008852 | 3.70E-05 | 0.99743 | 0.627769 |
| Combined parent's age at death (z-score) | rs34991484 | 4:3260768_CA_C | 0.604318 | -0.03224 | 0.007815 | 3.70E-05 | 0.97344 | 0.06269 |
| Combined parent's age at death (z-score) | rs3095073 | 4:3263138_G_A | 0.611701 | -0.03195 | 0.007743 | 3.70E-05 | 0.99861 | 0.031769 |
| Combined parent's age at death (z-score) | rs77848619 | 1:85791050_G_A | 0.983711 | -0.15556 | 0.03773 | 3.70E-05 | 0.6128 | 0.854966 |
| Combined parent's age at death (z-score) | rs1839154 | 2:33889032_G_C | 0.23185 | 0.036883 | 0.008946 | 3.70E-05 | 0.98922 | 0.833079 |
| Combined parent's age at death (z-score) | rs28635562 | 19:19769690_C_T | 0.744996 | 0.035549 | 0.008618 | 3.70E-05 | 0.99728 | 0.82004 |
| Combined parent's age at death (z-score) | rs7246984 | 19:33568670_C_T | 0.915341 | 0.05589 | 0.013554 | 3.70E-05 | 0.98853 | 0.192926 |
| Combined parent's age at death (z-score) | rs61160702 | 6:12405482_A_G | 0.882542 | -0.04851 | 0.01176 | 3.70E-05 | 1 | 0.683345 |
| Combined parent's age at death (z-score) | rs4928025 | 3:54296186_C_G | 0.335922 | 0.033588 | 0.008139 | 3.70E-05 | 0.95038 | 0.984517 |
| Combined parent's age at death (z-score) | rs8108329 | 19:33583919_A_G | 0.915898 | 0.055962 | 0.013591 | 3.80E-05 | 0.99054 | 0.25405 |
| Combined parent's age at death (z-score) | rs3826907 | 19:33599246_C_T | 0.9066 | 0.053108 | 0.012893 | 3.80E-05 | 0.99803 | 0.114438 |
| Combined parent's age at death (z-score) | rs140128821 | 19:19795456_TAACTC_T | 0.747444 | 0.036443 | 0.008847 | 3.80E-05 | 0.95426 | 0.748618 |
| Combined parent's age at death (z-score) | rs150441125 | 11:96589737_AAAAT_A | 0.425408 | 0.031845 | 0.007727 | 3.80E-05 | 0.95984 | 0.288337 |
| Combined parent's age at death (z-score) | rs6508636 | 18:27611774_C_A | 0.212902 | 0.038017 | 0.009226 | 3.80E-05 | 0.99849 | 0.109413 |
| Combined parent's age at death (z-score) | rs11279529 | 18:27620424_ATCACTTGAGC_A | 0.212703 | 0.038137 | 0.009256 | 3.80E-05 | 0.99305 | 0.094951 |
| Combined parent's age at death (z-score) | rs566415991 | 8:68080259_C_T | 0.983037 | -0.13352 | 0.032431 | 3.80E-05 | 0.79539 | 0.721074 |
| Combined parent's age at death (z-score) | rs850819 | 15:23939177_G_A | 0.0925742 | -0.054 | 0.013118 | 3.80E-05 | 0.98493 | 0.260136 |
| Combined parent's age at death (z-score) | rs5842938 | 21:22915334_TA_T | 0.467393 | 0.031078 | 0.007549 | 3.80E-05 | 0.99496 | 0.75431 |
| Combined parent's age at death (z-score) | rs2564898 | 9:114850947_T_C | 0.67554 | -0.03331 | 0.008091 | 3.80E-05 | 0.99465 | 0.947244 |
| Combined parent's age at death (z-score) | rs3755883 | 4:3223250_A_G | 0.67684 | -0.03321 | 0.008056 | 3.80E-05 | 0.99718 | 0.021822 |
| Combined parent's age at death (z-score) | rs6848813 | 4:24165287_G_A | 0.891579 | 0.049817 | 0.012112 | 3.90E-05 | 0.99067 | 0.150883 |
| Combined parent's age at death (z-score) | rs112155887 | 2:33889551_C_CTAAA | 0.231747 | 0.036785 | 0.008946 | 3.90E-05 | 0.98976 | 0.852042 |
| Combined parent's age at death (z-score) | rs73919713 | 2:23722870_C_T | 0.906459 | -0.05294 | 0.012873 | 3.90E-05 | 0.99701 | 0.77236 |
| Combined parent's age at death (z-score) | rs186242844 | 2:14337409_T_C | 0.987801 | -0.14907 | 0.03624 | 3.90E-05 | 0.87997 | 0.323937 |
| Combined parent's age at death (z-score) | rs1434611 | 4:162718310_C_G | 0.235306 | 0.036428 | 0.008861 | 3.90E-05 | 0.99724 | 0.444416 |
| Combined parent's age at death (z-score) | rs1982119 | 4:162719456_A_T | 0.235282 | 0.036458 | 0.008863 | 3.90E-05 | 0.99683 | 0.453921 |
| Combined parent's age at death (z-score) | rs5756828 | 22:38217061_G_C | 0.448088 | 0.031156 | 0.007572 | 3.90E-05 | 0.99145 | 0.025509 |
| Combined parent's age at death (z-score) | rs76586384 | 2:179242959_C_T | 0.979423 | -0.10947 | 0.026602 | 3.90E-05 | 1 | 0.001775 |
| Combined parent's age at death (z-score) | rs34188732 | 19:19778297_CA_C | 0.745402 | 0.035483 | 0.00863 | 3.90E-05 | 0.99538 | 0.687496 |
| Combined parent's age at death (z-score) | rs11878228 | 19:33567184_T_G | 0.915146 | 0.055726 | 0.01354 | 3.90E-05 | 0.98819 | 0.142456 |
| Combined parent's age at death (z-score) | rs8112039 | 19:53545534_G_A | 0.643672 | 0.034224 | 0.008324 | 3.90E-05 | 0.88497 | 0.25332 |
| Combined parent's age at death (z-score) | rs13319916 | 3:54238058_A_G | 0.652469 | -0.03258 | 0.007921 | 3.90E-05 | 0.98195 | 0.347392 |
| Combined parent's age at death (z-score) | rs144989011 | 5:157372698_C_T | 0.906427 | -0.05349 | 0.013004 | 3.90E-05 | 0.98493 | 0.80024 |
| Combined parent's age at death (z-score) | rs78814149 | 6:72110580_T_C | 0.955543 | 0.076138 | 0.018518 | 3.90E-05 | 0.97741 | 0.587733 |
| Combined parent's age at death (z-score) | rs77823684 | 6:72115845_T_C | 0.955543 | 0.076138 | 0.018518 | 3.90E-05 | 0.97741 | 0.587733 |
| Combined parent's age at death (z-score) | rs718834 | 9:114844799_C_A | 0.675652 | -0.0333 | 0.00809 | 3.90E-05 | 0.99469 | 0.905203 |
| Combined parent's age at death (z-score) | rs117248021 | 9:120306132_G_T | 0.982908 | -0.12196 | 0.029667 | 3.90E-05 | 0.95833 | 0.608808 |
| Combined parent's age at death (z-score) | rs4468707 | 18:27606758_G_A | 0.213023 | 0.037914 | 0.00922 | 3.90E-05 | 0.9991 | 0.11936 |
| Combined parent's age at death (z-score) | rs59231015 | 11:21148884_A_G | 0.804838 | -0.03929 | 0.009558 | 3.90E-05 | 0.99243 | 0.109668 |
| Combined parent's age at death (z-score) | rs12283473 | 11:18538263_T_C | 0.117597 | -0.04816 | 0.011712 | 3.90E-05 | 0.99265 | 0.095477 |
| Combined parent's age at death (z-score) | rs7943696 | 11:130581856_T_A | 0.757826 | 0.036512 | 0.008879 | 3.90E-05 | 0.96506 | 0.000387 |
| Combined parent's age at death (z-score) | rs144596004 | 11:108915869_G_A | 0.977368 | -0.10479 | 0.025458 | 3.90E-05 | 0.98532 | 0.693378 |
| Combined parent's age at death (z-score) | rs11036270 | 11:41193032_T_G | 0.842724 | -0.04266 | 0.010371 | 3.90E-05 | 0.99442 | 0.154736 |
| Combined parent's age at death (z-score) | rs142959682 | 17:6625923_A_C | 0.98978 | -0.17117 | 0.041591 | 3.90E-05 | 0.80067 | 0.551696 |
| Combined parent's age at death (z-score) | rs12944629 | 17:9719337_C_A | 0.55434 | 0.031272 | 0.007601 | 3.90E-05 | 0.98395 | 0.833645 |
| Combined parent's age at death (z-score) | rs8067825 | 17:9721536_A_G | 0.553555 | 0.03145 | 0.007655 | 4.00E-05 | 0.97131 | 0.95814 |
| Combined parent's age at death (z-score) | rs555676178 | 15:78760884_C_CT | 0.832563 | -0.04164 | 0.010138 | 4.00E-05 | 0.98409 | 0.429714 |
| Combined parent's age at death (z-score) | rs59111660 | 8:134175131_GT_G | 0.603541 | 0.03176 | 0.007735 | 4.00E-05 | 0.99148 | 0.167321 |
| Combined parent's age at death (z-score) | rs9408834 | 9:114844883_C_T | 0.678253 | -0.03335 | 0.008123 | 4.00E-05 | 0.9916 | 0.978799 |
| Combined parent's age at death (z-score) | rs62571465 | 9:79997124_T_C | 0.948353 | -0.07018 | 0.017091 | 4.00E-05 | 0.98227 | 0.372349 |
| Combined parent's age at death (z-score) | rs7109637 | 11:41185302_C_T | 0.843342 | -0.04263 | 0.010378 | 4.00E-05 | 0.99533 | 0.18332 |
| Combined parent's age at death (z-score) | rs199522841 | 11:18566599_C_CTA | 0.882428 | 0.047987 | 0.011689 | 4.00E-05 | 0.99621 | 0.122758 |
| Combined parent's age at death (z-score) | rs1434623 | 4:162699479_G_A | 0.235155 | 0.036379 | 0.008856 | 4.00E-05 | 0.99929 | 0.560596 |
| Combined parent's age at death (z-score) | rs1434624 | 4:162699530_A_T | 0.234758 | 0.036423 | 0.008866 | 4.00E-05 | 0.99813 | 0.538877 |
| Combined parent's age at death (z-score) | rs1347354 | 4:162702702_G_A | 0.235102 | 0.036368 | 0.008857 | 4.00E-05 | 0.99926 | 0.539237 |
| Combined parent's age at death (z-score) | rs13110364 | 4:162719425_C_T | 0.235345 | 0.0364 | 0.008864 | 4.00E-05 | 0.99656 | 0.454004 |
| Combined parent's age at death (z-score) | rs1821469 | 4:162724752_T_A | 0.235514 | 0.036388 | 0.008863 | 4.00E-05 | 0.99677 | 0.655526 |
| Combined parent's age at death (z-score) | rs72698728 | 14:101192954_C_G | 0.876802 | -0.04776 | 0.011628 | 4.00E-05 | 0.96777 | 0.520943 |
| Combined parent's age at death (z-score) | rs6737461 | 2:33889704_A_T | 0.231702 | 0.036748 | 0.008945 | 4.00E-05 | 0.98988 | 0.84566 |
| Combined parent's age at death (z-score) | rs558852141 | 6:57415189_C_T | 0.989596 | -0.18681 | 0.04547 | 4.00E-05 | 0.66201 | 0.122287 |
| Combined parent's age at death (z-score) | rs62382815 | 5:167060215_T_A | 0.908086 | 0.054885 | 0.013385 | 4.10E-05 | 0.94271 | 0.727009 |
| Combined parent's age at death (z-score) | rs149998442 | 5:112784140_C_G | 0.98899 | -0.15095 | 0.036779 | 4.10E-05 | 0.96285 | 1 |
| Combined parent's age at death (z-score) | rs75785250 | 5:25311326_T_C | 0.983427 | -0.12438 | 0.030306 | 4.10E-05 | 0.95391 | 0.387597 |
| Combined parent's age at death (z-score) | rs75354418 | 5:25172080_C_T | 0.982471 | -0.12118 | 0.029563 | 4.10E-05 | 0.94915 | 0.935906 |
| Combined parent's age at death (z-score) | rs141247529 | 3:150377064_G_C | 0.988385 | 0.157945 | 0.038509 | 4.10E-05 | 0.82869 | 0.057425 |
| Combined parent's age at death (z-score) | rs17392588 | 11:18586871_A_C | 0.882343 | 0.047919 | 0.011679 | 4.10E-05 | 0.99819 | 0.116493 |
| Combined parent's age at death (z-score) | rs1516752 | 11:21085218_G_C | 0.838075 | -0.04222 | 0.010292 | 4.10E-05 | 0.99208 | 0.201094 |
| Combined parent's age at death (z-score) | rs146437939 | 17:9719685_G_GTGTC | 0.556527 | 0.031235 | 0.007619 | 4.10E-05 | 0.98054 | 0.838039 |
| Combined parent's age at death (z-score) | rs62066026 | 17:9720108_G_A | 0.568934 | 0.031705 | 0.007725 | 4.10E-05 | 0.9594 | 0.929745 |
| Combined parent's age at death (z-score) | rs2320262 | 17:9721722_C_G | 0.554241 | 0.031601 | 0.007705 | 4.10E-05 | 0.95929 | 0.967427 |
| Combined parent's age at death (z-score) | rs850791 | 15:23936783_G_A | 0.0924729 | -0.05387 | 0.013125 | 4.10E-05 | 0.98489 | 0.245358 |
| Combined parent's age at death (z-score) | rs4350644 | 18:27613949_T_C | 0.213393 | 0.037854 | 0.009225 | 4.10E-05 | 0.99697 | 0.111946 |
| Combined parent's age at death (z-score) | rs185368687 | 9:4262959_C_T | 0.962116 | 0.080976 | 0.019751 | 4.10E-05 | 0.9849 | 0.37894 |
| Combined parent's age at death (z-score) | rs111889942 | 8:27459339_A_AGCCAGGCGTGCTG | 0.7418 | -0.03583 | 0.008736 | 4.10E-05 | 0.96697 | 0.026248 |
| Combined parent's age at death (z-score) | rs28486007 | 8:10804346_G_A | 0.736993 | -0.03503 | 0.00854 | 4.10E-05 | 1 | 0.88771 |
| Combined parent's age at death (z-score) | rs11124328 | 2:33863202_T_C | 0.0916012 | 0.053466 | 0.013039 | 4.10E-05 | 1 | 0.166682 |
| Combined parent's age at death (z-score) | rs73919714 | 2:23722921_A_G | 0.905965 | -0.05262 | 0.012826 | 4.10E-05 | 1 | 0.671955 |
| Combined parent's age at death (z-score) | rs13005539 | 2:41237408_T_C | 0.62459 | 0.032347 | 0.007883 | 4.10E-05 | 0.97753 | 0.844195 |
| Combined parent's age at death (z-score) | rs2798287 | 4:2938651_C_A | 0.504153 | 0.031089 | 0.007584 | 4.10E-05 | 0.98178 | 0.429507 |
| Combined parent's age at death (z-score) | rs13146883 | 4:162708071_T_A | 0.2351 | 0.036356 | 0.008864 | 4.10E-05 | 0.99749 | 0.544565 |
| Combined parent's age at death (z-score) | rs7676997 | 4:162716030_G_A | 0.235095 | 0.036364 | 0.008865 | 4.10E-05 | 0.99693 | 0.482981 |
| Combined parent's age at death (z-score) | rs117912778 | 12:12916226_T_G | 0.976762 | 0.102906 | 0.025104 | 4.10E-05 | 1 | 2.01E-06 |
| Combined parent's age at death (z-score) | rs4690075 | 4:3173273_C_T | 0.680714 | -0.03308 | 0.008073 | 4.20E-05 | 0.99663 | 0.088818 |
| Combined parent's age at death (z-score) | rs146326847 | 4:25240912_G_A | 0.963171 | 0.083323 | 0.020337 | 4.20E-05 | 0.96268 | 0.748928 |
| Combined parent's age at death (z-score) | rs59100189 | 14:101184901_G_A | 0.836354 | -0.04202 | 0.010257 | 4.20E-05 | 0.9861 | 0.257448 |
| Combined parent's age at death (z-score) | rs546229041 | 1:21967567_A_AT | 0.493035 | 0.032214 | 0.007867 | 4.20E-05 | 0.90688 | 0.677922 |
| Combined parent's age at death (z-score) | rs34879269 | 2:41237387_A_G | 0.624621 | 0.032311 | 0.007883 | 4.20E-05 | 0.97766 | 0.848999 |
| Combined parent's age at death (z-score) | rs6837801 | 4:162698496_G_C | 0.235241 | 0.036265 | 0.008854 | 4.20E-05 | 0.99942 | 0.549984 |
| Combined parent's age at death (z-score) | rs11937221 | 4:162699017_A_G | 0.23522 | 0.036255 | 0.008854 | 4.20E-05 | 0.99952 | 0.555303 |
| Combined parent's age at death (z-score) | rs11946425 | 4:162699020_G_A | 0.23522 | 0.036255 | 0.008854 | 4.20E-05 | 0.99952 | 0.555303 |
| Combined parent's age at death (z-score) | rs6841007 | 4:162700722_T_C | 0.235199 | 0.03629 | 0.008855 | 4.20E-05 | 0.99948 | 0.549946 |
| Combined parent's age at death (z-score) | rs6815645 | 4:162700825_C_G | 0.234972 | 0.036282 | 0.008859 | 4.20E-05 | 0.99936 | 0.560415 |
| Combined parent's age at death (z-score) | rs7665737 | 4:162701948_A_C | 0.235189 | 0.036284 | 0.008856 | 4.20E-05 | 0.99938 | 0.528847 |
| Combined parent's age at death (z-score) | rs1975224 | 4:162705907_G_A | 0.235131 | 0.036298 | 0.008856 | 4.20E-05 | 0.99934 | 0.571321 |
| Combined parent's age at death (z-score) | rs1975225 | 4:162705971_A_G | 0.23513 | 0.036295 | 0.008856 | 4.20E-05 | 0.99933 | 0.571308 |
| Combined parent's age at death (z-score) | rs7677571 | 4:162716320_G_T | 0.235076 | 0.036319 | 0.008866 | 4.20E-05 | 0.99666 | 0.487933 |
| Combined parent's age at death (z-score) | rs7682730 | 4:162716575_G_A | 0.235344 | 0.036311 | 0.008859 | 4.20E-05 | 0.99751 | 0.4783 |
| Combined parent's age at death (z-score) | rs5763687 | 22:30392772_A_G | 0.979147 | -0.10873 | 0.026538 | 4.20E-05 | 0.99175 | 0.526842 |
| Combined parent's age at death (z-score) | rs72816508 | 5:157356031_G_T | 0.906122 | -0.05318 | 0.012986 | 4.20E-05 | 0.98574 | 0.775015 |
| Combined parent's age at death (z-score) | rs149761488 | 13:19213460_C_T | 0.982212 | 0.120292 | 0.029361 | 4.20E-05 | 0.93528 | 0.151293 |
| Combined parent's age at death (z-score) | rs112042879 | 6:72118627_C_G | 0.955059 | 0.075975 | 0.018555 | 4.20E-05 | 0.96464 | 0.614455 |
| Combined parent's age at death (z-score) | rs8067724 | 17:9721775_C_T | 0.553983 | 0.031603 | 0.007719 | 4.20E-05 | 0.95605 | 0.995346 |
| Combined parent's age at death (z-score) | rs8068320 | 17:9721776_A_G | 0.553881 | 0.031764 | 0.00775 | 4.20E-05 | 0.9485 | 1 |
| Combined parent's age at death (z-score) | rs16935557 | 11:18591992_G_A | 0.88241 | 0.04783 | 0.011684 | 4.20E-05 | 0.99759 | 0.110047 |
| Combined parent's age at death (z-score) | rs113201690 | 18:51038707_T_C | 0.535024 | 0.030942 | 0.007557 | 4.20E-05 | 0.99287 | 0.745727 |
| Combined parent's age at death (z-score) | rs185257697 | 16:50313002_C_T | 0.987912 | 0.147842 | 0.036076 | 4.20E-05 | 0.9061 | 0.389538 |
| Combined parent's age at death (z-score) | 17:9720096_GTC_G | 17:9720096_G_A | 0.560937 | 0.031377 | 0.007674 | 4.30E-05 | 0.61868 | 1 |
| Combined parent's age at death (z-score) | 17:9720096_GTC_G | 17:9720096_GTC_G | 0.560937 | 0.031377 | 0.007674 | 4.30E-05 | 0.97086 | 0.879077 |
| Combined parent's age at death (z-score) | rs117387787 | 10:105365517_A_G | 0.980368 | 0.115057 | 0.028134 | 4.30E-05 | 0.92507 | 0.544189 |
| Combined parent's age at death (z-score) | rs35557446 | 18:51034571_A_AT | 0.534257 | 0.031012 | 0.007583 | 4.30E-05 | 0.98472 | 0.55874 |
| Combined parent's age at death (z-score) | rs4461153 | 18:27606769_A_G | 0.215276 | 0.037677 | 0.009206 | 4.30E-05 | 0.99482 | 0.139576 |
| Combined parent's age at death (z-score) | rs7238896 | 18:1840658_A_G | 0.859358 | 0.044598 | 0.010909 | 4.30E-05 | 0.97805 | 0.327115 |
| Combined parent's age at death (z-score) | rs2271079 | 11:18562982_C_T | 0.88245 | 0.04783 | 0.011692 | 4.30E-05 | 0.99631 | 0.107046 |
| Combined parent's age at death (z-score) | rs1260149 | 11:21159558_T_C | 0.802505 | -0.03899 | 0.009534 | 4.30E-05 | 0.99434 | 0.019449 |
| Combined parent's age at death (z-score) | rs190997287 | 11:72359590_C_T | 0.98833 | 0.147637 | 0.036089 | 4.30E-05 | 0.94771 | 0.253355 |
| Combined parent's age at death (z-score) | rs781955 | 9:114896872_A_G | 0.586379 | -0.0314 | 0.007677 | 4.30E-05 | 0.99488 | 0.250594 |
| Combined parent's age at death (z-score) | rs73615262 | 13:110619049_C_T | 0.958293 | -0.07745 | 0.018936 | 4.30E-05 | 0.98662 | 0.830248 |
| Combined parent's age at death (z-score) | rs2193597 | 14:75234329_G_A | 0.333812 | 0.032698 | 0.007988 | 4.30E-05 | 0.99987 | 0.335066 |
| Combined parent's age at death (z-score) | rs67569915 | 4:162712557_CAA_C | 0.234674 | 0.036296 | 0.00887 | 4.30E-05 | 0.99726 | 0.533568 |
| Combined parent's age at death (z-score) | rs1594901 | 4:162714118_C_G | 0.235232 | 0.036223 | 0.008855 | 4.30E-05 | 0.99915 | 0.544716 |
| Combined parent's age at death (z-score) | rs7676679 | 4:162716060_A_C | 0.235239 | 0.036264 | 0.00886 | 4.30E-05 | 0.99761 | 0.488158 |
| Combined parent's age at death (z-score) | rs13135079 | 4:162724065_T_C | 0.235337 | 0.036236 | 0.008862 | 4.30E-05 | 0.99778 | 0.609972 |
| Combined parent's age at death (z-score) | rs4277843 | 4:111777749_A_G | 0.226263 | -0.03701 | 0.009051 | 4.30E-05 | 0.98591 | 0.445769 |
| Combined parent's age at death (z-score) | rs112546886 | 6:72161276_G_A | 0.956061 | 0.076318 | 0.018645 | 4.30E-05 | 0.97033 | 0.679251 |
| Combined parent's age at death (z-score) | rs6568453 | 6:107264985_T_C | 0.368076 | -0.03327 | 0.008138 | 4.30E-05 | 0.9264 | 0.660344 |
| Combined parent's age at death (z-score) | rs12460367 | 19:33599961_A_G | 0.906756 | 0.052793 | 0.012909 | 4.30E-05 | 0.99696 | 0.092683 |
| Combined parent's age at death (z-score) | rs139417721 | 20:10392509_T_C | 0.987478 | -0.15096 | 0.03691 | 4.30E-05 | 0.8339 | 0.472835 |
| Combined parent's age at death (z-score) | rs147937094 | 13:19204514_G_A | 0.983437 | 0.121546 | 0.029697 | 4.30E-05 | 0.97707 | 0.308042 |
| Combined parent's age at death (z-score) | rs140873147 | 5:157357591_TGA_T | 0.906181 | -0.05311 | 0.012986 | 4.30E-05 | 0.98586 | 0.736411 |
| Combined parent's age at death (z-score) | rs76360533 | 6:72120033_C_T | 0.955893 | 0.075758 | 0.018555 | 4.40E-05 | 0.9815 | 0.58479 |
| Combined parent's age at death (z-score) | rs10418246 | 19:33572397_G_A | 0.915493 | 0.055417 | 0.013569 | 4.40E-05 | 0.98844 | 0.205338 |
| Combined parent's age at death (z-score) | rs13345828 | 19:33599323_G_T | 0.905846 | 0.052437 | 0.012835 | 4.40E-05 | 0.99867 | 0.063577 |
| Combined parent's age at death (z-score) | rs8103733 | 19:33604531_C_T | 0.906364 | 0.05255 | 0.012859 | 4.40E-05 | 1 | 0.047698 |
| Combined parent's age at death (z-score) | rs77974295 | 3:150472259_C_T | 0.989319 | 0.167966 | 0.041137 | 4.40E-05 | 0.78654 | 0.686136 |
| Combined parent's age at death (z-score) | rs10936995 | 3:178973722_A_C | 0.508952 | -0.03099 | 0.007584 | 4.40E-05 | 0.99777 | 0.214872 |
| Combined parent's age at death (z-score) | rs11004598 | 10:56613047_G_A | 0.765221 | 0.037771 | 0.009247 | 4.40E-05 | 0.92403 | 0.595717 |
| Combined parent's age at death (z-score) | rs11004599 | 10:56613051_A_G | 0.765222 | 0.037779 | 0.009248 | 4.40E-05 | 0.92398 | 0.595703 |
| Combined parent's age at death (z-score) | rs62513764 | 8:68371330_T_C | 0.968445 | -0.0931 | 0.022785 | 4.40E-05 | 0.88563 | 0.66558 |
| Combined parent's age at death (z-score) | rs72871727 | 11:18522316_G_A | 0.882344 | 0.047824 | 0.011709 | 4.40E-05 | 0.9917 | 0.126468 |
| Combined parent's age at death (z-score) | rs2658547 | 11:18528815_A_G | 0.117888 | -0.04781 | 0.011696 | 4.40E-05 | 0.99305 | 0.101801 |
| Combined parent's age at death (z-score) | rs1973412 | 11:18535343_T_C | 0.117636 | -0.04782 | 0.011701 | 4.40E-05 | 0.99351 | 0.116397 |
| Combined parent's age at death (z-score) | rs72871772 | 11:18547801_G_A | 0.882473 | 0.047815 | 0.011711 | 4.40E-05 | 0.99303 | 0.101002 |
| Combined parent's age at death (z-score) | rs7687422 | 4:162699997_A_T | 0.235667 | 0.036201 | 0.008855 | 4.40E-05 | 0.99798 | 0.52942 |
| Combined parent's age at death (z-score) | rs9686044 | 4:162700252_G_A | 0.235109 | 0.036205 | 0.008857 | 4.40E-05 | 0.99927 | 0.565926 |
| Combined parent's age at death (z-score) | rs7665128 | 4:162701583_C_T | 0.235165 | 0.036198 | 0.008856 | 4.40E-05 | 0.99944 | 0.539299 |
| Combined parent's age at death (z-score) | rs7666089 | 4:162702044_C_G | 0.235165 | 0.036198 | 0.008856 | 4.40E-05 | 0.99945 | 0.539299 |
| Combined parent's age at death (z-score) | rs7679398 | 4:162704139_C_T | 0.235165 | 0.036198 | 0.008856 | 4.40E-05 | 0.99945 | 0.539299 |
| Combined parent's age at death (z-score) | rs6841860 | 4:162704354_A_G | 0.235165 | 0.036198 | 0.008856 | 4.40E-05 | 0.99945 | 0.539299 |
| Combined parent's age at death (z-score) | rs13130537 | 4:162718372_T_C | 0.235519 | 0.036232 | 0.008866 | 4.40E-05 | 0.99496 | 0.394385 |
| Combined parent's age at death (z-score) | rs66684864 | 4:3182849_T_G | 0.68048 | -0.033 | 0.008075 | 4.40E-05 | 0.99557 | 0.079396 |
| Combined parent's age at death (z-score) | rs10133241 | 14:75098193_A_G | 0.432798 | 0.0311 | 0.007614 | 4.40E-05 | 0.98786 | 0.201442 |
| Combined parent's age at death (z-score) | rs34043436 | 2:41247694_C_G | 0.592441 | 0.032085 | 0.007858 | 4.40E-05 | 0.95277 | 0.707066 |
| Combined parent's age at death (z-score) | rs35261648 | 2:41247746_C_A | 0.592433 | 0.032105 | 0.007858 | 4.40E-05 | 0.95275 | 0.707066 |
| Combined parent's age at death (z-score) | rs7589532 | 2:41248468_A_G | 0.592443 | 0.032093 | 0.007858 | 4.40E-05 | 0.95278 | 0.707066 |
| Combined parent's age at death (z-score) | rs1975226 | 4:162706053_C_A | 0.235377 | 0.036132 | 0.008858 | 4.50E-05 | 0.99818 | 0.577019 |
| Combined parent's age at death (z-score) | rs11100389 | 4:162711890_A_T | 0.235307 | 0.036137 | 0.008855 | 4.50E-05 | 0.99894 | 0.555431 |
| Combined parent's age at death (z-score) | rs1434609 | 4:162712090_T_C | 0.235267 | 0.036151 | 0.008855 | 4.50E-05 | 0.99902 | 0.539473 |
| Combined parent's age at death (z-score) | rs113291422 | 4:157544479_TAA_T | 0.745618 | 0.035395 | 0.008679 | 4.50E-05 | 0.98693 | 0.93339 |
| Combined parent's age at death (z-score) | rs4115271 | 4:111783779_G_A | 0.222702 | -0.03693 | 0.009049 | 4.50E-05 | 0.99662 | 0.690526 |
| Combined parent's age at death (z-score) | rs1298631 | 4:111792537_C_G | 0.230193 | -0.03649 | 0.008946 | 4.50E-05 | 0.99623 | 0.377181 |
| Combined parent's age at death (z-score) | rs1891701 | 4:3141130_G_A | 0.681892 | -0.03303 | 0.008094 | 4.50E-05 | 0.99445 | 0.085645 |
| Combined parent's age at death (z-score) | rs13175049 | 5:152502998_C_G | 0.978341 | -0.10528 | 0.025799 | 4.50E-05 | 1 | 0.235738 |
| Combined parent's age at death (z-score) | rs3786934 | 19:33602856_A_G | 0.906201 | 0.052471 | 0.012853 | 4.50E-05 | 0.99953 | 0.084528 |
| Combined parent's age at death (z-score) | rs11667844 | 19:19775740_T_G | 0.745263 | 0.035256 | 0.008635 | 4.50E-05 | 0.99404 | 0.831729 |
| Combined parent's age at death (z-score) | rs7249670 | 19:19783420_T_C | 0.745129 | 0.035244 | 0.008642 | 4.50E-05 | 0.99216 | 0.76157 |
| Combined parent's age at death (z-score) | rs566488245 | 6:68395621_A_C | 0.812582 | -0.04015 | 0.009834 | 4.50E-05 | 0.96681 | 0.296647 |
| Combined parent's age at death (z-score) | rs72871724 | 11:18521989_C_T | 0.882575 | 0.047794 | 0.011718 | 4.50E-05 | 0.99219 | 0.125791 |
| Combined parent's age at death (z-score) | rs10837586 | 11:41175225_C_A | 0.843198 | -0.04233 | 0.010374 | 4.50E-05 | 0.9956 | 0.176795 |
| Combined parent's age at death (z-score) | rs7109993 | 11:41185452_G_A | 0.843137 | -0.04229 | 0.010369 | 4.50E-05 | 0.99629 | 0.176807 |
| Combined parent's age at death (z-score) | rs71372276 | 17:18200779_G_C | 0.595255 | 0.032009 | 0.007849 | 4.50E-05 | 0.95409 | 0.552484 |
| Combined parent's age at death (z-score) | rs2292116 | 15:78789676_T_C | 0.835147 | -0.0413 | 0.010122 | 4.50E-05 | 0.99873 | 0.528032 |
| Combined parent's age at death (z-score) | rs4630627 | 18:27599333_C_T | 0.211752 | 0.037782 | 0.009259 | 4.50E-05 | 0.99576 | 0.095455 |
| Combined parent's age at death (z-score) | rs112337935 | 15:78773693_C_T | 0.836597 | -0.04144 | 0.010165 | 4.60E-05 | 0.99699 | 0.484685 |
| Combined parent's age at death (z-score) | rs149959208 | 15:78912710_T_TGCGCGGGGCAGGGCGACGGGCA | 0.665318 | 0.032773 | 0.008045 | 4.60E-05 | 0.9966 | 0.0264 |
| Combined parent's age at death (z-score) | rs181411579 | 9:33263640_G_A | 0.987322 | -0.1402 | 0.034383 | 4.60E-05 | 0.93351 | 0.005003 |
| Combined parent's age at death (z-score) | rs62571394 | 9:79706937_T_G | 0.919088 | -0.05605 | 0.013759 | 4.60E-05 | 0.99679 | 0.332086 |
| Combined parent's age at death (z-score) | rs8067462 | 17:18263571_C_A | 0.600492 | 0.03132 | 0.007689 | 4.60E-05 | 0.99644 | 0.847083 |
| Combined parent's age at death (z-score) | rs8181489 | 11:41189446_C_G | 0.842558 | -0.04222 | 0.010361 | 4.60E-05 | 0.99542 | 0.154964 |
| Combined parent's age at death (z-score) | rs985295 | 11:41201624_A_C | 0.844101 | -0.04239 | 0.010405 | 4.60E-05 | 0.99404 | 0.107954 |
| Combined parent's age at death (z-score) | rs2643868 | 11:18527393_T_C | 0.117805 | -0.04765 | 0.011699 | 4.60E-05 | 0.99301 | 0.104482 |
| Combined parent's age at death (z-score) | rs77461213 | 11:21151037_G_A | 0.810511 | -0.03939 | 0.009671 | 4.60E-05 | 0.99238 | 0.05793 |
| Combined parent's age at death (z-score) | rs61884750 | 11:30582806_G_T | 0.848119 | -0.04256 | 0.010451 | 4.60E-05 | 1 | 0.946889 |
| Combined parent's age at death (z-score) | rs113294358 | 4:3110794_AT_A | 0.64933 | -0.03223 | 0.007905 | 4.60E-05 | 0.9945 | 0.101791 |
| Combined parent's age at death (z-score) | rs199585288 | 4:3110796_AT_A | 0.64933 | -0.03223 | 0.007905 | 4.60E-05 | 0.9945 | 0.101791 |
| Combined parent's age at death (z-score) | rs2298967 | 4:3185747_T_C | 0.680401 | -0.03292 | 0.008074 | 4.60E-05 | 0.99594 | 0.081733 |
| Combined parent's age at death (z-score) | rs362336 | 4:3213832_G_A | 0.680929 | -0.03289 | 0.008066 | 4.60E-05 | 0.99877 | 0.097814 |
| Combined parent's age at death (z-score) | rs4690081 | 4:3262105_T_G | 0.611842 | -0.03163 | 0.007763 | 4.60E-05 | 0.99285 | 0.044627 |
| Combined parent's age at death (z-score) | rs142692478 | 4:25266544_G_A | 0.963839 | 0.084437 | 0.020731 | 4.60E-05 | 0.94177 | 0.714463 |
| Combined parent's age at death (z-score) | rs73919712 | 2:23722341_A_G | 0.906982 | -0.05258 | 0.012911 | 4.60E-05 | 0.99675 | 0.632106 |
| Combined parent's age at death (z-score) | rs11563204 | 2:234917377_G_A | 0.800329 | -0.03845 | 0.009431 | 4.60E-05 | 0.99301 | 0.88627 |
| Combined parent's age at death (z-score) | rs11281968 | 4:162700896_G_GGATTATTT | 0.235731 | 0.036101 | 0.008856 | 4.60E-05 | 0.99768 | 0.52942 |
| Combined parent's age at death (z-score) | rs7665339 | 4:162701725_C_T | 0.235143 | 0.036094 | 0.008857 | 4.60E-05 | 0.99928 | 0.565926 |
| Combined parent's age at death (z-score) | rs1075273 | 4:162713575_T_G | 0.235203 | 0.036096 | 0.008856 | 4.60E-05 | 0.99897 | 0.539399 |
| Combined parent's age at death (z-score) | rs1560927 | 4:162715455_C_T | 0.235882 | 0.036108 | 0.008858 | 4.60E-05 | 0.99677 | 0.498953 |
| Combined parent's age at death (z-score) | rs7159354 | 14:75253847_A_G | 0.312984 | 0.032944 | 0.008087 | 4.60E-05 | 1 | 0.000374 |
| Combined parent's age at death (z-score) | rs10133424 | 14:83264580_A_G | 0.684099 | -0.03303 | 0.008102 | 4.60E-05 | 1 | 0.671352 |
| Combined parent's age at death (z-score) | rs11582745 | 1:59835699_G_T | 0.963938 | 0.090827 | 0.022298 | 4.60E-05 | 0.81029 | 0.026098 |
| Combined parent's age at death (z-score) | rs74535640 | 6:72105694_A_G | 0.955532 | 0.075373 | 0.018499 | 4.60E-05 | 0.9793 | 0.587746 |
| Combined parent's age at death (z-score) | rs74739435 | 6:72157588_T_C | 0.955956 | 0.075865 | 0.018625 | 4.60E-05 | 0.97017 | 0.655043 |
| Combined parent's age at death (z-score) | rs77658242 | 6:72158069_C_T | 0.955956 | 0.075865 | 0.018625 | 4.60E-05 | 0.97017 | 0.655043 |
| Combined parent's age at death (z-score) | rs111317382 | 6:72161759_C_A | 0.955956 | 0.075869 | 0.018625 | 4.60E-05 | 0.97018 | 0.655043 |
| Combined parent's age at death (z-score) | rs2165584 | 3:178249095_T_G | 0.685004 | -0.0331 | 0.008133 | 4.70E-05 | 0.99492 | 0.683015 |
| Combined parent's age at death (z-score) | rs11963678 | 6:22160595_G_C | 0.969934 | -0.09064 | 0.02226 | 4.70E-05 | 0.97782 | 0.96028 |
| Combined parent's age at death (z-score) | rs12463355 | 19:33572972_A_G | 0.909501 | 0.054276 | 0.013339 | 4.70E-05 | 0.96152 | 0.08841 |
| Combined parent's age at death (z-score) | rs13173299 | 5:152507916_C_T | 0.780584 | -0.03728 | 0.009162 | 4.70E-05 | 0.97943 | 0.290329 |
| Combined parent's age at death (z-score) | rs57203218 | 13:19204676_C_G | 0.983792 | 0.120947 | 0.02971 | 4.70E-05 | 1 | 0.259974 |
| Combined parent's age at death (z-score) | rs3019724 | 11:96581526_C_T | 0.428011 | 0.031566 | 0.007755 | 4.70E-05 | 0.95132 | 0.225082 |
| Combined parent's age at death (z-score) | rs72876735 | 11:18555212_A_G | 0.882178 | 0.047555 | 0.01168 | 4.70E-05 | 0.99631 | 0.101707 |
| Combined parent's age at death (z-score) | rs140284508 | 11:9032177_G_C | 0.921366 | -0.05704 | 0.014012 | 4.70E-05 | 0.99337 | 0.718191 |
| Combined parent's age at death (z-score) | rs77612006 | 11:9034984_A_C | 0.921343 | -0.05702 | 0.014012 | 4.70E-05 | 0.99305 | 0.733314 |
| Combined parent's age at death (z-score) | rs78207582 | 11:9035283_G_A | 0.921366 | -0.05705 | 0.014012 | 4.70E-05 | 0.99336 | 0.718191 |
| Combined parent's age at death (z-score) | rs57338032 | 15:78798939_A_G | 0.83576 | -0.04159 | 0.010219 | 4.70E-05 | 0.98314 | 0.590487 |
| Combined parent's age at death (z-score) | rs4243084 | 15:78911672_G_C | 0.663408 | 0.03266 | 0.008024 | 4.70E-05 | 1 | 0.021963 |
| Combined parent's age at death (z-score) | rs11645642 | 16:49936901_A_T | 0.900061 | 0.051892 | 0.012744 | 4.70E-05 | 0.96806 | 0.637148 |
| Combined parent's age at death (z-score) | rs66969288 | 8:27459640_G_A | 0.732831 | -0.03467 | 0.00852 | 4.70E-05 | 0.99389 | 0.01953 |
| Combined parent's age at death (z-score) | rs146323377 | 9:6319760_A_G | 0.978649 | 0.110042 | 0.027034 | 4.70E-05 | 0.92086 | 0.165067 |
| Combined parent's age at death (z-score) | rs781957 | 9:114898634_A_C | 0.585411 | -0.03124 | 0.007671 | 4.70E-05 | 0.99508 | 0.317556 |
| Combined parent's age at death (z-score) | rs112833631 | 13:110614217_C_A | 0.969591 | -0.09046 | 0.022219 | 4.70E-05 | 0.97635 | 1 |
| Combined parent's age at death (z-score) | rs2164129 | 4:162703219_C_T | 0.235193 | 0.036039 | 0.008856 | 4.70E-05 | 0.99937 | 0.549921 |
| Combined parent's age at death (z-score) | rs1836294 | 4:162714028_A_G | 0.235222 | 0.036027 | 0.008856 | 4.70E-05 | 0.99894 | 0.539436 |
| Combined parent's age at death (z-score) | rs1975229 | 4:162714839_T_C | 0.234958 | 0.03608 | 0.008863 | 4.70E-05 | 0.99854 | 0.587516 |
| Combined parent's age at death (z-score) | rs199911714 | 4:157524687_A_G | 0.349802 | -0.03213 | 0.007896 | 4.70E-05 | 0.98931 | 0.148044 |
| Combined parent's age at death (z-score) | rs4285153 | 4:111778733_A_G | 0.222589 | -0.03686 | 0.009052 | 4.70E-05 | 0.99649 | 0.684353 |
| Combined parent's age at death (z-score) | rs1019047 | 4:111805594_A_G | 0.229918 | -0.03653 | 0.008978 | 4.70E-05 | 0.98976 | 0.408169 |
| Combined parent's age at death (z-score) | rs6840579 | 4:162700493_T_A | 0.234632 | 0.036094 | 0.008875 | 4.80E-05 | 0.99714 | 0.587192 |
| Combined parent's age at death (z-score) | rs6574188 | 14:75106960_A_G | 0.452239 | 0.030646 | 0.007541 | 4.80E-05 | 0.9958 | 0.00798 |
| Combined parent's age at death (z-score) | rs34871676 | 2:41247430_G_C | 0.592692 | 0.031936 | 0.00786 | 4.80E-05 | 0.95248 | 0.720361 |
| Combined parent's age at death (z-score) | rs75753793 | 1:226118336_C_T | 0.964338 | -0.08339 | 0.020508 | 4.80E-05 | 0.97876 | 0.562225 |
| Combined parent's age at death (z-score) | rs17865678 | 2:234919314_G_A | 0.730951 | -0.03453 | 0.008492 | 4.80E-05 | 0.99695 | 0.77046 |
| Combined parent's age at death (z-score) | rs3213844 | 2:222301357_T_C | 0.264895 | 0.035111 | 0.008633 | 4.80E-05 | 0.97198 | 0.272891 |
| Combined parent's age at death (z-score) | rs143342731 | 5:16573160_C_T | 0.968323 | 0.097874 | 0.024082 | 4.80E-05 | 0.79529 | 0.963443 |
| Combined parent's age at death (z-score) | rs1648811 | 15:42222390_A_G | 0.489019 | -0.03187 | 0.00784 | 4.80E-05 | 0.91852 | 0.917301 |
| Combined parent's age at death (z-score) | rs1857909 | 11:18548881_A_G | 0.1176 | -0.04762 | 0.011708 | 4.80E-05 | 0.99289 | 0.107046 |
| Combined parent's age at death (z-score) | rs72876779 | 11:18569810_A_C | 0.88283 | 0.047637 | 0.011716 | 4.80E-05 | 0.99503 | 0.128505 |
| Combined parent's age at death (z-score) | rs77516091 | 11:21084814_C_T | 0.83893 | -0.04188 | 0.010303 | 4.80E-05 | 0.9957 | 0.15288 |
| Combined parent's age at death (z-score) | rs681998 | 17:18264449_A_G | 0.602431 | 0.03126 | 0.007692 | 4.80E-05 | 0.99804 | 0.804491 |
| Combined parent's age at death (z-score) | rs35616645 | 9:118644122_C_CT | 0.671962 | 0.036898 | 0.009085 | 4.90E-05 | 0.77723 | 0.926996 |
| Combined parent's age at death (z-score) | rs6999469 | 8:135577530_G_A | 0.757442 | -0.03563 | 0.008772 | 4.90E-05 | 0.99485 | 0.004542 |
| Combined parent's age at death (z-score) | rs72876768 | 11:18564927_A_G | 0.882425 | 0.047503 | 0.011696 | 4.90E-05 | 0.9952 | 0.116115 |
| Combined parent's age at death (z-score) | rs10837592 | 11:41186712_G_A | 0.835599 | -0.04146 | 0.010216 | 4.90E-05 | 0.98746 | 0.053186 |
| Combined parent's age at death (z-score) | rs2919037 | 11:96596128_G_A | 0.425364 | 0.031295 | 0.007707 | 4.90E-05 | 0.96416 | 0.178636 |
| Combined parent's age at death (z-score) | rs2404665 | 18:72869656_A_G | 0.870489 | -0.04527 | 0.011152 | 4.90E-05 | 1 | 0.740014 |
| Combined parent's age at death (z-score) | rs8011071 | 14:75286705_T_C | 0.315669 | 0.032874 | 0.008098 | 4.90E-05 | 0.99876 | 0.074148 |
| Combined parent's age at death (z-score) | rs363099 | 4:3162056_C_T | 0.68096 | -0.03279 | 0.008074 | 4.90E-05 | 0.99689 | 0.095126 |
| Combined parent's age at death (z-score) | rs11276773 | 4:3174496_TGATTCACA_T | 0.681701 | -0.03283 | 0.008083 | 4.90E-05 | 0.99678 | 0.101538 |
| Combined parent's age at death (z-score) | rs73193303 | 4:3192200_A_G | 0.681469 | -0.0328 | 0.008078 | 4.90E-05 | 0.99734 | 0.114884 |
| Combined parent's age at death (z-score) | rs3135169 | 4:3262374_C_T | 0.612006 | -0.03154 | 0.007764 | 4.90E-05 | 0.9927 | 0.046569 |
| Combined parent's age at death (z-score) | rs6840765 | 4:162700542_T_G | 0.23453 | 0.036059 | 0.008878 | 4.90E-05 | 0.99679 | 0.57069 |
| Father's age at death | rs4243084 | 15:78911672_G_C | 0.659924 | 0.027688 | 0.004845 | 1.10E-08 | 1 | 0.021963 |
| Father's age at death | rs931794 | 15:78826180_G_A | 0.339898 | -0.0276 | 0.004844 | 1.20E-08 | 1 | 0.078813 |
| Father's age at death | rs564549950 | 15:78899560_CAA_C | 0.662675 | 0.027712 | 0.004859 | 1.20E-08 | 0.99789 | 0.031412 |
| Father's age at death | rs8039449 | 15:78914534_C_T | 0.631833 | 0.027091 | 0.00476 | 1.30E-08 | 0.9959 | 0.405462 |
| Father's age at death | rs72738786 | 15:78828086_G_T | 0.662175 | 0.02756 | 0.004853 | 1.40E-08 | 0.99854 | 0.078995 |
| Father's age at death | rs10519203 | 15:78814046_G_A | 0.339877 | -0.02741 | 0.004844 | 1.50E-08 | 1 | 0.065524 |
| Father's age at death | rs1317286 | 15:78896129_A_G | 0.66338 | 0.027494 | 0.004856 | 1.50E-08 | 1 | 0.030726 |
| Father's age at death | rs375962788 | 15:78821143_C_CA | 0.675897 | 0.028249 | 0.005012 | 1.70E-08 | 0.95671 | 0.03207 |
| Father's age at death | rs56390833 | 15:78877381_C_A | 0.665573 | 0.027395 | 0.004866 | 1.80E-08 | 0.99979 | 0.017531 |
| Father's age at death | rs4887067 | 15:78886947_G_A | 0.665064 | 0.027367 | 0.004864 | 1.80E-08 | 0.99989 | 0.020249 |
| Father's age at death | rs17486195 | 15:78865197_A_G | 0.665428 | 0.027354 | 0.004866 | 1.90E-08 | 0.99952 | 0.014699 |
| Father's age at death | rs16969968 | 15:78882925_G_A | 0.665166 | 0.02733 | 0.004864 | 1.90E-08 | 1 | 0.021667 |
| Father's age at death | rs8192482 | 15:78886198_C_T | 0.665101 | 0.027342 | 0.004864 | 1.90E-08 | 0.9998 | 0.019893 |
| Father's age at death | rs149959208 | 15:78912710_T_TGCGCGGGGCAGGGCGACGGGCA | 0.661882 | 0.027324 | 0.004858 | 1.90E-08 | 0.9966 | 0.0264 |
| Father's age at death | rs951266 | 15:78878541_G_A | 0.665439 | 0.027318 | 0.004865 | 2.00E-08 | 1 | 0.016947 |
| Father's age at death | rs140330585 | 15:78866445_G_A | 0.665445 | 0.027256 | 0.004866 | 2.10E-08 | 0.99958 | 0.014434 |
| Father's age at death | rs56077333 | 15:78899003_C_A | 0.671474 | 0.027506 | 0.004913 | 2.20E-08 | 0.98869 | 0.020068 |
| Father's age at death | rs17486278 | 15:78867482_A_C | 0.665135 | 0.027146 | 0.004863 | 2.40E-08 | 1 | 0.01888 |
| Father's age at death | rs7180002 | 15:78873993_A_T | 0.665345 | 0.027148 | 0.004866 | 2.40E-08 | 1 | 0.009636 |
| Father's age at death | rs8042849 | 15:78817929_C_T | 0.345679 | -0.02689 | 0.004826 | 2.50E-08 | 0.99896 | 0.052966 |
| Father's age at death | rs72740964 | 15:78868636_G_A | 0.665539 | 0.027101 | 0.004871 | 2.60E-08 | 1 | 0.001201 |
| Father's age at death | rs55676755 | 15:78898932_C_G | 0.664706 | 0.027028 | 0.004862 | 2.70E-08 | 0.99969 | 0.029916 |
| Father's age at death | rs112878080 | 15:78900647_A_G | 0.66454 | 0.02703 | 0.004862 | 2.70E-08 | 0.99945 | 0.027133 |
| Father's age at death | rs138544659 | 15:78900701_T_G | 0.66454 | 0.02703 | 0.004862 | 2.70E-08 | 0.99945 | 0.027133 |
| Father's age at death | rs147144681 | 15:78900908_C_T | 0.664661 | 0.02703 | 0.004863 | 2.70E-08 | 0.99945 | 0.02896 |
| Father's age at death | rs113931022 | 15:78901113_C_T | 0.66454 | 0.02703 | 0.004862 | 2.70E-08 | 0.99945 | 0.027133 |
| Father's age at death | rs8031948 | 15:78816057_G_T | 0.66171 | 0.026955 | 0.004853 | 2.80E-08 | 0.9986 | 0.046111 |
| Father's age at death | rs1051730 | 15:78894339_G_A | 0.66462 | 0.026929 | 0.00486 | 3.00E-08 | 1 | 0.050338 |
| Father's age at death | rs146009840 | 15:78906177_A_T | 0.664836 | 0.026956 | 0.004863 | 3.00E-08 | 0.99945 | 0.028913 |
| Father's age at death | rs7172118 | 15:78862453_C_A | 0.665587 | 0.026987 | 0.004874 | 3.10E-08 | 0.99649 | 0.0139 |
| Father's age at death | rs34684276 | 15:78813155_G_A | 0.661692 | 0.026834 | 0.004853 | 3.20E-08 | 0.99828 | 0.051286 |
| Father's age at death | rs111704647 | 15:78900650_C_T | 0.664654 | 0.026902 | 0.004863 | 3.20E-08 | 0.99916 | 0.02896 |
| Father's age at death | rs11633958 | 15:78862064_C_T | 0.66574 | 0.026934 | 0.004875 | 3.30E-08 | 0.99648 | 0.013381 |
| Father's age at death | rs55958997 | 15:78915872_C_A | 0.643839 | 0.026736 | 0.004847 | 3.50E-08 | 0.97566 | 0.241379 |
| Father's age at death | rs8034191 | 15:78806023_T_C | 0.661205 | 0.026538 | 0.004848 | 4.40E-08 | 1 | 0.042218 |
| Father's age at death | . | 15:78859605_A_G | 0.660058 | 0.026185 | 0.004853 | 6.80E-08 | 0.82847 | 1 |
| Father's age at death | . | 15:78859605_AAAAAG_A | 0.660058 | 0.026185 | 0.004853 | 6.80E-08 | 0.9965 | 0.032004 |
| Father's age at death | rs58365910 | 15:78849034_T_C | 0.662887 | 0.026273 | 0.004878 | 7.20E-08 | 0.99043 | 0.047163 |
| Father's age at death | rs11852372 | 15:78801394_A_C | 0.663214 | 0.026246 | 0.004883 | 7.70E-08 | 0.98844 | 0.036108 |
| Father's age at death | rs2036527 | 15:78851615_G_A | 0.661827 | 0.02614 | 0.004864 | 7.70E-08 | 0.9947 | 0.047501 |
| Father's age at death | rs9788721 | 15:78802869_C_T | 0.344289 | -0.0258 | 0.004828 | 9.10E-08 | 1 | 0.032085 |
| Father's age at death | rs4533264 | 15:91431524_T_C | 0.648739 | -0.02587 | 0.004843 | 9.20E-08 | 0.97797 | 0.884453 |
| Father's age at death | rs72740955 | 15:78849779_C_T | 0.661622 | 0.025946 | 0.004858 | 9.30E-08 | 0.99657 | 0.047566 |
| Father's age at death | rs11331126 | 15:78850501_AT_A | 0.664109 | 0.026113 | 0.00489 | 9.30E-08 | 0.98735 | 0.019059 |
| Father's age at death | rs55853698 | 15:78857939_T_G | 0.660375 | 0.025723 | 0.004847 | 1.10E-07 | 1 | 0.031923 |
| Father's age at death | rs55781567 | 15:78857986_C_G | 0.660496 | 0.025713 | 0.004847 | 1.10E-07 | 1 | 0.035099 |
| Father's age at death | rs4932179 | 15:91430907_G_A | 0.650434 | -0.02576 | 0.004846 | 1.10E-07 | 0.97939 | 0.718141 |
| Father's age at death | rs4548831 | 15:91431470_G_A | 0.649802 | -0.02562 | 0.004843 | 1.20E-07 | 0.97952 | 0.819815 |
| Father's age at death | rs2071384 | 15:91430617_G_A | 0.650502 | -0.02555 | 0.004846 | 1.30E-07 | 0.97919 | 0.737123 |
| Father's age at death | rs113738722 | 15:91431807_G_GGGGCTGTTTT | 0.643494 | -0.02548 | 0.004824 | 1.30E-07 | 0.97824 | 0.90507 |
| Father's age at death | rs2677739 | 15:91441678_G_T | 0.649859 | -0.02546 | 0.004828 | 1.30E-07 | 0.98826 | 0.207883 |
| Father's age at death | rs4932374 | 15:91431255_G_A | 0.649847 | -0.02552 | 0.004843 | 1.40E-07 | 0.97951 | 0.775836 |
| Father's age at death | rs2677737 | 15:91441207_C_T | 0.649343 | -0.02533 | 0.004817 | 1.50E-07 | 0.99143 | 0.317665 |
| Father's age at death | rs8182016 | 15:91440926_G_C | 0.649185 | -0.02526 | 0.004818 | 1.60E-07 | 0.99119 | 0.285273 |
| Father's age at death | rs2521499 | 15:91441729_G_A | 0.650356 | -0.02529 | 0.004833 | 1.70E-07 | 0.98685 | 0.209855 |
| Father's age at death | rs2521500 | 15:91441383_A_G | 0.649676 | -0.02512 | 0.004819 | 1.80E-07 | 0.99157 | 0.276484 |
| Father's age at death | rs2677738 | 15:91441673_G_A | 0.649822 | -0.02494 | 0.004802 | 2.10E-07 | 1 | 0.094798 |
| Father's age at death | rs10744971 | 15:91443059_T_C | 0.615098 | -0.02466 | 0.004755 | 2.10E-07 | 0.97825 | 0.374542 |
| Father's age at death | rs199637917 | 11:33125029_G_GA | 0.985089 | 0.104012 | 0.020064 | 2.20E-07 | 0.88234 | 1 |
| Father's age at death | rs2869548 | 15:78922638_G_A | 0.646832 | 0.024856 | 0.004812 | 2.40E-07 | 0.99295 | 0.151266 |
| Father's age at death | rs17487223 | 15:78923987_C_T | 0.646812 | 0.024776 | 0.004814 | 2.70E-07 | 0.99196 | 0.154917 |
| Father's age at death | rs34903924 | 15:78804071_TA_T | 0.341146 | -0.02506 | 0.00489 | 3.00E-07 | 0.97927 | 0.052228 |
| Father's age at death | rs1029420 | 15:91441086_T_C | 0.383404 | 0.024118 | 0.004743 | 3.70E-07 | 0.98431 | 0.40059 |
| Father's age at death | rs4932376 | 15:91433265_C_G | 0.647692 | -0.02457 | 0.004839 | 3.80E-07 | 0.97876 | 0.79097 |
| Father's age at death | rs2521498 | 15:91442152_A_T | 0.385089 | 0.024063 | 0.00475 | 4.10E-07 | 0.98012 | 0.333588 |
| Father's age at death | rs72743158 | 15:78926445_T_C | 0.638241 | 0.024256 | 0.004795 | 4.20E-07 | 0.98872 | 0.317044 |
| Father's age at death | rs62405340 | 6:20936555_C_T | 0.879237 | 0.03622 | 0.00716 | 4.20E-07 | 0.95553 | 0.050513 |
| Father's age at death | rs72905002 | 11:33142577_C_T | 0.985487 | 0.101485 | 0.020311 | 5.80E-07 | 0.88398 | 0.838641 |
| Father's age at death | rs116764587 | 3:32934992_A_G | 0.986063 | 0.104917 | 0.02106 | 6.30E-07 | 0.86189 | 0.835817 |
| Father's age at death | rs8192477 | 15:78910463_G_C | 0.72246 | -0.02542 | 0.005114 | 6.70E-07 | 0.99859 | 0.880134 |
| Father's age at death | rs146873082 | 2:128650488_T_C | 0.98639 | -0.10274 | 0.020696 | 6.90E-07 | 0.90449 | 0.137208 |
| Father's age at death | rs11638635 | 15:91435516_C_G | 0.629352 | -0.0239 | 0.004817 | 7.00E-07 | 0.96866 | 0.285509 |
| Father's age at death | rs8192478 | 15:78910462_A_G | 0.723235 | -0.02545 | 0.005153 | 7.90E-07 | 0.98548 | 0.773517 |
| Father's age at death | rs12909124 | 15:91435422_G_A | 0.629563 | -0.02376 | 0.004817 | 8.10E-07 | 0.96885 | 0.279853 |
| Father's age at death | rs143557209 | 11:33318846_G_A | 0.986943 | 0.109297 | 0.022179 | 8.30E-07 | 0.81982 | 0.260531 |
| Father's age at death | rs6807140 | 3:4936864_A_G | 0.452525 | 0.022954 | 0.004668 | 8.80E-07 | 0.96282 | 0.213118 |
| Father's age at death | rs11970030 | 6:20930113_G_C | 0.874651 | 0.034277 | 0.007019 | 1.00E-06 | 0.9622 | 0.021503 |
| Father's age at death | rs76658847 | 9:91203980_G_T | 0.952751 | 0.053302 | 0.010974 | 1.20E-06 | 0.96119 | 0.67459 |
| Father's age at death | rs201522476 | 15:78828640_TACAC_T | 0.454234 | -0.02386 | 0.004911 | 1.20E-06 | 0.88043 | 0.086099 |
| Father's age at death | rs563997271 | 9:75738856_A_G | 0.988626 | 0.124237 | 0.025635 | 1.30E-06 | 0.70726 | 1 |
| Father's age at death | rs62405352 | 6:20968951_A_G | 0.877078 | 0.034243 | 0.00708 | 1.30E-06 | 0.96315 | 0.102624 |
| Father's age at death | rs9358378 | 6:20927156_T_A | 0.885595 | 0.036449 | 0.007564 | 1.40E-06 | 0.89879 | 0.176718 |
| Father's age at death | rs4307160 | 6:20942196_T_C | 0.879061 | 0.034284 | 0.007111 | 1.40E-06 | 0.96786 | 0.075666 |
| Father's age at death | rs992567 | 10:36548567_C_G | 0.0400584 | 0.056171 | 0.011647 | 1.40E-06 | 1 | 0.85105 |
| Father's age at death | rs1219587 | 10:36548695_G_A | 0.0394574 | 0.056701 | 0.011745 | 1.40E-06 | 0.99822 | 0.969592 |
| Father's age at death | rs1219599 | 10:36545612_A_T | 0.0395266 | 0.056559 | 0.011752 | 1.50E-06 | 0.99529 | 0.939306 |
| Father's age at death | rs979288 | 10:36545269_G_A | 0.0394171 | 0.056357 | 0.011753 | 1.60E-06 | 0.99764 | 0.939146 |
| Father's age at death | rs979287 | 10:36545321_T_C | 0.0394038 | 0.056454 | 0.011757 | 1.60E-06 | 0.99729 | 0.908841 |
| Father's age at death | rs1219597 | 10:36546115_C_T | 0.0394231 | 0.056401 | 0.011751 | 1.60E-06 | 0.99777 | 0.939154 |
| Father's age at death | rs1219592 | 10:36547655_A_T | 0.0394222 | 0.056395 | 0.011751 | 1.60E-06 | 0.99785 | 0.939161 |
| Father's age at death | rs1219591 | 10:36547975_G_A | 0.0394222 | 0.056395 | 0.011751 | 1.60E-06 | 0.99785 | 0.939161 |
| Father's age at death | rs1219590 | 10:36548072_C_A | 0.0394383 | 0.05646 | 0.011751 | 1.60E-06 | 0.99746 | 0.9392 |
| Father's age at death | rs16884302 | 6:20926317_A_T | 0.873896 | 0.033808 | 0.007037 | 1.60E-06 | 0.95235 | 0.020668 |
| Father's age at death | rs62405341 | 6:20954580_C_T | 0.878699 | 0.034097 | 0.007099 | 1.60E-06 | 0.9683 | 0.067811 |
| Father's age at death | rs11961135 | 6:20982605_G_A | 0.87913 | 0.034168 | 0.007114 | 1.60E-06 | 0.96741 | 0.069056 |
| Father's age at death | rs56069995 | 6:87713256_CA_C | 0.0520442 | 0.054997 | 0.011455 | 1.60E-06 | 0.79914 | 0.015378 |
| Father's age at death | rs9266141 | 6:31324023_C_G | 0.763029 | 0.02731 | 0.005701 | 1.70E-06 | 0.89186 | 0.917293 |
| Father's age at death | rs189267161 | 3:49776818_G_A | 0.96348 | -0.0593 | 0.012398 | 1.70E-06 | 0.96321 | 0.232252 |
| Father's age at death | rs375021466 | 15:78867314_T_TA | 0.741067 | 0.026211 | 0.005479 | 1.70E-06 | 0.91332 | 0.154533 |
| Father's age at death | rs11539637 | 15:91428290_C_T | 0.4765 | -0.02193 | 0.004594 | 1.80E-06 | 0.9953 | 0.373379 |
| Father's age at death | rs6940952 | 6:20964315_A_G | 0.878225 | 0.03384 | 0.007091 | 1.80E-06 | 0.96744 | 0.06094 |
| Father's age at death | rs11966306 | 6:20964849_C_T | 0.879762 | 0.034063 | 0.007152 | 1.90E-06 | 0.96122 | 0.060076 |
| Father's age at death | rs7177338 | 15:91428636_G_A | 0.477157 | -0.0219 | 0.004598 | 1.90E-06 | 0.99419 | 0.300898 |
| Father's age at death | rs2071382 | 15:91428197_T_C | 0.475882 | -0.02183 | 0.004595 | 2.00E-06 | 0.99526 | 0.328587 |
| Father's age at death | rs540932416 | 6:13739700_T_TAC | 0.937499 | -0.04495 | 0.009458 | 2.00E-06 | 0.9893 | 0.000341 |
| Father's age at death | rs187652687 | 3:49282865_C_T | 0.96291 | -0.05835 | 0.012275 | 2.00E-06 | 0.96878 | 0.255929 |
| Father's age at death | rs55980841 | 20:2213737_G_A | 0.655165 | -0.02339 | 0.004933 | 2.10E-06 | 0.96234 | 0.097387 |
| Father's age at death | rs1219595 | 10:36546690_T_A | 0.0393633 | 0.05584 | 0.011763 | 2.10E-06 | 0.99718 | 0.90872 |
| Father's age at death | rs72748240 | 9:80040835_C_A | 0.909269 | 0.037851 | 0.007975 | 2.10E-06 | 0.99608 | 0.701262 |
| Father's age at death | rs7183988 | 15:91428589_T_G | 0.476785 | -0.02174 | 0.004596 | 2.20E-06 | 0.9949 | 0.32867 |
| Father's age at death | rs1894401 | 15:91429042_G_A | 0.476488 | -0.02176 | 0.004596 | 2.20E-06 | 0.9954 | 0.311767 |
| Father's age at death | rs518425 | 15:78883813_A_G | 0.717789 | -0.0241 | 0.005091 | 2.20E-06 | 0.99808 | 0.64408 |
| Father's age at death | rs56320800 | 6:13718569_T_A | 0.938098 | -0.04486 | 0.009475 | 2.20E-06 | 0.99563 | 0.001542 |
| Father's age at death | rs6905662 | 6:13727788_T_C | 0.937875 | -0.0448 | 0.00946 | 2.20E-06 | 0.99531 | 0.00113 |
| Father's age at death | rs75648813 | 6:13738507_T_G | 0.937694 | -0.0447 | 0.009468 | 2.30E-06 | 0.99053 | 0.000627 |
| Father's age at death | rs55810540 | 6:13743913_A_G | 0.938263 | -0.04498 | 0.009527 | 2.30E-06 | 0.98663 | 0.000466 |
| Father's age at death | rs149908616 | 3:49317988_G_T | 0.963638 | -0.05879 | 0.012438 | 2.30E-06 | 0.96189 | 0.230696 |
| Father's age at death | rs550336358 | 2:128607895_T_C | 0.985916 | -0.09318 | 0.019761 | 2.40E-06 | 0.95973 | 0.261078 |
| Father's age at death | rs140666829 | 15:53704081_T_C | 0.973413 | 0.070815 | 0.015019 | 2.40E-06 | 0.89699 | 0.546387 |
| Father's age at death | rs71713299 | 13:86385321_C_CA | 0.707671 | -0.02428 | 0.005161 | 2.50E-06 | 0.94371 | 0.175142 |
| Father's age at death | rs76062289 | 6:13729392_C_T | 0.937711 | -0.04446 | 0.009453 | 2.60E-06 | 0.99442 | 0.001068 |
| Father's age at death | rs76856459 | 6:13729394_C_T | 0.937711 | -0.04446 | 0.009453 | 2.60E-06 | 0.99442 | 0.001068 |
| Father's age at death | rs142359393 | 6:13741337_C_A | 0.937709 | -0.04457 | 0.009476 | 2.60E-06 | 0.98911 | 0.000623 |
| Father's age at death | rs55920516 | 20:2213746_A_T | 0.654949 | -0.0232 | 0.004932 | 2.60E-06 | 0.96238 | 0.106854 |
| Father's age at death | rs12528373 | 6:13718121_A_G | 0.937971 | -0.04438 | 0.009461 | 2.70E-06 | 0.99692 | 0.001571 |
| Father's age at death | rs199642525 | 15:78901173_GA_G | 0.786292 | 0.029171 | 0.006214 | 2.70E-06 | 0.80745 | 0.870224 |
| Father's age at death | rs146023589 | 9:80023931_C_A | 0.909692 | 0.037495 | 0.007986 | 2.70E-06 | 0.99806 | 0.792812 |
| Father's age at death | rs72744286 | 9:79919444_G_A | 0.908626 | 0.03719 | 0.007937 | 2.80E-06 | 1 | 0.781646 |
| Father's age at death | rs77649360 | 6:13715999_G_C | 0.937945 | -0.04433 | 0.009463 | 2.80E-06 | 0.99601 | 0.001579 |
| Father's age at death | rs34618730 | 6:13745594_TTG_T | 0.937651 | -0.04447 | 0.009497 | 2.80E-06 | 0.98385 | 0.000574 |
| Father's age at death | rs72843393 | 2:128417583_A_G | 0.985611 | -0.09122 | 0.019491 | 2.90E-06 | 0.96727 | 0.616042 |
| Father's age at death | rs7860643 | 9:9910431_T_C | 0.679152 | -0.02299 | 0.004914 | 2.90E-06 | 0.99023 | 0.525618 |
| Father's age at death | rs17340954 | 9:80032988_T_A | 0.909375 | 0.037227 | 0.007974 | 3.00E-06 | 0.9975 | 0.713954 |
| Father's age at death | rs114205165 | 3:49807614_C_G | 0.963773 | -0.05865 | 0.012563 | 3.00E-06 | 0.94646 | 0.455145 |
| Father's age at death | rs12524913 | 6:13745214_T_C | 0.937661 | -0.04435 | 0.009497 | 3.00E-06 | 0.98391 | 0.000572 |
| Father's age at death | rs72746010 | 9:79942402_A_G | 0.909103 | 0.037162 | 0.007964 | 3.10E-06 | 0.99788 | 0.780675 |
| Father's age at death | rs58845081 | 13:25785941_T_C | 0.926651 | 0.041925 | 0.00899 | 3.10E-06 | 0.95161 | 0.308234 |
| Father's age at death | rs10759102 | 9:9910123_G_A | 0.679275 | -0.0228 | 0.004894 | 3.20E-06 | 1 | 1 |
| Father's age at death | rs12479920 | 20:2205978_T_C | 0.663297 | -0.02274 | 0.004884 | 3.20E-06 | 0.99343 | 0.056247 |
| Father's age at death | rs12661776 | 6:13744533_G_A | 0.93775 | -0.0442 | 0.009502 | 3.30E-06 | 0.98435 | 0.000738 |
| Father's age at death | rs17797976 | 6:13744794_C_T | 0.937634 | -0.04415 | 0.009495 | 3.30E-06 | 0.98428 | 0.000689 |
| Father's age at death | rs75300520 | 6:13744942_G_A | 0.937692 | -0.04419 | 0.009499 | 3.30E-06 | 0.98414 | 0.000623 |
| Father's age at death | rs7758467 | 6:20901267_T_C | 0.874979 | 0.032772 | 0.007046 | 3.30E-06 | 0.95885 | 0.11723 |
| Father's age at death | rs950387 | 5:81940028_C_G | 0.264941 | 0.024275 | 0.005216 | 3.30E-06 | 0.98753 | 0.970461 |
| Father's age at death | rs146573576 | 9:80028799_A_C | 0.909116 | 0.037049 | 0.007971 | 3.30E-06 | 0.99644 | 0.767308 |
| Father's age at death | rs199764931 | 3:49561065_ACCCC_A | 0.964159 | -0.05867 | 0.012639 | 3.40E-06 | 0.94447 | 0.275429 |
| Father's age at death | rs6082442 | 20:2205138_C_T | 0.660817 | -0.02254 | 0.004859 | 3.50E-06 | 1 | 0.067952 |
| Father's age at death | rs72746025 | 9:79964951_G_T | 0.908854 | 0.037006 | 0.00798 | 3.50E-06 | 0.99147 | 0.626816 |
| Father's age at death | rs36122798 | 9:9909462_GA_G | 0.678982 | -0.0227 | 0.004895 | 3.50E-06 | 0.99739 | 0.504359 |
| Father's age at death | rs66926624 | 9:79924574_T_G | 0.908735 | 0.03679 | 0.007941 | 3.60E-06 | 0.99977 | 0.715764 |
| Father's age at death | rs113047626 | 6:13715947_G_A | 0.938086 | -0.04388 | 0.009476 | 3.60E-06 | 0.99536 | 0.001295 |
| Father's age at death | rs150426469 | 3:49079495_C_T | 0.962544 | -0.05696 | 0.012311 | 3.70E-06 | 0.95347 | 0.228155 |
| Father's age at death | rs150525910 | 3:49451639_A_G | 0.963006 | -0.0568 | 0.012268 | 3.70E-06 | 0.9726 | 0.290262 |
| Father's age at death | rs72744288 | 9:79922010_G_A | 0.908915 | 0.036783 | 0.007962 | 3.80E-06 | 0.99574 | 0.626773 |
| Father's age at death | rs60329496 | 9:80003450_AAC_A | 0.908419 | 0.036697 | 0.007941 | 3.80E-06 | 0.99678 | 0.71649 |
| Father's age at death | rs41289983 | 9:80022280_T_C | 0.908794 | 0.036764 | 0.007952 | 3.80E-06 | 0.99784 | 0.741652 |
| Father's age at death | rs17385118 | 10:8455084_T_C | 0.891329 | -0.0339 | 0.007335 | 3.80E-06 | 1 | 0.436415 |
| Father's age at death | rs564585 | 15:78886227_A_G | 0.723185 | -0.02366 | 0.005118 | 3.80E-06 | 0.99922 | 0.703089 |
| Father's age at death | rs4728017 | 7:79766397_G_A | 0.794659 | 0.026749 | 0.005784 | 3.80E-06 | 0.95689 | 0.565588 |
| Father's age at death | rs2008021 | 5:81939689_C_T | 0.254684 | 0.024364 | 0.00528 | 3.90E-06 | 0.98822 | 0.987872 |
| Father's age at death | rs287230 | 1:55660292_G_C | 0.876128 | -0.03233 | 0.007003 | 3.90E-06 | 0.98107 | 0.660055 |
| Father's age at death | rs1123033 | 1:201536347_G_C | 0.957966 | 0.052864 | 0.011445 | 3.90E-06 | 0.99374 | 0.351844 |
| Father's age at death | rs6082441 | 20:2205092_C_T | 0.660811 | -0.02244 | 0.00486 | 3.90E-06 | 0.99964 | 0.060562 |
| Father's age at death | rs72748232 | 9:80021100_T_C | 0.908687 | 0.036673 | 0.007945 | 3.90E-06 | 0.99853 | 0.755023 |
| Father's age at death | rs72744287 | 9:79920830_T_C | 0.908767 | 0.036624 | 0.007942 | 4.00E-06 | 0.99983 | 0.715715 |
| Father's age at death | rs150916776 | 9:79920961_CTTA_C | 0.90876 | 0.036623 | 0.007942 | 4.00E-06 | 0.99974 | 0.728669 |
| Father's age at death | rs72744291 | 9:79922133_C_T | 0.908785 | 0.036638 | 0.007943 | 4.00E-06 | 0.9998 | 0.715649 |
| Father's age at death | rs2889772 | 9:79937117_T_G | 0.908724 | 0.036618 | 0.007942 | 4.00E-06 | 0.99956 | 0.741805 |
| Father's age at death | rs139375017 | 9:80014019_C_A | 0.908742 | 0.03664 | 0.007944 | 4.00E-06 | 0.99913 | 0.754901 |
| Father's age at death | rs56074928 | 9:80014869_A_G | 0.908726 | 0.036652 | 0.007944 | 4.00E-06 | 0.99903 | 0.741805 |
| Father's age at death | rs187222318 | 9:80027093_T_C | 0.908966 | 0.036707 | 0.007963 | 4.00E-06 | 0.99667 | 0.741225 |
| Father's age at death | rs17469355 | 1:201536120_A_G | 0.95814 | 0.052727 | 0.011436 | 4.00E-06 | 1 | 0.28053 |
| Father's age at death | rs287227 | 1:55656075_G_T | 0.876129 | -0.03228 | 0.007003 | 4.00E-06 | 0.9811 | 0.66004 |
| Father's age at death | rs186360436 | 9:79919110_T_G | 0.908762 | 0.036602 | 0.007943 | 4.10E-06 | 0.99957 | 0.728669 |
| Father's age at death | rs17340309 | 9:80006305_T_C | 0.908657 | 0.036567 | 0.007942 | 4.10E-06 | 0.99862 | 0.690224 |
| Father's age at death | rs72748228 | 9:80006365_A_G | 0.908657 | 0.036567 | 0.007942 | 4.10E-06 | 0.99862 | 0.690224 |
| Father's age at death | rs76780136 | 3:72208640_G_A | 0.973155 | 0.066166 | 0.014371 | 4.10E-06 | 0.96832 | 0.696691 |
| Father's age at death | rs72744282 | 9:79911079_A_G | 0.908745 | 0.036553 | 0.007942 | 4.20E-06 | 0.99957 | 0.728702 |
| Father's age at death | rs17423381 | 9:79917660_C_G | 0.908743 | 0.036548 | 0.007942 | 4.20E-06 | 0.99956 | 0.728719 |
| Father's age at death | rs17339033 | 9:79929754_T_G | 0.908765 | 0.036524 | 0.007942 | 4.20E-06 | 0.99982 | 0.715665 |
| Father's age at death | rs55973302 | 9:79916494_A_G | 0.908592 | 0.036501 | 0.00794 | 4.30E-06 | 0.99893 | 0.781682 |
| Father's age at death | rs72746090 | 9:79974824_T_C | 0.90933 | 0.036754 | 0.007993 | 4.30E-06 | 0.99282 | 0.675445 |
| Father's age at death | rs113346504 | 9:80009644_T_G | 0.908648 | 0.036501 | 0.007942 | 4.30E-06 | 0.99858 | 0.703056 |
| Father's age at death | rs321472 | 16:26745376_G_A | 0.349806 | -0.02215 | 0.00482 | 4.30E-06 | 0.98707 | 0.20239 |
| Father's age at death | rs6861773 | 5:81937225_A_G | 0.221836 | 0.025371 | 0.005518 | 4.30E-06 | 0.99319 | 0.482292 |
| Father's age at death | rs112317830 | 9:79925892_A_C | 0.909189 | 0.036575 | 0.007967 | 4.40E-06 | 0.99771 | 0.701698 |
| Father's age at death | rs17423984 | 9:79936415_A_G | 0.908823 | 0.036486 | 0.007947 | 4.40E-06 | 0.99915 | 0.728518 |
| Father's age at death | rs17340234 | 9:79984434_A_G | 0.908657 | 0.036484 | 0.007942 | 4.40E-06 | 0.99872 | 0.690224 |
| Father's age at death | rs72748207 | 9:79993672_T_C | 0.908499 | 0.036468 | 0.007945 | 4.40E-06 | 0.99635 | 0.703493 |
| Father's age at death | rs140320210 | 9:80024405_T_C | 0.908757 | 0.036479 | 0.007951 | 4.50E-06 | 0.99773 | 0.715715 |
| Father's age at death | rs11372849 | 15:91419432_T_TC | 0.5205 | 0.021119 | 0.004605 | 4.50E-06 | 0.99151 | 0.231761 |
| Father's age at death | rs72744289 | 9:79922022_G_C | 0.909101 | 0.036455 | 0.007966 | 4.70E-06 | 0.99696 | 0.689001 |
| Father's age at death | rs6804429 | 3:4939354_C_T | 0.404981 | 0.021691 | 0.004737 | 4.70E-06 | 0.96702 | 0.900057 |
| Father's age at death | rs72746028 | 9:79969947_T_C | 0.908551 | 0.03633 | 0.007941 | 4.80E-06 | 0.99796 | 0.729172 |
| Father's age at death | rs72746098 | 9:79983279_G_A | 0.908652 | 0.036333 | 0.007943 | 4.80E-06 | 0.99837 | 0.703008 |
| Father's age at death | . | 15:91412849_C_CCAAAGGCA | 0.527311 | 0.021249 | 0.004646 | 4.80E-06 | 0.97351 | 0.559088 |
| Father's age at death | rs578776 | 15:78888400_G_A | 0.72258 | -0.02337 | 0.005112 | 4.80E-06 | 1 | 0.703493 |
| Father's age at death | rs504156 | 1:201533736_G_A | 0.958108 | 0.052328 | 0.011457 | 4.90E-06 | 0.99518 | 0.297461 |
| Father's age at death | rs17202631 | 2:112444132_C_T | 0.969182 | 0.060611 | 0.013265 | 4.90E-06 | 0.99055 | 0.664462 |
| Father's age at death | rs72746029 | 9:79970505_T_G | 0.908742 | 0.036252 | 0.007945 | 5.00E-06 | 0.99882 | 0.702797 |
| Father's age at death | rs72748204 | 9:79990049_A_T | 0.908649 | 0.036243 | 0.00794 | 5.00E-06 | 0.99905 | 0.690224 |
| Father's age at death | rs7871708 | 9:9910425_C_T | 0.679641 | -0.02244 | 0.004915 | 5.00E-06 | 0.99059 | 0.520991 |
| Father's age at death | rs17340032 | 9:79975040_A_G | 0.908731 | 0.036222 | 0.007945 | 5.10E-06 | 0.99877 | 0.70283 |
| Father's age at death | rs10491843 | 9:79980974_A_C | 0.908681 | 0.036219 | 0.007944 | 5.10E-06 | 0.99857 | 0.677439 |
| Father's age at death | rs138174176 | 9:79983426_A_AT | 0.908883 | 0.036311 | 0.007958 | 5.10E-06 | 0.9968 | 0.689477 |
| Father's age at death | rs72746026 | 9:79969798_A_T | 0.908752 | 0.036208 | 0.007946 | 5.20E-06 | 0.99883 | 0.702765 |
| Father's age at death | rs17340192 | 9:79984232_T_C | 0.90868 | 0.036212 | 0.007944 | 5.20E-06 | 0.99856 | 0.677439 |
| Father's age at death | rs73733101 | 6:20832891_A_C | 0.891674 | 0.033684 | 0.007401 | 5.30E-06 | 0.98713 | 0.676317 |
| Father's age at death | rs6663252 | 1:55630151_T_C | 0.874443 | -0.03165 | 0.006954 | 5.30E-06 | 0.98367 | 0.663682 |
| Father's age at death | rs10054026 | 5:81925926_A_T | 0.254442 | 0.023974 | 0.00527 | 5.40E-06 | 0.99248 | 0.89714 |
| Father's age at death | rs17080898 | 4:32194941_A_G | 0.913536 | -0.03713 | 0.008175 | 5.60E-06 | 0.99439 | 0.51331 |
| Father's age at death | rs12054570 | 4:32208145_T_G | 0.913904 | -0.03711 | 0.00818 | 5.70E-06 | 0.99708 | 0.511434 |
| Father's age at death | rs72746094 | 9:79983083_G_C | 0.909435 | 0.036261 | 0.007991 | 5.70E-06 | 0.99426 | 0.82038 |
| Father's age at death | rs141237998 | 15:53689480_T_C | 0.969961 | 0.063215 | 0.013935 | 5.70E-06 | 0.92454 | 0.465372 |
| Father's age at death | rs544330253 | 9:79992257_T_TC | 0.908775 | 0.036049 | 0.007952 | 5.80E-06 | 0.99759 | 0.754797 |
| Father's age at death | rs73126288 | 4:32205108_G_A | 0.913896 | -0.03709 | 0.00818 | 5.80E-06 | 0.99716 | 0.49985 |
| Father's age at death | rs73126294 | 4:32206428_G_A | 0.913895 | -0.03709 | 0.00818 | 5.80E-06 | 0.99717 | 0.49985 |
| Father's age at death | rs112220806 | 4:32206864_A_ACAACTT | 0.913879 | -0.03709 | 0.008179 | 5.80E-06 | 0.99717 | 0.547356 |
| Father's age at death | rs62398109 | 6:20881816_T_C | 0.875017 | 0.031885 | 0.007036 | 5.90E-06 | 0.96351 | 0.263106 |
| Father's age at death | . | 10:98684132_TTTTGTTTG_T | 0.98964 | -0.13116 | 0.028982 | 6.00E-06 | 0.60559 | 0.674311 |
| Father's age at death | rs72746091 | 9:79977921_C_T | 0.908765 | 0.035938 | 0.007947 | 6.10E-06 | 0.99863 | 0.689906 |
| Father's age at death | rs56779627 | 2:61844444_CA_C | 0.282112 | -0.02378 | 0.005258 | 6.10E-06 | 0.93651 | 0.237405 |
| Father's age at death | rs17080919 | 4:32201920_C_T | 0.913941 | -0.03702 | 0.008182 | 6.10E-06 | 0.9972 | 0.511234 |
| Father's age at death | rs72832363 | 6:20822865_A_G | 0.890432 | 0.033259 | 0.007357 | 6.20E-06 | 0.98874 | 0.497565 |
| Father's age at death | rs6931788 | 6:20821841_G_C | 0.890435 | 0.033223 | 0.007359 | 6.30E-06 | 0.98811 | 0.479104 |
| Father's age at death | . | 2:112427172_G_GT | 0.968682 | 0.060242 | 0.013338 | 6.30E-06 | 0.96531 | 0.634819 |
| Father's age at death | . | 2:112427172_G_GTTTTTT | 0.968682 | 0.060242 | 0.013338 | 6.30E-06 | 0.72393 | 0.315595 |
| Father's age at death | rs17064378 | 18:55786359_C_T | 0.438802 | 0.021058 | 0.004664 | 6.30E-06 | 0.97341 | 0.16167 |
| Father's age at death | rs73128212 | 4:32210014_G_T | 0.913992 | -0.03702 | 0.008194 | 6.30E-06 | 0.99472 | 0.522902 |
| Father's age at death | rs138025226 | 4:45750383_T_C | 0.984935 | -0.08851 | 0.019593 | 6.30E-06 | 0.92194 | 0.619013 |
| Father's age at death | rs6452468 | 5:81928659_T_A | 0.25465 | 0.023759 | 0.005261 | 6.30E-06 | 0.99526 | 0.891167 |
| Father's age at death | rs6900509 | 6:20823848_A_G | 0.890442 | 0.033215 | 0.007362 | 6.40E-06 | 0.98728 | 0.469916 |
| Father's age at death | rs6452469 | 5:81928819_T_C | 0.22111 | 0.024901 | 0.005523 | 6.50E-06 | 0.99419 | 0.562937 |
| Father's age at death | rs4593264 | 5:124047014_A_C | 0.0132162 | -0.09045 | 0.020053 | 6.50E-06 | 1 | 0.370766 |
| Father's age at death | rs71148543 | 15:78888782_AT_A | 0.705328 | -0.02298 | 0.005094 | 6.50E-06 | 0.97259 | 0.589074 |
| Father's age at death | rs72748205 | 9:79992002_C_T | 0.908761 | 0.035825 | 0.007949 | 6.60E-06 | 0.99801 | 0.664523 |
| Father's age at death | rs114646143 | 3:53685498_G_A | 0.988714 | -0.09924 | 0.02203 | 6.60E-06 | 0.96089 | 0.184506 |
| Father's age at death | rs73126229 | 4:32200539_A_T | 0.914243 | -0.03689 | 0.008198 | 6.80E-06 | 0.99608 | 0.558085 |
| Father's age at death | rs12082008 | 1:66008710_G_T | 0.678043 | -0.02273 | 0.005053 | 6.80E-06 | 0.93839 | 0.848207 |
| Father's age at death | rs55964802 | 1:66010433_G_A | 0.678058 | -0.02276 | 0.005057 | 6.80E-06 | 0.937 | 0.843032 |
| Father's age at death | rs74514669 | 9:4015213_G_A | 0.988426 | 0.099733 | 0.022178 | 6.90E-06 | 0.92646 | 0.708794 |
| Father's age at death | rs57515981 | 15:91412850_A_AAAGGCAG | 0.520442 | 0.020746 | 0.004616 | 7.00E-06 | 0.98414 | 0.517655 |
| Father's age at death | rs17385180 | 10:8455749_G_T | 0.891376 | -0.03308 | 0.007359 | 7.00E-06 | 0.99401 | 0.481781 |
| Father's age at death | rs73122330 | 4:32178345_A_G | 0.912986 | -0.03658 | 0.008138 | 7.00E-06 | 0.99747 | 0.385771 |
| Father's age at death | rs146415646 | 1:99424562_AC_A | 0.889895 | 0.033674 | 0.007499 | 7.10E-06 | 0.94755 | 0.837259 |
| Father's age at death | rs1847040 | 5:81938623_A_C | 0.220918 | 0.024843 | 0.005531 | 7.10E-06 | 0.99187 | 0.579776 |
| Father's age at death | rs12087382 | 1:66008702_T_A | 0.680084 | -0.0228 | 0.005082 | 7.20E-06 | 0.93069 | 0.905111 |
| Father's age at death | rs1317305 | 9:79910407_A_G | 0.911894 | 0.036589 | 0.008151 | 7.20E-06 | 0.97944 | 0.628825 |
| Father's age at death | rs55882247 | 9:79946618_A_G | 0.908474 | 0.035612 | 0.007935 | 7.20E-06 | 0.99874 | 0.628131 |
| Father's age at death | rs8039305 | 15:91422543_T_C | 0.520847 | 0.020664 | 0.004606 | 7.20E-06 | 0.99158 | 0.272345 |
| Father's age at death | rs576796248 | 2:129160087_G_A | 0.986 | -0.0897 | 0.019983 | 7.20E-06 | 0.94207 | 0.212838 |
| Father's age at death | . | 17:46697690_C_T | 0.0180226 | 0.076792 | 0.017126 | 7.30E-06 | 0.93572 | 0.34015 |
| Father's age at death | . | rs11417933 | 0.0180226 | 0.076792 | 0.017126 | 7.30E-06 | 1 | 0.135866 |
| Father's age at death | rs60804467 | 15:99488091_C_T | 0.783343 | 0.025014 | 0.005584 | 7.50E-06 | 0.98938 | 0.918608 |
| Father's age at death | rs55735303 | 9:79950784_A_T | 0.90853 | 0.035546 | 0.00794 | 7.60E-06 | 0.99815 | 0.640251 |
| Father's age at death | rs72848814 | 2:128589164_C_T | 0.986447 | -0.09142 | 0.020428 | 7.60E-06 | 0.93211 | 0.457783 |
| Father's age at death | rs4815058 | 20:2214141_T_G | 0.660798 | -0.02201 | 0.004919 | 7.60E-06 | 0.97627 | 0.055475 |
| Father's age at death | rs35987145 | 18:55786217_G_GC | 0.438675 | 0.020901 | 0.004674 | 7.70E-06 | 0.96982 | 0.205935 |
| Father's age at death | rs60342931 | 6:20810101_T_C | 0.890911 | 0.032942 | 0.007369 | 7.80E-06 | 0.9896 | 0.52453 |
| Father's age at death | rs28542200 | 15:78184496_C_T | 0.858498 | -0.02934 | 0.006572 | 8.00E-06 | 0.99186 | 0.325267 |
| Father's age at death | rs72746024 | 9:79963881_A_G | 0.90849 | 0.035418 | 0.00794 | 8.20E-06 | 0.9978 | 0.615857 |
| Father's age at death | rs12481003 | 20:2214087_T_A | 0.654863 | -0.02185 | 0.0049 | 8.20E-06 | 0.97501 | 0.08529 |
| Father's age at death | rs142773590 | 4:19826923_G_A | 0.989476 | -0.10656 | 0.023906 | 8.30E-06 | 0.87919 | 0.580644 |
| Father's age at death | rs11664018 | 18:55786208_G_C | 0.437464 | 0.020778 | 0.004666 | 8.40E-06 | 0.97357 | 0.179664 |
| Father's age at death | rs72743868 | 5:40325599_G_T | 0.989385 | 0.123522 | 0.027779 | 8.70E-06 | 0.64644 | 0.492754 |
| Father's age at death | rs72841716 | 2:128744480_T_C | 0.986033 | -0.08653 | 0.019466 | 8.80E-06 | 1 | 0.536364 |
| Father's age at death | rs4254287 | 15:78170040_G_A | 0.139903 | 0.029547 | 0.006651 | 8.90E-06 | 0.97845 | 0.606876 |
| Father's age at death | rs7656079 | 4:32183956_A_G | 0.913166 | -0.0362 | 0.008156 | 9.10E-06 | 0.99495 | 0.394939 |
| Father's age at death | rs6827447 | 4:32177890_G_A | 0.913341 | -0.03616 | 0.00815 | 9.20E-06 | 0.99791 | 0.457172 |
| Father's age at death | rs73128299 | 4:32217515_G_A | 0.913953 | -0.03642 | 0.008213 | 9.20E-06 | 0.9904 | 0.38104 |
| Father's age at death | rs149279096 | 13:56274993_T_C | 0.830895 | 0.02829 | 0.006379 | 9.20E-06 | 0.91239 | 0.268968 |
| Father's age at death | rs6941522 | 6:13655339_A_G | 0.938585 | -0.04227 | 0.009531 | 9.20E-06 | 0.99176 | 0.003044 |
| Father's age at death | rs114824772 | 4:45773639_T_G | 0.985287 | -0.0879 | 0.019853 | 9.50E-06 | 0.9183 | 0.838154 |
| Father's age at death | rs11858836 | 15:78783277_G_A | 0.67605 | 0.021544 | 0.004869 | 9.60E-06 | 1 | 0.008682 |
| Father's age at death | rs73122314 | 4:32171701_G_A | 0.913308 | -0.03607 | 0.008156 | 9.80E-06 | 0.99636 | 0.457362 |
| Father's age at death | rs9547380 | 13:86375448_A_T | 0.742424 | -0.02341 | 0.005296 | 9.90E-06 | 0.97075 | 0.309913 |
| Father's age at death | rs11663320 | 18:55786131_G_T | 0.440286 | 0.020597 | 0.004664 | 1.00E-05 | 0.97331 | 0.221366 |
| Father's age at death | rs55977216 | 6:13653508_T_C | 0.938951 | -0.04219 | 0.009568 | 1.00E-05 | 0.99035 | 0.006333 |
| Father's age at death | rs537535711 | 2:87794572_C_T | 0.988149 | -0.1371 | 0.031054 | 1.00E-05 | 0.46166 | 0.710412 |
| Father's age at death | rs7686517 | 4:32162926_T_C | 0.912978 | -0.03593 | 0.008148 | 1.00E-05 | 0.99461 | 0.44792 |
| Father's age at death | rs73120402 | 4:32164053_T_C | 0.913227 | -0.03603 | 0.00816 | 1.00E-05 | 0.99425 | 0.479833 |
| Father's age at death | rs60093589 | 4:32164685_C_T | 0.912986 | -0.03591 | 0.008147 | 1.00E-05 | 0.99479 | 0.437069 |
| Father's age at death | rs73122306 | 4:32166522_T_C | 0.912988 | -0.03591 | 0.008147 | 1.00E-05 | 0.99491 | 0.437069 |
| Father's age at death | rs6811811 | 4:32218192_G_A | 0.913923 | -0.03622 | 0.00821 | 1.00E-05 | 0.99099 | 0.361518 |
| Father's age at death | rs7679485 | 4:32221925_T_C | 0.914023 | -0.03626 | 0.008215 | 1.00E-05 | 0.99085 | 0.361027 |
| Father's age at death | rs566390227 | 1:118390284_AT_A | 0.961317 | -0.05897 | 0.013358 | 1.00E-05 | 0.78663 | 0.331681 |
| Father's age at death | rs72739956 | 1:201535636_G_A | 0.957877 | 0.050529 | 0.011438 | 1.00E-05 | 0.99298 | 0.317285 |
| Father's age at death | rs59795439 | 4:32172681_A_G | 0.913019 | -0.03583 | 0.008136 | 1.10E-05 | 0.99804 | 0.426363 |
| Father's age at death | rs34757316 | 4:32176640_G_T | 0.913015 | -0.03582 | 0.008135 | 1.10E-05 | 0.99815 | 0.426363 |
| Father's age at death | rs187654180 | 4:45805815_T_C | 0.987149 | -0.09735 | 0.022126 | 1.10E-05 | 0.84624 | 0.907718 |
| Father's age at death | rs17847195 | 15:99492061_G_A | 0.780821 | 0.024509 | 0.005563 | 1.10E-05 | 0.98974 | 0.691417 |
| Father's age at death | rs9547381 | 13:86377979_G_C | 0.745257 | -0.02334 | 0.005298 | 1.10E-05 | 0.97736 | 0.292301 |
| Father's age at death | rs148152046 | 9:91228783_G_GC | 0.954243 | 0.048895 | 0.011124 | 1.10E-05 | 0.96283 | 0.463863 |
| Father's age at death | rs140806849 | 9:91231402_G_C | 0.95392 | 0.048734 | 0.011064 | 1.10E-05 | 0.96703 | 0.508323 |
| Father's age at death | rs143438639 | 9:133686990_A_G | 0.985329 | -0.09219 | 0.020939 | 1.10E-05 | 0.82839 | 0.765914 |
| Father's age at death | rs35935056 | 19:51254489_C_CA | 0.472386 | -0.02057 | 0.004685 | 1.10E-05 | 0.95103 | 0.100501 |
| Father's age at death | rs11962712 | 6:13666901_G_C | 0.940117 | -0.04237 | 0.009652 | 1.10E-05 | 0.98939 | 0.00131 |
| Father's age at death | rs9267123 | 6:31427395_G_C | 0.84153 | 0.027585 | 0.006272 | 1.10E-05 | 0.99313 | 0.162506 |
| Father's age at death | rs1142422 | 6:32610897_C_A | 0.722244 | 0.023054 | 0.005261 | 1.20E-05 | 0.94741 | 0.115308 |
| Father's age at death | rs1142429 | 6:32610908_C_T | 0.722244 | 0.023053 | 0.005261 | 1.20E-05 | 0.94741 | 0.115308 |
| Father's age at death | rs116559222 | 6:13664606_T_C | 0.93873 | -0.04169 | 0.009543 | 1.20E-05 | 0.99192 | 0.004786 |
| Father's age at death | rs545670340 | 2:128558200_G_A | 0.988597 | -0.10385 | 0.023771 | 1.20E-05 | 0.81451 | 0.205305 |
| Father's age at death | rs72843356 | 2:128337959_G_A | 0.98496 | -0.08347 | 0.019045 | 1.20E-05 | 0.96666 | 0.210097 |
| Father's age at death | rs73122336 | 4:32179310_G_A | 0.913091 | -0.03558 | 0.008133 | 1.20E-05 | 1 | 0.365814 |
| Father's age at death | rs72730370 | 1:99427319_T_A | 0.889125 | 0.031909 | 0.007282 | 1.20E-05 | 0.99793 | 0.661988 |
| Father's age at death | rs114026987 | 1:105216989_T_C | 0.978674 | 0.070468 | 0.016105 | 1.20E-05 | 0.96237 | 0.270116 |
| Father's age at death | rs77527371 | 9:4012701_C_A | 0.98855 | 0.098021 | 0.022388 | 1.20E-05 | 0.92005 | 0.800789 |
| Father's age at death | . | 17:46697690_C_T | 0.984418 | -0.08326 | 0.019021 | 1.20E-05 | 0.93572 | 0.34015 |
| Father's age at death | . | rs11417933 | 0.984418 | -0.08326 | 0.019021 | 1.20E-05 | 1 | 0.135866 |
| Father's age at death | rs148619673 | 13:108436851_T_C | 0.980727 | 0.08153 | 0.018637 | 1.20E-05 | 0.78737 | 0.001824 |
| Father's age at death | rs146013239 | 15:78751682_G_GT | 0.671545 | 0.021364 | 0.004886 | 1.20E-05 | 0.99733 | 0.225834 |
| Father's age at death | rs77733240 | 10:36544830_TGC_T | 0.0458483 | 0.049314 | 0.011263 | 1.20E-05 | 0.93998 | 0.973684 |
| Father's age at death | rs531114346 | 12:12234390_CA_C | 0.886693 | -0.03327 | 0.007618 | 1.30E-05 | 0.89539 | 0.852546 |
| Father's age at death | rs28424579 | 15:78182572_G_C | 0.859197 | -0.0288 | 0.0066 | 1.30E-05 | 0.9875 | 0.329124 |
| Father's age at death | rs11299859 | 13:93983885_CT_C | 0.763367 | 0.023912 | 0.005494 | 1.30E-05 | 0.96056 | 0.892278 |
| Father's age at death | rs7569339 | 2:133469717_A_T | 0.921706 | -0.03731 | 0.008555 | 1.30E-05 | 0.99212 | 0.889594 |
| Father's age at death | rs57385568 | 6:20798765_C_A | 0.891087 | 0.032211 | 0.007375 | 1.30E-05 | 0.98953 | 0.573311 |
| Father's age at death | rs2523451 | 6:31369151_G_A | 0.645962 | 0.020898 | 0.004791 | 1.30E-05 | 1 | 0.596197 |
| Father's age at death | . | 8:86356614_A_ATA | 0.820657 | 0.028589 | 0.006564 | 1.30E-05 | 0.83223 | 0.180094 |
| Father's age at death | rs281530 | 2:225866243_G_A | 0.320846 | 0.02146 | 0.00492 | 1.30E-05 | 0.98114 | 0.002691 |
| Father's age at death | rs77060969 | 3:53733031_C_A | 0.989389 | -0.09789 | 0.02246 | 1.30E-05 | 0.98344 | 0.158296 |
| Father's age at death | rs72730375 | 1:99433269_G_A | 0.889144 | 0.031788 | 0.007278 | 1.30E-05 | 0.99918 | 0.640973 |
| Father's age at death | rs10489920 | 1:99434493_T_A | 0.889144 | 0.031788 | 0.007278 | 1.30E-05 | 0.99918 | 0.640973 |
| Father's age at death | rs56157718 | 1:99434543_A_C | 0.889013 | 0.03173 | 0.007279 | 1.30E-05 | 0.99815 | 0.683487 |
| Father's age at death | rs72730392 | 1:99439292_A_C | 0.88922 | 0.031699 | 0.007281 | 1.30E-05 | 0.99876 | 0.609776 |
| Father's age at death | rs55646170 | 1:99455931_T_C | 0.918718 | 0.036758 | 0.008434 | 1.30E-05 | 0.98112 | 0.524675 |
| Father's age at death | rs6811434 | 4:32218043_G_C | 0.914081 | -0.0359 | 0.00822 | 1.30E-05 | 0.99045 | 0.360778 |
| Father's age at death | rs75706884 | 16:88506405_G_A | 0.981583 | 0.074093 | 0.016998 | 1.30E-05 | 1 | 0.687607 |
| Father's age at death | rs72730383 | 1:99434916_A_T | 0.889199 | 0.031622 | 0.00728 | 1.40E-05 | 0.99905 | 0.672388 |
| Father's age at death | rs11582650 | 1:99438625_A_G | 0.889168 | 0.031625 | 0.007278 | 1.40E-05 | 0.99913 | 0.609944 |
| Father's age at death | rs5776446 | 1:99427868_AT_A | 0.889121 | 0.031658 | 0.007285 | 1.40E-05 | 0.99726 | 0.651418 |
| Father's age at death | rs187113452 | 7:125888287_T_G | 0.981977 | 0.083378 | 0.019215 | 1.40E-05 | 0.80195 | 1 |
| Father's age at death | rs200586310 | 17:46697688_T_G | 0.984447 | -0.08273 | 0.01901 | 1.40E-05 | 0.93834 | 0.33847 |
| Father's age at death | rs1130148 | 6:32610868_C_T | 0.721444 | 0.022884 | 0.005261 | 1.40E-05 | 0.94592 | 0.117617 |
| Father's age at death | rs1142414 | 6:32610869_C_G | 0.721444 | 0.022884 | 0.005261 | 1.40E-05 | 0.94592 | 0.117617 |
| Father's age at death | rs78348777 | 6:13659944_C_G | 0.939434 | -0.04171 | 0.009599 | 1.40E-05 | 0.99067 | 0.003715 |
| Father's age at death | rs7581693 | 2:133469994_G_A | 0.921862 | -0.0372 | 0.00856 | 1.40E-05 | 0.99267 | 0.952472 |
| Father's age at death | rs79901388 | 3:53697049_T_C | 0.989257 | -0.09632 | 0.022273 | 1.50E-05 | 0.98808 | 0.124951 |
| Father's age at death | rs913220 | 20:10966476_C_G | 0.615421 | 0.020495 | 0.004734 | 1.50E-05 | 0.99095 | 0.376357 |
| Father's age at death | rs6077996 | 20:10972839_A_G | 0.614908 | 0.020426 | 0.00472 | 1.50E-05 | 0.99649 | 0.332269 |
| Father's age at death | rs2849687 | 21:43078849_T_C | 0.360977 | 0.020534 | 0.004751 | 1.50E-05 | 1 | 0.098039 |
| Father's age at death | rs281529 | 2:225866470_G_A | 0.319748 | 0.021302 | 0.004923 | 1.50E-05 | 0.98172 | 0.00237 |
| Father's age at death | rs5020631 | 1:242842300_G_A | 0.789547 | 0.025142 | 0.005803 | 1.50E-05 | 0.93456 | 0.626513 |
| Father's age at death | rs10489922 | 1:99441320_C_A | 0.889201 | 0.031453 | 0.007277 | 1.50E-05 | 0.99946 | 0.540344 |
| Father's age at death | rs56068933 | 1:99442430_T_C | 0.889369 | 0.031485 | 0.007283 | 1.50E-05 | 0.99909 | 0.520595 |
| Father's age at death | rs55934047 | 1:99455717_G_A | 0.889318 | 0.031565 | 0.007285 | 1.50E-05 | 0.99795 | 0.5021 |
| Father's age at death | rs72732429 | 1:99457546_T_C | 0.88921 | 0.031547 | 0.007284 | 1.50E-05 | 0.99772 | 0.511764 |
| Father's age at death | rs1110179 | 14:83080520_G_A | 0.883028 | 0.031155 | 0.007195 | 1.50E-05 | 0.97627 | 0.431877 |
| Father's age at death | rs201775425 | 17:46697689_G_C | 0.9845 | -0.08245 | 0.019046 | 1.50E-05 | 0.9379 | 0.336225 |
| Father's age at death | rs41283427 | 17:39928598_C_T | 0.957512 | 0.050811 | 0.011742 | 1.50E-05 | 0.93132 | 0.353973 |
| Father's age at death | rs17469997 | 10:8455932_G_C | 0.891768 | -0.03195 | 0.007383 | 1.50E-05 | 0.99084 | 0.508885 |
| Father's age at death | rs150856583 | 15:78178220_GCACACAGACACAGATACA_G | 0.857703 | -0.02846 | 0.006583 | 1.50E-05 | 0.98394 | 0.288151 |
| Father's age at death | rs5812158 | 15:40636250_A_AC | 0.19816 | 0.025731 | 0.005939 | 1.50E-05 | 0.93151 | 0.478333 |
| Father's age at death | rs10491842 | 9:79982671_C_G | 0.89596 | 0.03244 | 0.007497 | 1.50E-05 | 0.99826 | 0.506536 |
| Father's age at death | rs116200172 | 11:33074042_G_A | 0.989613 | 0.11575 | 0.026827 | 1.60E-05 | 0.70456 | 0.482992 |
| Father's age at death | rs7162616 | 15:78188471_G_T | 0.857337 | -0.02813 | 0.006529 | 1.60E-05 | 1 | 0.741669 |
| Father's age at death | rs72738732 | 15:78752188_C_G | 0.672094 | 0.021074 | 0.004886 | 1.60E-05 | 0.99791 | 0.213157 |
| Father's age at death | rs17484235 | 15:78761414_C_G | 0.671838 | 0.021057 | 0.004883 | 1.60E-05 | 0.99875 | 0.249012 |
| Father's age at death | rs150744542 | 9:4041608_GTTC_G | 0.98866 | 0.09405 | 0.021812 | 1.60E-05 | 0.98242 | 0.527327 |
| Father's age at death | rs183938645 | 3:48806775_A_T | 0.9596 | -0.0538 | 0.012489 | 1.60E-05 | 0.86075 | 0.331872 |
| Father's age at death | rs7267407 | 20:10972082_A_G | 0.614888 | 0.020348 | 0.00472 | 1.60E-05 | 0.99649 | 0.323236 |
| Father's age at death | rs576120134 | 16:86141557_A_G | 0.959103 | 0.061128 | 0.01419 | 1.60E-05 | 0.66138 | 0.970695 |
| Father's age at death | rs17119341 | 1:99439198_A_G | 0.889221 | 0.031458 | 0.007284 | 1.60E-05 | 0.99806 | 0.609776 |
| Father's age at death | rs17273773 | 1:99439496_C_G | 0.889538 | 0.031507 | 0.007295 | 1.60E-05 | 0.99781 | 0.71471 |
| Father's age at death | rs72732404 | 1:99445571_T_C | 0.889214 | 0.03138 | 0.007275 | 1.60E-05 | 0.99989 | 0.521183 |
| Father's age at death | rs11584061 | 1:99445930_T_C | 0.889213 | 0.031383 | 0.007275 | 1.60E-05 | 0.99985 | 0.521183 |
| Father's age at death | rs72732408 | 1:99448523_C_T | 0.889164 | 0.031429 | 0.007277 | 1.60E-05 | 0.99895 | 0.511918 |
| Father's age at death | rs138553015 | 1:99449441_G_A | 0.889181 | 0.031422 | 0.007277 | 1.60E-05 | 0.99911 | 0.511882 |
| Father's age at death | rs4399180 | 1:99451122_T_A | 0.889132 | 0.031359 | 0.007277 | 1.60E-05 | 0.99886 | 0.502701 |
| Father's age at death | rs4593847 | 1:99451272_C_T | 0.889182 | 0.031425 | 0.007277 | 1.60E-05 | 0.99912 | 0.511882 |
| Father's age at death | rs72732417 | 1:99453967_T_C | 0.88919 | 0.031394 | 0.007278 | 1.60E-05 | 0.99893 | 0.511835 |
| Father's age at death | rs72732418 | 1:99453975_C_T | 0.889357 | 0.031466 | 0.007289 | 1.60E-05 | 0.99751 | 0.520715 |
| Father's age at death | rs72732419 | 1:99454697_T_C | 0.889183 | 0.031422 | 0.007277 | 1.60E-05 | 0.99913 | 0.511882 |
| Father's age at death | rs72732421 | 1:99455081_A_G | 0.889179 | 0.031419 | 0.007277 | 1.60E-05 | 0.99908 | 0.521291 |
| Father's age at death | rs11587711 | 1:99455319_G_A | 0.889182 | 0.031424 | 0.007277 | 1.60E-05 | 0.99912 | 0.511882 |
| Father's age at death | rs72732425 | 1:99456350_C_A | 0.88954 | 0.031505 | 0.007294 | 1.60E-05 | 0.99763 | 0.548896 |
| Father's age at death | rs7522359 | 1:99430688_C_T | 0.888768 | 0.031411 | 0.007281 | 1.60E-05 | 0.99598 | 0.73821 |
| Father's age at death | rs530193509 | 4:1548856_C_T | 0.710868 | -0.02314 | 0.005367 | 1.60E-05 | 0.88134 | 0.238814 |
| Father's age at death | rs548634178 | 4:1548857_T_A | 0.710868 | -0.02314 | 0.005367 | 1.60E-05 | 0.88134 | 0.238814 |
| Father's age at death | rs78769711 | 4:31575258_A_G | 0.980755 | 0.080222 | 0.018675 | 1.70E-05 | 0.79611 | 1 |
| Father's age at death | rs148769073 | 7:125909880_T_C | 0.981906 | 0.082513 | 0.019177 | 1.70E-05 | 0.8019 | 1 |
| Father's age at death | rs188249561 | 12:11225158_C_T | 0.978455 | 0.077899 | 0.018091 | 1.70E-05 | 0.7588 | 0.890301 |
| Father's age at death | rs73985884 | 17:46689008_A_G | 0.98385 | -0.07893 | 0.01836 | 1.70E-05 | 0.97142 | 0.200244 |
| Father's age at death | rs2025946 | 13:93975485_G_T | 0.791668 | 0.024562 | 0.005707 | 1.70E-05 | 0.97342 | 0.902815 |
| Father's age at death | rs11598479 | 10:73232572_T_G | 0.983836 | 0.079614 | 0.018528 | 1.70E-05 | 0.95773 | 0.176068 |
| Father's age at death | rs72507747 | 15:78188412_GC_G | 0.857622 | -0.0281 | 0.006539 | 1.70E-05 | 0.99684 | 0.37016 |
| Father's age at death | rs540124715 | 20:10970723_G_GA | 0.618842 | 0.02042 | 0.004752 | 1.70E-05 | 0.98719 | 0.306627 |
| Father's age at death | rs74382585 | 6:13695366_C_T | 0.939151 | -0.04111 | 0.009566 | 1.70E-05 | 0.99249 | 0.002354 |
| Father's age at death | rs1130144 | 6:32610857_G_A | 0.72166 | 0.022662 | 0.005262 | 1.70E-05 | 0.94569 | 0.137117 |
| Father's age at death | rs1130145 | 6:32610858_C_T | 0.72166 | 0.022662 | 0.005262 | 1.70E-05 | 0.94569 | 0.137117 |
| Father's age at death | rs2905719 | 6:31344390_A_G | 0.852509 | 0.027731 | 0.006455 | 1.70E-05 | 0.99964 | 0.705277 |
| Father's age at death | rs2523546 | 6:31332920_G_A | 0.852479 | 0.027756 | 0.006454 | 1.70E-05 | 0.99999 | 0.70531 |
| Father's age at death | rs2844577 | 6:31334422_T_C | 0.852488 | 0.027739 | 0.006454 | 1.70E-05 | 0.99999 | 0.679923 |
| Father's age at death | rs2596430 | 6:31335431_T_C | 0.852493 | 0.027734 | 0.006454 | 1.70E-05 | 0.99997 | 0.679923 |
| Father's age at death | rs2922994 | 6:31335901_A_G | 0.852479 | 0.027743 | 0.006454 | 1.70E-05 | 0.99993 | 0.671568 |
| Father's age at death | rs3094012 | 6:31434520_G_C | 0.852134 | 0.027716 | 0.00644 | 1.70E-05 | 0.9995 | 0.70557 |
| Father's age at death | rs114953223 | 3:49280206_A_G | 0.953881 | -0.04719 | 0.010963 | 1.70E-05 | 0.9834 | 0.121964 |
| Father's age at death | rs34413503 | 3:72208267_G_A | 0.971283 | 0.058896 | 0.013686 | 1.70E-05 | 1 | 1 |
| Father's age at death | rs201110529 | 3:49125405_GAC_G | 0.958875 | -0.05249 | 0.012251 | 1.80E-05 | 0.87929 | 0.240093 |
| Father's age at death | rs72525425 | 20:10969095_C_CTT | 0.612671 | 0.020223 | 0.004719 | 1.80E-05 | 0.99534 | 0.233024 |
| Father's age at death | rs72619590 | 22:50982272_G_A | 0.931622 | 0.03941 | 0.009187 | 1.80E-05 | 0.97149 | 0.838637 |
| Father's age at death | rs58446907 | 2:133468238_A_G | 0.921009 | -0.03647 | 0.008516 | 1.80E-05 | 0.99296 | 1 |
| Father's age at death | rs9265949 | 6:31315229_C_T | 0.856202 | 0.028025 | 0.006536 | 1.80E-05 | 0.99485 | 0.916054 |
| Father's age at death | rs2442735 | 6:31346653_A_G | 0.852499 | 0.027684 | 0.006455 | 1.80E-05 | 0.99684 | 0.543576 |
| Father's age at death | rs2523457 | 6:31365707_G_A | 0.646046 | 0.020547 | 0.004789 | 1.80E-05 | 0.99952 | 0.869703 |
| Father's age at death | rs1130139 | 6:32610841_T_C | 0.72233 | 0.022554 | 0.005266 | 1.80E-05 | 0.94775 | 0.039673 |
| Father's age at death | rs1130142 | 6:32610846_C_G | 0.722338 | 0.02255 | 0.005266 | 1.80E-05 | 0.94773 | 0.040365 |
| Father's age at death | rs647915 | 2:225867775_C_A | 0.320623 | 0.021121 | 0.004918 | 1.80E-05 | 0.98193 | 0.002057 |
| Father's age at death | rs377275135 | 7:79769294_CT_C | 0.767638 | 0.024435 | 0.005703 | 1.80E-05 | 0.90117 | 0.764733 |
| Father's age at death | rs10489921 | 1:99440787_T_C | 0.889463 | 0.031212 | 0.007285 | 1.80E-05 | 0.99894 | 0.483181 |
| Father's age at death | rs72730399 | 1:99441096_A_T | 0.889463 | 0.031212 | 0.007285 | 1.80E-05 | 0.99895 | 0.483181 |
| Father's age at death | rs72730401 | 1:99444102_A_T | 0.889463 | 0.031212 | 0.007285 | 1.80E-05 | 0.99895 | 0.483181 |
| Father's age at death | rs17483548 | 15:78730313_G_A | 0.672079 | 0.020953 | 0.004889 | 1.80E-05 | 0.99753 | 0.123581 |
| Father's age at death | rs17405217 | 15:78731149_C_T | 0.672136 | 0.020994 | 0.004889 | 1.80E-05 | 0.99738 | 0.120387 |
| Father's age at death | rs17484524 | 15:78772676_A_G | 0.671923 | 0.020926 | 0.004883 | 1.80E-05 | 0.99851 | 0.248966 |
| Father's age at death | rs72744273 | 9:79891880_T_C | 0.91257 | 0.034822 | 0.008123 | 1.80E-05 | 0.99349 | 0.800866 |
| Father's age at death | rs35973211 | 12:14041370_T_TA | 0.417276 | -0.02401 | 0.005594 | 1.80E-05 | 0.68172 | 0.24542 |
| Father's age at death | rs11255684 | 10:8452638_G_A | 0.891034 | -0.03201 | 0.007455 | 1.80E-05 | 0.96485 | 0.221871 |
| Father's age at death | rs17483686 | 15:78733390_A_T | 0.668608 | 0.020855 | 0.004873 | 1.90E-05 | 0.99761 | 0.227955 |
| Father's age at death | rs6224 | 15:91423543_G_T | 0.524122 | 0.019661 | 0.004598 | 1.90E-05 | 0.99491 | 0.282273 |
| Father's age at death | rs6561605 | 13:51565390_T_G | 0.411729 | -0.02024 | 0.004736 | 1.90E-05 | 0.96355 | 0.957282 |
| Father's age at death | rs77570471 | 17:65963519_A_C | 0.96754 | -0.05963 | 0.01395 | 1.90E-05 | 0.85053 | 0.77922 |
| Father's age at death | rs72742565 | 9:79817081_A_G | 0.912529 | 0.03463 | 0.008105 | 1.90E-05 | 0.99691 | 0.7871 |
| Father's age at death | rs6077992 | 20:10967661_T_C | 0.615767 | 0.020245 | 0.004734 | 1.90E-05 | 0.99098 | 0.423994 |
| Father's age at death | rs56091950 | 6:145352653_A_T | 0.907533 | 0.03453 | 0.008079 | 1.90E-05 | 0.96083 | 0.07251 |
| Father's age at death | rs116544378 | 3:29828590_T_C | 0.933733 | -0.03989 | 0.009329 | 1.90E-05 | 0.97109 | 0.851924 |
| Father's age at death | rs9273064 | 6:32612202_A_G | 0.668976 | 0.021947 | 0.005132 | 1.90E-05 | 0.90437 | 0.015631 |
| Father's age at death | rs9273068 | 6:32612214_T_A | 0.668949 | 0.021943 | 0.00513 | 1.90E-05 | 0.90388 | 0.050594 |
| Father's age at death | rs2923008 | 6:31316911_G_T | 0.85527 | 0.027802 | 0.006507 | 1.90E-05 | 0.99942 | 0.666438 |
| Father's age at death | rs72730402 | 1:99444284_C_T | 0.889072 | 0.031118 | 0.007275 | 1.90E-05 | 0.99891 | 0.550427 |
| Father's age at death | rs11944911 | 4:32222282_T_C | 0.915187 | -0.03576 | 0.008368 | 1.90E-05 | 0.96526 | 0.553988 |
| Father's age at death | rs139961336 | 4:32222514_T_TTA | 0.921777 | -0.03827 | 0.008944 | 1.90E-05 | 0.91112 | 0.32964 |
| Father's age at death | rs199564024 | 4:48964602_TC_T | 0.886662 | -0.04671 | 0.010913 | 1.90E-05 | 0.43683 | 0.720377 |
| Father's age at death | rs9992204 | 4:89010495_A_T | 0.983167 | 0.076441 | 0.017898 | 1.90E-05 | 0.9823 | 0.485389 |
| Father's age at death | rs4563466 | 4:89006249_C_G | 0.983116 | 0.076161 | 0.017878 | 2.00E-05 | 0.98145 | 0.487326 |
| Father's age at death | rs62465206 | 7:132279785_C_T | 0.163656 | 0.02666 | 0.006245 | 2.00E-05 | 0.97488 | 0.16814 |
| Father's age at death | rs41478548 | 1:99456080_G_A | 0.889293 | 0.031074 | 0.007281 | 2.00E-05 | 0.99901 | 0.502169 |
| Father's age at death | rs59879664 | 1:99531458_AC_A | 0.905949 | 0.033583 | 0.007867 | 2.00E-05 | 0.98977 | 0.589183 |
| Father's age at death | rs72740397 | 9:79804894_T_C | 0.912357 | 0.03456 | 0.008095 | 2.00E-05 | 0.99771 | 0.843273 |
| Father's age at death | rs72740399 | 9:79807472_C_T | 0.912409 | 0.034511 | 0.0081 | 2.00E-05 | 0.99712 | 0.843199 |
| Father's age at death | rs72740401 | 9:79807768_G_A | 0.912346 | 0.034549 | 0.008094 | 2.00E-05 | 0.99771 | 0.84329 |
| Father's age at death | rs143896542 | 9:79810680_C_CTTTATT | 0.912328 | 0.034484 | 0.008094 | 2.00E-05 | 0.99769 | 0.843327 |
| Father's age at death | rs72742564 | 9:79816880_T_C | 0.912337 | 0.034455 | 0.008089 | 2.00E-05 | 0.99883 | 0.829276 |
| Father's age at death | rs17063528 | 9:79924674_A_C | 0.903025 | 0.033031 | 0.007735 | 2.00E-05 | 0.9989 | 0.543688 |
| Father's age at death | rs9279179 | 6:31346849_CG_C | 0.852235 | 0.027506 | 0.006443 | 2.00E-05 | 0.99626 | 0.128003 |
| Father's age at death | . | 6:31367322_C_A | 0.909769 | 0.044866 | 0.01052 | 2.00E-05 | 0.58041 | 0.171321 |
| Father's age at death | rs9273054 | 6:32612079_T_C | 0.670237 | 0.021801 | 0.005106 | 2.00E-05 | 0.90792 | 0.783935 |
| Father's age at death | rs9273055 | 6:32612088_G_T | 0.670199 | 0.021791 | 0.005106 | 2.00E-05 | 0.9079 | 0.809137 |
| Father's age at death | rs3131926 | 6:31013085_C_T | 0.515665 | 0.019428 | 0.004561 | 2.00E-05 | 0.99995 | 0.045793 |
| Father's age at death | rs76503181 | 6:13647364_T_C | 0.939028 | -0.04083 | 0.009568 | 2.00E-05 | 0.99067 | 0.003922 |
| Father's age at death | rs8101336 | 19:51254550_A_G | 0.445076 | -0.01959 | 0.004588 | 2.00E-05 | 1 | 0.082054 |
| Father's age at death | rs201924326 | 3:49085059_G_GGCAT | 0.954325 | -0.04702 | 0.011026 | 2.00E-05 | 0.98136 | 0.173628 |
| Father's age at death | rs141260127 | 3:49374250_C_T | 0.958337 | -0.05173 | 0.012134 | 2.00E-05 | 0.88504 | 0.17911 |
| Father's age at death | rs77560262 | 2:193108685_C_G | 0.984299 | 0.077918 | 0.018281 | 2.00E-05 | 1 | 0.000502 |
| Father's age at death | rs72844763 | 2:133469250_T_A | 0.921301 | -0.03629 | 0.008524 | 2.10E-05 | 0.99417 | 0.905716 |
| Father's age at death | rs151188051 | 6:21116195_TA_T | 0.872392 | 0.029765 | 0.007001 | 2.10E-05 | 0.95548 | 0.114911 |
| Father's age at death | rs9266001 | 6:31316695_C_A | 0.85534 | 0.027722 | 0.00651 | 2.10E-05 | 0.99872 | 0.73536 |
| Father's age at death | rs148123229 | 2:211643510_T_C | 0.987534 | 0.093946 | 0.022101 | 2.10E-05 | 0.86718 | 0.479126 |
| Father's age at death | rs1487565 | 4:141779593_A_G | 0.150763 | 0.027226 | 0.006399 | 2.10E-05 | 1 | 0.991038 |
| Father's age at death | rs11285211 | 7:132278070_GA_G | 0.160576 | 0.026791 | 0.006302 | 2.10E-05 | 0.97209 | 0.199885 |
| Father's age at death | rs571514888 | 16:67725420_CA_C | 0.95254 | -0.04946 | 0.011615 | 2.10E-05 | 0.85585 | 1 |
| Father's age at death | rs72742563 | 9:79816709_C_G | 0.912487 | 0.034496 | 0.008102 | 2.10E-05 | 0.99731 | 0.815036 |
| Father's age at death | rs17422898 | 9:79889415_A_G | 0.911957 | 0.034369 | 0.008086 | 2.10E-05 | 0.99608 | 0.843927 |
| Father's age at death | rs17483721 | 15:78733731_T_C | 0.669087 | 0.02073 | 0.004876 | 2.10E-05 | 0.99718 | 0.243047 |
| Father's age at death | rs7047834 | 9:10230457_T_C | 0.949731 | -0.04704 | 0.011068 | 2.10E-05 | 0.89463 | 0.787136 |
| Father's age at death | rs79677664 | 18:9819331_C_T | 0.985306 | 0.082942 | 0.019533 | 2.20E-05 | 0.94949 | 0.42503 |
| Father's age at death | rs2089162 | 15:78739763_A_G | 0.672123 | 0.020761 | 0.004888 | 2.20E-05 | 0.99782 | 0.130077 |
| Father's age at death | rs72740392 | 9:79797593_A_G | 0.912311 | 0.034363 | 0.008096 | 2.20E-05 | 0.99716 | 0.857395 |
| Father's age at death | rs55760280 | 9:79813068_A_T | 0.913708 | 0.034772 | 0.00819 | 2.20E-05 | 0.98869 | 0.841103 |
| Father's age at death | rs62330864 | 5:2571642_G_C | 0.987989 | -0.08974 | 0.021143 | 2.20E-05 | 0.98593 | 0.902757 |
| Father's age at death | rs532367474 | 5:2574965_G_A | 0.987458 | -0.08752 | 0.020632 | 2.20E-05 | 0.99264 | 0.907137 |
| Father's age at death | rs71058553 | 2:234305662_TGGG_T | 0.28628 | -0.02298 | 0.005422 | 2.20E-05 | 0.86585 | 0.337676 |
| Father's age at death | rs9265994 | 6:31316526_T_C | 0.855071 | 0.027601 | 0.006503 | 2.20E-05 | 0.99972 | 0.64992 |
| Father's age at death | rs72624911 | 3:49064576_C_T | 0.953867 | -0.04636 | 0.010916 | 2.20E-05 | 0.99188 | 0.157384 |
| Father's age at death | rs114934413 | 3:48939197_C_T | 0.955192 | -0.0477 | 0.011247 | 2.20E-05 | 0.96041 | 0.120276 |
| Father's age at death | rs11590124 | 1:99507778_C_A | 0.905608 | 0.033263 | 0.007832 | 2.20E-05 | 0.99535 | 0.66179 |
| Father's age at death | rs10158769 | 1:99530158_T_C | 0.905615 | 0.033318 | 0.007847 | 2.20E-05 | 0.9918 | 0.66179 |
| Father's age at death | rs72734411 | 1:99551047_T_C | 0.875184 | 0.029797 | 0.007022 | 2.20E-05 | 0.9666 | 0.130716 |
| Father's age at death | rs140416690 | 1:79928794_G_C | 0.973166 | 0.063319 | 0.014928 | 2.20E-05 | 0.90012 | 0.439258 |
| Father's age at death | rs187607506 | 1:55491915_C_T | 0.756366 | -0.02301 | 0.005424 | 2.20E-05 | 0.96165 | 0.12613 |
| Father's age at death | rs5887584 | 7:132288623_CA_C | 0.174081 | 0.025601 | 0.006038 | 2.20E-05 | 0.99165 | 0.052036 |
| Father's age at death | rs971553 | 7:132288635_T_C | 0.174079 | 0.025598 | 0.006038 | 2.20E-05 | 0.99166 | 0.052036 |
| Father's age at death | rs79082970 | 4:89006009_T_C | 0.983122 | 0.07592 | 0.017879 | 2.20E-05 | 0.98157 | 0.487125 |
| Father's age at death | rs114953699 | 4:89006008_C_T | 0.983111 | 0.075753 | 0.017879 | 2.30E-05 | 0.98093 | 0.487517 |
| Father's age at death | rs13234673 | 7:132290051_C_A | 0.850755 | -0.02718 | 0.006427 | 2.30E-05 | 0.99207 | 0.28653 |
| Father's age at death | rs17390663 | 1:99479687_C_G | 0.907251 | 0.033333 | 0.007876 | 2.30E-05 | 0.99858 | 0.595895 |
| Father's age at death | rs72732456 | 1:99483259_A_G | 0.907221 | 0.033361 | 0.007871 | 2.30E-05 | 0.99945 | 0.560932 |
| Father's age at death | rs72732457 | 1:99485832_A_G | 0.907259 | 0.033339 | 0.007877 | 2.30E-05 | 0.99856 | 0.560815 |
| Father's age at death | rs11585201 | 1:99487671_C_T | 0.907264 | 0.033329 | 0.007877 | 2.30E-05 | 0.99854 | 0.56079 |
| Father's age at death | rs140868217 | 1:99544191_G_T | 0.875894 | 0.029696 | 0.007011 | 2.30E-05 | 0.97425 | 0.096228 |
| Father's age at death | rs62330869 | 5:2578621_A_C | 0.984822 | -0.07974 | 0.018818 | 2.30E-05 | 0.98672 | 0.501233 |
| Father's age at death | rs4887056 | 15:78734585_A_G | 0.668736 | 0.020629 | 0.004874 | 2.30E-05 | 0.99765 | 0.208387 |
| Father's age at death | rs139043337 | 15:78734588_ATTAC_A | 0.668646 | 0.020646 | 0.004874 | 2.30E-05 | 0.99742 | 0.206096 |
| Father's age at death | rs55983731 | 15:78735269_C_T | 0.668741 | 0.020624 | 0.004874 | 2.30E-05 | 0.99764 | 0.208372 |
| Father's age at death | rs72738718 | 15:78735438_G_C | 0.668765 | 0.020613 | 0.004874 | 2.30E-05 | 0.99757 | 0.208372 |
| Father's age at death | rs10759100 | 9:9908722_T_C | 0.803226 | -0.0243 | 0.005738 | 2.30E-05 | 0.99717 | 0.048395 |
| Father's age at death | rs10816224 | 9:9909933_G_A | 0.80312 | -0.0243 | 0.005742 | 2.30E-05 | 0.99595 | 0.084101 |
| Father's age at death | rs139079434 | 17:65515342_G_A | 0.94971 | -0.047 | 0.011107 | 2.30E-05 | 0.88704 | 0.409525 |
| Father's age at death | rs11016259 | 10:130264979_C_T | 0.531271 | -0.01959 | 0.004623 | 2.30E-05 | 0.98039 | 0.871232 |
| Father's age at death | rs182552329 | 3:48951365_A_T | 0.955562 | -0.04806 | 0.011338 | 2.30E-05 | 0.95278 | 0.143097 |
| Father's age at death | rs199678483 | 3:49967567_AT_A | 0.970815 | -0.06134 | 0.014496 | 2.30E-05 | 0.87794 | 0.878478 |
| Father's age at death | rs9830051 | 3:85396965_T_G | 0.562258 | -0.02053 | 0.004845 | 2.30E-05 | 0.8994 | 0.348626 |
| Father's age at death | rs72839434 | 2:129065289_G_A | 0.978861 | -0.06911 | 0.016312 | 2.30E-05 | 0.94394 | 0.030996 |
| Father's age at death | rs139871602 | 2:160216002_G_C | 0.986178 | -0.09695 | 0.022895 | 2.30E-05 | 0.72976 | 0.287587 |
| Father's age at death | rs2523607 | 6:31322790_T_A | 0.849804 | 0.027158 | 0.006409 | 2.30E-05 | 0.9995 | 0.595155 |
| Father's age at death | rs2596495 | 6:31323416_G_C | 0.849809 | 0.027141 | 0.006409 | 2.30E-05 | 0.99925 | 0.626859 |
| Father's age at death | rs528186543 | 6:31323500_CCT_C | 0.849878 | 0.027121 | 0.006412 | 2.30E-05 | 0.99875 | 0.650969 |
| Father's age at death | rs4990036 | 6:31323506_C_T | 0.849879 | 0.027127 | 0.006412 | 2.30E-05 | 0.99876 | 0.650969 |
| Father's age at death | rs539339910 | 6:31363431_AAAAC_A | 0.852259 | 0.027343 | 0.006456 | 2.30E-05 | 0.99671 | 0.963476 |
| Father's age at death | rs547306579 | 6:32610781_C_T | 0.728258 | 0.022292 | 0.005264 | 2.30E-05 | 0.9645 | 0.000645 |
| Father's age at death | rs200359098 | 19:30934399_G_GT | 0.984272 | -0.07921 | 0.018694 | 2.30E-05 | 0.96231 | 0.120362 |
| Father's age at death | rs550552339 | 19:30934400_C_CAATG | 0.984272 | -0.07921 | 0.018694 | 2.30E-05 | 0.96231 | 0.120362 |
| Father's age at death | rs140343754 | 19:52151130_G_A | 0.974982 | 0.062912 | 0.014874 | 2.30E-05 | 0.97235 | 0.185067 |
| Father's age at death | rs1048645 | 6:32610786_C_T | 0.728159 | 0.022246 | 0.005264 | 2.40E-05 | 0.96457 | 0.000536 |
| Father's age at death | rs9273080 | 6:32612333_C_T | 0.695222 | 0.022304 | 0.005276 | 2.40E-05 | 0.89336 | 0.27874 |
| Father's age at death | rs9272667 | 6:32608760_T_C | 0.688551 | 0.02163 | 0.005125 | 2.40E-05 | 0.92826 | 0.518406 |
| Father's age at death | rs9272668 | 6:32608763_G_A | 0.688652 | 0.021624 | 0.005125 | 2.40E-05 | 0.92855 | 0.531496 |
| Father's age at death | rs9262572 | 6:31012879_C_T | 0.582299 | 0.020835 | 0.004938 | 2.40E-05 | 0.87672 | 0.200198 |
| Father's age at death | rs9262573 | 6:31012880_C_T | 0.582299 | 0.020835 | 0.004938 | 2.40E-05 | 0.87672 | 0.200198 |
| Father's age at death | rs2523454 | 6:31367865_G_A | 0.652197 | 0.020289 | 0.004807 | 2.40E-05 | 1 | 0.974633 |
| Father's age at death | rs569200394 | 6:31391757_GA_G | 0.604367 | 0.019808 | 0.004687 | 2.40E-05 | 0.99538 | 0.642347 |
| Father's age at death | rs9265969 | 6:31315706_G_A | 0.836253 | 0.026152 | 0.006186 | 2.40E-05 | 0.99775 | 0.720261 |
| Father's age at death | rs72732462 | 1:99490015_C_T | 0.907694 | 0.033402 | 0.007906 | 2.40E-05 | 0.99566 | 0.618429 |
| Father's age at death | rs72732478 | 1:99504017_G_A | 0.905434 | 0.033055 | 0.007827 | 2.40E-05 | 0.99503 | 0.699278 |
| Father's age at death | rs144986806 | 1:99544047_G_A | 0.875525 | 0.029602 | 0.00701 | 2.40E-05 | 0.97227 | 0.102511 |
| Father's age at death | rs467289 | 5:178651577_G_A | 0.298397 | 0.022115 | 0.005236 | 2.40E-05 | 0.91466 | 0.241363 |
| Father's age at death | rs1504550 | 15:78766250_A_G | 0.673831 | 0.020704 | 0.004899 | 2.40E-05 | 0.9956 | 0.229182 |
| Father's age at death | rs117596516 | 9:79842647_C_T | 0.911525 | 0.034098 | 0.008067 | 2.40E-05 | 0.99738 | 0.971585 |
| Father's age at death | rs73654005 | 9:79886642_A_G | 0.912321 | 0.034292 | 0.00811 | 2.40E-05 | 0.99396 | 0.857403 |
| Father's age at death | rs74513092 | 9:79900746_T_C | 0.911644 | 0.034017 | 0.008061 | 2.40E-05 | 0.99929 | 0.844427 |
| Father's age at death | . | 9:79902140_T_TTC | 0.911633 | 0.034073 | 0.00806 | 2.40E-05 | 0.99016 | 0.069295 |
| Father's age at death | . | 9:79902140_T_TTCTCTC | 0.911633 | 0.034073 | 0.00806 | 2.40E-05 | 0.99928 | 0.85841 |
| Father's age at death | rs34177641 | 9:9909068_GA_G | 0.802966 | -0.02422 | 0.005734 | 2.40E-05 | 0.99764 | 0.052942 |
| Father's age at death | rs11244755 | 10:127687017_C_T | 0.672761 | -0.02068 | 0.004904 | 2.50E-05 | 0.98902 | 0.627769 |
| Father's age at death | rs17157634 | 10:2140808_C_T | 0.925483 | 0.036685 | 0.008706 | 2.50E-05 | 1 | 0.720643 |
| Father's age at death | rs72742574 | 9:79828516_A_G | 0.91167 | 0.033968 | 0.008065 | 2.50E-05 | 0.99852 | 0.844386 |
| Father's age at death | rs145189157 | 9:79829077_A_G | 0.911697 | 0.03397 | 0.008066 | 2.50E-05 | 0.99847 | 0.830409 |
| Father's age at death | rs72742577 | 9:79830220_C_G | 0.911697 | 0.03397 | 0.008066 | 2.50E-05 | 0.99847 | 0.830409 |
| Father's age at death | rs17785835 | 9:79834267_A_G | 0.91167 | 0.033968 | 0.008065 | 2.50E-05 | 0.99852 | 0.844386 |
| Father's age at death | rs72742584 | 9:79835177_A_C | 0.911647 | 0.033967 | 0.008063 | 2.50E-05 | 0.99882 | 0.844442 |
| Father's age at death | rs56180139 | 9:79892600_T_C | 0.911692 | 0.034012 | 0.008064 | 2.50E-05 | 0.99897 | 0.844332 |
| Father's age at death | rs199814594 | 9:79897872_G_GA | 0.908008 | 0.033676 | 0.007992 | 2.50E-05 | 0.98099 | 0.890437 |
| Father's age at death | rs72744275 | 9:79899146_A_T | 0.911673 | 0.033958 | 0.008061 | 2.50E-05 | 0.99952 | 0.858365 |
| Father's age at death | rs72744280 | 9:79901905_C_T | 0.911898 | 0.034092 | 0.008081 | 2.50E-05 | 0.99685 | 0.830051 |
| Father's age at death | rs1049848 | 17:27401176_G_C | 0.981622 | 0.077261 | 0.018342 | 2.50E-05 | 0.85924 | 0.629463 |
| Father's age at death | rs200266974 | 15:91412848_G_GC | 0.642463 | 0.021934 | 0.005208 | 2.50E-05 | 0.83683 | 0.777678 |
| Father's age at death | rs28593679 | 15:78186467_T_C | 0.882792 | -0.03012 | 0.007149 | 2.50E-05 | 0.98848 | 0.697124 |
| Father's age at death | rs7748141 | 6:31288877_T_C | 0.855065 | 0.027668 | 0.006564 | 2.50E-05 | 0.97383 | 0.036174 |
| Father's age at death | rs9272672 | 6:32608849_T_C | 0.720278 | 0.022114 | 0.005245 | 2.50E-05 | 0.94079 | 0.231761 |
| Father's age at death | rs16884376 | 6:21014996_A_T | 0.880666 | 0.029963 | 0.007116 | 2.50E-05 | 0.9802 | 0.336775 |
| Father's age at death | rs79636955 | 6:13661551_A_T | 0.939588 | -0.04047 | 0.009596 | 2.50E-05 | 0.99363 | 0.003926 |
| Father's age at death | rs137950269 | 3:48990051_G_C | 0.953985 | -0.04611 | 0.01094 | 2.50E-05 | 0.99011 | 0.147238 |
| Father's age at death | rs2698951 | 8:7778470_G_C | 0.911069 | -0.0504 | 0.011952 | 2.50E-05 | 0.45015 | 0.176807 |
| Father's age at death | rs568162171 | 20:7024910_AGG_A | 0.989309 | 0.106253 | 0.025195 | 2.50E-05 | 0.77881 | 0.677267 |
| Father's age at death | rs11586703 | 1:99518560_G_T | 0.905573 | 0.032986 | 0.007824 | 2.50E-05 | 0.99696 | 0.67414 |
| Father's age at death | rs1017288 | 7:132277378_C_T | 0.174014 | 0.025599 | 0.006077 | 2.50E-05 | 0.9794 | 0.063768 |
| Father's age at death | rs117920163 | 14:33055967_G_A | 0.979868 | -0.08233 | 0.019548 | 2.50E-05 | 0.69735 | 0.65734 |
| Father's age at death | rs11583085 | 1:99438844_T_C | 0.891661 | 0.031089 | 0.007393 | 2.60E-05 | 0.98774 | 0.542101 |
| Father's age at death | rs72732488 | 1:99515890_C_A | 0.905564 | 0.032941 | 0.007831 | 2.60E-05 | 0.99498 | 0.649756 |
| Father's age at death | rs12032415 | 1:99546661_C_T | 0.876272 | 0.029552 | 0.007021 | 2.60E-05 | 0.97352 | 0.092704 |
| Father's age at death | rs528839050 | 1:55646724_A_AT | 0.870085 | -0.02926 | 0.006953 | 2.60E-05 | 0.95457 | 0.788243 |
| Father's age at death | rs2009746 | 15:78754102_A_G | 0.66758 | 0.020471 | 0.004869 | 2.60E-05 | 0.99733 | 0.352176 |
| Father's age at death | rs77010649 | 5:2568720_C_A | 0.984921 | -0.07946 | 0.018878 | 2.60E-05 | 0.98678 | 0.698313 |
| Father's age at death | rs56003362 | 9:79818579_T_C | 0.91164 | 0.033898 | 0.008062 | 2.60E-05 | 0.99885 | 0.858438 |
| Father's age at death | rs72742573 | 9:79828406_C_T | 0.911681 | 0.033922 | 0.008065 | 2.60E-05 | 0.99852 | 0.830445 |
| Father's age at death | rs112582806 | 9:79828579_G_T | 0.911681 | 0.033922 | 0.008065 | 2.60E-05 | 0.99852 | 0.830445 |
| Father's age at death | rs139014781 | 9:79942569_A_ATTAT | 0.911646 | 0.033884 | 0.008061 | 2.60E-05 | 0.99941 | 0.844394 |
| Father's age at death | . | 20:10984360_T_C | 0.532249 | 0.020567 | 0.004894 | 2.60E-05 | 0.87691 | 0.76768 |
| Father's age at death | . | 20:10984360_T_TC | 0.532249 | 0.020567 | 0.004894 | 2.60E-05 | 0.79861 | 0.306084 |
| Father's age at death | rs531761147 | 6:20912877_A_AT | 0.933329 | 0.039812 | 0.009466 | 2.60E-05 | 0.93085 | 0.033497 |
| Father's age at death | rs9265811 | 6:31310372_C_T | 0.849277 | 0.027079 | 0.006442 | 2.60E-05 | 0.98567 | 0.839352 |
| Father's age at death | rs2394976 | 6:31311912_G_T | 0.848213 | 0.026997 | 0.006421 | 2.60E-05 | 0.9877 | 0.582989 |
| Father's age at death | rs3131618 | 6:31434621_A_G | 0.856975 | 0.027478 | 0.006528 | 2.60E-05 | 1 | 0.732774 |
| Father's age at death | rs147997200 | 3:49726178_G_A | 0.911348 | -0.03385 | 0.008048 | 2.60E-05 | 0.9939 | 0.067562 |
| Father's age at death | rs28656351 | 3:29855885_T_G | 0.930423 | -0.03789 | 0.009022 | 2.70E-05 | 0.99171 | 0.964513 |
| Father's age at death | rs2854011 | 6:31311211_G_A | 0.849024 | 0.027066 | 0.006442 | 2.70E-05 | 0.984 | 0.874881 |
| Father's age at death | rs9273097 | 6:32612548_A_G | 0.648162 | 0.021389 | 0.005097 | 2.70E-05 | 0.88238 | 0.849486 |
| Father's age at death | rs6082418 | 20:2197401_T_G | 0.476532 | 0.019396 | 0.004621 | 2.70E-05 | 0.98957 | 0.038001 |
| Father's age at death | rs111218728 | 6:141003140_C_T | 0.436145 | -0.01977 | 0.004713 | 2.70E-05 | 0.95454 | 0.185178 |
| Father's age at death | rs41383244 | 2:218680591_A_G | 0.653172 | -0.02025 | 0.004824 | 2.70E-05 | 0.99398 | 0.601672 |
| Father's age at death | rs35668501 | 2:225864429_G_GTT | 0.337924 | 0.020623 | 0.004914 | 2.70E-05 | 0.9591 | 0.006501 |
| Father's age at death | rs112661155 | 2:39834510_G_A | 0.92846 | 0.03896 | 0.00928 | 2.70E-05 | 0.91202 | 0.913091 |
| Father's age at death | rs112803306 | 2:133670380_C_T | 0.98385 | -0.07702 | 0.018351 | 2.70E-05 | 0.97554 | 0.531447 |
| Father's age at death | rs572943393 | 14:24426343_T_C | 0.961974 | -0.05307 | 0.012654 | 2.70E-05 | 0.89378 | 0.843012 |
| Father's age at death | rs1456322 | 1:99519632_C_T | 0.90538 | 0.03287 | 0.007824 | 2.70E-05 | 0.99532 | 0.650354 |
| Father's age at death | rs12032498 | 1:99546987_C_T | 0.876292 | 0.029459 | 0.007023 | 2.70E-05 | 0.97301 | 0.090116 |
| Father's age at death | rs12068876 | 1:204391386_T_C | 0.758717 | 0.022638 | 0.005397 | 2.70E-05 | 0.98257 | 0.454098 |
| Father's age at death | rs10273617 | 7:132278851_A_T | 0.17414 | 0.0255 | 0.006075 | 2.70E-05 | 0.97973 | 0.059745 |
| Father's age at death | rs183436268 | 7:67736860_C_T | 0.980682 | -0.07222 | 0.017216 | 2.70E-05 | 0.92946 | 0.878211 |
| Father's age at death | rs72742587 | 9:79836755_C_T | 0.911654 | 0.033828 | 0.008065 | 2.70E-05 | 0.99838 | 0.872455 |
| Father's age at death | rs141529141 | 9:79863194_C_T | 0.91152 | 0.033837 | 0.008061 | 2.70E-05 | 0.99763 | 0.816866 |
| Father's age at death | rs113423650 | 9:79876559_G_A | 0.911659 | 0.033828 | 0.008062 | 2.70E-05 | 0.99915 | 0.844402 |
| Father's age at death | rs72738736 | 15:78765122_G_T | 0.669121 | 0.020444 | 0.004874 | 2.70E-05 | 0.99752 | 0.31521 |
| Father's age at death | rs57999695 | 5:2571622_A_G | 0.984896 | -0.07921 | 0.018853 | 2.70E-05 | 0.98771 | 0.627524 |
| Father's age at death | rs118162937 | 5:2586911_G_C | 0.984797 | -0.0791 | 0.018837 | 2.70E-05 | 0.98353 | 0.441184 |
| Father's age at death | rs17723920 | 9:79834453_C_G | 0.911435 | 0.033785 | 0.008058 | 2.80E-05 | 0.99753 | 0.816992 |
| Father's age at death | rs55943354 | 9:79841043_T_C | 0.911659 | 0.033802 | 0.008063 | 2.80E-05 | 0.99904 | 0.872433 |
| Father's age at death | rs72744204 | 9:79853733_T_C | 0.912365 | 0.033943 | 0.008101 | 2.80E-05 | 0.99668 | 0.871507 |
| Father's age at death | rs143141642 | 9:79859619_AT_A | 0.911664 | 0.033778 | 0.008062 | 2.80E-05 | 0.99908 | 0.844403 |
| Father's age at death | rs112234487 | 9:79881738_G_T | 0.911553 | 0.03379 | 0.00806 | 2.80E-05 | 0.99851 | 0.844555 |
| Father's age at death | rs113164026 | 9:79881773_T_C | 0.911552 | 0.033786 | 0.00806 | 2.80E-05 | 0.9985 | 0.844555 |
| Father's age at death | rs72744211 | 9:79883572_C_T | 0.911671 | 0.033786 | 0.008062 | 2.80E-05 | 0.99915 | 0.830461 |
| Father's age at death | rs72744213 | 9:79885000_T_C | 0.91164 | 0.033773 | 0.008061 | 2.80E-05 | 0.99914 | 0.844427 |
| Father's age at death | rs142351363 | 9:107719310_C_T | 0.981768 | -0.07668 | 0.018308 | 2.80E-05 | 0.86458 | 0.087821 |
| Father's age at death | rs10759101 | 9:9909726_G_A | 0.804294 | -0.0241 | 0.005756 | 2.80E-05 | 0.99522 | 0.050641 |
| Father's age at death | rs80008378 | 18:9814436_C_T | 0.985857 | 0.082706 | 0.01976 | 2.80E-05 | 0.96313 | 0.534158 |
| Father's age at death | rs77030572 | 18:9814541_G_A | 0.985855 | 0.0827 | 0.019754 | 2.80E-05 | 0.96366 | 0.534331 |
| Father's age at death | rs7181486 | 15:78741618_T_C | 0.66856 | 0.020425 | 0.004872 | 2.80E-05 | 0.99813 | 0.227987 |
| Father's age at death | rs17483929 | 15:78742376_G_A | 0.668505 | 0.020416 | 0.004871 | 2.80E-05 | 0.99843 | 0.230553 |
| Father's age at death | rs8038761 | 15:78181805_A_G | 0.851606 | -0.02707 | 0.00646 | 2.80E-05 | 0.98599 | 0.231846 |
| Father's age at death | rs142250945 | 2:129094082_T_TA | 0.985367 | -0.08093 | 0.019311 | 2.80E-05 | 0.96762 | 0.425755 |
| Father's age at death | rs9273072 | 6:32612265_C_A | 0.664466 | 0.021432 | 0.005119 | 2.80E-05 | 0.89573 | 0.760939 |
| Father's age at death | rs3132510 | 6:31172151_T_C | 0.861991 | 0.02781 | 0.006639 | 2.80E-05 | 1 | 0.568748 |
| Father's age at death | rs2523877 | 6:31010683_C_T | 0.522594 | 0.019138 | 0.004566 | 2.80E-05 | 0.99924 | 0.077002 |
| Father's age at death | rs140338175 | 6:35474831_A_G | 0.984944 | -0.08509 | 0.02031 | 2.80E-05 | 0.85281 | 0.492674 |
| Father's age at death | rs111395772 | 3:29831093_T_G | 0.928754 | -0.03767 | 0.008998 | 2.80E-05 | 0.97438 | 0.760169 |
| Father's age at death | rs62333382 | 4:164264166_A_G | 0.53277 | 0.020933 | 0.004995 | 2.80E-05 | 0.84136 | 0.598219 |
| Father's age at death | rs72732459 | 1:99487006_G_A | 0.90767 | 0.033104 | 0.007899 | 2.80E-05 | 0.99716 | 0.594525 |
| Father's age at death | rs7529074 | 1:99495599_G_A | 0.90694 | 0.032982 | 0.007873 | 2.80E-05 | 0.99647 | 0.527971 |
| Father's age at death | rs7515804 | 1:99497080_G_A | 0.906937 | 0.03299 | 0.007873 | 2.80E-05 | 0.9965 | 0.51688 |
| Father's age at death | rs2168482 | 1:99507007_C_A | 0.906312 | 0.032995 | 0.007874 | 2.80E-05 | 0.99146 | 0.709349 |
| Father's age at death | rs55861260 | 1:99517928_G_A | 0.905569 | 0.032751 | 0.007826 | 2.90E-05 | 0.99642 | 0.649771 |
| Father's age at death | rs72734406 | 1:99549304_G_A | 0.87651 | 0.029417 | 0.00704 | 2.90E-05 | 0.97011 | 0.10868 |
| Father's age at death | rs147972973 | 1:79882208_C_T | 0.974381 | 0.064022 | 0.015304 | 2.90E-05 | 0.89634 | 0.297256 |
| Father's age at death | rs6692947 | 1:187270818_A_G | 0.299547 | 0.021068 | 0.005038 | 2.90E-05 | 0.98543 | 0.047228 |
| Father's age at death | rs1426495 | 7:132281079_G_A | 0.17414 | 0.025406 | 0.006074 | 2.90E-05 | 0.97993 | 0.066865 |
| Father's age at death | rs2656052 | 15:78740932_A_C | 0.669018 | 0.020413 | 0.004879 | 2.90E-05 | 0.99596 | 0.217801 |
| Father's age at death | rs72744214 | 9:79886523_A_G | 0.911651 | 0.033727 | 0.008062 | 2.90E-05 | 0.99914 | 0.844403 |
| Father's age at death | rs72744217 | 9:79887308_A_G | 0.911638 | 0.033702 | 0.008061 | 2.90E-05 | 0.9991 | 0.85841 |
| Father's age at death | rs58139137 | 9:79977941_C_G | 0.899046 | 0.031826 | 0.007618 | 2.90E-05 | 0.99404 | 0.924317 |
| Father's age at death | rs111546663 | 9:80043087_C_T | 0.927496 | 0.037062 | 0.008856 | 2.90E-05 | 0.99155 | 0.830954 |
| Father's age at death | rs2844647 | 6:31011013_G_A | 0.522908 | 0.019095 | 0.004565 | 2.90E-05 | 1 | 0.115958 |
| Father's age at death | rs2523875 | 6:31012033_G_T | 0.52282 | 0.01909 | 0.004565 | 2.90E-05 | 0.99982 | 0.078941 |
| Father's age at death | rs4947307 | 6:31302265_A_G | 0.857437 | 0.027435 | 0.006564 | 2.90E-05 | 0.99138 | 0.555213 |
| Father's age at death | rs117988060 | 20:40558506_A_C | 0.978721 | -0.06624 | 0.015857 | 2.90E-05 | 1 | 0.628985 |
| Father's age at death | rs114212365 | 8:127022675_T_G | 0.974227 | -0.06084 | 0.014554 | 2.90E-05 | 0.98401 | 0.647321 |
| Father's age at death | rs2428481 | 6:31360663_C_T | 0.855436 | 0.027178 | 0.006511 | 3.00E-05 | 0.99767 | 0.99069 |
| Father's age at death | rs9265827 | 6:31311318_A_G | 0.849387 | 0.0269 | 0.006443 | 3.00E-05 | 0.98595 | 0.812911 |
| Father's age at death | rs2156874 | 6:31335976_G_C | 0.855527 | 0.027179 | 0.006509 | 3.00E-05 | 0.99975 | 0.806358 |
| Father's age at death | rs2844646 | 6:31012466_G_A | 0.522875 | 0.019048 | 0.004565 | 3.00E-05 | 0.99981 | 0.080928 |
| Father's age at death | rs182104808 | 6:21116197_G_C | 0.868653 | 0.028659 | 0.006864 | 3.00E-05 | 0.96976 | 0.093799 |
| Father's age at death | rs75706791 | 6:38307031_C_T | 0.989223 | 0.095518 | 0.022872 | 3.00E-05 | 0.94401 | 0.425579 |
| Father's age at death | rs9832167 | 3:29856282_T_G | 0.930076 | -0.03758 | 0.008999 | 3.00E-05 | 0.99198 | 0.876821 |
| Father's age at death | rs150265020 | 3:61832815_TATA_T | 0.973706 | 0.065005 | 0.015588 | 3.00E-05 | 0.83357 | 0.033551 |
| Father's age at death | rs71298647 | 3:63333864_A_T | 0.929748 | 0.037335 | 0.008953 | 3.00E-05 | 1 | 0.427868 |
| Father's age at death | rs72844755 | 2:133461865_A_C | 0.922241 | -0.03569 | 0.008558 | 3.00E-05 | 0.99604 | 0.675274 |
| Father's age at death | rs186670979 | 6:141003035_C_T | 0.446732 | -0.01982 | 0.004751 | 3.00E-05 | 0.93301 | 0.078127 |
| Father's age at death | rs11578754 | 1:99491426_A_G | 0.907056 | 0.032867 | 0.007873 | 3.00E-05 | 0.99767 | 0.596554 |
| Father's age at death | rs11581137 | 1:99492720_G_A | 0.906914 | 0.032807 | 0.007865 | 3.00E-05 | 0.99818 | 0.550554 |
| Father's age at death | rs11166167 | 1:99539892_G_A | 0.876331 | 0.029187 | 0.00699 | 3.00E-05 | 0.98295 | 0.109164 |
| Father's age at death | rs576838 | 4:111804002_T_C | 0.227452 | -0.0228 | 0.005461 | 3.00E-05 | 0.9988 | 0.501938 |
| Father's age at death | rs72742583 | 9:79834556_C_T | 0.911765 | 0.033686 | 0.008071 | 3.00E-05 | 0.99772 | 0.816381 |
| Father's age at death | rs72742601 | 9:79852887_C_T | 0.911802 | 0.033694 | 0.008074 | 3.00E-05 | 0.99761 | 0.872242 |
| Father's age at death | rs17422316 | 9:79864565_A_C | 0.911259 | 0.033645 | 0.008064 | 3.00E-05 | 0.99483 | 0.91509 |
| Father's age at death | rs72744208 | 9:79867510_T_C | 0.911202 | 0.03358 | 0.008048 | 3.00E-05 | 0.99812 | 0.735868 |
| Father's age at death | rs2568494 | 15:78740964_G_A | 0.668999 | 0.020365 | 0.004879 | 3.00E-05 | 0.99603 | 0.220257 |
| Father's age at death | . | 15:91428521_CT_C | 0.673184 | 0.020342 | 0.004877 | 3.00E-05 | 0.99541 | 0.331948 |
| Father's age at death | . | rs11330240 | 0.673184 | 0.020342 | 0.004877 | 3.00E-05 | 1 | 0.319713 |
| Father's age at death | rs7850092 | 9:9909119_G_A | 0.803522 | -0.02393 | 0.005733 | 3.00E-05 | 1 | 0.034626 |
| Father's age at death | rs185330447 | 17:46680018_A_G | 0.984457 | -0.07778 | 0.018664 | 3.10E-05 | 0.97749 | 0.506174 |
| Father's age at death | rs17619307 | 6:13631555_A_C | 0.937212 | -0.03921 | 0.009403 | 3.10E-05 | 1 | 0.033297 |
| Father's age at death | rs2248386 | 6:31011247_C_T | 0.522393 | 0.018991 | 0.00456 | 3.10E-05 | 1 | 0.011184 |
| Father's age at death | rs2596545 | 6:31329374_A_C | 0.855366 | 0.027101 | 0.006511 | 3.10E-05 | 0.99818 | 0.870306 |
| Father's age at death | rs114236653 | 8:127026135_C_A | 0.973686 | -0.06009 | 0.014418 | 3.10E-05 | 0.98241 | 0.614144 |
| Father's age at death | rs3856519 | 2:60167497_T_C | 0.376006 | 0.019729 | 0.004737 | 3.10E-05 | 0.99132 | 0.950968 |
| Father's age at death | rs6451824 | 5:45841630_A_G | 0.092933 | -0.03304 | 0.007927 | 3.10E-05 | 0.99163 | 0.770797 |
| Father's age at death | rs56335091 | 1:99475650_G_A | 0.907145 | 0.032832 | 0.007877 | 3.10E-05 | 0.99744 | 0.596403 |
| Father's age at death | rs17390516 | 1:99476191_G_C | 0.907095 | 0.032837 | 0.007878 | 3.10E-05 | 0.99689 | 0.596568 |
| Father's age at death | rs11583181 | 1:99478540_C_G | 0.907145 | 0.032832 | 0.007877 | 3.10E-05 | 0.99744 | 0.596403 |
| Father's age at death | rs543323178 | 1:105392325_A_G | 0.987473 | 0.096144 | 0.023091 | 3.10E-05 | 0.79253 | 0.40927 |
| Father's age at death | rs7781482 | 7:132277835_G_C | 0.17386 | 0.025322 | 0.006078 | 3.10E-05 | 0.97983 | 0.066527 |
| Father's age at death | rs1994810 | 4:55229484_G_A | 0.702275 | 0.020908 | 0.005018 | 3.10E-05 | 0.98737 | 0.158537 |
| Father's age at death | rs76194397 | 7:95173082_A_G | 0.875246 | -0.02976 | 0.007149 | 3.10E-05 | 0.93052 | 0.19602 |
| Father's age at death | rs41282399 | 4:89028544_A_C | 0.984546 | 0.077134 | 0.018553 | 3.20E-05 | 0.99338 | 0.344429 |
| Father's age at death | rs72732445 | 1:99472729_A_C | 0.907099 | 0.032773 | 0.007878 | 3.20E-05 | 0.99698 | 0.596596 |
| Father's age at death | rs17392827 | 1:99478977_A_G | 0.907132 | 0.032721 | 0.007875 | 3.20E-05 | 0.99795 | 0.572994 |
| Father's age at death | rs7872010 | 9:79999824_T_C | 0.902371 | 0.032168 | 0.007731 | 3.20E-05 | 0.99466 | 0.695248 |
| Father's age at death | rs11244738 | 10:127660351_C_T | 0.66296 | -0.0212 | 0.005096 | 3.20E-05 | 0.90604 | 0.272458 |
| Father's age at death | rs4750960 | 10:130269723_C_T | 0.512089 | -0.01908 | 0.004586 | 3.20E-05 | 0.99347 | 0.867108 |
| Father's age at death | rs56219465 | 15:78742579_A_G | 0.668254 | 0.020274 | 0.004871 | 3.20E-05 | 0.99795 | 0.230749 |
| Father's age at death | rs2656065 | 15:78750549_G_A | 0.668025 | 0.020273 | 0.004871 | 3.20E-05 | 0.99721 | 0.322389 |
| Father's age at death | rs9265990 | 6:31316448_T_A | 0.85473 | 0.026993 | 0.006495 | 3.20E-05 | 0.99804 | 0.798314 |
| Father's age at death | rs2523573 | 6:31328988_C_G | 0.855425 | 0.027086 | 0.006509 | 3.20E-05 | 0.99927 | 0.815513 |
| Father's age at death | rs2844530 | 6:31353435_C_G | 0.855766 | 0.027113 | 0.006517 | 3.20E-05 | 0.99861 | 0.725872 |
| Father's age at death | rs62403362 | 6:21009383_T_C | 0.869116 | 0.028452 | 0.006845 | 3.20E-05 | 0.97549 | 0.025829 |
| Father's age at death | rs62440287 | 6:145339646_C_T | 0.912384 | 0.034468 | 0.008289 | 3.20E-05 | 0.95932 | 0.015396 |
| Father's age at death | rs75177655 | 3:29843783_C_G | 0.930431 | -0.03761 | 0.009042 | 3.20E-05 | 0.98825 | 0.77236 |
| Father's age at death | rs13277591 | 8:3206207_C_T | 0.702947 | -0.02103 | 0.005059 | 3.20E-05 | 0.97945 | 0.333688 |
| Father's age at death | rs58111256 | 22:50981043_C_T | 0.933577 | 0.03817 | 0.009182 | 3.20E-05 | 1 | 0.83418 |
| Father's age at death | rs181621935 | 19:52151291_A_T | 0.975191 | 0.062011 | 0.014901 | 3.20E-05 | 0.97677 | 0.181673 |
| Father's age at death | rs12996739 | 2:231003780_C_T | 0.988051 | -0.10261 | 0.024675 | 3.20E-05 | 0.72891 | 0.619883 |
| Father's age at death | rs72732461 | 1:99489762_T_G | 0.906923 | 0.032668 | 0.007869 | 3.30E-05 | 0.9974 | 0.528117 |
| Father's age at death | rs72732500 | 1:99538081_G_A | 0.876141 | 0.028986 | 0.006984 | 3.30E-05 | 0.98325 | 0.11563 |
| Father's age at death | rs6843032 | 4:88975684_G_T | 0.968903 | 0.054561 | 0.013144 | 3.30E-05 | 1 | 0.475149 |
| Father's age at death | rs117894000 | 12:3720753_C_T | 0.979387 | -0.06824 | 0.016428 | 3.30E-05 | 0.96174 | 0.254314 |
| Father's age at death | rs147611419 | 10:73814074_T_C | 0.986676 | -0.09584 | 0.023066 | 3.30E-05 | 0.74928 | 0.581072 |
| Father's age at death | rs10759098 | 9:9908465_A_C | 0.802628 | -0.02382 | 0.005733 | 3.30E-05 | 0.99636 | 0.04998 |
| Father's age at death | rs61636068 | 17:78144963_C_T | 0.70133 | -0.02263 | 0.005456 | 3.40E-05 | 0.84038 | 0.830927 |
| Father's age at death | rs34990909 | 18:55202356_T_G | 0.182276 | -0.02466 | 0.005948 | 3.40E-05 | 0.9956 | 0.325402 |
| Father's age at death | rs951985 | 15:78720923_T_G | 0.670184 | 0.02026 | 0.004887 | 3.40E-05 | 0.99496 | 0.195767 |
| Father's age at death | rs72742595 | 9:79849758_G_A | 0.911884 | 0.033497 | 0.008078 | 3.40E-05 | 0.99735 | 0.830027 |
| Father's age at death | rs200638863 | 9:75738884_A_G | 0.975605 | 0.074349 | 0.017927 | 3.40E-05 | 0.68391 | 0.951527 |
| Father's age at death | rs77863365 | 19:52153382_G_A | 0.975193 | 0.061312 | 0.014799 | 3.40E-05 | 0.98976 | 0.224849 |
| Father's age at death | rs3047566 | 19:41462401_A_ACAAAGAGC | 0.387879 | -0.01953 | 0.004711 | 3.40E-05 | 0.98783 | 0.078055 |
| Father's age at death | rs118158248 | 19:30946136_G_A | 0.984908 | -0.08034 | 0.019399 | 3.40E-05 | 0.93034 | 0.127207 |
| Father's age at death | rs4566534 | 3:143391126_T_C | 0.889061 | -0.03023 | 0.007294 | 3.40E-05 | 0.99354 | 0.143245 |
| Father's age at death | rs2596565 | 6:31353329_G_A | 0.855494 | 0.026997 | 0.00651 | 3.40E-05 | 0.99934 | 0.735021 |
| Father's age at death | rs2523593 | 6:31326703_T_C | 0.855485 | 0.026961 | 0.006509 | 3.40E-05 | 0.99948 | 0.888632 |
| Father's age at death | rs2596547 | 6:31329642_C_T | 0.85543 | 0.026967 | 0.006509 | 3.40E-05 | 0.99941 | 0.815513 |
| Father's age at death | rs2523571 | 6:31329691_T_A | 0.855444 | 0.026962 | 0.006509 | 3.40E-05 | 0.99946 | 0.824571 |
| Father's age at death | rs115072796 | 2:73397085_T_C | 0.864105 | 0.028928 | 0.006975 | 3.40E-05 | 0.90891 | 0.056338 |
| Father's age at death | rs78400445 | 2:171977636_A_C | 0.798696 | -0.02379 | 0.005741 | 3.40E-05 | 0.98456 | 0.673271 |
| Father's age at death | rs11769493 | 7:132279805_T_C | 0.849017 | -0.02668 | 0.006441 | 3.40E-05 | 0.97842 | 0.216586 |
| Father's age at death | rs7414543 | 1:99441452_G_C | 0.889688 | 0.030254 | 0.007299 | 3.40E-05 | 0.99724 | 0.529091 |
| Father's age at death | rs11577812 | 1:99478227_T_G | 0.907101 | 0.032583 | 0.007877 | 3.50E-05 | 0.99721 | 0.620426 |
| Father's age at death | rs4274116 | 1:146733632_G_C | 0.353042 | -0.01981 | 0.00479 | 3.50E-05 | 0.99237 | 0.516 |
| Father's age at death | rs17356284 | 1:146748962_C_G | 0.689827 | 0.020439 | 0.004939 | 3.50E-05 | 0.99746 | 0.9089 |
| Father's age at death | rs1991313 | 7:132294607_C_T | 0.169946 | 0.025113 | 0.006072 | 3.50E-05 | 1 | 0.12813 |
| Father's age at death | rs492291 | 4:111804548_T_A | 0.227737 | -0.02259 | 0.005462 | 3.50E-05 | 0.99727 | 0.518024 |
| Father's age at death | rs79248248 | 4:113443447_C_A | 0.977663 | 0.064991 | 0.015715 | 3.50E-05 | 0.97582 | 0.296156 |
| Father's age at death | rs74861022 | 4:113445495_G_A | 0.977657 | 0.06506 | 0.015715 | 3.50E-05 | 0.97566 | 0.296162 |
| Father's age at death | rs963443 | 5:58071799_A_G | 0.399014 | -0.01928 | 0.00466 | 3.50E-05 | 1 | 0.713658 |
| Father's age at death | rs72746022 | 9:79959015_A_G | 0.901913 | 0.031946 | 0.007716 | 3.50E-05 | 0.99455 | 0.558406 |
| Father's age at death | rs10435831 | 9:9910402_G_A | 0.802416 | -0.02375 | 0.00574 | 3.50E-05 | 0.99383 | 0.067066 |
| Father's age at death | rs143919512 | 17:46749833_G_T | 0.983627 | -0.07512 | 0.01814 | 3.50E-05 | 0.97834 | 0.068992 |
| Father's age at death | rs2523584 | 6:31327890_A_G | 0.855403 | 0.026926 | 0.006509 | 3.50E-05 | 0.99932 | 0.797482 |
| Father's age at death | rs540270479 | 6:31414051_CTTTTT_C | 0.855635 | 0.026974 | 0.006521 | 3.50E-05 | 0.99176 | 0.198815 |
| Father's age at death | rs3132090 | 6:31430752_G_A | 0.85634 | 0.026959 | 0.006518 | 3.50E-05 | 0.99962 | 0.778287 |
| Father's age at death | rs2853999 | 6:31326074_A_T | 0.855583 | 0.026894 | 0.006511 | 3.60E-05 | 0.99914 | 0.962751 |
| Father's age at death | rs2523495 | 6:31377978_C_T | 0.855476 | 0.026951 | 0.006522 | 3.60E-05 | 0.99244 | 0.43429 |
| Father's age at death | . | 6:32610795_G_C | 0.726153 | 0.02177 | 0.005267 | 3.60E-05 | 0.959 | 0.00051 |
| Father's age at death | rs9273077 | 6:32612307_A_G | 0.69709 | 0.021788 | 0.005271 | 3.60E-05 | 0.89554 | 0.511705 |
| Father's age at death | rs10174929 | 2:60131533_C_G | 0.383439 | 0.019461 | 0.004713 | 3.60E-05 | 0.99507 | 0.783646 |
| Father's age at death | rs2093045 | 20:61585706_C_T | 0.355598 | 0.02037 | 0.004933 | 3.60E-05 | 0.93813 | 0.855205 |
| Father's age at death | rs6692948 | 1:187270821_A_C | 0.299477 | 0.020815 | 0.005037 | 3.60E-05 | 0.98594 | 0.042786 |
| Father's age at death | rs72732468 | 1:99497390_T_A | 0.907141 | 0.032596 | 0.007885 | 3.60E-05 | 0.9956 | 0.527254 |
| Father's age at death | rs576842 | 4:111804004_A_G | 0.227654 | -0.02254 | 0.005456 | 3.60E-05 | 1 | 0.456478 |
| Father's age at death | rs5861029 | 4:111804051_CA_C | 0.227634 | -0.02254 | 0.005457 | 3.60E-05 | 0.99947 | 0.502158 |
| Father's age at death | rs577781 | 4:111804131_A_G | 0.227654 | -0.02257 | 0.00546 | 3.60E-05 | 0.99827 | 0.512637 |
| Father's age at death | rs11063048 | 12:4341158_G_A | 0.669634 | -0.02011 | 0.004868 | 3.60E-05 | 0.99152 | 0.016792 |
| Father's age at death | rs28446119 | 15:22916559_A_T | 0.349219 | -0.02019 | 0.004885 | 3.60E-05 | 0.95977 | 0.029902 |
| Father's age at death | rs7860150 | 9:9910039_T_C | 0.803225 | -0.02367 | 0.005739 | 3.70E-05 | 0.99764 | 0.080668 |
| Father's age at death | rs117689979 | 5:2575054_G_A | 0.983238 | -0.07356 | 0.017826 | 3.70E-05 | 0.9984 | 0.726775 |
| Father's age at death | rs77315549 | 5:2575058_G_T | 0.983238 | -0.07356 | 0.017826 | 3.70E-05 | 0.9984 | 0.726775 |
| Father's age at death | rs554288859 | 10:98424703_A_ACCCACCTGACAGAGTGGGGTGGTCTCAGACCTT | 0.933046 | 0.039936 | 0.009682 | 3.70E-05 | 0.89744 | 0.341853 |
| Father's age at death | rs139859119 | 20:40554609_C_A | 0.978831 | -0.0657 | 0.015938 | 3.70E-05 | 0.99427 | 0.487809 |
| Father's age at death | rs9366770 | 6:31170228_G_C | 0.615857 | -0.0194 | 0.004706 | 3.70E-05 | 0.99526 | 0.220714 |
| Father's age at death | rs9265831 | 6:31311449_T_A | 0.850897 | 0.026605 | 0.006452 | 3.70E-05 | 0.99135 | 0.690892 |
| Father's age at death | rs3093958 | 6:31410521_A_G | 0.855002 | 0.026879 | 0.00651 | 3.70E-05 | 0.99179 | 0.268986 |
| Father's age at death | rs3094013 | 6:31434366_G_A | 0.856224 | 0.026869 | 0.006516 | 3.70E-05 | 1 | 0.851187 |
| Father's age at death | rs75304112 | 19:52152481_G_A | 0.975033 | 0.060581 | 0.014679 | 3.70E-05 | 1 | 0.184672 |
| Father's age at death | rs34536749 | 3:49803078_TA_T | 0.590598 | 0.02138 | 0.005182 | 3.70E-05 | 0.80373 | 0.900411 |
| Father's age at death | rs12621229 | 2:60167527_A_C | 0.375087 | 0.019546 | 0.004741 | 3.70E-05 | 0.99022 | 0.950922 |
| Father's age at death | rs2203550 | 2:161832500_A_G | 0.0364586 | -0.05075 | 0.012306 | 3.70E-05 | 0.98566 | 0.901482 |
| Father's age at death | rs13268750 | 8:3199607_C_T | 0.643642 | -0.01979 | 0.004799 | 3.70E-05 | 0.98861 | 0.984955 |
| Father's age at death | rs4285153 | 4:111778733_A_G | 0.223383 | -0.0227 | 0.005501 | 3.70E-05 | 0.99649 | 0.684353 |
| Father's age at death | rs56246631 | 1:99510445_G_A | 0.906543 | 0.03251 | 0.007877 | 3.70E-05 | 0.99293 | 0.721622 |
| Father's age at death | rs12091826 | 1:66008158_A_T | 0.528275 | -0.01913 | 0.004645 | 3.80E-05 | 0.97286 | 0.866756 |
| Father's age at death | rs4956487 | 4:141783251_A_T | 0.149705 | 0.026476 | 0.006432 | 3.80E-05 | 0.99553 | 0.927979 |
| Father's age at death | rs139519547 | 4:45191296_G_A | 0.986573 | -0.09316 | 0.02263 | 3.80E-05 | 0.77347 | 1 |
| Father's age at death | rs75495877 | 9:28032863_C_A | 0.980192 | 0.068078 | 0.016531 | 3.80E-05 | 0.98723 | 0.764962 |
| Father's age at death | rs117976681 | 17:9825332_T_C | 0.977938 | -0.0641 | 0.015553 | 3.80E-05 | 1 | 0.588695 |
| Father's age at death | rs138874249 | 15:78477394_GGA_G | 0.234283 | -0.02306 | 0.005599 | 3.80E-05 | 0.92455 | 0.443874 |
| Father's age at death | rs76423146 | 8:19820480_C_T | 0.972496 | 0.058138 | 0.014109 | 3.80E-05 | 0.98117 | 0.95704 |
| Father's age at death | rs11581280 | 1:242823765_C_G | 0.794311 | 0.023406 | 0.005679 | 3.80E-05 | 0.99235 | 0.859627 |
| Father's age at death | rs4062238 | 19:41451576_A_G | 0.609757 | 0.019366 | 0.004701 | 3.80E-05 | 0.99283 | 0.385869 |
| Father's age at death | rs2844531 | 6:31353171_A_G | 0.85542 | 0.026821 | 0.006511 | 3.80E-05 | 1 | 0.332859 |
| Father's age at death | rs6457301 | 6:30998879_T_C | 0.671491 | -0.02004 | 0.004868 | 3.80E-05 | 1 | 0.671151 |
| Father's age at death | rs8118931 | 20:61553595_T_C | 0.662613 | -0.02031 | 0.00493 | 3.80E-05 | 0.96321 | 0.907612 |
| Father's age at death | rs6108784 | 20:10964366_T_C | 0.583296 | 0.019286 | 0.004683 | 3.80E-05 | 0.98569 | 0.232632 |
| Father's age at death | rs6108787 | 20:10967214_T_G | 0.519848 | 0.019006 | 0.004614 | 3.80E-05 | 0.99077 | 0.120163 |
| Father's age at death | rs4566899 | 6:141016002_A_T | 0.41879 | -0.01919 | 0.004661 | 3.80E-05 | 0.98533 | 0.095984 |
| Father's age at death | rs74967732 | 3:29843833_G_A | 0.931147 | -0.03755 | 0.009123 | 3.80E-05 | 0.9804 | 0.736088 |
| Father's age at death | rs368898569 | 2:46728053_TTC_T | 0.930862 | 0.051079 | 0.0124 | 3.80E-05 | 0.52649 | 0.636576 |
| Father's age at death | rs55780214 | 8:116655078_T_A | 0.708719 | -0.02084 | 0.005069 | 3.90E-05 | 0.98614 | 0.758735 |
| Father's age at death | rs144144720 | 20:10984418_T_TATAAAAAA | 0.717275 | 0.021124 | 0.005137 | 3.90E-05 | 0.9818 | 0.412572 |
| Father's age at death | . | 20:11681093_A_ACAAAC | 0.838522 | -0.02864 | 0.006968 | 3.90E-05 | 0.98573 | 0.507072 |
| Father's age at death | . | 20:11681093_A_C | 0.838522 | -0.02864 | 0.006968 | 3.90E-05 | 0.7926 | 0.432803 |
| Father's age at death | rs78571229 | 20:40552060_G_A | 0.978804 | -0.06549 | 0.015912 | 3.90E-05 | 0.99607 | 0.5325 |
| Father's age at death | rs3130907 | 6:31431813_A_G | 0.856224 | 0.026794 | 0.006516 | 3.90E-05 | 0.99996 | 0.796451 |
| Father's age at death | rs3128986 | 6:31433693_T_C | 0.856224 | 0.026813 | 0.006516 | 3.90E-05 | 0.99989 | 0.778413 |
| Father's age at death | rs9272674 | 6:32608885_C_T | 0.722467 | 0.021636 | 0.005258 | 3.90E-05 | 0.94599 | 0.480684 |
| Father's age at death | rs201098770 | 6:20766161_G_GAAGAT | 0.918534 | 0.036619 | 0.008896 | 3.90E-05 | 0.88302 | 0.714727 |
| Father's age at death | rs200883629 | 6:141002885_G_A | 0.430877 | -0.01935 | 0.004701 | 3.90E-05 | 0.9621 | 0.15476 |
| Father's age at death | rs13230417 | 7:132288756_C_T | 0.849816 | -0.02637 | 0.006416 | 3.90E-05 | 0.99076 | 0.33752 |
| Father's age at death | rs113822702 | 4:89023515_C_T | 0.98451 | 0.076263 | 0.01853 | 3.90E-05 | 0.99338 | 0.345947 |
| Father's age at death | rs78781548 | 16:73449846_C_G | 0.984162 | -0.07705 | 0.018729 | 3.90E-05 | 0.95802 | 0.926537 |
| Father's age at death | rs4115271 | 4:111783779_G_A | 0.223449 | -0.02263 | 0.0055 | 3.90E-05 | 0.99662 | 0.690526 |
| Father's age at death | rs34323931 | 15:64068236_C_CA | 0.537796 | -0.02171 | 0.005277 | 3.90E-05 | 0.75605 | 0.803038 |
| Father's age at death | rs1894400 | 15:91428955_C_T | 0.673441 | 0.020035 | 0.004874 | 3.90E-05 | 0.99755 | 0.348329 |
| Father's age at death | rs4932373 | 15:91429287_A_C | 0.672669 | 0.020256 | 0.004923 | 3.90E-05 | 0.97726 | 0.439491 |
| Father's age at death | rs577620809 | 13:93588700_G_GCTCTTTTGC | 0.985359 | -0.08763 | 0.021295 | 3.90E-05 | 0.79798 | 0.689684 |
| Father's age at death | rs76670417 | 5:2575032_T_G | 0.983255 | -0.07335 | 0.017836 | 3.90E-05 | 0.99824 | 0.726558 |
| Father's age at death | rs201024533 | 12:100402423_TTTAA_T | 0.973521 | 0.060414 | 0.014694 | 3.90E-05 | 0.93854 | 1 |
| Father's age at death | rs143916026 | 9:79829774_G_GA | 0.911985 | 0.033239 | 0.008081 | 3.90E-05 | 0.99763 | 0.857934 |
| Father's age at death | rs8040868 | 15:78911181_T_C | 0.603145 | 0.019255 | 0.004685 | 4.00E-05 | 1 | 0.264606 |
| Father's age at death | rs34573245 | 15:78915370_CT_C | 0.594562 | 0.019245 | 0.004686 | 4.00E-05 | 0.99047 | 0.675103 |
| Father's age at death | rs33985722 | 15:22920355_TATGC_T | 0.349145 | -0.01976 | 0.004811 | 4.00E-05 | 0.98976 | 0.0403 |
| Father's age at death | rs11244772 | 10:127711296_A_G | 0.671935 | -0.02011 | 0.004898 | 4.00E-05 | 0.98991 | 0.73855 |
| Father's age at death | rs12774026 | 10:46100529_C_A | 0.981981 | -0.07204 | 0.017535 | 4.00E-05 | 0.96114 | 0.685836 |
| Father's age at death | rs138916858 | 19:52151100_T_G | 0.974919 | 0.060992 | 0.014844 | 4.00E-05 | 0.97375 | 0.207076 |
| Father's age at death | rs9272669 | 6:32608816_A_G | 0.740837 | 0.02224 | 0.005411 | 4.00E-05 | 0.93649 | 0.151227 |
| Father's age at death | rs3131619 | 6:31434331_A_T | 0.856346 | 0.026789 | 0.006519 | 4.00E-05 | 0.99934 | 0.733635 |
| Father's age at death | rs6457300 | 6:30998742_T_G | 0.671375 | -0.02 | 0.004868 | 4.00E-05 | 1 | 0.719449 |
| Father's age at death | rs10807077 | 6:31001467_A_G | 0.67221 | -0.02 | 0.00487 | 4.00E-05 | 1 | 0.619213 |
| Father's age at death | rs2508015 | 6:31010200_G_A | 0.598418 | 0.01915 | 0.004664 | 4.00E-05 | 1 | 0.643976 |
| Father's age at death | rs7676584 | 4:88985045_T_A | 0.968972 | 0.054267 | 0.013208 | 4.00E-05 | 0.99274 | 0.445113 |
| Father's age at death | rs4472805 | 1:146733649_A_G | 0.352146 | -0.0196 | 0.004774 | 4.00E-05 | 0.9998 | 0.487394 |
| Father's age at death | rs7515995 | 1:99519111_C_T | 0.201034 | -0.02342 | 0.005704 | 4.00E-05 | 0.99593 | 0.152283 |
| Father's age at death | rs113077626 | 4:111346101_GA_G | 0.934819 | 0.039971 | 0.00974 | 4.10E-05 | 0.90671 | 0.758403 |
| Father's age at death | rs10235019 | 7:107477440_G_T | 0.540879 | -0.01914 | 0.004662 | 4.10E-05 | 0.96528 | 0.205802 |
| Father's age at death | rs118046464 | 9:28026777_T_G | 0.980121 | 0.067596 | 0.016477 | 4.10E-05 | 0.99088 | 0.823506 |
| Father's age at death | rs2394981 | 6:31313029_G_T | 0.851782 | 0.026484 | 0.00646 | 4.10E-05 | 0.99456 | 0.515217 |
| Father's age at death | rs77121684 | 20:40553415_G_A | 0.978925 | -0.06552 | 0.015967 | 4.10E-05 | 0.99448 | 0.443588 |
| Father's age at death | rs17001607 | 22:50981053_A_T | 0.933773 | 0.037757 | 0.009201 | 4.10E-05 | 0.99829 | 0.907169 |
| Father's age at death | rs192376327 | 19:401900_G_A | 0.989623 | 0.097277 | 0.023721 | 4.10E-05 | 0.90673 | 1 |
| Father's age at death | rs185611121 | 19:401901_C_A | 0.989623 | 0.097277 | 0.023721 | 4.10E-05 | 0.90673 | 1 |
| Father's age at death | rs2251396 | 6:31364707_G_A | 0.662403 | 0.019826 | 0.004841 | 4.20E-05 | 1 | 0.665626 |
| Father's age at death | rs549219764 | 6:31415876_TG_T | 0.694801 | 0.020319 | 0.004962 | 4.20E-05 | 0.99956 | 0.495621 |
| Father's age at death | rs4535568 | 6:141005059_G_A | 0.418542 | -0.0191 | 0.004662 | 4.20E-05 | 0.98486 | 0.087943 |
| Father's age at death | rs6941828 | 6:79370812_C_G | 0.50089 | -0.0189 | 0.004612 | 4.20E-05 | 0.98733 | 0.077625 |
| Father's age at death | rs4875661 | 8:3207196_T_A | 0.665375 | -0.02005 | 0.004892 | 4.20E-05 | 0.98198 | 0.654983 |
| Father's age at death | rs74779858 | 8:19828835_C_T | 0.972386 | 0.057377 | 0.014003 | 4.20E-05 | 0.98158 | 0.000578 |
| Father's age at death | rs17001608 | 22:50981326_C_T | 0.934442 | 0.037936 | 0.009261 | 4.20E-05 | 0.9949 | 0.981226 |
| Father's age at death | rs117767767 | 20:40548889_G_A | 0.978831 | -0.06519 | 0.015915 | 4.20E-05 | 0.99639 | 0.40605 |
| Father's age at death | rs1887320 | 20:10965998_G_A | 0.520819 | 0.018894 | 0.004614 | 4.20E-05 | 0.99048 | 0.15021 |
| Father's age at death | rs75845755 | 3:29874997_A_G | 0.931298 | -0.03716 | 0.009068 | 4.20E-05 | 0.99344 | 0.892621 |
| Father's age at death | rs201452139 | 3:49862148_TCAA_T | 0.95431 | -0.04608 | 0.011245 | 4.20E-05 | 0.94462 | 0.369598 |
| Father's age at death | rs10235033 | 7:107477472_G_A | 0.503875 | -0.0191 | 0.004666 | 4.20E-05 | 0.95901 | 0.223676 |
| Father's age at death | rs6593752 | 1:146729968_T_C | 0.352132 | -0.01957 | 0.004774 | 4.20E-05 | 1 | 0.483448 |
| Father's age at death | rs10984328 | 9:121705002_G_T | 0.883129 | 0.029197 | 0.007125 | 4.20E-05 | 1 | 0.594497 |
| Father's age at death | rs2938670 | 15:78740688_T_G | 0.668247 | 0.019948 | 0.004872 | 4.20E-05 | 0.99755 | 0.230738 |
| Father's age at death | rs62330868 | 5:2577264_C_T | 0.983226 | -0.07299 | 0.01781 | 4.20E-05 | 0.9994 | 0.726926 |
| Father's age at death | rs11063059 | 12:4350185_A_C | 0.660325 | -0.01995 | 0.004878 | 4.30E-05 | 0.97409 | 0.037342 |
| Father's age at death | rs11244750 | 10:127673877_C_T | 0.669517 | -0.02005 | 0.004902 | 4.30E-05 | 0.98701 | 0.368205 |
| Father's age at death | rs540622205 | 6:145328627_G_GT | 0.912577 | 0.034271 | 0.008376 | 4.30E-05 | 0.9423 | 0.006611 |
| Father's age at death | rs3132089 | 6:31430010_G_A | 0.856208 | 0.026655 | 0.006515 | 4.30E-05 | 0.99993 | 0.769449 |
| Father's age at death | rs3099839 | 6:31430065_C_T | 0.856207 | 0.026648 | 0.006515 | 4.30E-05 | 0.99994 | 0.769449 |
| Father's age at death | rs9265946 | 6:31315138_G_A | 0.854385 | 0.026579 | 0.006501 | 4.30E-05 | 0.99539 | 0.834724 |
| Father's age at death | rs34657736 | 2:144234252_A_G | 0.766474 | 0.022116 | 0.005408 | 4.30E-05 | 0.99802 | 0.458818 |
| Father's age at death | rs12701347 | 7:34201438_A_G | 0.985155 | -0.08707 | 0.021278 | 4.30E-05 | 0.78503 | 0.167467 |
| Father's age at death | rs561625712 | 1:1488398_CA_C | 0.619293 | 0.02261 | 0.005526 | 4.30E-05 | 0.72575 | 0.897824 |
| Father's age at death | rs17013808 | 4:89004957_G_A | 0.983231 | 0.073655 | 0.017996 | 4.30E-05 | 0.9748 | 0.430665 |
| Father's age at death | rs62465218 | 7:132294312_C_A | 0.851381 | -0.0263 | 0.006432 | 4.30E-05 | 0.99454 | 0.36271 |
| Father's age at death | rs114469351 | 4:137107142_T_A | 0.978579 | -0.06473 | 0.015815 | 4.30E-05 | 1 | 0.62513 |
| Father's age at death | rs138858569 | 5:107068580_T_A | 0.988129 | 0.0912 | 0.022288 | 4.30E-05 | 0.89515 | 0.21968 |
| Father's age at death | rs1875479 | 5:169510967_G_A | 0.924882 | -0.03556 | 0.008688 | 4.30E-05 | 0.99437 | 0.468727 |
| Father's age at death | rs201732208 | 1:55490861_T_C | 0.746669 | -0.02162 | 0.00529 | 4.40E-05 | 0.98468 | 0.09583 |
| Father's age at death | rs7519804 | 1:146729573_C_A | 0.352434 | -0.01951 | 0.004776 | 4.40E-05 | 0.99867 | 0.51568 |
| Father's age at death | rs10793656 | 1:146751272_T_C | 0.688029 | 0.020152 | 0.004931 | 4.40E-05 | 0.99838 | 0.96254 |
| Father's age at death | rs10493907 | 1:99516241_C_G | 0.834276 | 0.025099 | 0.006147 | 4.40E-05 | 0.99697 | 0.186488 |
| Father's age at death | rs77909876 | 7:128023128_G_A | 0.971024 | -0.05634 | 0.013791 | 4.40E-05 | 0.98053 | 0.470197 |
| Father's age at death | rs12523414 | 5:58064794_C_T | 0.392771 | -0.01915 | 0.00469 | 4.40E-05 | 0.99243 | 0.672001 |
| Father's age at death | rs3866825 | 4:111784832_G_A | 0.227143 | -0.02232 | 0.005466 | 4.40E-05 | 0.99816 | 0.44088 |
| Father's age at death | rs55994233 | 4:169861339_G_A | 0.982238 | 0.071517 | 0.017509 | 4.40E-05 | 0.97165 | 0.084217 |
| Father's age at death | rs150898062 | 4:169887649_G_A | 0.981952 | 0.070617 | 0.01729 | 4.40E-05 | 0.98122 | 0.104894 |
| Father's age at death | rs7038457 | 9:91613066_T_C | 0.908897 | -0.0325 | 0.007957 | 4.40E-05 | 0.99321 | 0.231804 |
| Father's age at death | rs5790624 | 11:27068465_A_ATG | 0.0731855 | 0.042334 | 0.010368 | 4.40E-05 | 0.71946 | 0.702716 |
| Father's age at death | rs79087984 | 18:5374273_C_T | 0.989374 | -0.09797 | 0.023972 | 4.40E-05 | 0.86223 | 0.211939 |
| Father's age at death | rs113867469 | 18:43417782_C_A | 0.964091 | 0.053218 | 0.013033 | 4.40E-05 | 0.88889 | 0.362577 |
| Father's age at death | rs4773732 | 13:93976726_A_T | 0.792076 | 0.023258 | 0.005693 | 4.40E-05 | 0.97975 | 0.902666 |
| Father's age at death | rs5025201 | 2:144206076_T_G | 0.765766 | 0.022094 | 0.005407 | 4.40E-05 | 0.9961 | 0.464665 |
| Father's age at death | rs11888341 | 2:60131753_C_T | 0.383513 | 0.019257 | 0.004712 | 4.40E-05 | 0.99495 | 0.85 |
| Father's age at death | rs3132474 | 6:31309785_G_A | 0.850651 | 0.026372 | 0.006452 | 4.40E-05 | 0.99055 | 0.699649 |
| Father's age at death | rs3094605 | 6:31430694_G_C | 0.856234 | 0.026624 | 0.006516 | 4.40E-05 | 1 | 0.796425 |
| Father's age at death | rs3132619 | 6:30374211_A_G | 0.867288 | 0.027703 | 0.00678 | 4.40E-05 | 0.99082 | 0.643295 |
| Father's age at death | rs6137362 | 20:2201540_G_T | 0.581929 | -0.01916 | 0.004688 | 4.40E-05 | 0.98494 | 0.141778 |
| Father's age at death | rs78394124 | 20:40552142_T_C | 0.978851 | -0.06512 | 0.015942 | 4.40E-05 | 0.99448 | 0.487125 |
| Father's age at death | rs12729667 | 1:242822941_G_A | 0.79424 | 0.023181 | 0.005677 | 4.40E-05 | 0.99255 | 0.797682 |
| Father's age at death | rs12750493 | 1:242832399_G_A | 0.84535 | 0.025803 | 0.006325 | 4.50E-05 | 1 | 1 |
| Father's age at death | rs11591075 | 1:242836776_G_C | 0.790298 | 0.023048 | 0.005648 | 4.50E-05 | 0.98958 | 0.688525 |
| Father's age at death | rs75017343 | 2:144659699_G_A | 0.984945 | 0.076491 | 0.018742 | 4.50E-05 | 1 | 0.143113 |
| Father's age at death | rs9616866 | 22:50978649_C_G | 0.933985 | 0.037697 | 0.009241 | 4.50E-05 | 0.99255 | 1 |
| Father's age at death | rs3132472 | 6:31386131_G_A | 0.855297 | 0.026609 | 0.006517 | 4.50E-05 | 0.99298 | 0.462679 |
| Father's age at death | rs139158380 | 1:99433161_CTTAA_C | 0.887268 | 0.029555 | 0.007241 | 4.50E-05 | 0.99482 | 0.645996 |
| Father's age at death | rs4404867 | 7:158629779_C_A | 0.896457 | 0.03077 | 0.007539 | 4.50E-05 | 0.99147 | 0.576501 |
| Father's age at death | rs4728019 | 7:79773243_C_T | 0.472797 | -0.01903 | 0.004667 | 4.50E-05 | 0.96278 | 0.388705 |
| Father's age at death | rs62639488 | 5:2585750_A_T | 0.983241 | -0.07278 | 0.01783 | 4.50E-05 | 0.99809 | 0.600399 |
| Father's age at death | rs9410438 | 9:91622691_A_G | 0.909209 | -0.03243 | 0.00795 | 4.50E-05 | 0.99772 | 0.198103 |
| Father's age at death | rs559668099 | 10:6161043_T_G | 0.845736 | 0.029526 | 0.007234 | 4.50E-05 | 0.76771 | 0.407418 |
| Father's age at death | . | 11:30367759_TAAAAAAAA_T | 0.780931 | -0.02322 | 0.0057 | 4.60E-05 | 0.94006 | 0.87317 |
| Father's age at death | rs944484 | 9:91629040_C_A | 0.0872765 | 0.033142 | 0.008128 | 4.60E-05 | 0.98981 | 0.320967 |
| Father's age at death | rs75106474 | 13:44922345_G_A | 0.987824 | -0.0909 | 0.022305 | 4.60E-05 | 0.87574 | 0.715435 |
| Father's age at death | rs35124629 | 15:99482965_C_T | 0.782033 | 0.022651 | 0.005557 | 4.60E-05 | 0.99559 | 0.450474 |
| Father's age at death | rs9273028 | 6:32611792_A_G | 0.712869 | 0.021484 | 0.005273 | 4.60E-05 | 0.91449 | 0.010271 |
| Father's age at death | rs73210448 | 8:19107387_A_G | 0.956899 | -0.04821 | 0.011828 | 4.60E-05 | 0.9113 | 0.59826 |
| Father's age at death | rs155327 | 8:3193091_A_C | 0.299026 | 0.020488 | 0.005031 | 4.60E-05 | 0.98628 | 0.5223 |
| Father's age at death | rs73196883 | 8:10860265_G_A | 0.917669 | 0.036182 | 0.008883 | 4.60E-05 | 0.8668 | 0.000583 |
| Father's age at death | rs145121879 | 22:50980173_TTTTG_T | 0.933872 | 0.037557 | 0.009216 | 4.60E-05 | 0.99626 | 0.981372 |
| Father's age at death | rs141816490 | 20:40556102_T_A | 0.978876 | -0.0649 | 0.015921 | 4.60E-05 | 0.99819 | 0.48653 |
| Father's age at death | rs117548524 | 20:40561205_T_A | 0.979085 | -0.0655 | 0.016069 | 4.60E-05 | 0.98971 | 0.482259 |
| Father's age at death | rs4546601 | 7:132283100_A_C | 0.167294 | 0.025151 | 0.006171 | 4.60E-05 | 0.97996 | 0.062849 |
| Father's age at death | rs12641800 | 4:55232262_A_G | 0.70329 | 0.020528 | 0.005039 | 4.60E-05 | 0.98078 | 0.142023 |
| Father's age at death | rs12046217 | 1:99522014_C_T | 0.834097 | 0.025036 | 0.006141 | 4.60E-05 | 0.99814 | 0.173356 |
| Father's age at death | rs113079049 | 1:146728013_CTT_C | 0.687459 | 0.020113 | 0.004936 | 4.60E-05 | 0.99511 | 0.951897 |
| Father's age at death | rs2391973 | 1:99529337_A_C | 0.322711 | -0.01986 | 0.004881 | 4.70E-05 | 0.99591 | 0.023856 |
| Father's age at death | rs7536627 | 1:187265963_T_C | 0.294543 | 0.020507 | 0.005039 | 4.70E-05 | 0.99519 | 0.034419 |
| Father's age at death | rs62475606 | 7:158630119_C_T | 0.896488 | 0.030689 | 0.007544 | 4.70E-05 | 0.99055 | 0.565861 |
| Father's age at death | rs72740120 | 5:40082486_T_A | 0.989036 | 0.110114 | 0.027063 | 4.70E-05 | 0.66119 | 0.691767 |
| Father's age at death | rs1731282 | 7:26424419_C_T | 0.986801 | -0.08296 | 0.020392 | 4.70E-05 | 0.96188 | 0.071285 |
| Father's age at death | rs74987941 | 10:46091170_G_C | 0.982033 | -0.07089 | 0.017422 | 4.70E-05 | 0.97605 | 0.684763 |
| Father's age at death | rs1323981 | 13:93984067_A_C | 0.791743 | 0.023039 | 0.005663 | 4.70E-05 | 0.98986 | 0.827348 |
| Father's age at death | rs17192371 | 9:89172631_C_A | 0.984748 | -0.08459 | 0.020792 | 4.70E-05 | 0.80814 | 0.773945 |
| Father's age at death | rs61912077 | 12:11222703_C_G | 0.979007 | 0.066172 | 0.016267 | 4.70E-05 | 0.95951 | 0.258756 |
| Father's age at death | rs1800628 | 6:31546850_G_A | 0.860224 | 0.026872 | 0.006602 | 4.70E-05 | 0.99677 | 0.773304 |
| Father's age at death | rs6917412 | 6:20830106_T_C | 0.810812 | 0.023859 | 0.00586 | 4.70E-05 | 0.98864 | 0.145824 |
| Father's age at death | rs12194825 | 6:20835260_T_A | 0.812046 | 0.023897 | 0.005874 | 4.70E-05 | 0.98819 | 0.151517 |
| Father's age at death | rs11965800 | 6:13685551_A_C | 0.93993 | -0.03935 | 0.009666 | 4.70E-05 | 0.98525 | 0.007018 |
| Father's age at death | rs201584071 | 3:49853574_TG_T | 0.922892 | -0.03522 | 0.008651 | 4.70E-05 | 0.97685 | 0.04573 |
| Father's age at death | rs1372634 | 8:3206284_G_C | 0.674007 | -0.02004 | 0.004925 | 4.70E-05 | 0.98229 | 0.458437 |
| Father's age at death | rs10181358 | 2:36999437_C_T | 0.291819 | -0.02152 | 0.00529 | 4.70E-05 | 0.90432 | 0.670239 |
| Father's age at death | rs77368741 | 2:79064150_C_T | 0.96987 | 0.054512 | 0.013401 | 4.70E-05 | 1 | 0.352257 |
| Father's age at death | rs74602614 | 2:112582523_G_A | 0.659691 | 0.019741 | 0.004851 | 4.70E-05 | 0.99183 | 0.418447 |
| Father's age at death | rs77402594 | 2:112612637_C_A | 0.659161 | 0.019768 | 0.004857 | 4.70E-05 | 0.98839 | 0.37246 |
| Father's age at death | rs75145714 | 2:161845453_T_C | 0.96551 | 0.051037 | 0.012548 | 4.80E-05 | 1 | 0.726106 |
| Father's age at death | rs1634776 | 6:31308988_C_T | 0.851349 | 0.02625 | 0.006458 | 4.80E-05 | 0.99203 | 0.801979 |
| Father's age at death | rs6570409 | 6:141015483_C_A | 0.419024 | -0.01897 | 0.004665 | 4.80E-05 | 0.98332 | 0.077553 |
| Father's age at death | rs61839730 | 1:242839751_G_A | 0.790892 | 0.023066 | 0.005676 | 4.80E-05 | 0.98182 | 0.70081 |
| Father's age at death | rs3289 | 8:19823192_T_C | 0.972593 | 0.05745 | 0.01413 | 4.80E-05 | 0.98118 | 1 |
| Father's age at death | rs112944764 | 14:24426320_C_G | 0.960449 | -0.05045 | 0.012406 | 4.80E-05 | 0.89533 | 0.819164 |
| Father's age at death | rs1496091 | 1:187287241_T_G | 0.306744 | 0.020292 | 0.00499 | 4.80E-05 | 0.99166 | 0.035067 |
| Father's age at death | rs11239963 | 1:146739068_T_C | 0.688679 | 0.02005 | 0.004931 | 4.80E-05 | 0.99933 | 0.973206 |
| Father's age at death | rs11239984 | 1:146791729_G_A | 0.689014 | 0.020048 | 0.004932 | 4.80E-05 | 1 | 0.866555 |
| Father's age at death | rs116030389 | 1:105239370_A_C | 0.978357 | 0.064815 | 0.01594 | 4.80E-05 | 0.96881 | 0.378016 |
| Father's age at death | rs11063058 | 12:4349995_C_G | 0.66739 | -0.01992 | 0.004898 | 4.80E-05 | 0.97646 | 0.023041 |
| Father's age at death | rs11635251 | 15:99480671_A_G | 0.781052 | 0.022547 | 0.005546 | 4.80E-05 | 0.99627 | 0.462136 |
| Father's age at death | rs34029266 | 15:91429196_CCA_C | 0.673025 | 0.019794 | 0.004867 | 4.80E-05 | 0.99944 | 0.256838 |
| Father's age at death | rs7984095 | 13:93984669_G_T | 0.791427 | 0.022993 | 0.005656 | 4.80E-05 | 0.99077 | 0.889113 |
| Father's age at death | rs62330862 | 5:2570584_C_T | 0.983336 | -0.07268 | 0.017877 | 4.80E-05 | 0.99838 | 0.929981 |
| Father's age at death | rs12269763 | 11:78496828_T_C | 0.975226 | 0.061221 | 0.015067 | 4.80E-05 | 0.95639 | 0.765967 |
| Father's age at death | rs56282439 | 17:78155004_G_A | 0.911069 | -0.0327 | 0.008047 | 4.80E-05 | 1 | 0.218565 |
| Father's age at death | rs4742637 | 9:9910539_T_A | 0.803737 | -0.02335 | 0.005743 | 4.80E-05 | 0.99812 | 0.074114 |
| Father's age at death | ss1388097078 | 12:9345380_T_G | 0.986428 | -0.08191 | 0.020171 | 4.90E-05 | 0.95668 | 0.23252 |
| Father's age at death | rs72730871 | 9:80065119_T_C | 0.929166 | 0.036512 | 0.008997 | 4.90E-05 | 0.98072 | 0.420078 |
| Father's age at death | rs77334359 | 10:8453167_G_T | 0.902252 | -0.03155 | 0.007765 | 4.90E-05 | 0.97814 | 0.236712 |
| Father's age at death | rs9556280 | 13:93981272_G_T | 0.792081 | 0.023038 | 0.005671 | 4.90E-05 | 0.98758 | 0.986064 |
| Father's age at death | rs9561319 | 13:93992377_T_C | 0.791864 | 0.022945 | 0.005652 | 4.90E-05 | 0.99346 | 0.965195 |
| Father's age at death | rs12729159 | 1:242835181_C_A | 0.790894 | 0.022935 | 0.005647 | 4.90E-05 | 0.99168 | 0.746621 |
| Father's age at death | rs9265979 | 6:31316044_C_T | 0.853788 | 0.026311 | 0.006481 | 4.90E-05 | 0.99909 | 0.619341 |
| Father's age at death | rs9265998 | 6:31316613_G_A | 0.853863 | 0.026333 | 0.006484 | 4.90E-05 | 0.99879 | 0.587016 |
| Father's age at death | rs10947124 | 6:31001496_G_T | 0.675828 | -0.01989 | 0.004897 | 4.90E-05 | 0.99416 | 0.519242 |
| Father's age at death | rs9273109 | 6:32612627_A_G | 0.646051 | 0.020612 | 0.005074 | 4.90E-05 | 0.88388 | 0.060104 |
| Father's age at death | rs68069111 | 6:20820391_C_A | 0.810775 | 0.023777 | 0.005857 | 4.90E-05 | 0.98921 | 0.138236 |
| Father's age at death | rs1054866 | 20:61571208_C_A | 0.309851 | 0.020105 | 0.004951 | 4.90E-05 | 0.99762 | 0.88743 |
| Father's age at death | rs149121422 | 22:24352781_A_C | 0.980062 | 0.085047 | 0.020935 | 4.90E-05 | 0.61133 | 0.465232 |
| Father's age at death | rs78707203 | 2:112610641_G_A | 0.659962 | 0.019733 | 0.004859 | 4.90E-05 | 0.98953 | 0.345017 |
| Father's age at death | rs577877070 | 6:141033361_T_TATA | 0.448731 | -0.01931 | 0.004758 | 4.90E-05 | 0.92979 | 0.099038 |
| Father's age at death | rs1365363 | 7:132283209_G_A | 0.167154 | 0.025052 | 0.006173 | 4.90E-05 | 0.97986 | 0.055731 |
| Father's age at death | rs12119390 | 1:146728149_A_G | 0.688668 | 0.019998 | 0.004932 | 5.00E-05 | 0.99908 | 0.983923 |
| Father's age at death | rs7523685 | 1:146728953_A_C | 0.688759 | 0.019995 | 0.004931 | 5.00E-05 | 0.99957 | 0.978561 |
| Father's age at death | rs1359199 | 13:93982196_A_G | 0.791374 | 0.022972 | 0.005663 | 5.00E-05 | 0.98819 | 0.889129 |
| Father's age at death | . | 15:91428521_CT_C | 0.327821 | -0.01973 | 0.004862 | 5.00E-05 | 0.99541 | 0.331948 |
| Father's age at death | . | rs11330240 | 0.327821 | -0.01973 | 0.004862 | 5.00E-05 | 1 | 0.319713 |
| Father's age at death | rs75446549 | 5:2587093_C_T | 0.983276 | -0.07261 | 0.017899 | 5.00E-05 | 0.99271 | 0.599667 |
| Father's age at death | rs62330888 | 5:2588390_G_A | 0.983276 | -0.07261 | 0.017899 | 5.00E-05 | 0.99271 | 0.599667 |
| Father's age at death | rs79555963 | 5:2588752_T_G | 0.983276 | -0.07261 | 0.017899 | 5.00E-05 | 0.99271 | 0.599667 |
| Father's age at death | rs111218866 | 6:141002653_G_A | 0.427158 | -0.01902 | 0.00469 | 5.00E-05 | 0.96786 | 0.096605 |
| Father's age at death | rs9321782 | 6:141000537_G_A | 0.418138 | -0.0189 | 0.004662 | 5.00E-05 | 0.98518 | 0.087862 |
| Father's age at death | rs4239511 | 19:41453582_C_T | 0.384761 | -0.01907 | 0.004705 | 5.00E-05 | 0.99518 | 0.222869 |
| Father's age at death | rs189936901 | 6:20766156_C_A | 0.892598 | 0.029954 | 0.00739 | 5.00E-05 | 0.99847 | 0.868768 |
| Father's age at death | rs9265974 | 6:31315879_T_G | 0.854396 | 0.026379 | 0.006506 | 5.00E-05 | 0.99452 | 0.710641 |
| Father's age at death | rs9265976 | 6:31315933_A_G | 0.853737 | 0.02628 | 0.006479 | 5.00E-05 | 0.9995 | 0.619455 |
| Father's age at death | rs2730041 | 8:3190005_A_G | 0.305478 | 0.02036 | 0.005018 | 5.00E-05 | 0.97888 | 0.698474 |
| Father's age at death | rs1019615 | 2:60131050_A_G | 0.377364 | 0.019165 | 0.004726 | 5.00E-05 | 0.99537 | 0.806041 |
| Father's age at death | rs6751255 | 2:60153321_C_G | 0.369426 | 0.019275 | 0.004756 | 5.10E-05 | 0.99205 | 0.539821 |
| Father's age at death | rs2394979 | 6:31312729_G_A | 0.853484 | 0.026231 | 0.006478 | 5.10E-05 | 0.99865 | 0.533409 |
| Father's age at death | rs9265973 | 6:31315861_G_A | 0.853798 | 0.026251 | 0.00648 | 5.10E-05 | 0.99922 | 0.62748 |
| Father's age at death | rs402175 | 6:31308717_G_A | 0.853579 | 0.026223 | 0.006477 | 5.10E-05 | 0.99923 | 0.644392 |
| Father's age at death | rs10947125 | 6:31001633_A_G | 0.672297 | -0.01974 | 0.004871 | 5.10E-05 | 0.99963 | 0.58276 |
| Father's age at death | rs10947126 | 6:31001668_G_A | 0.672283 | -0.01973 | 0.004871 | 5.10E-05 | 0.99957 | 0.578296 |
| Father's age at death | rs564818958 | 19:14158022_A_AT | 0.830033 | -0.02557 | 0.006315 | 5.10E-05 | 0.9297 | 0.774248 |
| Father's age at death | rs11239985 | 1:146793381_A_G | 0.689081 | 0.01998 | 0.004932 | 5.10E-05 | 0.99938 | 0.994636 |
| Father's age at death | rs12032298 | 1:175595129_G_T | 0.802991 | 0.023407 | 0.005778 | 5.10E-05 | 0.99341 | 0.163708 |
| Father's age at death | rs4536028 | 1:103738034_C_T | 0.160482 | 0.025464 | 0.006283 | 5.10E-05 | 0.98631 | 0.578989 |
| Father's age at death | rs6680712 | 1:99538754_A_G | 0.323098 | -0.01984 | 0.004899 | 5.10E-05 | 0.98838 | 0.026074 |
| Father's age at death | rs1329469 | 1:99427638_C_T | 0.908294 | 0.032167 | 0.007937 | 5.10E-05 | 0.99477 | 0.931204 |
| Father's age at death | rs17134378 | 7:51720942_G_T | 0.84955 | 0.026088 | 0.006437 | 5.10E-05 | 0.98857 | 0.644525 |
| Father's age at death | rs531911437 | 7:134488447_AT_A | 0.0707482 | 0.041993 | 0.010366 | 5.10E-05 | 0.73563 | 0.337598 |
| Father's age at death | rs4277843 | 4:111777749_A_G | 0.227181 | -0.02229 | 0.0055 | 5.10E-05 | 0.98591 | 0.445769 |
| Father's age at death | rs76705118 | 9:9910982_CTA_C | 0.803648 | -0.02327 | 0.005743 | 5.10E-05 | 0.99788 | 0.075683 |
| Father's age at death | rs10851907 | 15:78915864_G_A | 0.594756 | 0.01912 | 0.00472 | 5.10E-05 | 0.97667 | 0.782943 |
| Father's age at death | rs74421934 | 10:2148380_G_T | 0.925537 | 0.035571 | 0.008783 | 5.10E-05 | 0.98454 | 0.882789 |
| Father's age at death | rs11618902 | 13:93982012_G_A | 0.7919 | 0.022947 | 0.005667 | 5.10E-05 | 0.9882 | 0.979109 |
| Father's age at death | rs4773735 | 13:93986812_T_G | 0.791521 | 0.022891 | 0.005654 | 5.10E-05 | 0.99197 | 0.861597 |
| Father's age at death | rs4771870 | 13:93987755_C_T | 0.791418 | 0.022885 | 0.005653 | 5.20E-05 | 0.99191 | 0.875359 |
| Father's age at death | rs62330867 | 5:2576366_A_G | 0.983509 | -0.07291 | 0.018018 | 5.20E-05 | 0.99323 | 0.79046 |
| Father's age at death | rs7497304 | 15:91429176_G_T | 0.673035 | 0.019699 | 0.004866 | 5.20E-05 | 1 | 0.238089 |
| Father's age at death | rs6606805 | 15:22918960_G_C | 0.346033 | -0.01943 | 0.004799 | 5.20E-05 | 1 | 0.070178 |
| Father's age at death | rs9265982 | 6:31316080_C_T | 0.853683 | 0.026215 | 0.006477 | 5.20E-05 | 0.99989 | 0.611421 |
| Father's age at death | rs9265985 | 6:31316234_A_C | 0.853718 | 0.026212 | 0.006478 | 5.20E-05 | 0.99976 | 0.587314 |
| Father's age at death | rs9265993 | 6:31316520_G_A | 0.853709 | 0.026208 | 0.006478 | 5.20E-05 | 0.99979 | 0.587314 |
| Father's age at death | rs117149753 | 8:116232226_G_A | 0.96343 | 0.055159 | 0.013639 | 5.20E-05 | 0.80439 | 0.371664 |
| Father's age at death | rs12209083 | 6:141000892_A_G | 0.418233 | -0.01886 | 0.004661 | 5.20E-05 | 0.98551 | 0.085704 |
| Father's age at death | rs4566898 | 6:141005285_A_C | 0.418241 | -0.01886 | 0.004661 | 5.20E-05 | 0.98548 | 0.086796 |
| Father's age at death | rs9403202 | 6:141009653_C_T | 0.418228 | -0.01885 | 0.004661 | 5.20E-05 | 0.98574 | 0.087882 |
| Father's age at death | rs7738110 | 6:141013225_C_G | 0.418232 | -0.01886 | 0.00466 | 5.20E-05 | 0.98587 | 0.086796 |
| Father's age at death | rs6904735 | 6:141013878_A_G | 0.418222 | -0.01885 | 0.00466 | 5.20E-05 | 0.9859 | 0.086776 |
| Father's age at death | rs6910729 | 6:141014669_G_C | 0.418219 | -0.01885 | 0.00466 | 5.20E-05 | 0.98591 | 0.086776 |
| Father's age at death | rs6074226 | 20:10984609_C_G | 0.709758 | 0.020464 | 0.005056 | 5.20E-05 | 0.99679 | 0.541851 |
| Father's age at death | rs34117762 | 8:3193683_GA_G | 0.673798 | -0.01988 | 0.004915 | 5.20E-05 | 0.98507 | 0.64613 |
| Mother's age at death | rs62227724 | 22:44523907_C_G | 0.968512 | 0.092149 | 0.016689 | 3.40E-08 | 0.75641 | 0.293116 |
| Mother's age at death | rs4709783 | 6:164295151_T_C | 0.426007 | 0.028085 | 0.005161 | 5.30E-08 | 0.99244 | 0.339852 |
| Mother's age at death | rs6707813 | 2:173923137_A_G | 0.215709 | 0.031952 | 0.006202 | 2.60E-07 | 0.98945 | 0.195344 |
| Mother's age at death | rs4709021 | 6:164300258_G_A | 0.388989 | 0.026759 | 0.005247 | 3.40E-07 | 0.98667 | 0.560893 |
| Mother's age at death | rs9346978 | 6:164309315_C_T | 0.388647 | 0.026714 | 0.005258 | 3.80E-07 | 0.98331 | 0.623548 |
| Mother's age at death | rs9364708 | 6:164309316_A_G | 0.388685 | 0.026709 | 0.005258 | 3.80E-07 | 0.98315 | 0.623563 |
| Mother's age at death | . | 3:81524690_AAAAAAAAC_A | 0.902205 | 0.049498 | 0.009758 | 3.90E-07 | 0.76845 | 0.68182 |
| Mother's age at death | rs1455213 | 4:15183197_A_G | 0.403809 | 0.02659 | 0.005248 | 4.00E-07 | 0.97697 | 0.957016 |
| Mother's age at death | rs9295237 | 6:164297589_T_C | 0.389394 | 0.02638 | 0.005218 | 4.30E-07 | 0.99771 | 0.556993 |
| Mother's age at death | rs568114142 | 6:164315453_CTT_C | 0.389821 | 0.026695 | 0.005281 | 4.30E-07 | 0.97294 | 0.685078 |
| Mother's age at death | rs4709785 | 6:164298306_G_C | 0.389098 | 0.02634 | 0.005216 | 4.40E-07 | 0.99844 | 0.536723 |
| Mother's age at death | rs9365626 | 6:164301472_G_T | 0.388563 | 0.026483 | 0.005248 | 4.50E-07 | 0.98667 | 0.577178 |
| Mother's age at death | rs1809139 | 2:173982876_C_T | 0.823972 | -0.03376 | 0.006688 | 4.50E-07 | 0.99349 | 0.153649 |
| Mother's age at death | rs9346977 | 6:164302273_A_C | 0.388685 | 0.026405 | 0.005248 | 4.90E-07 | 0.9866 | 0.556711 |
| Mother's age at death | rs78664552 | 4:15176383_C_A | 0.405083 | 0.026366 | 0.005244 | 5.00E-07 | 0.97717 | 0.995227 |
| Mother's age at death | rs4709787 | 6:164300684_G_C | 0.388548 | 0.026339 | 0.005247 | 5.20E-07 | 0.98693 | 0.560725 |
| Mother's age at death | rs9458924 | 6:164304652_A_G | 0.388639 | 0.026303 | 0.005246 | 5.30E-07 | 0.98717 | 0.548567 |
| Mother's age at death | rs9346979 | 6:164309479_T_C | 0.388604 | 0.026333 | 0.00525 | 5.30E-07 | 0.9861 | 0.593854 |
| Mother's age at death | rs9295236 | 6:164297121_A_G | 0.388978 | 0.026119 | 0.005213 | 5.40E-07 | 1 | 0.54068 |
| Mother's age at death | rs4709790 | 6:164312470_G_A | 0.38873 | 0.026318 | 0.00525 | 5.40E-07 | 0.98583 | 0.602338 |
| Mother's age at death | rs3799337 | 6:164298780_G_A | 0.389171 | 0.026118 | 0.005224 | 5.70E-07 | 0.99557 | 0.56097 |
| Mother's age at death | rs10647141 | 6:164311206_T_TAC | 0.390067 | 0.026191 | 0.005253 | 6.20E-07 | 0.98331 | 0.529103 |
| Mother's age at death | rs2974238 | 19:48117061_G_A | 0.248156 | 0.029561 | 0.005929 | 6.20E-07 | 0.98773 | 0.578256 |
| Mother's age at death | rs7738627 | 6:164291369_T_G | 0.387428 | 0.026107 | 0.005242 | 6.40E-07 | 0.99038 | 0.369258 |
| Mother's age at death | rs7438047 | 4:15189913_C_A | 0.403265 | 0.026159 | 0.005259 | 6.60E-07 | 0.97365 | 0.746294 |
| Mother's age at death | rs4709792 | 6:164316375_A_G | 0.388509 | 0.026161 | 0.005268 | 6.80E-07 | 0.97926 | 0.552561 |
| Mother's age at death | . | 3:81524681_TAAAAAAA_T | 0.924683 | 0.051281 | 0.010341 | 7.10E-07 | 0.86673 | 0.63317 |
| Mother's age at death | rs731978 | 4:15194513_T_C | 0.40316 | 0.026045 | 0.005276 | 7.90E-07 | 0.96747 | 0.89987 |
| Mother's age at death | rs2011605 | 4:15190627_A_G | 0.402879 | 0.025929 | 0.005257 | 8.10E-07 | 0.97439 | 0.843224 |
| Mother's age at death | rs4709781 | 6:164293404_C_T | 0.387418 | 0.025835 | 0.005236 | 8.10E-07 | 0.99264 | 0.340855 |
| Mother's age at death | rs118142265 | 9:132194033_G_T | 0.982526 | -0.11033 | 0.022451 | 8.90E-07 | 0.74259 | 0.234812 |
| Mother's age at death | rs2913996 | 19:48116694_T_C | 0.245473 | 0.029178 | 0.00594 | 9.00E-07 | 0.99057 | 0.508475 |
| Mother's age at death | rs242402 | 14:56663760_T_G | 0.802198 | -0.03245 | 0.006611 | 9.20E-07 | 0.93526 | 0.78538 |
| Mother's age at death | rs201250103 | 2:48988722_CT_C | 0.776409 | -0.03058 | 0.006264 | 1.00E-06 | 0.93517 | 0.000208 |
| Mother's age at death | rs55922727 | 5:137402011_CA_C | 0.0288721 | -0.10643 | 0.021825 | 1.10E-06 | 0.48141 | 0.071269 |
| Mother's age at death | rs375820810 | 6:2861224_ACT_A | 0.935562 | -0.0522 | 0.01079 | 1.30E-06 | 0.91989 | 0.600302 |
| Mother's age at death | rs2913995 | 19:48117028_T_C | 0.246358 | 0.028724 | 0.005936 | 1.30E-06 | 0.98993 | 0.587043 |
| Mother's age at death | rs4972539 | 2:174107299_G_A | 0.789134 | -0.03089 | 0.006408 | 1.40E-06 | 0.94006 | 0.110002 |
| Mother's age at death | rs145455367 | 2:207654320_C_CAA | 0.928532 | 0.049273 | 0.010281 | 1.60E-06 | 0.9255 | 0.965148 |
| Mother's age at death | rs34602589 | 20:43768734_T_A | 0.81831 | 0.031763 | 0.006619 | 1.60E-06 | 0.99271 | 0.695232 |
| Mother's age at death | rs34973476 | 20:43770291_G_A | 0.818253 | 0.031669 | 0.006617 | 1.70E-06 | 0.99283 | 0.680863 |
| Mother's age at death | rs34448893 | 20:43771407_A_G | 0.818253 | 0.031669 | 0.006617 | 1.70E-06 | 0.99283 | 0.680863 |
| Mother's age at death | rs11343808 | 19:48097804_CT_C | 0.227901 | 0.03064 | 0.006409 | 1.70E-06 | 0.89748 | 0.954177 |
| Mother's age at death | rs35726706 | 2:173924662_A_G | 0.200773 | 0.030499 | 0.006378 | 1.70E-06 | 0.98805 | 0.448508 |
| Mother's age at death | rs75169866 | 3:15819505_T_C | 0.975873 | 0.080943 | 0.016927 | 1.70E-06 | 0.95708 | 0.248056 |
| Mother's age at death | rs10553010 | 2:173924663_TAG_T | 0.200839 | 0.030429 | 0.006379 | 1.80E-06 | 0.98691 | 0.454078 |
| Mother's age at death | rs17710065 | 2:174005866_C_G | 0.795087 | -0.03037 | 0.006364 | 1.80E-06 | 0.97891 | 0.482781 |
| Mother's age at death | rs150468613 | 2:207695112_TAAA_T | 0.91417 | 0.04349 | 0.009107 | 1.80E-06 | 0.99776 | 0.970635 |
| Mother's age at death | rs6032017 | 20:43768712_T_C | 0.8181 | 0.031609 | 0.006615 | 1.80E-06 | 0.99269 | 0.702781 |
| Mother's age at death | rs200606944 | 6:2861104_TTA_T | 0.926805 | -0.04719 | 0.00988 | 1.80E-06 | 0.97719 | 0.882308 |
| Mother's age at death | rs66792821 | 6:2861611_A_G | 0.926905 | -0.0471 | 0.00989 | 1.90E-06 | 0.97619 | 1 |
| Mother's age at death | rs55962025 | 4:3112109_A_C | 0.642311 | -0.02542 | 0.005337 | 1.90E-06 | 0.99317 | 0.103748 |
| Mother's age at death | rs4972529 | 2:173952115_T_C | 0.783608 | -0.02936 | 0.006181 | 2.00E-06 | 0.99562 | 0.393577 |
| Mother's age at death | rs7680032 | 4:15173351_C_T | 0.53847 | 0.024511 | 0.005162 | 2.00E-06 | 0.97728 | 0.680346 |
| Mother's age at death | rs7349694 | 4:6210296_T_C | 0.925519 | 0.045909 | 0.00966 | 2.00E-06 | 1 | 0.338361 |
| Mother's age at death | rs908017 | 4:6221614_T_G | 0.92487 | 0.045763 | 0.009619 | 2.00E-06 | 1 | 0.456552 |
| Mother's age at death | rs76108901 | 7:117674806_T_C | 0.941466 | -0.05207 | 0.010958 | 2.00E-06 | 0.97573 | 0.310084 |
| Mother's age at death | rs761500 | 20:43772272_G_A | 0.817741 | 0.031474 | 0.006614 | 2.00E-06 | 0.99122 | 0.564339 |
| Mother's age at death | rs34313230 | 20:43755858_C_G | 0.820323 | 0.031586 | 0.006659 | 2.10E-06 | 0.98807 | 0.50173 |
| Mother's age at death | rs13038485 | 20:43767759_T_A | 0.818157 | 0.031281 | 0.006593 | 2.10E-06 | 0.99935 | 0.617689 |
| Mother's age at death | rs78080193 | 7:117674084_C_A | 0.94152 | -0.05188 | 0.010946 | 2.10E-06 | 0.97883 | 0.322151 |
| Mother's age at death | rs734993 | 2:174025999_A_C | 0.819746 | -0.03137 | 0.00661 | 2.10E-06 | 0.99561 | 0.070065 |
| Mother's age at death | rs4257365 | 2:173927569_G_A | 0.801746 | -0.03041 | 0.006425 | 2.20E-06 | 0.98246 | 0.357775 |
| Mother's age at death | rs6433392 | 2:173933162_G_T | 0.854411 | -0.03429 | 0.007243 | 2.20E-06 | 0.98938 | 0.208387 |
| Mother's age at death | rs746179 | 2:174026324_A_G | 0.81941 | -0.03127 | 0.006605 | 2.20E-06 | 0.99621 | 0.078433 |
| Mother's age at death | rs34919068 | 20:43755830_C_A | 0.820166 | 0.031519 | 0.006657 | 2.20E-06 | 0.98829 | 0.502077 |
| Mother's age at death | rs13042991 | 20:43767178_A_G | 0.817947 | 0.031128 | 0.006588 | 2.30E-06 | 1 | 0.645803 |
| Mother's age at death | rs17423617 | 20:43768050_G_C | 0.818326 | 0.031189 | 0.006599 | 2.30E-06 | 0.99851 | 0.666438 |
| Mother's age at death | rs17332620 | 20:43777506_C_T | 0.818093 | 0.031114 | 0.006621 | 2.60E-06 | 0.99224 | 0.906651 |
| Mother's age at death | rs61151985 | 2:173930266_G_A | 0.853663 | -0.03393 | 0.007224 | 2.60E-06 | 0.99026 | 0.140783 |
| Mother's age at death | rs10049467 | 3:197075743_G_A | 0.987827 | -0.11324 | 0.024085 | 2.60E-06 | 0.93109 | 0.552116 |
| Mother's age at death | rs13082584 | 3:81523959_G_T | 0.909511 | 0.041857 | 0.008904 | 2.60E-06 | 0.99136 | 0.505278 |
| Mother's age at death | rs2921552 | 19:48104647_T_A | 0.26073 | 0.027788 | 0.005916 | 2.60E-06 | 0.96106 | 0.664554 |
| Mother's age at death | rs13038275 | 20:43780988_A_T | 0.818184 | 0.031069 | 0.006625 | 2.70E-06 | 0.99168 | 0.898874 |
| Mother's age at death | rs13043809 | 20:43753220_A_G | 0.817762 | 0.030906 | 0.006598 | 2.80E-06 | 0.99632 | 0.564287 |
| Mother's age at death | rs13044116 | 20:43754444_T_C | 0.817544 | 0.030872 | 0.00659 | 2.80E-06 | 0.99764 | 0.577923 |
| Mother's age at death | rs62208373 | 20:43764426_G_A | 0.817944 | 0.030898 | 0.006595 | 2.80E-06 | 0.99797 | 0.617974 |
| Mother's age at death | rs62208374 | 20:43765628_C_T | 0.817944 | 0.030898 | 0.006595 | 2.80E-06 | 0.99797 | 0.617974 |
| Mother's age at death | rs34811768 | 20:43765800_T_C | 0.818314 | 0.030948 | 0.006606 | 2.80E-06 | 0.996 | 0.631292 |
| Mother's age at death | rs34638860 | 20:43765839_C_T | 0.818337 | 0.030973 | 0.006606 | 2.80E-06 | 0.99614 | 0.638234 |
| Mother's age at death | rs7844919 | 8:2226153_C_T | 0.464963 | -0.02941 | 0.00628 | 2.80E-06 | 0.66643 | 0.021038 |
| Mother's age at death | rs2798282 | 4:3029916_A_G | 0.293684 | 0.026591 | 0.005674 | 2.80E-06 | 0.97193 | 0.021757 |
| Mother's age at death | rs113294358 | 4:3110794_AT_A | 0.652641 | -0.02515 | 0.005367 | 2.80E-06 | 0.9945 | 0.101791 |
| Mother's age at death | rs199585288 | 4:3110796_AT_A | 0.652641 | -0.02515 | 0.005367 | 2.80E-06 | 0.9945 | 0.101791 |
| Mother's age at death | rs61348208 | 4:3089564_C_T | 0.603369 | -0.02448 | 0.005231 | 2.90E-06 | 0.99116 | 0.239651 |
| Mother's age at death | rs201988934 | 19:48112137_C_CA | 0.302999 | 0.026273 | 0.005614 | 2.90E-06 | 0.9715 | 0.170569 |
| Mother's age at death | rs145583025 | 18:14443241_G_C | 0.850428 | -0.03888 | 0.008311 | 2.90E-06 | 0.74023 | 0.261981 |
| Mother's age at death | rs35882692 | 20:43763968_T_C | 0.818569 | 0.030934 | 0.006614 | 2.90E-06 | 0.99517 | 0.644926 |
| Mother's age at death | rs55648802 | 6:2856200_C_T | 0.926559 | -0.04557 | 0.009751 | 3.00E-06 | 1 | 0.784079 |
| Mother's age at death | rs34783916 | 3:81514433_T_C | 0.909328 | 0.041543 | 0.008893 | 3.00E-06 | 0.99227 | 0.506081 |
| Mother's age at death | rs7642446 | 3:81519861_G_A | 0.909303 | 0.041533 | 0.008892 | 3.00E-06 | 0.99216 | 0.495074 |
| Mother's age at death | rs62175799 | 2:173935026_G_C | 0.853288 | -0.03375 | 0.007223 | 3.00E-06 | 0.9882 | 0.187103 |
| Mother's age at death | rs60965711 | 2:207673702_A_C | 0.914833 | 0.042735 | 0.009145 | 3.00E-06 | 0.99705 | 0.911287 |
| Mother's age at death | rs59065534 | 2:207717680_TGAG_T | 0.913729 | 0.042499 | 0.009096 | 3.00E-06 | 0.99527 | 0.897931 |
| Mother's age at death | rs2099052 | 19:48110625_G_T | 0.300718 | 0.026404 | 0.005654 | 3.00E-06 | 0.96137 | 0.15439 |
| Mother's age at death | rs74174932 | 20:43756133_G_A | 0.817266 | 0.030758 | 0.006591 | 3.10E-06 | 0.99622 | 0.605299 |
| Mother's age at death | rs34622539 | 20:43756548_A_T | 0.817745 | 0.030768 | 0.006594 | 3.10E-06 | 0.99732 | 0.570874 |
| Mother's age at death | rs13100565 | 3:81510613_T_C | 0.909312 | 0.041532 | 0.0089 | 3.10E-06 | 0.99049 | 0.495074 |
| Mother's age at death | rs4498089 | 4:3080199_A_G | 0.617209 | -0.02464 | 0.005282 | 3.10E-06 | 0.98517 | 0.235863 |
| Mother's age at death | rs76229543 | 4:6224224_G_A | 0.929024 | 0.046385 | 0.009939 | 3.10E-06 | 0.9884 | 0.420543 |
| Mother's age at death | . | 3:81525853_G_GT | 0.910022 | 0.041621 | 0.008941 | 3.20E-06 | 0.98771 | 0.572902 |
| Mother's age at death | rs79941005 | 9:78502989_T_C | 0.977371 | -0.08241 | 0.017702 | 3.20E-06 | 0.92677 | 0.429181 |
| Mother's age at death | rs17332243 | 20:43756071_T_C | 0.81772 | 0.030709 | 0.006594 | 3.20E-06 | 0.99713 | 0.604589 |
| Mother's age at death | rs150432060 | 8:4087035_A_C | 0.989024 | -0.12172 | 0.02613 | 3.20E-06 | 0.87413 | 0.595909 |
| Mother's age at death | rs71339824 | 20:43790296_T_TA | 0.816372 | 0.030707 | 0.006601 | 3.30E-06 | 0.99056 | 0.861347 |
| Mother's age at death | rs34343273 | 20:43795293_G_A | 0.818957 | 0.030792 | 0.006621 | 3.30E-06 | 0.99657 | 0.992174 |
| Mother's age at death | rs57039370 | 2:207717630_A_G | 0.913103 | 0.042067 | 0.009047 | 3.30E-06 | 1 | 0.912952 |
| Mother's age at death | rs71339821 | 20:43756027_GTATA_G | 0.817611 | 0.03061 | 0.006591 | 3.40E-06 | 0.99765 | 0.597971 |
| Mother's age at death | rs74174934 | 20:43761331_T_G | 0.817655 | 0.030613 | 0.006592 | 3.40E-06 | 0.99771 | 0.604672 |
| Mother's age at death | rs17423897 | 20:43780307_T_C | 0.81845 | 0.030749 | 0.006622 | 3.40E-06 | 0.99362 | 0.937584 |
| Mother's age at death | rs55895052 | 6:2856400_G_A | 0.926761 | -0.04542 | 0.009774 | 3.40E-06 | 0.99682 | 0.983137 |
| Mother's age at death | rs12686292 | 9:78474471_A_G | 0.979769 | -0.08369 | 0.018007 | 3.40E-06 | 0.99491 | 0.018306 |
| Mother's age at death | rs76799679 | 9:78480255_T_C | 0.979779 | -0.08385 | 0.018051 | 3.40E-06 | 0.99178 | 0.046817 |
| Mother's age at death | rs117955115 | 9:78480263_C_T | 0.979781 | -0.08384 | 0.018052 | 3.40E-06 | 0.99192 | 0.046666 |
| Mother's age at death | . | 9:83163200_G_GA | 0.219693 | -0.02921 | 0.006287 | 3.40E-06 | 0.95384 | 0.334287 |
| Mother's age at death | rs56213767 | 16:15379572_A_G | 0.405955 | 0.028136 | 0.006057 | 3.40E-06 | 0.73405 | 0.697686 |
| Mother's age at death | rs62128735 | 19:48113604_G_A | 0.30449 | 0.02606 | 0.005609 | 3.40E-06 | 0.97091 | 0.213427 |
| Mother's age at death | rs116956858 | 9:78472096_G_T | 0.979763 | -0.08343 | 0.017995 | 3.50E-06 | 0.99574 | 0.012272 |
| Mother's age at death | rs10869663 | 9:78486641_T_C | 0.979705 | -0.0837 | 0.018054 | 3.50E-06 | 0.98943 | 0.092087 |
| Mother's age at death | rs144266544 | 2:173941776_TAATG_T | 0.835123 | -0.03193 | 0.006881 | 3.50E-06 | 0.98895 | 0.107892 |
| Mother's age at death | rs112745132 | 7:117655836_G_T | 0.943077 | -0.05093 | 0.01098 | 3.50E-06 | 0.99802 | 0.393206 |
| Mother's age at death | rs35434469 | 20:43778544_C_T | 0.818533 | 0.030711 | 0.006623 | 3.50E-06 | 0.99347 | 0.914227 |
| Mother's age at death | rs35615384 | 20:43787352_AT_A | 0.818874 | 0.03065 | 0.006611 | 3.50E-06 | 0.99869 | 0.91412 |
| Mother's age at death | rs17423416 | 20:43758144_G_T | 0.817584 | 0.030556 | 0.006594 | 3.60E-06 | 0.99675 | 0.591262 |
| Mother's age at death | rs13037651 | 20:43782230_C_G | 0.818449 | 0.030666 | 0.006621 | 3.60E-06 | 0.99369 | 0.92203 |
| Mother's age at death | rs35227660 | 20:43756697_C_G | 0.817567 | 0.030449 | 0.006591 | 3.80E-06 | 0.99758 | 0.598026 |
| Mother's age at death | rs34163611 | 20:43778990_T_C | 0.818701 | 0.030656 | 0.006632 | 3.80E-06 | 0.99141 | 0.937512 |
| Mother's age at death | rs34916007 | 20:43782952_T_G | 0.818471 | 0.030626 | 0.006622 | 3.80E-06 | 0.9935 | 0.922028 |
| Mother's age at death | rs35009666 | 20:43786020_C_T | 0.818648 | 0.03056 | 0.006611 | 3.80E-06 | 0.99732 | 0.898678 |
| Mother's age at death | rs2013140 | 6:14337780_T_C | 0.385874 | -0.02448 | 0.005295 | 3.80E-06 | 0.97581 | 0.932142 |
| Mother's age at death | rs139851107 | 9:78469955_CCTAGACT_C | 0.979742 | -0.0833 | 0.018026 | 3.80E-06 | 0.99118 | 0.012451 |
| Mother's age at death | rs2604554 | 4:15168679_G_A | 0.495433 | 0.023793 | 0.00515 | 3.80E-06 | 0.97798 | 0.826531 |
| Mother's age at death | rs141063535 | 3:15781355_C_T | 0.97542 | 0.077058 | 0.016679 | 3.80E-06 | 0.96744 | 0.120559 |
| Mother's age at death | rs3754747 | 2:173965296_G_A | 0.835392 | -0.03178 | 0.006874 | 3.80E-06 | 0.99247 | 0.112057 |
| Mother's age at death | . | 2:207702027_ACACT_A | 0.921758 | 0.045238 | 0.009793 | 3.90E-06 | 0.93698 | 0.872694 |
| Mother's age at death | rs6017495 | 20:43756955_G_A | 0.817941 | 0.03048 | 0.006603 | 3.90E-06 | 0.99532 | 0.604241 |
| Mother's age at death | rs67568324 | 20:43779134_GATA_G | 0.817673 | 0.030568 | 0.006619 | 3.90E-06 | 0.99134 | 0.937812 |
| Mother's age at death | rs80323300 | 20:43785287_T_C | 0.818545 | 0.030471 | 0.006604 | 3.90E-06 | 0.99955 | 0.929775 |
| Mother's age at death | rs62208382 | 20:43785549_C_T | 0.818545 | 0.030471 | 0.006604 | 3.90E-06 | 0.99955 | 0.929775 |
| Mother's age at death | rs34085254 | 20:43786170_T_G | 0.818497 | 0.030477 | 0.006604 | 3.90E-06 | 0.99917 | 0.914237 |
| Mother's age at death | rs62208389 | 20:43794783_A_T | 0.818778 | 0.030544 | 0.006616 | 3.90E-06 | 0.99704 | 0.945296 |
| Mother's age at death | rs62206437 | 20:43759316_T_C | 0.81854 | 0.030491 | 0.006613 | 4.00E-06 | 0.99532 | 0.673333 |
| Mother's age at death | rs13039211 | 20:43784396_C_T | 0.81852 | 0.030458 | 0.006604 | 4.00E-06 | 0.9994 | 0.929784 |
| Mother's age at death | rs74174935 | 20:43784872_G_A | 0.818542 | 0.03047 | 0.006604 | 4.00E-06 | 0.99953 | 0.929775 |
| Mother's age at death | rs17018945 | 3:81502898_A_G | 0.909529 | 0.041158 | 0.008921 | 4.00E-06 | 0.98795 | 0.483103 |
| Mother's age at death | rs8110285 | 19:48116478_A_G | 0.24134 | 0.027582 | 0.005991 | 4.10E-06 | 0.98462 | 0.390859 |
| Mother's age at death | rs34233258 | 20:43788265_G_A | 0.818699 | 0.030429 | 0.006613 | 4.20E-06 | 0.99759 | 0.945314 |
| Mother's age at death | rs2868234 | 20:43794026_C_T | 0.818475 | 0.030392 | 0.006608 | 4.20E-06 | 0.99841 | 0.953163 |
| Mother's age at death | rs2868237 | 20:43794189_C_T | 0.818475 | 0.030392 | 0.006608 | 4.20E-06 | 0.99841 | 0.953163 |
| Mother's age at death | rs35361894 | 20:43794403_G_A | 0.818582 | 0.030428 | 0.006611 | 4.20E-06 | 0.99789 | 1 |
| Mother's age at death | rs62208390 | 20:43794844_A_C | 0.818475 | 0.030392 | 0.006608 | 4.20E-06 | 0.99841 | 0.953163 |
| Mother's age at death | rs362296 | 4:3247007_C_A | 0.624056 | -0.02414 | 0.005245 | 4.20E-06 | 1 | 0.707783 |
| Mother's age at death | rs34614969 | 17:16400754_CT_C | 0.428514 | -0.02395 | 0.005214 | 4.30E-06 | 0.96997 | 0.521039 |
| Mother's age at death | rs6032006 | 20:43755053_G_A | 0.817331 | 0.030247 | 0.006587 | 4.40E-06 | 0.99792 | 0.674916 |
| Mother's age at death | rs34073275 | 20:43788917_C_T | 0.818588 | 0.03036 | 0.006611 | 4.40E-06 | 0.99809 | 0.953141 |
| Mother's age at death | rs62208385 | 20:43789823_A_T | 0.818471 | 0.030335 | 0.006608 | 4.40E-06 | 0.99841 | 0.953163 |
| Mother's age at death | rs2223901 | 20:43789924_C_G | 0.818501 | 0.030342 | 0.006609 | 4.40E-06 | 0.99811 | 0.96876 |
| Mother's age at death | rs34976710 | 20:43796772_C_A | 0.818471 | 0.030336 | 0.006608 | 4.40E-06 | 0.99841 | 0.953163 |
| Mother's age at death | rs13038813 | 20:43797025_A_G | 0.818439 | 0.030323 | 0.006607 | 4.40E-06 | 0.99846 | 0.960968 |
| Mother's age at death | rs73907811 | 20:43801395_T_A | 0.818438 | 0.030324 | 0.006607 | 4.40E-06 | 0.99846 | 0.960968 |
| Mother's age at death | rs62208386 | 20:43790872_G_A | 0.818222 | 0.030301 | 0.006607 | 4.50E-06 | 0.99729 | 0.945425 |
| Mother's age at death | rs59858599 | 4:6215568_C_T | 0.926202 | 0.044533 | 0.009711 | 4.50E-06 | 0.99811 | 0.366303 |
| Mother's age at death | rs79111099 | 4:6217368_C_A | 0.926113 | 0.044538 | 0.009709 | 4.50E-06 | 0.99718 | 0.355803 |
| Mother's age at death | rs113568918 | 18:67546690_G_A | 0.955416 | -0.05672 | 0.012362 | 4.50E-06 | 0.98455 | 0.018403 |
| Mother's age at death | rs77106567 | 4:6215012_A_G | 0.926154 | 0.044482 | 0.009706 | 4.60E-06 | 0.99826 | 0.366505 |
| Mother's age at death | rs144201052 | 4:15177337_A_T | 0.584968 | 0.024345 | 0.005312 | 4.60E-06 | 0.94305 | 0.79378 |
| Mother's age at death | rs2285087 | 4:3088211_A_G | 0.641084 | -0.02447 | 0.005341 | 4.60E-06 | 0.98985 | 0.086836 |
| Mother's age at death | rs79939629 | 7:117669497_T_C | 0.94319 | -0.05036 | 0.010986 | 4.60E-06 | 0.99888 | 0.377877 |
| Mother's age at death | rs75975386 | 7:117660634_G_A | 0.943196 | -0.05032 | 0.010987 | 4.70E-06 | 0.99877 | 0.407277 |
| Mother's age at death | rs73191188 | 4:3105200_G_A | 0.644424 | -0.02448 | 0.005346 | 4.70E-06 | 0.99201 | 0.116829 |
| Mother's age at death | rs148369819 | 2:142873026_CTATA_C | 0.492091 | -0.02334 | 0.0051 | 4.70E-06 | 0.99448 | 1 |
| Mother's age at death | rs35770435 | 20:43788804_C_T | 0.816899 | 0.030211 | 0.006602 | 4.70E-06 | 0.99397 | 0.953468 |
| Mother's age at death | rs183453256 | 8:4086021_C_G | 0.989563 | -0.12464 | 0.027238 | 4.70E-06 | 0.84556 | 0.483905 |
| Mother's age at death | rs117356829 | 9:78488796_A_G | 0.979766 | -0.08268 | 0.018055 | 4.70E-06 | 0.99229 | 0.090469 |
| Mother's age at death | rs143084282 | 9:78491344_A_C | 0.979763 | -0.08259 | 0.018051 | 4.70E-06 | 0.99261 | 0.090573 |
| Mother's age at death | rs150999769 | 9:78493282_A_G | 0.979761 | -0.08252 | 0.018048 | 4.80E-06 | 0.99292 | 0.122042 |
| Mother's age at death | rs34855539 | 20:43799036_T_G | 0.818419 | 0.030232 | 0.006608 | 4.80E-06 | 0.99808 | 0.96097 |
| Mother's age at death | rs13044826 | 20:43801865_G_A | 0.818406 | 0.030221 | 0.006607 | 4.80E-06 | 0.99832 | 0.937597 |
| Mother's age at death | rs62208416 | 20:43803226_G_A | 0.818399 | 0.03021 | 0.006607 | 4.80E-06 | 0.99813 | 0.937601 |
| Mother's age at death | rs3132541 | 6:31098734_A_C | 0.845434 | 0.032324 | 0.007065 | 4.80E-06 | 0.99395 | 0.938333 |
| Mother's age at death | rs111455290 | 7:117670313_T_C | 0.943017 | -0.05012 | 0.010965 | 4.90E-06 | 1 | 0.393586 |
| Mother's age at death | rs149291579 | 1:79240882_C_A | 0.989611 | 0.121682 | 0.026621 | 4.90E-06 | 0.88137 | 0.771312 |
| Mother's age at death | rs13042694 | 20:43799368_G_A | 0.817732 | 0.030196 | 0.006614 | 5.00E-06 | 0.99338 | 1 |
| Mother's age at death | rs56387543 | 20:43802600_GT_G | 0.818312 | 0.030118 | 0.006605 | 5.10E-06 | 0.99842 | 0.953196 |
| Mother's age at death | rs17333180 | 20:43803708_C_A | 0.818819 | 0.030208 | 0.006625 | 5.10E-06 | 0.99454 | 0.859914 |
| Mother's age at death | rs373066887 | 2:173904029_CA_C | 0.806169 | -0.03025 | 0.006635 | 5.10E-06 | 0.9358 | 0.108056 |
| Mother's age at death | rs12463612 | 2:173922213_C_T | 0.850891 | -0.03266 | 0.007162 | 5.10E-06 | 0.99283 | 0.167201 |
| Mother's age at death | rs964714 | 7:82382624_G_A | 0.965767 | 0.064337 | 0.014124 | 5.20E-06 | 0.98767 | 0.012465 |
| Mother's age at death | rs35565301 | 3:81501178_G_A | 0.942257 | 0.049957 | 0.010967 | 5.20E-06 | 0.98658 | 0.575692 |
| Mother's age at death | rs75609941 | 9:78496364_T_G | 0.979545 | -0.08208 | 0.018006 | 5.20E-06 | 0.98762 | 0.126061 |
| Mother's age at death | rs12684060 | 9:78500593_C_G | 0.979921 | -0.08273 | 0.01817 | 5.30E-06 | 0.98823 | 0.138299 |
| Mother's age at death | rs17333103 | 20:43803613_C_T | 0.81793 | 0.030063 | 0.006601 | 5.30E-06 | 0.99792 | 0.922189 |
| Mother's age at death | rs6921917 | 6:2860290_C_T | 0.928973 | -0.04582 | 0.010068 | 5.30E-06 | 0.96838 | 0.728182 |
| Mother's age at death | rs73057435 | 2:207713959_G_A | 0.913266 | 0.041247 | 0.009059 | 5.30E-06 | 0.99851 | 0.970912 |
| Mother's age at death | rs12463503 | 2:207715699_G_C | 0.913296 | 0.04124 | 0.009059 | 5.30E-06 | 0.99882 | 0.956372 |
| Mother's age at death | rs73055370 | 2:207705091_T_C | 0.913119 | 0.041217 | 0.009058 | 5.40E-06 | 0.99695 | 0.913015 |
| Mother's age at death | rs73055373 | 2:207705956_G_A | 0.913203 | 0.041194 | 0.00906 | 5.40E-06 | 0.99761 | 0.970928 |
| Mother's age at death | rs148494279 | 2:173964000_TCTTC_T | 0.821438 | -0.03026 | 0.006653 | 5.40E-06 | 0.99155 | 0.074439 |
| Mother's age at death | rs3130557 | 6:31094703_C_T | 0.843859 | 0.031962 | 0.007028 | 5.40E-06 | 1 | 0.112083 |
| Mother's age at death | rs2868235 | 20:43794069_T_C | 0.823726 | 0.030696 | 0.006746 | 5.40E-06 | 0.97974 | 0.904397 |
| Mother's age at death | rs2868236 | 20:43794070_G_A | 0.823726 | 0.030696 | 0.006746 | 5.40E-06 | 0.97974 | 0.904397 |
| Mother's age at death | rs56063128 | 20:43783210_G_A | 0.818428 | 0.03 | 0.006601 | 5.50E-06 | 1 | 1 |
| Mother's age at death | rs6921916 | 6:2860289_C_T | 0.928793 | -0.04566 | 0.010049 | 5.50E-06 | 0.96962 | 0.794831 |
| Mother's age at death | rs350245 | 16:12200115_C_T | 0.42109 | 0.023651 | 0.005205 | 5.50E-06 | 0.98342 | 0.156282 |
| Mother's age at death | rs12478987 | 2:174092229_C_T | 0.795083 | -0.02898 | 0.006375 | 5.50E-06 | 0.97329 | 0.19533 |
| Mother's age at death | rs12473372 | 2:207699785_C_G | 0.913159 | 0.041177 | 0.009059 | 5.50E-06 | 0.99741 | 0.970944 |
| Mother's age at death | rs73055368 | 2:207704283_G_A | 0.913169 | 0.041173 | 0.00906 | 5.50E-06 | 0.99729 | 0.970937 |
| Mother's age at death | rs10164949 | 2:207681005_G_T | 0.911167 | 0.040659 | 0.008953 | 5.60E-06 | 1 | 0.985762 |
| Mother's age at death | rs979641 | 20:43786336_C_T | 0.820044 | 0.030121 | 0.006635 | 5.60E-06 | 0.99607 | 0.782582 |
| Mother's age at death | rs3130985 | 6:31085356_C_T | 0.844041 | 0.031813 | 0.007012 | 5.70E-06 | 1 | 0.73377 |
| Mother's age at death | rs1634726 | 6:30985828_G_A | 0.846841 | 0.032105 | 0.007078 | 5.70E-06 | 0.99702 | 0.656055 |
| Mother's age at death | rs12684062 | 9:78500628_C_A | 0.979811 | -0.08218 | 0.018109 | 5.70E-06 | 0.98944 | 0.141277 |
| Mother's age at death | rs16824789 | 2:207705364_C_A | 0.913138 | 0.041066 | 0.009059 | 5.80E-06 | 0.99739 | 0.98547 |
| Mother's age at death | rs62465927 | 7:82379653_C_T | 0.966557 | 0.064577 | 0.014246 | 5.80E-06 | 0.9927 | 0.019602 |
| Mother's age at death | rs7659144 | 4:3098321_C_G | 0.652521 | -0.02437 | 0.005378 | 5.90E-06 | 0.99042 | 0.143061 |
| Mother's age at death | rs3121418 | 4:3251575_G_A | 0.63311 | -0.02408 | 0.005318 | 5.90E-06 | 0.98559 | 0.243372 |
| Mother's age at death | rs11892772 | 2:207714744_A_G | 0.913154 | 0.041026 | 0.009054 | 5.90E-06 | 0.99828 | 0.941913 |
| Mother's age at death | rs1634716 | 6:30969754_G_A | 0.846951 | 0.03208 | 0.007081 | 5.90E-06 | 0.99797 | 0.973341 |
| Mother's age at death | rs1634721 | 6:30977680_G_A | 0.846959 | 0.03208 | 0.007081 | 5.90E-06 | 0.99791 | 0.973339 |
| Mother's age at death | rs35476703 | 20:43802875_C_G | 0.819115 | 0.029982 | 0.006626 | 6.00E-06 | 0.99594 | 0.937404 |
| Mother's age at death | rs371025208 | 20:43928602_TGGGTGTGTGTGTGTAGGG_T | 0.771691 | 0.027963 | 0.006176 | 6.00E-06 | 0.96813 | 0.28547 |
| Mother's age at death | rs75809482 | 7:117644147_T_C | 0.943214 | -0.04984 | 0.011014 | 6.00E-06 | 0.99436 | 0.391913 |
| Mother's age at death | rs73231764 | 4:40470036_C_T | 0.837553 | -0.03228 | 0.007132 | 6.00E-06 | 0.93166 | 0.504789 |
| Mother's age at death | rs897348 | 4:15186038_A_T | 0.537479 | 0.023373 | 0.005169 | 6.10E-06 | 0.97493 | 0.697509 |
| Mother's age at death | . | 2:207702004_T_TAC | 0.912786 | 0.040969 | 0.009058 | 6.10E-06 | 0.75115 | 0.427592 |
| Mother's age at death | . | 2:207702004_T_TACACAC | 0.912786 | 0.040969 | 0.009058 | 6.10E-06 | 0.97373 | 0.249373 |
| Mother's age at death | . | 2:207702004_TACAC_T | 0.912786 | 0.040969 | 0.009058 | 6.10E-06 | 0.99388 | 0.985523 |
| Mother's age at death | rs570335692 | 4:3138324_G_GA | 0.647586 | -0.02428 | 0.005367 | 6.10E-06 | 0.98462 | 0.241958 |
| Mother's age at death | rs7875403 | 9:78490835_A_G | 0.979656 | -0.08154 | 0.018027 | 6.10E-06 | 0.99057 | 0.107342 |
| Mother's age at death | rs35281662 | 17:16396138_C_CTG | 0.445738 | -0.02343 | 0.005179 | 6.10E-06 | 0.97411 | 0.838158 |
| Mother's age at death | rs35632684 | 20:43802889_C_T | 0.819133 | 0.029977 | 0.006626 | 6.10E-06 | 0.99607 | 0.9374 |
| Mother's age at death | rs60461555 | 8:60599331_T_G | 0.701059 | -0.02598 | 0.005748 | 6.20E-06 | 0.93397 | 0.989011 |
| Mother's age at death | rs3754749 | 2:173906149_A_G | 0.826512 | -0.0307 | 0.006793 | 6.20E-06 | 0.97803 | 0.258012 |
| Mother's age at death | rs199662777 | 18:14442707_T_A | 0.837122 | -0.0353 | 0.00781 | 6.20E-06 | 0.77871 | 0.589156 |
| Mother's age at death | rs56191952 | 20:43802487_A_G | 0.819442 | 0.029964 | 0.006635 | 6.30E-06 | 0.99453 | 0.992157 |
| Mother's age at death | rs1632863 | 6:30968769_C_G | 0.846985 | 0.031992 | 0.007084 | 6.30E-06 | 0.99753 | 0.991111 |
| Mother's age at death | rs12466107 | 2:207678112_C_T | 0.913154 | 0.041052 | 0.009087 | 6.30E-06 | 0.99044 | 0.742711 |
| Mother's age at death | rs28865982 | 2:207672957_G_A | 0.916337 | 0.041781 | 0.009256 | 6.40E-06 | 0.98813 | 0.969882 |
| Mother's age at death | rs111850617 | 7:117636637_C_T | 0.943099 | -0.04995 | 0.011069 | 6.40E-06 | 0.98278 | 0.378051 |
| Mother's age at death | rs3131934 | 6:30931844_T_C | 0.827128 | 0.030416 | 0.006741 | 6.40E-06 | 1 | 0.871588 |
| Mother's age at death | rs4033080 | 5:137482988_A_AAT | 0.668053 | 0.024765 | 0.005486 | 6.40E-06 | 0.97147 | 0.498138 |
| Mother's age at death | rs56151620 | 6:2858215_C_G | 0.92088 | -0.04276 | 0.009484 | 6.50E-06 | 0.98733 | 0.650429 |
| Mother's age at death | rs55767422 | 20:43802497_A_G | 0.819386 | 0.029925 | 0.006635 | 6.50E-06 | 0.99418 | 0.968646 |
| Mother's age at death | rs80126911 | 1:61455134_A_G | 0.953186 | -0.05584 | 0.012384 | 6.50E-06 | 0.94535 | 0.817817 |
| Mother's age at death | rs555918833 | 19:48112734_A_AC | 0.355341 | 0.025111 | 0.005574 | 6.60E-06 | 0.90769 | 0.12184 |
| Mother's age at death | rs78972723 | 7:117638879_C_T | 0.943342 | -0.04986 | 0.011072 | 6.70E-06 | 0.98614 | 0.39094 |
| Mother's age at death | rs1547410 | 4:3254281_C_G | 0.631394 | -0.02398 | 0.005324 | 6.70E-06 | 0.98141 | 0.185016 |
| Mother's age at death | rs148909372 | 3:15576104_G_A | 0.97593 | 0.076213 | 0.01692 | 6.70E-06 | 0.96034 | 0.302448 |
| Mother's age at death | rs73030679 | 3:15937127_T_C | 0.974877 | 0.075113 | 0.01668 | 6.70E-06 | 0.94422 | 0.413114 |
| Mother's age at death | rs3094222 | 6:31081434_A_G | 0.844473 | 0.031665 | 0.007031 | 6.70E-06 | 0.99783 | 0.90372 |
| Mother's age at death | rs4566421 | 20:43783761_G_T | 0.825382 | 0.030211 | 0.006713 | 6.80E-06 | 0.99734 | 0.824229 |
| Mother's age at death | rs36019441 | 20:43892401_G_A | 0.7707 | 0.027382 | 0.006086 | 6.80E-06 | 0.99346 | 0.371415 |
| Mother's age at death | rs114326774 | 2:207725532_A_G | 0.948977 | 0.052509 | 0.011672 | 6.80E-06 | 0.97764 | 0.188695 |
| Mother's age at death | rs570662154 | 2:238817052_C_CTGGTGGTGGTAGTGGTGG | 0.824807 | -0.03359 | 0.007471 | 6.90E-06 | 0.80422 | 0.734412 |
| Mother's age at death | rs4690071 | 4:3086753_A_T | 0.640657 | -0.02405 | 0.005353 | 7.00E-06 | 0.98474 | 0.151165 |
| Mother's age at death | rs3121417 | 4:3252678_G_A | 0.632009 | -0.02384 | 0.005303 | 7.00E-06 | 0.98914 | 0.300621 |
| Mother's age at death | rs6032086 | 20:43899903_T_C | 0.771077 | 0.027362 | 0.00609 | 7.00E-06 | 0.99372 | 0.336853 |
| Mother's age at death | rs991048 | 20:43898227_T_C | 0.770994 | 0.027349 | 0.00609 | 7.10E-06 | 0.99332 | 0.332859 |
| Mother's age at death | rs8092234 | 18:9371333_C_T | 0.898751 | 0.037948 | 0.008449 | 7.10E-06 | 0.99486 | 0.657658 |
| Mother's age at death | rs116924021 | 13:52658460_C_T | 0.971935 | -0.07186 | 0.015998 | 7.10E-06 | 0.92732 | 0.83329 |
| Mother's age at death | rs74417392 | 2:174086285_C_T | 0.792262 | -0.0284 | 0.006322 | 7.10E-06 | 0.979 | 0.165116 |
| Mother's age at death | rs6017532 | 20:43904095_G_A | 0.771101 | 0.027318 | 0.006089 | 7.20E-06 | 0.99397 | 0.336822 |
| Mother's age at death | rs2741500 | 20:43944958_G_T | 0.780353 | 0.027736 | 0.006181 | 7.20E-06 | 0.99221 | 0.361468 |
| Mother's age at death | rs13042474 | 20:43893068_G_T | 0.770832 | 0.027302 | 0.006088 | 7.30E-06 | 0.99304 | 0.371202 |
| Mother's age at death | rs3131788 | 6:31024796_G_A | 0.844866 | 0.03153 | 0.007032 | 7.30E-06 | 1 | 0.912246 |
| Mother's age at death | rs117902543 | 9:78490915_G_A | 0.979638 | -0.08082 | 0.018017 | 7.30E-06 | 0.99077 | 0.10756 |
| Mother's age at death | . | 9:78492438_A_AAT | 0.970658 | -0.07064 | 0.015753 | 7.30E-06 | 0.90294 | 0.126976 |
| Mother's age at death | rs112059289 | 22:36298862_A_T | 0.978868 | -0.10177 | 0.022691 | 7.30E-06 | 0.60946 | 0.485043 |
| Mother's age at death | rs9941695 | 2:142818354_T_C | 0.334205 | -0.02415 | 0.005391 | 7.40E-06 | 0.99653 | 0.790533 |
| Mother's age at death | rs12471204 | 2:207718894_T_G | 0.91447 | 0.041015 | 0.00915 | 7.40E-06 | 0.99092 | 1 |
| Mother's age at death | rs10919022 | 1:168908721_G_A | 0.634267 | 0.023967 | 0.005349 | 7.40E-06 | 0.97628 | 0.290362 |
| Mother's age at death | rs112300862 | 7:117636559_A_G | 0.943354 | -0.04964 | 0.011073 | 7.40E-06 | 0.98624 | 0.421076 |
| Mother's age at death | rs115339085 | 7:117642316_C_T | 0.943354 | -0.04964 | 0.011073 | 7.40E-06 | 0.98624 | 0.421076 |
| Mother's age at death | rs76322319 | 7:117638276_G_A | 0.943327 | -0.0496 | 0.011073 | 7.50E-06 | 0.98586 | 0.436958 |
| Mother's age at death | rs73070067 | 2:207646236_C_T | 0.915496 | 0.041077 | 0.00917 | 7.50E-06 | 0.99878 | 0.970186 |
| Mother's age at death | rs350251 | 16:12202572_A_G | 0.420676 | 0.02332 | 0.005205 | 7.50E-06 | 0.9838 | 0.133444 |
| Mother's age at death | rs193252 | 16:12203844_T_C | 0.421058 | 0.023289 | 0.005204 | 7.60E-06 | 0.98403 | 0.130491 |
| Mother's age at death | rs13439915 | 9:114918170_A_G | 0.731414 | 0.028313 | 0.006326 | 7.60E-06 | 0.82365 | 0.988249 |
| Mother's age at death | rs11687539 | 2:174023857_A_G | 0.794377 | -0.02817 | 0.006294 | 7.60E-06 | 0.9966 | 0.352241 |
| Mother's age at death | rs67950027 | 4:3152786_A_C | 0.672622 | -0.02459 | 0.005498 | 7.70E-06 | 0.97591 | 0.02293 |
| Mother's age at death | rs10928134 | 2:142813955_C_G | 0.333999 | -0.02412 | 0.005396 | 7.80E-06 | 0.99479 | 0.800461 |
| Mother's age at death | rs2197910 | 2:142820369_T_C | 0.334453 | -0.0241 | 0.005391 | 7.80E-06 | 0.99606 | 0.775675 |
| Mother's age at death | rs1375603 | 2:142820421_A_G | 0.334453 | -0.0241 | 0.005391 | 7.80E-06 | 0.99606 | 0.775675 |
| Mother's age at death | rs35967905 | 20:43944041_TA_T | 0.779242 | 0.027694 | 0.006197 | 7.80E-06 | 0.9843 | 0.312385 |
| Mother's age at death | rs35710116 | 2:142813871_G_GA | 0.333794 | -0.02412 | 0.005399 | 7.90E-06 | 0.99428 | 0.825557 |
| Mother's age at death | rs3762569 | 2:207635594_G_T | 0.915401 | 0.040961 | 0.009166 | 7.90E-06 | 0.99828 | 0.985103 |
| Mother's age at death | rs7605735 | 2:207685614_A_G | 0.912924 | 0.040509 | 0.009065 | 7.90E-06 | 0.99391 | 0.827285 |
| Mother's age at death | rs7593417 | 2:207685615_T_C | 0.912924 | 0.040509 | 0.009065 | 7.90E-06 | 0.99391 | 0.827285 |
| Mother's age at death | rs5834895 | 2:142817110_G_GT | 0.334437 | -0.02407 | 0.005392 | 8.00E-06 | 0.99588 | 0.785615 |
| Mother's age at death | rs350248 | 16:12202424_G_C | 0.420863 | 0.023244 | 0.005204 | 8.00E-06 | 0.98424 | 0.130452 |
| Mother's age at death | rs3130562 | 6:31100974_T_C | 0.843724 | 0.031315 | 0.007011 | 8.00E-06 | 0.99947 | 0.930176 |
| Mother's age at death | rs55804402 | 6:2858426_C_T | 0.92072 | -0.04231 | 0.009477 | 8.00E-06 | 0.98669 | 0.723269 |
| Mother's age at death | rs1793894 | 6:31196862_G_A | 0.830223 | 0.030316 | 0.006794 | 8.10E-06 | 0.99098 | 0.076542 |
| Mother's age at death | rs4522674 | 20:43893276_T_C | 0.770461 | 0.027153 | 0.006086 | 8.10E-06 | 0.99284 | 0.367358 |
| Mother's age at death | rs12618881 | 2:142814226_T_A | 0.334676 | -0.02408 | 0.005397 | 8.10E-06 | 0.99394 | 0.820741 |
| Mother's age at death | rs34199343 | 2:142863149_CAT_C | 0.485281 | -0.02276 | 0.005101 | 8.10E-06 | 0.99403 | 0.986185 |
| Mother's age at death | rs536968098 | 2:207662722_A_AT | 0.915648 | 0.041037 | 0.009199 | 8.10E-06 | 0.99437 | 0.910626 |
| Mother's age at death | rs201502153 | 2:207675343_AGG_A | 0.912934 | 0.040447 | 0.009063 | 8.10E-06 | 0.99356 | 0.785037 |
| Mother's age at death | rs2798287 | 4:2938651_C_A | 0.501215 | 0.022926 | 0.005139 | 8.10E-06 | 0.98178 | 0.429507 |
| Mother's age at death | rs11080763 | 18:14462687_G_A | 0.834637 | -0.03122 | 0.006994 | 8.10E-06 | 0.96272 | 0.420707 |
| Mother's age at death | rs3129322 | 4:3252852_T_C | 0.626942 | -0.02369 | 0.005311 | 8.20E-06 | 0.98064 | 0.355165 |
| Mother's age at death | . | 3:185383797_G_C | 0.940498 | -0.06102 | 0.013684 | 8.20E-06 | 0.6154 | 0.73514 |
| Mother's age at death | rs60556681 | 2:207663289_CT_C | 0.915731 | 0.041021 | 0.009199 | 8.20E-06 | 0.99518 | 0.910538 |
| Mother's age at death | rs73055333 | 2:207663982_A_G | 0.91573 | 0.041034 | 0.009199 | 8.20E-06 | 0.99516 | 0.910538 |
| Mother's age at death | rs12105584 | 2:142816862_T_C | 0.334055 | -0.02407 | 0.005395 | 8.20E-06 | 0.99545 | 0.825633 |
| Mother's age at death | rs993935 | 2:142817168_T_C | 0.334485 | -0.02404 | 0.005393 | 8.30E-06 | 0.99542 | 0.790624 |
| Mother's age at death | rs10497402 | 2:174088266_G_A | 0.792954 | -0.02825 | 0.006339 | 8.30E-06 | 0.97618 | 0.172286 |
| Mother's age at death | rs3095311 | 6:31051675_A_G | 0.844799 | 0.031343 | 0.00703 | 8.30E-06 | 0.99997 | 0.894827 |
| Mother's age at death | rs3130544 | 6:31058340_C_A | 0.845698 | 0.031385 | 0.007047 | 8.40E-06 | 1 | 0.903107 |
| Mother's age at death | rs935006 | 2:174037491_G_A | 0.221118 | 0.027313 | 0.006132 | 8.40E-06 | 0.99514 | 0.512578 |
| Mother's age at death | rs4662370 | 2:142812609_T_G | 0.334495 | -0.02402 | 0.005392 | 8.40E-06 | 0.99521 | 0.75591 |
| Mother's age at death | rs115999916 | 18:9372399_G_A | 0.904198 | 0.03915 | 0.008789 | 8.40E-06 | 0.96568 | 0.594758 |
| Mother's age at death | rs71334215 | 19:48097616_C_CTTAAT | 0.279379 | 0.02585 | 0.005804 | 8.40E-06 | 0.95432 | 0.220978 |
| Mother's age at death | rs183008 | 16:12201691_T_C | 0.420904 | 0.023182 | 0.005204 | 8.40E-06 | 0.98418 | 0.138147 |
| Mother's age at death | rs4394477 | 9:83162551_G_T | 0.205086 | -0.02804 | 0.006295 | 8.40E-06 | 1 | 0.363287 |
| Mother's age at death | rs17280449 | 2:207686461_G_T | 0.912184 | 0.040092 | 0.009006 | 8.50E-06 | 0.99873 | 0.800498 |
| Mother's age at death | rs6746658 | 2:174087310_T_A | 0.794536 | -0.02839 | 0.006378 | 8.50E-06 | 0.97046 | 0.18398 |
| Mother's age at death | rs6435351 | 2:207659014_T_C | 0.915186 | 0.040815 | 0.009174 | 8.60E-06 | 0.99464 | 0.911052 |
| Mother's age at death | rs12470278 | 2:207686910_C_T | 0.912182 | 0.040081 | 0.009005 | 8.60E-06 | 0.99873 | 0.814449 |
| Mother's age at death | rs4731367 | 7:127178162_C_T | 0.378704 | 0.023252 | 0.005226 | 8.60E-06 | 0.99608 | 0.002641 |
| Mother's age at death | rs3131920 | 6:31018908_T_G | 0.845692 | 0.031356 | 0.007046 | 8.60E-06 | 0.99984 | 0.816265 |
| Mother's age at death | rs55876241 | 6:2849520_C_T | 0.911593 | -0.03989 | 0.008975 | 8.80E-06 | 0.99601 | 0.762097 |
| Mother's age at death | rs28435282 | 19:48119969_C_T | 0.515057 | 0.022944 | 0.005163 | 8.80E-06 | 0.98056 | 0.088654 |
| Mother's age at death | rs28409356 | 19:48119986_C_T | 0.515171 | 0.022938 | 0.005162 | 8.90E-06 | 0.98078 | 0.091939 |
| Mother's age at death | rs350252 | 16:12202934_C_G | 0.42111 | 0.023117 | 0.005205 | 8.90E-06 | 0.98377 | 0.126058 |
| Mother's age at death | . | 2:207658000_A_AC | 0.915426 | 0.040784 | 0.009181 | 8.90E-06 | 0.99567 | 0.940479 |
| Mother's age at death | rs2193885 | 2:207658595_T_A | 0.915426 | 0.040784 | 0.009181 | 8.90E-06 | 0.99567 | 0.940479 |
| Mother's age at death | rs73055376 | 2:207710934_T_G | 0.912023 | 0.040081 | 0.009021 | 8.90E-06 | 0.99506 | 1 |
| Mother's age at death | rs1900934 | 2:142812740_G_T | 0.334451 | -0.02396 | 0.005392 | 8.90E-06 | 0.99526 | 0.746071 |
| Mother's age at death | . | 2:207682196_G_C | 0.912487 | 0.040147 | 0.00904 | 9.00E-06 | 0.99415 | 0.772076 |
| Mother's age at death | rs11690597 | 2:174085741_A_G | 0.79211 | -0.02806 | 0.00632 | 9.00E-06 | 0.97907 | 0.176267 |
| Mother's age at death | rs539354792 | 6:31072695_T_C | 0.846204 | 0.031472 | 0.007087 | 9.00E-06 | 0.99016 | 0.578882 |
| Mother's age at death | rs145186643 | 9:78498300_G_A | 0.979626 | -0.07999 | 0.018029 | 9.10E-06 | 0.98911 | 0.107669 |
| Mother's age at death | rs74956353 | 2:207664740_A_G | 0.916225 | 0.041017 | 0.009245 | 9.10E-06 | 0.99083 | 0.777553 |
| Mother's age at death | rs57848535 | 2:207683276_A_C | 0.912232 | 0.039981 | 0.00901 | 9.10E-06 | 0.99809 | 0.814372 |
| Mother's age at death | rs12475891 | 2:207637397_C_T | 0.915284 | 0.040618 | 0.00916 | 9.20E-06 | 0.99864 | 0.940542 |
| Mother's age at death | rs73055344 | 2:207678712_A_C | 0.912149 | 0.039992 | 0.009015 | 9.20E-06 | 0.99644 | 0.828552 |
| Mother's age at death | rs115372136 | 2:207731193_G_T | 0.949891 | 0.051885 | 0.0117 | 9.20E-06 | 0.98995 | 0.224388 |
| Mother's age at death | rs144711978 | 2:207675144_T_C | 0.912191 | 0.039958 | 0.009012 | 9.30E-06 | 0.99768 | 0.913747 |
| Mother's age at death | rs12105566 | 2:142816442_T_C | 0.334444 | -0.02392 | 0.005396 | 9.30E-06 | 0.99451 | 0.805612 |
| Mother's age at death | rs144846110 | 6:31066633_A_G | 0.842579 | 0.030987 | 0.006992 | 9.30E-06 | 0.99977 | 0.921932 |
| Mother's age at death | rs13038342 | 20:43800232_G_A | 0.817381 | 0.029238 | 0.006595 | 9.30E-06 | 0.99726 | 0.876139 |
| Mother's age at death | rs66838096 | 20:43944468_AAGAG_A | 0.78104 | 0.027425 | 0.006187 | 9.30E-06 | 0.99283 | 0.378538 |
| Mother's age at death | . | 9:83308288_A_T | 0.21172 | -0.02762 | 0.006232 | 9.30E-06 | 0.70688 | 1 |
| Mother's age at death | . | 9:83308288_ATT_A | 0.21172 | -0.02762 | 0.006232 | 9.30E-06 | 0.99011 | 0.179449 |
| Mother's age at death | rs35806161 | 10:86079188_CA_C | 0.879311 | 0.038168 | 0.008611 | 9.30E-06 | 0.82414 | 0.267744 |
| Mother's age at death | . | 18:9371295_CTT_C | 0.897582 | 0.037366 | 0.008436 | 9.40E-06 | 0.98814 | 0.471173 |
| Mother's age at death | rs2914008 | 19:48097205_T_A | 0.247215 | 0.02688 | 0.006067 | 9.40E-06 | 0.94872 | 0.993813 |
| Mother's age at death | rs28863886 | 2:207672271_G_A | 0.912183 | 0.039929 | 0.009012 | 9.40E-06 | 0.99766 | 0.928088 |
| Mother's age at death | rs73985719 | 2:207638894_A_C | 0.915417 | 0.040584 | 0.009166 | 9.50E-06 | 0.99841 | 0.985102 |
| Mother's age at death | rs75843327 | 2:173939405_T_C | 0.827659 | -0.03001 | 0.006777 | 9.50E-06 | 0.98733 | 0.295053 |
| Mother's age at death | rs3130574 | 6:31016550_A_G | 0.846024 | 0.031269 | 0.007063 | 9.50E-06 | 0.99739 | 0.859265 |
| Mother's age at death | rs187455946 | 5:5639551_G_T | 0.989646 | 0.115478 | 0.026086 | 9.60E-06 | 0.92176 | 0.154899 |
| Mother's age at death | rs57562572 | 2:207677485_A_G | 0.912025 | 0.039845 | 0.009003 | 9.60E-06 | 0.99772 | 0.814744 |
| Mother's age at death | rs12474018 | 2:207677783_A_G | 0.912025 | 0.039845 | 0.009003 | 9.60E-06 | 0.99772 | 0.814744 |
| Mother's age at death | rs12694062 | 2:207678216_A_G | 0.912025 | 0.039845 | 0.009003 | 9.60E-06 | 0.99772 | 0.814744 |
| Mother's age at death | rs6726245 | 2:207679227_G_A | 0.912025 | 0.039845 | 0.009003 | 9.60E-06 | 0.99772 | 0.814744 |
| Mother's age at death | rs6754636 | 2:207679301_A_G | 0.912025 | 0.039845 | 0.009003 | 9.60E-06 | 0.99772 | 0.814744 |
| Mother's age at death | rs6728079 | 2:207681320_T_C | 0.911955 | 0.039831 | 0.009001 | 9.60E-06 | 0.99749 | 0.828928 |
| Mother's age at death | rs10204473 | 2:45492633_T_C | 0.690717 | -0.02458 | 0.005555 | 9.60E-06 | 0.98019 | 0.74571 |
| Mother's age at death | rs6466641 | 7:117646398_A_G | 0.944021 | -0.04912 | 0.011096 | 9.60E-06 | 0.99261 | 0.370288 |
| Mother's age at death | rs2914005 | 19:48096971_T_C | 0.247238 | 0.026835 | 0.006067 | 9.70E-06 | 0.94852 | 0.993813 |
| Mother's age at death | rs10409634 | 19:48118534_C_T | 0.513912 | 0.022813 | 0.00516 | 9.80E-06 | 0.98124 | 0.113841 |
| Mother's age at death | rs6731949 | 2:174087309_G_A | 0.794241 | -0.02819 | 0.006376 | 9.80E-06 | 0.96996 | 0.18156 |
| Mother's age at death | rs3094671 | 6:31044463_T_C | 0.845399 | 0.031182 | 0.007053 | 9.80E-06 | 0.99667 | 0.912009 |
| Mother's age at death | rs139797560 | 6:2853668_T_A | 0.925413 | -0.04302 | 0.009736 | 9.90E-06 | 0.98814 | 0.75504 |
| Mother's age at death | rs113177013 | 8:37692645_G_A | 0.987075 | -0.11066 | 0.025041 | 9.90E-06 | 0.81666 | 0.656478 |
| Mother's age at death | rs75543236 | 2:173939408_T_C | 0.828001 | -0.02999 | 0.006785 | 9.90E-06 | 0.98638 | 0.258327 |
| Mother's age at death | rs16861363 | 2:174086492_C_T | 0.792365 | -0.02792 | 0.006325 | 1.00E-05 | 0.97859 | 0.181593 |
| Mother's age at death | rs72387198 | 2:142860128_AAT_A | 0.510962 | -0.02291 | 0.005187 | 1.00E-05 | 0.96037 | 0.858053 |
| Mother's age at death | rs74559635 | 2:207640301_T_G | 0.915311 | 0.040397 | 0.009161 | 1.00E-05 | 0.99868 | 0.925697 |
| Mother's age at death | rs12478693 | 2:207644737_C_A | 0.91529 | 0.040441 | 0.00916 | 1.00E-05 | 0.99852 | 0.970249 |
| Mother's age at death | rs878832 | 2:207650638_T_C | 0.915331 | 0.040429 | 0.00916 | 1.00E-05 | 0.99902 | 0.955373 |
| Mother's age at death | rs879472 | 2:207651782_G_A | 0.915333 | 0.040414 | 0.009161 | 1.00E-05 | 0.99905 | 0.970235 |
| Mother's age at death | rs6727967 | 2:207681245_T_C | 0.912051 | 0.039747 | 0.009001 | 1.00E-05 | 0.99829 | 0.82876 |
| Mother's age at death | rs10181219 | 2:207682497_T_C | 0.912597 | 0.039891 | 0.009047 | 1.00E-05 | 0.99385 | 0.813699 |
| Mother's age at death | rs77298641 | 2:207694935_G_C | 0.912356 | 0.039759 | 0.009017 | 1.00E-05 | 0.99856 | 0.927964 |
| Mother's age at death | rs3762568 | 2:207631461_G_A | 0.915265 | 0.040392 | 0.009165 | 1.00E-05 | 0.9973 | 0.94057 |
| Mother's age at death | rs113322591 | 7:117635663_G_A | 0.943454 | -0.04902 | 0.0111 | 1.00E-05 | 0.98338 | 0.435863 |
| Mother's age at death | rs145785297 | 7:82379158_CAT_C | 0.965224 | 0.061827 | 0.014002 | 1.00E-05 | 0.99101 | 0.008557 |
| Mother's age at death | rs2914007 | 19:48097125_T_G | 0.247273 | 0.0268 | 0.006066 | 1.00E-05 | 0.9486 | 0.987628 |
| Mother's age at death | rs2921547 | 19:48097257_A_T | 0.247051 | 0.026792 | 0.00607 | 1.00E-05 | 0.94828 | 0.99381 |
| Mother's age at death | rs199644591 | 14:89974133_AAG_A | 0.969477 | 0.077891 | 0.017647 | 1.00E-05 | 0.7018 | 0.409789 |
| Mother's age at death | rs2664186 | 16:12210702_A_C | 0.41922 | 0.022937 | 0.005197 | 1.00E-05 | 0.98763 | 0.164627 |
| Mother's age at death | rs3131794 | 6:31035391_G_T | 0.842508 | 0.030902 | 0.006996 | 1.00E-05 | 0.99699 | 0.947929 |
| Mother's age at death | rs2233980 | 6:31079644_G_A | 0.844791 | 0.031015 | 0.00703 | 1.00E-05 | 0.99978 | 0.85143 |
| Mother's age at death | rs3131781 | 6:30937732_A_G | 0.846924 | 0.0313 | 0.007086 | 1.00E-05 | 0.99749 | 0.858521 |
| Mother's age at death | rs2442722 | 6:31320241_G_A | 0.795549 | 0.02792 | 0.006326 | 1.00E-05 | 0.98608 | 0.00364 |
| Mother's age at death | rs564078490 | 20:43863429_G_GT | 0.800989 | 0.028501 | 0.006456 | 1.00E-05 | 0.97778 | 0.609649 |
| Mother's age at death | rs13039345 | 20:43895932_G_C | 0.772544 | 0.026796 | 0.006108 | 1.10E-05 | 0.99203 | 0.248342 |
| Mother's age at death | rs2741502 | 20:43936265_G_A | 0.780395 | 0.027118 | 0.006172 | 1.10E-05 | 0.99555 | 0.44691 |
| Mother's age at death | rs6096437 | 20:50070623_C_T | 0.867206 | -0.0333 | 0.007589 | 1.10E-05 | 0.97251 | 0.034698 |
| Mother's age at death | rs117413728 | 10:118974724_T_C | 0.988379 | 0.120701 | 0.027402 | 1.10E-05 | 0.74441 | 0.440809 |
| Mother's age at death | rs10484910 | 6:2459289_G_C | 0.760103 | 0.03379 | 0.007683 | 1.10E-05 | 0.602 | 0.837412 |
| Mother's age at death | rs35881974 | 5:137455350_AT_A | 0.578762 | 0.023133 | 0.005269 | 1.10E-05 | 0.95061 | 0.040588 |
| Mother's age at death | rs114812432 | 6:17658032_T_C | 0.978227 | -0.07898 | 0.01799 | 1.10E-05 | 0.94499 | 0.460702 |
| Mother's age at death | rs55709717 | 8:2227564_T_C | 0.411912 | -0.02698 | 0.006143 | 1.10E-05 | 0.71579 | 0.005622 |
| Mother's age at death | rs9356165 | 6:164306190_A_T | 0.512261 | 0.022534 | 0.00512 | 1.10E-05 | 0.98398 | 0.285706 |
| Mother's age at death | rs186221247 | 6:71095933_G_A | 0.989727 | 0.116334 | 0.026511 | 1.10E-05 | 0.89777 | 0.042131 |
| Mother's age at death | rs1891701 | 4:3141130_G_A | 0.68491 | -0.02412 | 0.005494 | 1.10E-05 | 0.99445 | 0.085645 |
| Mother's age at death | rs448449 | 4:40467564_C_T | 0.835088 | -0.03126 | 0.007107 | 1.10E-05 | 0.92804 | 0.586516 |
| Mother's age at death | rs16838839 | 2:207630093_C_A | 0.915267 | 0.040356 | 0.009164 | 1.10E-05 | 0.99749 | 0.925734 |
| Mother's age at death | rs12468869 | 2:207630802_G_A | 0.915251 | 0.040373 | 0.009164 | 1.10E-05 | 0.99749 | 0.94057 |
| Mother's age at death | rs73070048 | 2:207641394_T_A | 0.915672 | 0.040375 | 0.009186 | 1.10E-05 | 0.99704 | 0.895726 |
| Mother's age at death | rs57475447 | 2:207653770_A_G | 0.915238 | 0.040216 | 0.009159 | 1.10E-05 | 0.99849 | 0.94057 |
| Mother's age at death | rs1001805 | 2:207659531_A_G | 0.91511 | 0.040335 | 0.009171 | 1.10E-05 | 0.99435 | 0.911144 |
| Mother's age at death | rs59114932 | 2:207687554_A_G | 0.912173 | 0.039661 | 0.009005 | 1.10E-05 | 0.99888 | 0.814479 |
| Mother's age at death | rs35342955 | 2:207696262_G_A | 0.912335 | 0.039639 | 0.009023 | 1.10E-05 | 0.99713 | 0.927996 |
| Mother's age at death | rs350255 | 16:12205542_A_G | 0.418579 | 0.0229 | 0.005206 | 1.10E-05 | 0.98438 | 0.169895 |
| Mother's age at death | rs1704116 | 16:12209658_A_G | 0.418949 | 0.022849 | 0.005196 | 1.10E-05 | 0.98778 | 0.173728 |
| Mother's age at death | rs1704112 | 16:12211425_C_G | 0.419431 | 0.022819 | 0.005197 | 1.10E-05 | 0.98711 | 0.170134 |
| Mother's age at death | rs350210 | 16:12212689_T_G | 0.418916 | 0.022841 | 0.005195 | 1.10E-05 | 0.98827 | 0.185204 |
| Mother's age at death | rs62571394 | 9:79706937_T_G | 0.919132 | -0.04105 | 0.009322 | 1.10E-05 | 0.99679 | 0.332086 |
| Mother's age at death | rs4801726 | 19:48121609_G_T | 0.514121 | 0.022668 | 0.005157 | 1.10E-05 | 0.9827 | 0.103662 |
| Mother's age at death | rs28538616 | 19:48122540_T_C | 0.51397 | 0.022718 | 0.005157 | 1.10E-05 | 0.98229 | 0.096516 |
| Mother's age at death | rs184479191 | 21:33959158_C_G | 0.985212 | 0.099467 | 0.02262 | 1.10E-05 | 0.85277 | 0.000263 |
| Mother's age at death | rs350254 | 16:12203789_C_T | 0.423017 | 0.022745 | 0.0052 | 1.20E-05 | 0.98371 | 0.165596 |
| Mother's age at death | rs350209 | 16:12212818_G_C | 0.418935 | 0.022717 | 0.005195 | 1.20E-05 | 0.98822 | 0.19317 |
| Mother's age at death | rs783543 | 15:83239486_A_C | 0.387919 | -0.02298 | 0.005257 | 1.20E-05 | 0.984 | 0.597379 |
| Mother's age at death | rs2213431 | 22:37639087_T_C | 0.579539 | 0.022742 | 0.005189 | 1.20E-05 | 0.98075 | 0.029027 |
| Mother's age at death | rs10746658 | 9:83159880_A_T | 0.203538 | -0.02767 | 0.006318 | 1.20E-05 | 0.9978 | 0.256035 |
| Mother's age at death | rs371205594 | 22:25336718_C_T | 0.963879 | 0.069169 | 0.015803 | 1.20E-05 | 0.74621 | 0.773499 |
| Mother's age at death | rs16955004 | 18:9358726_A_T | 0.899132 | 0.0371 | 0.008487 | 1.20E-05 | 0.98955 | 0.726675 |
| Mother's age at death | rs55730273 | 14:76515203_G_A | 0.989067 | 0.121755 | 0.027774 | 1.20E-05 | 0.77423 | 0.146171 |
| Mother's age at death | . | 14:21078275_C_G | 0.984549 | -0.09237 | 0.021088 | 1.20E-05 | 0.9583 | 0.849333 |
| Mother's age at death | rs3799338 | 6:164298692_C_G | 0.803099 | 0.028242 | 0.006448 | 1.20E-05 | 0.98249 | 0.37732 |
| Mother's age at death | rs4755313 | 11:45320727_G_C | 0.879236 | 0.034572 | 0.007881 | 1.20E-05 | 0.98831 | 0.141472 |
| Mother's age at death | rs6032059 | 20:43852443_A_T | 0.81652 | 0.029014 | 0.006618 | 1.20E-05 | 0.98923 | 0.891809 |
| Mother's age at death | rs6032060 | 20:43852454_A_T | 0.81649 | 0.028995 | 0.006617 | 1.20E-05 | 0.98941 | 0.891824 |
| Mother's age at death | rs377130964 | 5:137550604_CA_C | 0.617602 | 0.023481 | 0.005364 | 1.20E-05 | 0.95739 | 0.26356 |
| Mother's age at death | rs563855677 | 2:207679037_T_G | 0.908383 | 0.039082 | 0.008918 | 1.20E-05 | 0.98045 | 0.74114 |
| Mother's age at death | rs61120804 | 2:174014165_G_GT | 0.806474 | -0.02829 | 0.006456 | 1.20E-05 | 0.99166 | 0.187971 |
| Mother's age at death | rs10583454 | 2:142827412_GACT_G | 0.483564 | -0.02232 | 0.005092 | 1.20E-05 | 0.99753 | 0.995394 |
| Mother's age at death | rs1001629 | 2:142868530_T_A | 0.48453 | -0.02228 | 0.005092 | 1.20E-05 | 0.99826 | 0.96777 |
| Mother's age at death | rs66684864 | 4:3182849_T_G | 0.683407 | -0.02398 | 0.005484 | 1.20E-05 | 0.99557 | 0.079396 |
| Mother's age at death | rs73193303 | 4:3192200_A_G | 0.684295 | -0.02405 | 0.005484 | 1.20E-05 | 0.99734 | 0.114884 |
| Mother's age at death | rs11445574 | 4:3212336_T_TG | 0.665433 | -0.0237 | 0.005419 | 1.20E-05 | 0.99326 | 0.048117 |
| Mother's age at death | rs62465922 | 7:82378080_T_A | 0.965281 | 0.061357 | 0.014029 | 1.20E-05 | 0.98901 | 0.009581 |
| Mother's age at death | rs7803240 | 7:82379221_G_A | 0.965111 | 0.061184 | 0.01395 | 1.20E-05 | 0.99401 | 0.018009 |
| Mother's age at death | rs62465920 | 7:82377272_A_C | 0.96524 | 0.061215 | 0.014029 | 1.30E-05 | 0.98767 | 0.008557 |
| Mother's age at death | rs62465923 | 7:82378208_G_A | 0.965154 | 0.061218 | 0.014015 | 1.30E-05 | 0.98698 | 0.012552 |
| Mother's age at death | rs6952997 | 7:82378816_A_G | 0.965055 | 0.060823 | 0.013976 | 1.30E-05 | 0.98928 | 0.016218 |
| Mother's age at death | rs16843836 | 4:3113337_G_A | 0.68553 | -0.02395 | 0.005501 | 1.30E-05 | 0.99285 | 0.096828 |
| Mother's age at death | rs71949557 | 2:142805634_CTA_C | 0.432996 | -0.02242 | 0.005149 | 1.30E-05 | 0.99473 | 0.488315 |
| Mother's age at death | rs1449501 | 2:142806913_A_G | 0.433347 | -0.0224 | 0.005145 | 1.30E-05 | 0.99659 | 0.514577 |
| Mother's age at death | rs10928133 | 2:142809120_C_T | 0.433279 | -0.0224 | 0.005145 | 1.30E-05 | 0.9964 | 0.514565 |
| Mother's age at death | rs68085426 | 2:142850221_T_G | 0.501333 | -0.02248 | 0.005147 | 1.30E-05 | 0.97654 | 0.853593 |
| Mother's age at death | rs62155598 | 2:142870644_C_A | 0.484576 | -0.02217 | 0.005091 | 1.30E-05 | 0.99842 | 0.940186 |
| Mother's age at death | rs56675577 | 2:207646994_A_G | 0.915571 | 0.039981 | 0.009178 | 1.30E-05 | 0.99794 | 0.910674 |
| Mother's age at death | rs3791984 | 2:207654106_A_G | 0.915588 | 0.040031 | 0.009185 | 1.30E-05 | 0.99632 | 1 |
| Mother's age at death | rs17214441 | 2:207683777_A_G | 0.931137 | 0.043912 | 0.010055 | 1.30E-05 | 0.99787 | 0.58799 |
| Mother's age at death | rs35392675 | 21:33779209_CT_C | 0.835409 | 0.033379 | 0.007653 | 1.30E-05 | 0.7953 | 0.07663 |
| Mother's age at death | rs4806890 | 19:2939062_T_C | 0.229441 | -0.02726 | 0.006255 | 1.30E-05 | 0.93076 | 0.49815 |
| Mother's age at death | rs350256 | 16:12207491_G_A | 0.419822 | 0.022647 | 0.005204 | 1.30E-05 | 0.98492 | 0.172092 |
| Mother's age at death | . | 18:9364802_AAAG_A | 0.90977 | 0.039805 | 0.009112 | 1.30E-05 | 0.9499 | 0.972037 |
| Mother's age at death | rs1410534 | 9:83165544_G_A | 0.206095 | -0.02748 | 0.0063 | 1.30E-05 | 0.99438 | 0.346745 |
| Mother's age at death | rs35247152 | 9:132205506_G_C | 0.919834 | -0.04229 | 0.009704 | 1.30E-05 | 0.92789 | 0.667437 |
| Mother's age at death | rs3130566 | 6:31102618_C_G | 0.840772 | 0.030364 | 0.006954 | 1.30E-05 | 0.99821 | 0.627451 |
| Mother's age at death | rs3132566 | 6:31102790_G_T | 0.840748 | 0.030307 | 0.006954 | 1.30E-05 | 0.99828 | 0.627509 |
| Mother's age at death | rs9267445 | 6:31483481_G_C | 0.859769 | 0.032065 | 0.007346 | 1.30E-05 | 0.99458 | 0.791918 |
| Mother's age at death | rs9353229 | 6:85572786_C_G | 0.0742088 | -0.04259 | 0.009785 | 1.30E-05 | 0.98547 | 0.723436 |
| Mother's age at death | rs6017536 | 20:43907369_G_T | 0.765502 | 0.026318 | 0.006038 | 1.30E-05 | 0.9942 | 0.353378 |
| Mother's age at death | rs6032104 | 20:43932412_G_A | 0.778313 | 0.026825 | 0.006145 | 1.30E-05 | 1 | 0.173844 |
| Mother's age at death | rs2743317 | 20:43936931_C_T | 0.780535 | 0.026868 | 0.006173 | 1.30E-05 | 0.99595 | 0.38384 |
| Mother's age at death | rs113770106 | 11:133861409_A_G | 0.948191 | 0.051137 | 0.011738 | 1.30E-05 | 0.95532 | 0.68237 |
| Mother's age at death | rs1639108 | 6:31196554_T_C | 0.844167 | 0.030477 | 0.007027 | 1.40E-05 | 0.99286 | 0.102134 |
| Mother's age at death | rs114658269 | 6:31407380_C_T | 0.978623 | -0.07629 | 0.017551 | 1.40E-05 | 1 | 0.013671 |
| Mother's age at death | rs200744629 | 6:31037872_TTTTC_T | 0.838015 | 0.030372 | 0.006994 | 1.40E-05 | 0.97258 | 0.209411 |
| Mother's age at death | rs3094669 | 6:31103195_C_G | 0.840757 | 0.030222 | 0.006954 | 1.40E-05 | 0.9981 | 0.64288 |
| Mother's age at death | rs150453680 | 6:2847821_CGA_C | 0.921218 | -0.04127 | 0.009502 | 1.40E-05 | 0.988 | 1 |
| Mother's age at death | rs35398572 | 6:2858529_T_C | 0.905708 | -0.03815 | 0.008796 | 1.40E-05 | 0.97834 | 0.893209 |
| Mother's age at death | rs13043296 | 20:43805688_T_C | 0.809824 | 0.028248 | 0.006508 | 1.40E-05 | 0.99408 | 0.670656 |
| Mother's age at death | rs13039213 | 20:43805712_A_C | 0.809824 | 0.028248 | 0.006508 | 1.40E-05 | 0.99408 | 0.670656 |
| Mother's age at death | rs6104093 | 20:43908297_T_C | 0.765766 | 0.026155 | 0.006025 | 1.40E-05 | 0.9993 | 0.361601 |
| Mother's age at death | rs7272597 | 20:43908869_T_C | 0.765487 | 0.026187 | 0.00602 | 1.40E-05 | 1 | 0.401495 |
| Mother's age at death | rs736389 | 20:43946768_T_C | 0.781433 | 0.027398 | 0.006297 | 1.40E-05 | 0.95897 | 0.373224 |
| Mother's age at death | rs149910094 | 2:207640302_TTG_T | 0.916507 | 0.040115 | 0.009246 | 1.40E-05 | 0.99296 | 0.98492 |
| Mother's age at death | rs6706164 | 2:207624673_T_G | 0.915147 | 0.039818 | 0.00916 | 1.40E-05 | 0.997 | 0.940633 |
| Mother's age at death | rs12468081 | 2:207625164_C_A | 0.915147 | 0.039818 | 0.00916 | 1.40E-05 | 0.997 | 0.940633 |
| Mother's age at death | rs61475538 | 2:207626890_T_A | 0.914235 | 0.039638 | 0.009137 | 1.40E-05 | 0.99252 | 0.941207 |
| Mother's age at death | rs60531500 | 2:207626891_C_CA | 0.914238 | 0.039635 | 0.009137 | 1.40E-05 | 0.99249 | 0.970577 |
| Mother's age at death | rs146491677 | 2:142809040_GTTTTTTTGT_G | 0.433351 | -0.02236 | 0.005145 | 1.40E-05 | 0.99666 | 0.514577 |
| Mother's age at death | rs1449485 | 2:142871588_G_A | 0.484834 | -0.02213 | 0.005093 | 1.40E-05 | 0.99798 | 0.931007 |
| Mother's age at death | rs1349222 | 2:142871813_G_A | 0.484516 | -0.02217 | 0.005096 | 1.40E-05 | 0.99695 | 0.917251 |
| Mother's age at death | rs4690075 | 4:3173273_C_T | 0.68362 | -0.02378 | 0.005482 | 1.40E-05 | 0.99663 | 0.088818 |
| Mother's age at death | rs2269477 | 4:3184464_G_T | 0.68427 | -0.02389 | 0.005493 | 1.40E-05 | 0.9942 | 0.07449 |
| Mother's age at death | rs362336 | 4:3213832_G_A | 0.683827 | -0.02375 | 0.005477 | 1.40E-05 | 0.99877 | 0.097814 |
| Mother's age at death | rs2071703 | 4:3241413_A_C | 0.671904 | -0.02359 | 0.005431 | 1.40E-05 | 0.99611 | 0.199315 |
| Mother's age at death | rs7544386 | 1:83485083_T_C | 0.266755 | 0.025164 | 0.005784 | 1.40E-05 | 0.98304 | 0.166759 |
| Mother's age at death | rs1930279 | 1:83486265_C_T | 0.544188 | 0.02243 | 0.00517 | 1.40E-05 | 0.97706 | 0.576979 |
| Mother's age at death | rs76571763 | 4:177974017_C_A | 0.972977 | -0.06817 | 0.015678 | 1.40E-05 | 1 | 0.957005 |
| Mother's age at death | rs6953162 | 7:82378880_A_G | 0.965011 | 0.060237 | 0.013891 | 1.40E-05 | 1 | 0.016214 |
| Mother's age at death | rs6446476 | 4:6213513_C_T | 0.0666568 | -0.0458 | 0.010562 | 1.40E-05 | 0.92745 | 0.419044 |
| Mother's age at death | rs62135453 | 2:49003458_C_T | 0.724627 | 0.025293 | 0.005818 | 1.40E-05 | 0.95295 | 0.054088 |
| Mother's age at death | rs75562474 | 2:61349624_G_A | 0.965454 | -0.06174 | 0.014223 | 1.40E-05 | 0.96039 | 0.204348 |
| Mother's age at death | rs142383786 | 18:9373006_GA_G | 0.898907 | 0.036693 | 0.008453 | 1.40E-05 | 0.9959 | 0.680331 |
| Mother's age at death | rs76540506 | 18:9397264_T_C | 0.898821 | 0.036597 | 0.008432 | 1.40E-05 | 1 | 0.739537 |
| Mother's age at death | rs111890788 | 18:67546603_T_C | 0.948644 | -0.05017 | 0.01156 | 1.40E-05 | 0.98131 | 0.004213 |
| Mother's age at death | rs350207 | 16:12212914_T_G | 0.419169 | 0.022509 | 0.005199 | 1.50E-05 | 0.98667 | 0.197324 |
| Mother's age at death | rs569782909 | 19:48113865_A_C | 0.913351 | -0.04611 | 0.010668 | 1.50E-05 | 0.71917 | 0.70283 |
| Mother's age at death | rs288723 | 13:107495634_C_T | 0.691065 | -0.02388 | 0.00551 | 1.50E-05 | 1 | 0.983828 |
| Mother's age at death | rs78048962 | 18:9374236_G_A | 0.898896 | 0.036621 | 0.008453 | 1.50E-05 | 0.99555 | 0.703688 |
| Mother's age at death | rs78678009 | 18:9374861_C_T | 0.89896 | 0.03655 | 0.008455 | 1.50E-05 | 0.99583 | 0.703574 |
| Mother's age at death | rs140481834 | 18:9375106_G_A | 0.898962 | 0.036593 | 0.008454 | 1.50E-05 | 0.99586 | 0.703542 |
| Mother's age at death | rs77284725 | 18:9375488_A_G | 0.898896 | 0.036621 | 0.008453 | 1.50E-05 | 0.99555 | 0.703688 |
| Mother's age at death | rs77014755 | 18:9377080_G_A | 0.898896 | 0.036621 | 0.008453 | 1.50E-05 | 0.99555 | 0.703688 |
| Mother's age at death | rs143918754 | 18:9377365_A_C | 0.899244 | 0.036712 | 0.008475 | 1.50E-05 | 0.99371 | 0.750585 |
| Mother's age at death | rs11660308 | 18:9377903_G_C | 0.898878 | 0.03659 | 0.008453 | 1.50E-05 | 0.99538 | 0.692038 |
| Mother's age at death | rs35148474 | 18:9390104_T_A | 0.898865 | 0.036515 | 0.008436 | 1.50E-05 | 0.99935 | 0.692086 |
| Mother's age at death | rs28581817 | 14:90070616_C_G | 0.973006 | -0.07011 | 0.016199 | 1.50E-05 | 0.93647 | 0.413181 |
| Mother's age at death | rs2233896 | 20:43850400_C_A | 0.813763 | 0.028332 | 0.006548 | 1.50E-05 | 1 | 0.885519 |
| Mother's age at death | rs13038434 | 20:43888106_G_A | 0.814022 | 0.028413 | 0.006563 | 1.50E-05 | 0.99526 | 0.765738 |
| Mother's age at death | rs17335251 | 20:43902946_T_A | 0.772547 | 0.026399 | 0.006106 | 1.50E-05 | 0.99276 | 0.241596 |
| Mother's age at death | rs142741580 | 20:43936990_A_AGGGGAAGT | 0.775521 | 0.026498 | 0.006129 | 1.50E-05 | 0.99394 | 0.343036 |
| Mother's age at death | rs6096439 | 20:50075337_G_A | 0.860469 | -0.03209 | 0.007426 | 1.50E-05 | 0.97542 | 0.061901 |
| Mother's age at death | rs2233956 | 6:31081205_T_C | 0.778514 | 0.026515 | 0.006117 | 1.50E-05 | 1 | 0.180967 |
| Mother's age at death | rs7750641 | 6:31129310_C_T | 0.844347 | 0.030402 | 0.007022 | 1.50E-05 | 1 | 0.895148 |
| Mother's age at death | rs3094191 | 6:31138910_T_C | 0.758208 | 0.026818 | 0.006187 | 1.50E-05 | 0.92074 | 0.45006 |
| Mother's age at death | rs140953239 | 4:178058681_GT_G | 0.972437 | -0.07204 | 0.016645 | 1.50E-05 | 0.87109 | 0.368723 |
| Mother's age at death | rs61792530 | 4:3145866_T_C | 0.683715 | -0.02374 | 0.005485 | 1.50E-05 | 0.99533 | 0.095192 |
| Mother's age at death | rs363099 | 4:3162056_C_T | 0.683895 | -0.02371 | 0.005482 | 1.50E-05 | 0.99689 | 0.095126 |
| Mother's age at death | rs11276773 | 4:3174496_TGATTCACA_T | 0.684522 | -0.02378 | 0.005487 | 1.50E-05 | 0.99678 | 0.101538 |
| Mother's age at death | rs2298967 | 4:3185747_T_C | 0.683311 | -0.02376 | 0.005483 | 1.50E-05 | 0.99594 | 0.081733 |
| Mother's age at death | rs363088 | 4:3210330_A_T | 0.685071 | -0.02377 | 0.005483 | 1.50E-05 | 0.99818 | 0.099793 |
| Mother's age at death | rs2530598 | 4:3230804_A_C | 0.406609 | 0.022562 | 0.005211 | 1.50E-05 | 0.992 | 0.068976 |
| Mother's age at death | rs3041464 | 2:142800529_T_TGATA | 0.483311 | -0.0221 | 0.005109 | 1.50E-05 | 0.99217 | 0.808422 |
| Mother's age at death | rs34392492 | 2:142820961_T_C | 0.482973 | -0.02212 | 0.005102 | 1.50E-05 | 0.9944 | 0.995394 |
| Mother's age at death | rs115385345 | 2:142837077_T_A | 0.4623 | -0.02234 | 0.005169 | 1.50E-05 | 0.97282 | 0.82106 |
| Mother's age at death | rs13018671 | 2:142844452_G_A | 0.484823 | -0.02203 | 0.005088 | 1.50E-05 | 0.99949 | 0.940186 |
| Mother's age at death | rs68139760 | 2:142850170_T_C | 0.485274 | -0.02201 | 0.005093 | 1.50E-05 | 0.99779 | 0.898961 |
| Mother's age at death | rs12988578 | 2:142877109_A_G | 0.493644 | -0.02208 | 0.005107 | 1.50E-05 | 0.99186 | 0.954008 |
| Mother's age at death | rs1930282 | 1:83488322_C_A | 0.545623 | 0.02239 | 0.005174 | 1.50E-05 | 0.97589 | 0.568879 |
| Mother's age at death | rs10261124 | 7:131527754_C_T | 0.112883 | -0.03488 | 0.00807 | 1.50E-05 | 0.99755 | 0.305499 |
| Mother's age at death | rs182387204 | 7:12660206_C_T | 0.984079 | 0.094515 | 0.021838 | 1.50E-05 | 0.87171 | 0.349333 |
| Mother's age at death | rs185386017 | 7:12673937_C_A | 0.985237 | 0.097658 | 0.022565 | 1.50E-05 | 0.88189 | 0.226647 |
| Mother's age at death | rs140055830 | 7:47637450_C_A | 0.984476 | -0.09565 | 0.022162 | 1.60E-05 | 0.86521 | 0.50946 |
| Mother's age at death | rs17824487 | 2:142819370_G_T | 0.482953 | -0.02197 | 0.005101 | 1.60E-05 | 0.99506 | 0.990787 |
| Mother's age at death | rs11677754 | 2:142819834_A_C | 0.483007 | -0.02201 | 0.0051 | 1.60E-05 | 0.9952 | 0.967763 |
| Mother's age at death | rs2381203 | 2:142880602_A_G | 0.494945 | -0.02208 | 0.00512 | 1.60E-05 | 0.9873 | 0.889914 |
| Mother's age at death | rs6759847 | 2:173968996_C_A | 0.226778 | 0.026263 | 0.006088 | 1.60E-05 | 0.99483 | 0.644199 |
| Mother's age at death | rs4399689 | 2:174030566_A_T | 0.830448 | -0.02916 | 0.006766 | 1.60E-05 | 0.99846 | 0.065842 |
| Mother's age at death | rs62175824 | 2:174067657_T_G | 0.80388 | -0.02768 | 0.006408 | 1.60E-05 | 0.99668 | 0.30406 |
| Mother's age at death | rs7685686 | 4:3207142_A_G | 0.575732 | -0.02232 | 0.005171 | 1.60E-05 | 0.99554 | 0.090053 |
| Mother's age at death | rs72392820 | 4:3249924_CTCCGGT_C | 0.567328 | -0.02231 | 0.005168 | 1.60E-05 | 0.9893 | 0.057796 |
| Mother's age at death | . | 3:185383794_A_G | 0.940919 | -0.05919 | 0.013721 | 1.60E-05 | 0.6155 | 0.694337 |
| Mother's age at death | rs143198425 | 18:9377440_CT_C | 0.899015 | 0.036538 | 0.008462 | 1.60E-05 | 0.99462 | 0.715188 |
| Mother's age at death | rs148151168 | 18:9382352_C_A | 0.89895 | 0.036481 | 0.008454 | 1.60E-05 | 0.99588 | 0.703574 |
| Mother's age at death | rs7359716 | 18:9382768_G_A | 0.898961 | 0.036522 | 0.008454 | 1.60E-05 | 0.99595 | 0.703542 |
| Mother's age at death | rs34413792 | 18:9390245_G_A | 0.898851 | 0.036439 | 0.008439 | 1.60E-05 | 0.99858 | 0.692102 |
| Mother's age at death | rs1641855 | 16:12214171_G_C | 0.418357 | 0.022438 | 0.005198 | 1.60E-05 | 0.98745 | 0.183084 |
| Mother's age at death | rs148585092 | 16:14863345_G_A | 0.971056 | -0.07491 | 0.017362 | 1.60E-05 | 0.76863 | 0.83896 |
| Mother's age at death | rs56235883 | 16:73447977_C_A | 0.661115 | 0.023534 | 0.005449 | 1.60E-05 | 0.97483 | 0.811318 |
| Mother's age at death | rs59105199 | 22:36015593_G_C | 0.988725 | 0.106045 | 0.024578 | 1.60E-05 | 0.96129 | 0.797462 |
| Mother's age at death | rs10503607 | 8:17874480_G_T | 0.933159 | 0.043999 | 0.01021 | 1.60E-05 | 0.99665 | 0.381672 |
| Mother's age at death | rs34819311 | 8:23677896_GA_G | 0.703618 | -0.02417 | 0.005598 | 1.60E-05 | 0.99357 | 0.761711 |
| Mother's age at death | rs11749181 | 5:4311789_T_C | 0.846743 | -0.03035 | 0.007044 | 1.60E-05 | 0.99746 | 0.002725 |
| Mother's age at death | rs17200393 | 6:31389922_C_T | 0.978682 | -0.07597 | 0.01759 | 1.60E-05 | 0.99951 | 0.028418 |
| Mother's age at death | rs1634753 | 6:31306639_C_T | 0.845426 | 0.030757 | 0.007124 | 1.60E-05 | 0.97442 | 0.247042 |
| Mother's age at death | rs3129981 | 6:30758857_C_T | 0.826869 | 0.029066 | 0.006727 | 1.60E-05 | 0.99999 | 1 |
| Mother's age at death | rs111486924 | 6:2848082_A_T | 0.921193 | -0.04094 | 0.009491 | 1.60E-05 | 0.98964 | 0.952733 |
| Mother's age at death | rs11610062 | 12:102534206_A_G | 0.943603 | -0.04844 | 0.011232 | 1.60E-05 | 0.96795 | 0.281469 |
| Mother's age at death | rs9356161 | 6:164291168_T_G | 0.504914 | 0.022052 | 0.005109 | 1.60E-05 | 0.98822 | 0.182776 |
| Mother's age at death | rs9295239 | 6:164299483_A_G | 0.809515 | 0.027936 | 0.006485 | 1.60E-05 | 0.99712 | 0.525739 |
| Mother's age at death | rs9295240 | 6:164299587_A_G | 0.809773 | 0.027975 | 0.00649 | 1.60E-05 | 0.99683 | 0.568814 |
| Mother's age at death | rs9458916 | 6:164302095_T_A | 0.809243 | 0.027933 | 0.006484 | 1.60E-05 | 0.99632 | 0.502192 |
| Mother's age at death | . | 20:43756041_ATATG_A | 0.829029 | 0.030093 | 0.006997 | 1.70E-05 | 0.93081 | 0.628811 |
| Mother's age at death | rs16989763 | 20:43779963_T_C | 0.801279 | 0.027454 | 0.006392 | 1.70E-05 | 0.99243 | 0.493844 |
| Mother's age at death | rs576863822 | 20:43809477_CT_C | 0.79462 | 0.027828 | 0.006477 | 1.70E-05 | 0.94595 | 0.87264 |
| Mother's age at death | rs13252791 | 8:142593586_G_A | 0.298725 | 0.024105 | 0.005608 | 1.70E-05 | 0.97578 | 0.038503 |
| Mother's age at death | rs142882880 | 5:175505003_A_G | 0.985871 | -0.10818 | 0.025168 | 1.70E-05 | 0.74558 | 0.477463 |
| Mother's age at death | rs372580061 | 6:31415485_A_G | 0.979989 | -0.07941 | 0.018478 | 1.70E-05 | 0.96168 | 0.012999 |
| Mother's age at death | rs3769163 | 2:174064176_A_G | 0.803952 | -0.02755 | 0.006409 | 1.70E-05 | 0.99665 | 0.303934 |
| Mother's age at death | rs11687520 | 2:173926771_T_C | 0.828803 | -0.02925 | 0.006799 | 1.70E-05 | 0.98596 | 0.347384 |
| Mother's age at death | rs3820835 | 2:173966589_T_A | 0.795835 | -0.0272 | 0.006325 | 1.70E-05 | 0.99361 | 0.382825 |
| Mother's age at death | rs12621782 | 2:142817716_T_C | 0.483077 | -0.02193 | 0.005101 | 1.70E-05 | 0.99494 | 0.953962 |
| Mother's age at death | rs7558803 | 2:142820874_A_C | 0.483079 | -0.02194 | 0.005101 | 1.70E-05 | 0.9952 | 0.953962 |
| Mother's age at death | rs4662373 | 2:142844780_C_G | 0.484913 | -0.02186 | 0.005089 | 1.70E-05 | 0.99931 | 0.935597 |
| Mother's age at death | rs1375612 | 2:142878385_G_A | 0.493854 | -0.022 | 0.005111 | 1.70E-05 | 0.99038 | 0.935645 |
| Mother's age at death | rs115793979 | 2:61747384_A_G | 0.958032 | -0.05575 | 0.012976 | 1.70E-05 | 0.95074 | 0.386545 |
| Mother's age at death | rs10643592 | 2:45514726_C_CATG | 0.721659 | -0.02541 | 0.005911 | 1.70E-05 | 0.91987 | 0.536537 |
| Mother's age at death | rs11430179 | 4:3111078_T_TA | 0.577179 | -0.02227 | 0.00518 | 1.70E-05 | 0.99294 | 0.049659 |
| Mother's age at death | rs60197694 | 4:3154111_T_C | 0.683484 | -0.02359 | 0.005484 | 1.70E-05 | 0.99551 | 0.092704 |
| Mother's age at death | rs4690011 | 4:3171370_A_G | 0.576103 | -0.02224 | 0.005168 | 1.70E-05 | 0.99665 | 0.082414 |
| Mother's age at death | rs3755883 | 4:3223250_A_G | 0.679623 | -0.02352 | 0.005468 | 1.70E-05 | 0.99718 | 0.021822 |
| Mother's age at death | rs17109586 | 1:83488608_C_T | 0.544854 | 0.0222 | 0.005167 | 1.70E-05 | 0.97824 | 0.584857 |
| Mother's age at death | rs7242298 | 18:9367029_G_A | 0.913825 | 0.039241 | 0.009129 | 1.70E-05 | 0.98499 | 0.755892 |
| Mother's age at death | rs7244170 | 18:9367482_C_T | 0.913832 | 0.039259 | 0.009129 | 1.70E-05 | 0.9851 | 0.755875 |
| Mother's age at death | rs143585237 | 18:9385903_A_G | 0.899026 | 0.036365 | 0.008457 | 1.70E-05 | 0.99583 | 0.668405 |
| Mother's age at death | rs201687269 | 18:27520799_T_A | 0.671773 | 0.027771 | 0.00645 | 1.70E-05 | 0.70362 | 0.432803 |
| Mother's age at death | rs192613495 | 14:76418219_T_A | 0.989055 | 0.119398 | 0.027779 | 1.70E-05 | 0.77389 | 0.231985 |
| Mother's age at death | rs11438068 | 9:83151935_C_CA | 0.203067 | -0.02732 | 0.006343 | 1.70E-05 | 0.99162 | 0.223414 |
| Mother's age at death | rs67660249 | 9:83249463_TATGAGTCTACATATGAGTATATAGACTCC_T | 0.325564 | -0.02338 | 0.005459 | 1.80E-05 | 0.98527 | 0.994758 |
| Mother's age at death | rs12685421 | 9:78471793_A_G | 0.979156 | -0.07587 | 0.017698 | 1.80E-05 | 1 | 0.009988 |
| Mother's age at death | rs12985916 | 19:48101058_G_A | 0.2908 | 0.024682 | 0.00575 | 1.80E-05 | 0.94968 | 0.314572 |
| Mother's age at death | rs137940826 | 18:9386035_C_T | 0.898687 | 0.036214 | 0.008453 | 1.80E-05 | 0.99397 | 0.704255 |
| Mother's age at death | rs55914002 | 16:73447908_A_T | 0.673129 | 0.023564 | 0.005499 | 1.80E-05 | 0.97466 | 0.843706 |
| Mother's age at death | rs350203 | 16:12215791_A_G | 0.419104 | 0.022254 | 0.005185 | 1.80E-05 | 0.99186 | 0.197315 |
| Mother's age at death | rs3130909 | 6:31449710_T_C | 0.857083 | 0.031136 | 0.007261 | 1.80E-05 | 0.99925 | 0.39513 |
| Mother's age at death | rs3132514 | 6:31142758_T_C | 0.782656 | 0.026438 | 0.006158 | 1.80E-05 | 0.99925 | 0.188834 |
| Mother's age at death | rs3131010 | 6:31105147_C_T | 0.840568 | 0.029791 | 0.006949 | 1.80E-05 | 0.99833 | 0.666623 |
| Mother's age at death | rs6930354 | 6:164302584_A_G | 0.809416 | 0.027826 | 0.006489 | 1.80E-05 | 0.99549 | 0.50782 |
| Mother's age at death | rs17333381 | 20:43804919_C_T | 0.808441 | 0.027753 | 0.006473 | 1.80E-05 | 0.99919 | 0.658673 |
| Mother's age at death | rs17424696 | 20:43810734_G_A | 0.807985 | 0.027728 | 0.006471 | 1.80E-05 | 0.99804 | 0.693617 |
| Mother's age at death | rs79759043 | 20:43840755_A_T | 0.814799 | 0.028465 | 0.00663 | 1.80E-05 | 0.97837 | 0.831976 |
| Mother's age at death | . | 20:43845387_CTTTTCTTTTTT_C | 0.818758 | 0.028654 | 0.006689 | 1.80E-05 | 0.97813 | 0.829201 |
| Mother's age at death | rs35284867 | 20:43845836_A_T | 0.813507 | 0.028057 | 0.006542 | 1.80E-05 | 0.99924 | 0.832866 |
| Mother's age at death | rs35751129 | 20:43847658_C_A | 0.813518 | 0.028038 | 0.006542 | 1.80E-05 | 0.9993 | 0.84035 |
| Mother's age at death | rs6104092 | 20:43906730_G_A | 0.765979 | 0.025968 | 0.006047 | 1.80E-05 | 0.9925 | 0.352785 |
| Mother's age at death | rs6698372 | 1:83487372_T_C | 0.544823 | 0.022208 | 0.005172 | 1.80E-05 | 0.97679 | 0.621312 |
| Mother's age at death | rs7575319 | 2:174073287_A_G | 0.80523 | -0.0275 | 0.006421 | 1.80E-05 | 0.99774 | 0.268176 |
| Mother's age at death | rs362268 | 4:3242148_C_G | 0.682709 | -0.02344 | 0.005471 | 1.80E-05 | 0.99793 | 0.174056 |
| Mother's age at death | rs1263341 | 4:2921937_G_A | 0.49891 | 0.021945 | 0.005133 | 1.90E-05 | 0.98372 | 0.426148 |
| Mother's age at death | rs62315406 | 4:132664422_A_G | 0.19846 | 0.027793 | 0.0065 | 1.90E-05 | 0.96329 | 0.278465 |
| Mother's age at death | rs3769215 | 2:173905497_C_T | 0.791661 | -0.02711 | 0.006344 | 1.90E-05 | 0.97327 | 0.294938 |
| Mother's age at death | rs59795626 | 2:207619558_G_A | 0.91579 | 0.039386 | 0.0092 | 1.90E-05 | 0.99472 | 0.940228 |
| Mother's age at death | rs6708403 | 2:207622502_A_T | 0.916611 | 0.03956 | 0.009255 | 1.90E-05 | 0.99136 | 0.89467 |
| Mother's age at death | rs12998297 | 2:142811885_C_T | 0.484158 | -0.02184 | 0.005103 | 1.90E-05 | 0.99408 | 0.867056 |
| Mother's age at death | rs11688838 | 2:142819607_C_T | 0.482904 | -0.02182 | 0.005101 | 1.90E-05 | 0.9951 | 0.995393 |
| Mother's age at death | rs7598886 | 2:142820310_T_A | 0.483297 | -0.02182 | 0.005103 | 1.90E-05 | 0.99442 | 0.92182 |
| Mother's age at death | rs56030807 | 2:142836449_G_C | 0.483823 | -0.02174 | 0.005089 | 1.90E-05 | 0.99943 | 0.921826 |
| Mother's age at death | rs7566250 | 2:142845567_C_A | 0.484397 | -0.02182 | 0.005097 | 1.90E-05 | 0.99593 | 0.98158 |
| Mother's age at death | rs62155638 | 2:142879572_G_A | 0.494617 | -0.0219 | 0.005116 | 1.90E-05 | 0.98828 | 0.981595 |
| Mother's age at death | rs357974 | 2:3796132_G_C | 0.603931 | -0.02236 | 0.005232 | 1.90E-05 | 0.98951 | 0.781772 |
| Mother's age at death | rs113420217 | 2:10837820_C_G | 0.940888 | 0.05224 | 0.01223 | 1.90E-05 | 0.77508 | 0.87645 |
| Mother's age at death | rs41332550 | 2:33748025_T_C | 0.904669 | 0.037072 | 0.008658 | 1.90E-05 | 1 | 0.750464 |
| Mother's age at death | rs7038336 | 9:83154554_G_A | 0.203792 | -0.02704 | 0.00632 | 1.90E-05 | 0.99626 | 0.252947 |
| Mother's age at death | rs6559557 | 9:83155958_A_G | 0.203035 | -0.02702 | 0.006326 | 1.90E-05 | 0.99685 | 0.237105 |
| Mother's age at death | rs4877180 | 9:83157705_A_G | 0.203413 | -0.02707 | 0.006321 | 1.90E-05 | 0.99695 | 0.224042 |
| Mother's age at death | rs139989 | 22:36858174_A_G | 0.909013 | 0.038138 | 0.008924 | 1.90E-05 | 0.98719 | 0.120834 |
| Mother's age at death | rs11081475 | 18:9366356_G_A | 0.913753 | 0.039062 | 0.009133 | 1.90E-05 | 0.98357 | 0.756032 |
| Mother's age at death | rs62080630 | 18:14454213_A_G | 0.824451 | -0.03002 | 0.007023 | 1.90E-05 | 0.90999 | 0.454454 |
| Mother's age at death | rs139978527 | 20:43800492_TTGCATTAATATGTCATGACATATTTA_T | 0.833164 | 0.029616 | 0.006935 | 1.90E-05 | 0.96857 | 0.933271 |
| Mother's age at death | rs13042522 | 20:43829290_G_A | 0.810095 | 0.027871 | 0.006512 | 1.90E-05 | 0.99297 | 0.747739 |
| Mother's age at death | rs6017514 | 20:43844308_C_G | 0.814222 | 0.028061 | 0.006563 | 1.90E-05 | 0.99573 | 0.817403 |
| Mother's age at death | rs2743347 | 20:43920493_T_C | 0.766856 | 0.025903 | 0.006061 | 1.90E-05 | 0.99138 | 0.381689 |
| Mother's age at death | rs2743221 | 20:43927602_G_A | 0.772783 | 0.026059 | 0.006089 | 1.90E-05 | 0.999 | 0.338797 |
| Mother's age at death | rs78618990 | 20:43928604_G_T | 0.772657 | 0.026058 | 0.006089 | 1.90E-05 | 0.99874 | 0.32664 |
| Mother's age at death | rs2233093 | 20:43933196_C_T | 0.772664 | 0.026067 | 0.006089 | 1.90E-05 | 0.99916 | 0.25872 |
| Mother's age at death | rs130076 | 6:31122482_G_A | 0.754435 | 0.025264 | 0.005912 | 1.90E-05 | 1 | 0.882468 |
| Mother's age at death | rs191827672 | 6:31383119_G_A | 0.979728 | -0.07856 | 0.018396 | 1.90E-05 | 0.96843 | 0.427612 |
| Mother's age at death | rs3130908 | 6:31449552_T_C | 0.856997 | 0.03104 | 0.007259 | 1.90E-05 | 0.99952 | 0.408602 |
| Mother's age at death | rs2397312 | 6:57329158_G_A | 0.962647 | -0.06458 | 0.015087 | 1.90E-05 | 0.79713 | 0.87313 |
| Mother's age at death | rs17034271 | 12:104046045_T_C | 0.963805 | 0.060362 | 0.014116 | 1.90E-05 | 0.92863 | 0.743533 |
| Mother's age at death | rs13042431 | 20:43781253_A_G | 0.801536 | 0.027312 | 0.006396 | 2.00E-05 | 0.99191 | 0.47631 |
| Mother's age at death | rs6017511 | 20:43833132_C_T | 0.808356 | 0.027585 | 0.006469 | 2.00E-05 | 0.99983 | 0.714151 |
| Mother's age at death | rs17334064 | 20:43839197_T_C | 0.808577 | 0.027636 | 0.006483 | 2.00E-05 | 0.99714 | 0.873028 |
| Mother's age at death | rs62208441 | 20:43839360_T_G | 0.803768 | 0.027528 | 0.006451 | 2.00E-05 | 0.98722 | 0.796213 |
| Mother's age at death | rs6032061 | 20:43853276_C_T | 0.813574 | 0.027894 | 0.006544 | 2.00E-05 | 0.99936 | 0.862893 |
| Mother's age at death | rs6032066 | 20:43857700_C_T | 0.81364 | 0.027939 | 0.006547 | 2.00E-05 | 0.99877 | 0.893124 |
| Mother's age at death | rs35875037 | 20:43859921_A_G | 0.813553 | 0.027884 | 0.006544 | 2.00E-05 | 0.9991 | 0.855374 |
| Mother's age at death | rs6032067 | 20:43864809_G_T | 0.813514 | 0.027913 | 0.006546 | 2.00E-05 | 0.99831 | 0.85539 |
| Mother's age at death | rs2743348 | 20:43920523_A_G | 0.767033 | 0.025822 | 0.00606 | 2.00E-05 | 0.99215 | 0.381461 |
| Mother's age at death | rs11441867 | 11:20398675_C_CTT | 0.0550652 | 0.048103 | 0.011292 | 2.00E-05 | 0.97361 | 0.782348 |
| Mother's age at death | rs310297 | 8:23667658_C_T | 0.70278 | -0.02389 | 0.005598 | 2.00E-05 | 0.9917 | 0.72046 |
| Mother's age at death | rs566429 | 8:23696543_C_T | 0.706915 | -0.02423 | 0.005682 | 2.00E-05 | 0.97012 | 0.488 |
| Mother's age at death | rs1639109 | 6:31194583_G_A | 0.846026 | 0.030126 | 0.007062 | 2.00E-05 | 0.99222 | 0.086 |
| Mother's age at death | rs217264 | 5:137446998_T_C | 0.624765 | 0.022657 | 0.005315 | 2.00E-05 | 0.96445 | 1.02E-05 |
| Mother's age at death | rs486799 | 5:137472384_C_T | 0.632179 | 0.022914 | 0.00537 | 2.00E-05 | 0.96695 | 0.663957 |
| Mother's age at death | rs486835 | 5:137472398_C_T | 0.635189 | 0.022913 | 0.005378 | 2.00E-05 | 0.96627 | 0.784043 |
| Mother's age at death | rs3034863 | 4:2922508_CTT_C | 0.498873 | 0.021897 | 0.005133 | 2.00E-05 | 0.98367 | 0.422795 |
| Mother's age at death | rs362306 | 4:3242100_G_A | 0.683666 | -0.02336 | 0.005479 | 2.00E-05 | 0.9972 | 0.123166 |
| Mother's age at death | rs11163637 | 1:83482072_G_C | 0.545153 | 0.022083 | 0.005182 | 2.00E-05 | 0.97217 | 0.489148 |
| Mother's age at death | rs61261476 | 7:82380287_CT_C | 0.928811 | 0.048088 | 0.011283 | 2.00E-05 | 0.77146 | 0.606415 |
| Mother's age at death | rs1449490 | 2:142796193_C_G | 0.482777 | -0.02177 | 0.005107 | 2.00E-05 | 0.99307 | 0.742096 |
| Mother's age at death | rs1900933 | 2:142812720_A_C | 0.483855 | -0.02175 | 0.005105 | 2.00E-05 | 0.9932 | 0.926412 |
| Mother's age at death | rs9941495 | 2:142820271_T_A | 0.483134 | -0.02177 | 0.005104 | 2.00E-05 | 0.99426 | 0.940173 |
| Mother's age at death | rs6754907 | 2:142823667_A_C | 0.484313 | -0.02169 | 0.005092 | 2.00E-05 | 0.99814 | 0.926416 |
| Mother's age at death | rs957376 | 2:142840924_C_T | 0.483748 | -0.02169 | 0.005089 | 2.00E-05 | 0.99916 | 0.944774 |
| Mother's age at death | rs10803488 | 2:142843214_G_T | 0.484059 | -0.02168 | 0.005089 | 2.00E-05 | 0.9994 | 0.931001 |
| Mother's age at death | rs3820838 | 2:173905103_G_A | 0.791595 | -0.02705 | 0.006347 | 2.00E-05 | 0.97219 | 0.299102 |
| Mother's age at death | rs73985728 | 2:207668245_G_A | 0.930558 | 0.042871 | 0.010056 | 2.00E-05 | 0.99089 | 0.637647 |
| Mother's age at death | rs6559558 | 9:83156021_A_G | 0.203152 | -0.02695 | 0.006324 | 2.00E-05 | 0.99711 | 0.230351 |
| Mother's age at death | rs10683311 | 9:83156313_C_CCTT | 0.203143 | -0.02697 | 0.006323 | 2.00E-05 | 0.99729 | 0.220135 |
| Mother's age at death | rs8181094 | 9:83158347_C_T | 0.203124 | -0.02698 | 0.006324 | 2.00E-05 | 0.99734 | 0.237279 |
| Mother's age at death | rs10780424 | 9:83158820_A_G | 0.203136 | -0.02696 | 0.006324 | 2.00E-05 | 0.99735 | 0.240841 |
| Mother's age at death | rs10867573 | 9:83166447_T_G | 0.203647 | -0.02701 | 0.006331 | 2.00E-05 | 0.99359 | 0.320229 |
| Mother's age at death | rs1328502 | 9:83167757_T_G | 0.305656 | -0.02362 | 0.005538 | 2.00E-05 | 0.99092 | 0.106458 |
| Mother's age at death | rs77756848 | 21:17226780_T_A | 0.958957 | -0.05524 | 0.01296 | 2.00E-05 | 0.97907 | 0.244765 |
| Mother's age at death | rs80087526 | 18:9349207_T_A | 0.898808 | 0.036194 | 0.008503 | 2.10E-05 | 0.98345 | 0.763291 |
| Mother's age at death | rs720818 | 18:9403277_C_T | 0.89897 | 0.036077 | 0.00849 | 2.10E-05 | 0.98717 | 0.691799 |
| Mother's age at death | rs112477960 | 16:73449806_G_A | 0.674871 | 0.023488 | 0.005525 | 2.10E-05 | 0.96818 | 0.802221 |
| Mother's age at death | rs10118161 | 9:79763338_C_T | 0.905351 | -0.0368 | 0.008662 | 2.10E-05 | 1 | 0.070291 |
| Mother's age at death | rs74383715 | 22:36859051_A_G | 0.914841 | 0.039258 | 0.009232 | 2.10E-05 | 0.98144 | 0.045509 |
| Mother's age at death | rs147834876 | 5:137038879_C_CA | 0.8432 | 0.03001 | 0.007048 | 2.10E-05 | 0.98084 | 0.022548 |
| Mother's age at death | rs217265 | 5:137449954_C_T | 0.624491 | 0.022599 | 0.005316 | 2.10E-05 | 0.96379 | 9.98E-06 |
| Mother's age at death | rs10041215 | 5:137473517_G_A | 0.62807 | 0.022464 | 0.005281 | 2.10E-05 | 0.99735 | 0.271456 |
| Mother's age at death | rs150767541 | 6:2853667_G_C | 0.919367 | -0.03997 | 0.009396 | 2.10E-05 | 0.98842 | 0.922812 |
| Mother's age at death | rs34522873 | 20:43794338_TA_T | 0.800728 | 0.027171 | 0.00638 | 2.10E-05 | 0.99468 | 0.529871 |
| Mother's age at death | rs34885285 | 20:43804789_C_A | 0.809054 | 0.027604 | 0.006495 | 2.10E-05 | 0.99543 | 0.755875 |
| Mother's age at death | rs17424474 | 20:43804797_C_A | 0.80904 | 0.027624 | 0.006495 | 2.10E-05 | 0.99524 | 0.784693 |
| Mother's age at death | rs1997892 | 20:43810961_T_A | 0.808297 | 0.027562 | 0.006474 | 2.10E-05 | 0.99841 | 0.714167 |
| Mother's age at death | rs6017504 | 20:43818920_G_A | 0.808433 | 0.027543 | 0.006475 | 2.10E-05 | 0.99826 | 0.679297 |
| Mother's age at death | rs6032044 | 20:43819416_C_A | 0.80845 | 0.027539 | 0.006475 | 2.10E-05 | 0.99819 | 0.721008 |
| Mother's age at death | rs13039108 | 20:43829584_T_C | 0.80842 | 0.027576 | 0.006475 | 2.10E-05 | 0.99782 | 0.707082 |
| Mother's age at death | rs6032052 | 20:43829883_C_A | 0.808312 | 0.02749 | 0.006469 | 2.10E-05 | 1 | 0.799842 |
| Mother's age at death | rs2233883 | 20:43835822_T_C | 0.809329 | 0.027577 | 0.006489 | 2.10E-05 | 0.99717 | 0.671352 |
| Mother's age at death | rs62205501 | 20:43864089_G_A | 0.813465 | 0.027849 | 0.006545 | 2.10E-05 | 0.99862 | 0.855423 |
| Mother's age at death | rs6032069 | 20:43867388_T_C | 0.813465 | 0.027849 | 0.006545 | 2.10E-05 | 0.99862 | 0.855423 |
| Mother's age at death | rs6017526 | 20:43871555_A_T | 0.813472 | 0.027833 | 0.006546 | 2.10E-05 | 0.99837 | 0.855423 |
| Mother's age at death | rs310296 | 8:23664441_C_T | 0.703121 | -0.02377 | 0.005593 | 2.10E-05 | 0.99418 | 0.710035 |
| Mother's age at death | rs12191893 | 6:155759200_T_C | 0.719902 | -0.02471 | 0.005804 | 2.10E-05 | 0.95007 | 0.926113 |
| Mother's age at death | rs73055335 | 2:207664690_A_G | 0.913382 | 0.038654 | 0.009097 | 2.10E-05 | 0.99314 | 0.742181 |
| Mother's age at death | rs71230925 | 2:207620639_CTT_C | 0.913948 | 0.038936 | 0.009151 | 2.10E-05 | 0.98582 | 0.926736 |
| Mother's age at death | rs200841668 | 2:207623684_TTTG_T | 0.916557 | 0.039412 | 0.009275 | 2.10E-05 | 0.98723 | 0.939702 |
| Mother's age at death | rs111703822 | 2:207623776_GGTTT_G | 0.915636 | 0.039047 | 0.009189 | 2.10E-05 | 0.99564 | 0.955228 |
| Mother's age at death | rs61674203 | 2:207626284_A_G | 0.915309 | 0.039011 | 0.009174 | 2.10E-05 | 0.99601 | 0.896113 |
| Mother's age at death | rs1449478 | 2:142827984_C_T | 0.484348 | -0.02166 | 0.005089 | 2.10E-05 | 0.99912 | 0.995394 |
| Mother's age at death | rs74060011 | 1:21918463_T_C | 0.857004 | -0.03116 | 0.007329 | 2.10E-05 | 0.98024 | 0.405555 |
| Mother's age at death | rs17658206 | 7:127174748_C_T | 0.375152 | 0.022278 | 0.005233 | 2.10E-05 | 0.99776 | 0.005894 |
| Mother's age at death | rs113646072 | 2:61712011_A_C | 0.957585 | -0.05441 | 0.012777 | 2.10E-05 | 0.97071 | 0.299861 |
| Mother's age at death | rs1140085 | 4:3015553_G_A | 0.854814 | -0.03074 | 0.007228 | 2.10E-05 | 1 | 0.092704 |
| Mother's age at death | rs362275 | 4:3224602_C_T | 0.68291 | -0.02331 | 0.005475 | 2.10E-05 | 0.99849 | 0.060131 |
| Mother's age at death | rs112305536 | 1:175035778_G_A | 0.984972 | 0.090039 | 0.021172 | 2.10E-05 | 0.96986 | 0.549338 |
| Mother's age at death | rs7691627 | 4:3111410_G_A | 0.57736 | -0.02196 | 0.005173 | 2.20E-05 | 0.99527 | 0.054639 |
| Mother's age at death | rs363066 | 4:3135953_T_G | 0.695727 | -0.02351 | 0.005544 | 2.20E-05 | 0.99422 | 0.144398 |
| Mother's age at death | rs11409456 | 4:3136695_C_CA | 0.576935 | -0.02197 | 0.005173 | 2.20E-05 | 0.99508 | 0.060145 |
| Mother's age at death | rs2298971 | 4:3200079_A_G | 0.580102 | -0.02194 | 0.00517 | 2.20E-05 | 0.99752 | 0.135531 |
| Mother's age at death | rs82334 | 4:3225371_A_C | 0.682312 | -0.02324 | 0.005472 | 2.20E-05 | 0.9984 | 0.068061 |
| Mother's age at death | rs10719137 | 2:142825843_GC_G | 0.484905 | -0.02159 | 0.005091 | 2.20E-05 | 0.99815 | 1 |
| Mother's age at death | rs1449477 | 2:142827908_C_A | 0.484621 | -0.02159 | 0.00509 | 2.20E-05 | 0.99882 | 0.931005 |
| Mother's age at death | rs13000270 | 2:142836915_C_G | 0.483735 | -0.02161 | 0.005089 | 2.20E-05 | 0.99931 | 0.903512 |
| Mother's age at death | rs1375613 | 2:142874253_T_A | 0.503199 | -0.02167 | 0.005104 | 2.20E-05 | 0.99209 | 0.871722 |
| Mother's age at death | rs7568962 | 2:207612424_C_T | 0.915809 | 0.039055 | 0.009214 | 2.20E-05 | 0.99173 | 0.940206 |
| Mother's age at death | rs4141409 | 7:127166870_T_G | 0.375206 | 0.022199 | 0.005231 | 2.20E-05 | 0.99808 | 0.004974 |
| Mother's age at death | rs7515386 | 1:21921736_G_A | 0.857495 | -0.03116 | 0.007335 | 2.20E-05 | 0.98109 | 0.378164 |
| Mother's age at death | rs77930325 | 3:37201542_G_A | 0.975429 | -0.0875 | 0.020631 | 2.20E-05 | 0.63946 | 1 |
| Mother's age at death | rs12357710 | 10:72729877_G_A | 0.916042 | -0.03911 | 0.00921 | 2.20E-05 | 0.99248 | 0.499011 |
| Mother's age at death | rs7208561 | 17:17615528_A_G | 0.554779 | -0.02183 | 0.005143 | 2.20E-05 | 0.99467 | 0.543976 |
| Mother's age at death | rs138824476 | 16:30325213_C_T | 0.982882 | -0.08723 | 0.020568 | 2.20E-05 | 0.91336 | 0.500415 |
| Mother's age at death | rs4835675 | 5:137623683_T_C | 0.653613 | 0.022785 | 0.005375 | 2.20E-05 | 0.99462 | 0.256667 |
| Mother's age at death | rs75792163 | 5:137635947_C_T | 0.890685 | 0.034786 | 0.0082 | 2.20E-05 | 0.99451 | 0.504255 |
| Mother's age at death | rs3132534 | 6:31116636_G_A | 0.753537 | 0.025045 | 0.005897 | 2.20E-05 | 0.99964 | 0.331872 |
| Mother's age at death | rs1619179 | 6:31175946_A_C | 0.844733 | 0.029819 | 0.007035 | 2.20E-05 | 0.99534 | 0.285733 |
| Mother's age at death | rs2523607 | 6:31322790_T_A | 0.849654 | 0.030218 | 0.007128 | 2.20E-05 | 0.9995 | 0.595155 |
| Mother's age at death | rs2596495 | 6:31323416_G_C | 0.849661 | 0.030217 | 0.007129 | 2.20E-05 | 0.99925 | 0.626859 |
| Mother's age at death | rs528186543 | 6:31323500_CCT_C | 0.849732 | 0.030288 | 0.007132 | 2.20E-05 | 0.99875 | 0.650969 |
| Mother's age at death | rs4990036 | 6:31323506_C_T | 0.849733 | 0.030291 | 0.007132 | 2.20E-05 | 0.99876 | 0.650969 |
| Mother's age at death | rs115297016 | 6:31402863_C_A | 0.978854 | -0.07552 | 0.017795 | 2.20E-05 | 0.99357 | 0.268405 |
| Mother's age at death | rs116080241 | 6:31404361_A_C | 0.978854 | -0.07552 | 0.017795 | 2.20E-05 | 0.99359 | 0.268405 |
| Mother's age at death | rs73404267 | 6:31412836_T_C | 0.97861 | -0.07451 | 0.017557 | 2.20E-05 | 1 | 0.034049 |
| Mother's age at death | rs3095233 | 6:31469774_A_G | 0.857714 | 0.030859 | 0.007279 | 2.20E-05 | 0.99992 | 0.695889 |
| Mother's age at death | rs310305 | 8:23650620_A_T | 0.702328 | -0.02367 | 0.005582 | 2.20E-05 | 0.99604 | 0.906979 |
| Mother's age at death | rs429371 | 8:23654833_G_C | 0.702479 | -0.02371 | 0.005585 | 2.20E-05 | 0.99561 | 0.836577 |
| Mother's age at death | rs218871 | 8:23655756_T_C | 0.702559 | -0.02365 | 0.005575 | 2.20E-05 | 1 | 0.610801 |
| Mother's age at death | rs310290 | 8:23657060_C_G | 0.702713 | -0.0237 | 0.005582 | 2.20E-05 | 0.99719 | 0.762132 |
| Mother's age at death | rs310300 | 8:23673722_T_A | 0.702863 | -0.02375 | 0.005596 | 2.20E-05 | 0.99249 | 0.793707 |
| Mother's age at death | rs310316 | 8:23677172_A_C | 0.702499 | -0.02375 | 0.005593 | 2.20E-05 | 0.99319 | 0.751779 |
| Mother's age at death | rs16989769 | 20:43780475_C_T | 0.800776 | 0.027142 | 0.006392 | 2.20E-05 | 0.99078 | 0.506186 |
| Mother's age at death | rs45461302 | 20:43805274_G_A | 0.812473 | 0.027855 | 0.006569 | 2.20E-05 | 0.98639 | 0.77439 |
| Mother's age at death | rs11699850 | 20:43809049_T_C | 0.80819 | 0.027472 | 0.006472 | 2.20E-05 | 0.99872 | 0.714282 |
| Mother's age at death | rs17424668 | 20:43809647_A_G | 0.80819 | 0.027472 | 0.006472 | 2.20E-05 | 0.99872 | 0.714282 |
| Mother's age at death | rs17333555 | 20:43809761_G_A | 0.808206 | 0.027471 | 0.006472 | 2.20E-05 | 0.99863 | 0.707261 |
| Mother's age at death | rs876835 | 20:43827241_T_C | 0.808353 | 0.027449 | 0.006471 | 2.20E-05 | 0.9993 | 0.714134 |
| Mother's age at death | rs62208428 | 20:43827578_A_C | 0.808353 | 0.027449 | 0.006471 | 2.20E-05 | 0.9993 | 0.714134 |
| Mother's age at death | rs6032053 | 20:43829935_G_T | 0.8084 | 0.027456 | 0.006469 | 2.20E-05 | 0.99998 | 0.714102 |
| Mother's age at death | rs6017510 | 20:43831785_A_G | 0.8084 | 0.027456 | 0.006469 | 2.20E-05 | 0.99998 | 0.714102 |
| Mother's age at death | rs13037087 | 20:43834313_T_C | 0.8084 | 0.027456 | 0.006469 | 2.20E-05 | 0.99998 | 0.714102 |
| Mother's age at death | rs6017512 | 20:43835038_G_A | 0.808591 | 0.02745 | 0.006473 | 2.20E-05 | 0.9996 | 0.806865 |
| Mother's age at death | rs6017513 | 20:43835232_A_C | 0.8084 | 0.027456 | 0.006469 | 2.20E-05 | 0.99998 | 0.714102 |
| Mother's age at death | rs2233882 | 20:43835683_T_A | 0.808397 | 0.02746 | 0.006469 | 2.20E-05 | 0.99996 | 0.721124 |
| Mother's age at death | rs56131549 | 20:43842014_A_T | 0.808335 | 0.027441 | 0.006472 | 2.20E-05 | 0.99913 | 0.799853 |
| Mother's age at death | rs6032065 | 20:43855649_C_T | 0.81354 | 0.027786 | 0.006544 | 2.20E-05 | 0.99924 | 0.878021 |
| Mother's age at death | rs36032462 | 20:43873088_T_C | 0.813414 | 0.02779 | 0.006546 | 2.20E-05 | 0.99796 | 0.908404 |
| Mother's age at death | rs62205505 | 20:43874856_C_T | 0.81349 | 0.027777 | 0.006546 | 2.20E-05 | 0.99831 | 0.870488 |
| Mother's age at death | rs2743353 | 20:43920730_A_G | 0.766331 | 0.025664 | 0.006046 | 2.20E-05 | 0.9947 | 0.364981 |
| Mother's age at death | rs78227987 | 11:20393323_T_A | 0.956152 | -0.05366 | 0.01265 | 2.20E-05 | 0.96444 | 0.836267 |
| Mother's age at death | rs72429444 | 11:10244669_ATACTT_A | 0.978882 | -0.0777 | 0.018366 | 2.30E-05 | 0.92808 | 0.94412 |
| Mother's age at death | rs6032033 | 20:43795081_G_C | 0.801344 | 0.026986 | 0.00638 | 2.30E-05 | 0.99677 | 0.493719 |
| Mother's age at death | rs13038355 | 20:43805555_C_T | 0.808142 | 0.027397 | 0.006468 | 2.30E-05 | 0.99959 | 0.714315 |
| Mother's age at death | rs13043503 | 20:43805835_T_C | 0.808113 | 0.027389 | 0.006468 | 2.30E-05 | 0.99969 | 0.714348 |
| Mother's age at death | rs17424578 | 20:43806321_A_G | 0.808113 | 0.027389 | 0.006468 | 2.30E-05 | 0.99969 | 0.714348 |
| Mother's age at death | rs17424613 | 20:43806441_C_T | 0.808113 | 0.027389 | 0.006468 | 2.30E-05 | 0.99969 | 0.714348 |
| Mother's age at death | rs17424723 | 20:43814520_T_G | 0.808252 | 0.027381 | 0.006475 | 2.30E-05 | 0.99775 | 0.73536 |
| Mother's age at death | rs17424868 | 20:43818519_T_G | 0.808211 | 0.027394 | 0.006473 | 2.30E-05 | 0.99807 | 0.756763 |
| Mother's age at death | rs2143944 | 20:43819370_G_A | 0.807815 | 0.027406 | 0.00647 | 2.30E-05 | 0.99726 | 0.659569 |
| Mother's age at death | rs35880287 | 20:43821001_G_A | 0.808294 | 0.027418 | 0.006472 | 2.30E-05 | 0.99852 | 0.721207 |
| Mother's age at death | rs35475980 | 20:43821080_A_T | 0.808284 | 0.027398 | 0.006472 | 2.30E-05 | 0.99855 | 0.728249 |
| Mother's age at death | rs6032049 | 20:43825556_C_T | 0.808298 | 0.027428 | 0.006473 | 2.30E-05 | 0.99852 | 0.721191 |
| Mother's age at death | rs2868238 | 20:43826993_C_G | 0.808284 | 0.027399 | 0.006472 | 2.30E-05 | 0.99855 | 0.728249 |
| Mother's age at death | rs6017509 | 20:43830362_A_G | 0.808419 | 0.027402 | 0.00647 | 2.30E-05 | 0.99992 | 0.735208 |
| Mother's age at death | rs139422059 | 20:43840784_AAG_A | 0.811494 | 0.027847 | 0.006581 | 2.30E-05 | 0.97791 | 0.614384 |
| Mother's age at death | rs2233898 | 20:43850525_A_G | 0.814359 | 0.027823 | 0.006565 | 2.30E-05 | 0.99604 | 0.908025 |
| Mother's age at death | rs6032092 | 20:43911886_A_C | 0.765914 | 0.02555 | 0.006035 | 2.30E-05 | 0.99633 | 0.40994 |
| Mother's age at death | rs73269969 | 20:50071955_C_T | 0.860807 | -0.0315 | 0.007435 | 2.30E-05 | 0.97518 | 0.075998 |
| Mother's age at death | rs78231416 | 20:12689741_T_C | 0.872337 | -0.03296 | 0.007789 | 2.30E-05 | 0.96172 | 0.389153 |
| Mother's age at death | rs74625731 | 20:12699125_G_C | 0.86844 | -0.03199 | 0.007564 | 2.30E-05 | 0.99513 | 0.763361 |
| Mother's age at death | rs66999338 | 6:147959896_ATG_A | 0.989542 | -0.11766 | 0.027768 | 2.30E-05 | 0.81274 | 0.075422 |
| Mother's age at death | rs116476029 | 11:133866784_C_A | 0.949013 | 0.049767 | 0.011759 | 2.30E-05 | 0.96607 | 0.677891 |
| Mother's age at death | rs62502207 | 8:23663200_C_T | 0.768108 | -0.02579 | 0.006095 | 2.30E-05 | 0.98061 | 0.859014 |
| Mother's age at death | rs3130616 | 6:31473746_G_A | 0.8577 | 0.030839 | 0.007278 | 2.30E-05 | 0.99991 | 0.695905 |
| Mother's age at death | rs1062070 | 6:32148031_A_G | 0.79941 | 0.026871 | 0.006345 | 2.30E-05 | 1 | 0.589142 |
| Mother's age at death | rs3094012 | 6:31434520_G_C | 0.851894 | 0.030328 | 0.007161 | 2.30E-05 | 0.9995 | 0.70557 |
| Mother's age at death | rs7725421 | 5:4293537_T_C | 0.845867 | -0.02988 | 0.007058 | 2.30E-05 | 0.9894 | 0.006805 |
| Mother's age at death | rs55737935 | 2:207618702_T_G | 0.915524 | 0.038883 | 0.009178 | 2.30E-05 | 0.99679 | 0.940394 |
| Mother's age at death | rs57808141 | 2:207619167_G_A | 0.915531 | 0.038887 | 0.009178 | 2.30E-05 | 0.99679 | 0.94039 |
| Mother's age at death | rs59358632 | 2:207619779_A_G | 0.915531 | 0.038887 | 0.009178 | 2.30E-05 | 0.99679 | 0.94039 |
| Mother's age at death | rs16838824 | 2:207620803_T_C | 0.915531 | 0.038887 | 0.009178 | 2.30E-05 | 0.99679 | 0.94039 |
| Mother's age at death | rs73983102 | 2:207621140_T_C | 0.915436 | 0.038821 | 0.009178 | 2.30E-05 | 0.99588 | 0.955318 |
| Mother's age at death | rs59474646 | 2:207621409_A_G | 0.915531 | 0.038887 | 0.009178 | 2.30E-05 | 0.99679 | 0.94039 |
| Mother's age at death | rs73070029 | 2:207622972_A_G | 0.915531 | 0.038887 | 0.009178 | 2.30E-05 | 0.99679 | 0.94039 |
| Mother's age at death | rs570032427 | 2:207623495_C_CAAAAAA | 0.915524 | 0.038883 | 0.009178 | 2.30E-05 | 0.99679 | 0.940394 |
| Mother's age at death | rs200119136 | 2:207701415_GTATAT_G | 0.931484 | 0.042772 | 0.010106 | 2.30E-05 | 0.99355 | 0.69989 |
| Mother's age at death | rs12463410 | 2:207715608_C_T | 0.930986 | 0.04258 | 0.01005 | 2.30E-05 | 0.99799 | 0.701665 |
| Mother's age at death | rs4972532 | 2:174070578_G_A | 0.195288 | 0.027198 | 0.006419 | 2.30E-05 | 0.99616 | 0.285371 |
| Mother's age at death | rs372158708 | 2:174090332_A_AAC | 0.716003 | -0.02456 | 0.005807 | 2.30E-05 | 0.94149 | 0.1726 |
| Mother's age at death | rs35080458 | 2:48992663_T_C | 0.679526 | 0.023323 | 0.005514 | 2.30E-05 | 0.97349 | 0.245946 |
| Mother's age at death | rs363064 | 4:3141410_C_T | 0.696141 | -0.02349 | 0.005544 | 2.30E-05 | 0.99556 | 0.165863 |
| Mother's age at death | rs6844859 | 4:3190486_T_C | 0.576733 | -0.02185 | 0.005167 | 2.30E-05 | 0.99721 | 0.077215 |
| Mother's age at death | rs7515277 | 1:21921625_G_T | 0.857367 | -0.03102 | 0.00733 | 2.30E-05 | 0.98194 | 0.391336 |
| Mother's age at death | rs17208015 | 18:67557034_A_G | 0.947436 | -0.04867 | 0.01149 | 2.30E-05 | 0.97262 | 0.011166 |
| Mother's age at death | rs1265842 | 10:28924901_T_C | 0.482347 | -0.02164 | 0.005111 | 2.30E-05 | 0.98554 | 0.010354 |
| Mother's age at death | rs144882349 | 15:38878952_T_G | 0.986336 | -0.09396 | 0.022209 | 2.30E-05 | 0.97539 | 0.911066 |
| Mother's age at death | rs116278348 | 14:21038574_C_T | 0.982234 | -0.08389 | 0.019822 | 2.30E-05 | 0.94235 | 0.934777 |
| Mother's age at death | rs145760620 | 13:23258113_A_T | 0.948479 | -0.05048 | 0.011917 | 2.30E-05 | 0.93406 | 0.300961 |
| Mother's age at death | rs75095719 | 18:9372820_A_G | 0.899416 | 0.035889 | 0.008487 | 2.40E-05 | 0.99214 | 0.750136 |
| Mother's age at death | rs1267658 | 15:83214154_C_T | 0.382113 | -0.0221 | 0.005235 | 2.40E-05 | 0.99828 | 0.616567 |
| Mother's age at death | rs7702429 | 5:4293859_C_T | 0.846162 | -0.02985 | 0.007063 | 2.40E-05 | 0.98968 | 0.006065 |
| Mother's age at death | rs6032029 | 20:43786064_A_G | 0.801218 | 0.026926 | 0.006378 | 2.40E-05 | 0.99642 | 0.422241 |
| Mother's age at death | rs1011536 | 20:43793370_A_G | 0.801338 | 0.026979 | 0.00638 | 2.40E-05 | 0.99682 | 0.499505 |
| Mother's age at death | rs34412950 | 20:43805132_C_A | 0.811341 | 0.02769 | 0.006549 | 2.40E-05 | 0.98732 | 0.675787 |
| Mother's age at death | rs6032046 | 20:43823679_A_G | 0.808272 | 0.027359 | 0.006472 | 2.40E-05 | 0.99852 | 0.707229 |
| Mother's age at death | rs11476757 | 20:43854749_CA_C | 0.81393 | 0.027725 | 0.006565 | 2.40E-05 | 0.99429 | 0.855145 |
| Mother's age at death | rs138508186 | 20:43895058_GTTTA_G | 0.768829 | 0.025662 | 0.006077 | 2.40E-05 | 0.99052 | 0.340322 |
| Mother's age at death | rs11025542 | 11:20408926_C_T | 0.952654 | -0.05067 | 0.011996 | 2.40E-05 | 0.99709 | 0.974446 |
| Mother's age at death | rs3094662 | 6:31121945_A_C | 0.754511 | 0.024949 | 0.005904 | 2.40E-05 | 0.99997 | 0.315312 |
| Mother's age at death | rs3094005 | 6:31465047_G_T | 0.857809 | 0.030767 | 0.00728 | 2.40E-05 | 1 | 0.669607 |
| Mother's age at death | rs77085797 | 5:137776297_G_A | 0.891242 | 0.03479 | 0.008228 | 2.40E-05 | 0.99244 | 0.420407 |
| Mother's age at death | rs370696336 | 5:137470531_C_CAGCCTCCCGA | 0.642415 | 0.022874 | 0.005411 | 2.40E-05 | 0.96579 | 0.558882 |
| Mother's age at death | rs167456 | 8:23649551_G_A | 0.702224 | -0.02356 | 0.00558 | 2.40E-05 | 0.99643 | 0.912446 |
| Mother's age at death | rs576532423 | 8:65551653_GT_G | 0.989158 | -0.12714 | 0.030129 | 2.40E-05 | 0.65868 | 0.786339 |
| Mother's age at death | rs1449504 | 2:142808316_G_C | 0.483453 | -0.02157 | 0.005105 | 2.40E-05 | 0.99327 | 0.826361 |
| Mother's age at death | rs66595272 | 2:142814490_CTG_C | 0.483757 | -0.02158 | 0.005105 | 2.40E-05 | 0.99321 | 0.917242 |
| Mother's age at death | rs2218734 | 2:142829392_C_G | 0.483928 | -0.02152 | 0.005091 | 2.40E-05 | 0.99856 | 0.930999 |
| Mother's age at death | rs12467191 | 2:207641723_A_C | 0.914042 | 0.038381 | 0.009095 | 2.40E-05 | 0.99881 | 0.853914 |
| Mother's age at death | rs2359724 | 2:207615522_G_C | 0.917109 | 0.039363 | 0.00931 | 2.40E-05 | 0.98537 | 0.909143 |
| Mother's age at death | rs59577215 | 2:207619224_G_A | 0.915454 | 0.038789 | 0.009177 | 2.40E-05 | 0.99618 | 0.955309 |
| Mother's age at death | rs6752428 | 2:207622543_T_C | 0.91551 | 0.038787 | 0.009178 | 2.40E-05 | 0.99659 | 0.955287 |
| Mother's age at death | rs12465959 | 2:207623503_C_A | 0.928235 | 0.043619 | 0.010328 | 2.40E-05 | 0.91247 | 0.896501 |
| Mother's age at death | rs2071655 | 4:3118116_T_G | 0.578375 | -0.02188 | 0.00518 | 2.40E-05 | 0.99362 | 0.060716 |
| Mother's age at death | rs201785861 | 4:3135251_TAAAA_T | 0.696164 | -0.02341 | 0.005546 | 2.40E-05 | 0.99466 | 0.159669 |
| Mother's age at death | rs28403215 | 4:3172439_C_T | 0.577755 | -0.02188 | 0.005173 | 2.40E-05 | 0.996 | 0.086338 |
| Mother's age at death | rs1263348 | 4:2933271_G_A | 0.499503 | 0.021892 | 0.005181 | 2.40E-05 | 0.96737 | 0.241746 |
| Mother's age at death | rs363063 | 4:3141488_G_A | 0.584164 | -0.02187 | 0.005185 | 2.50E-05 | 0.99585 | 0.058939 |
| Mother's age at death | rs916171 | 4:3216815_C_G | 0.580387 | -0.0218 | 0.005168 | 2.50E-05 | 0.99853 | 0.12347 |
| Mother's age at death | rs362314 | 4:3235241_A_G | 0.580836 | -0.02181 | 0.005175 | 2.50E-05 | 0.99645 | 0.075422 |
| Mother's age at death | rs62135452 | 2:49000955_T_C | 0.846131 | 0.03054 | 0.00724 | 2.50E-05 | 0.94067 | 0.138976 |
| Mother's age at death | rs111944960 | 3:165137273_CA_C | 0.797802 | -0.03107 | 0.007366 | 2.50E-05 | 0.73766 | 0.514565 |
| Mother's age at death | rs10273635 | 7:127158357_T_G | 0.375133 | 0.022038 | 0.005234 | 2.50E-05 | 0.99759 | 0.006352 |
| Mother's age at death | rs113909141 | 2:207612273_TTGAG_T | 0.915392 | 0.038737 | 0.009186 | 2.50E-05 | 0.99344 | 0.910829 |
| Mother's age at death | rs55782969 | 2:207615013_T_C | 0.915471 | 0.038677 | 0.009175 | 2.50E-05 | 0.99665 | 0.955309 |
| Mother's age at death | rs55890679 | 2:207618921_A_G | 0.915925 | 0.038836 | 0.00921 | 2.50E-05 | 0.99408 | 0.865901 |
| Mother's age at death | rs12473950 | 2:207623347_A_G | 0.915538 | 0.038719 | 0.009179 | 2.50E-05 | 0.99666 | 0.925531 |
| Mother's age at death | rs12467467 | 2:207721132_T_C | 0.873242 | 0.032402 | 0.00768 | 2.50E-05 | 0.99396 | 0.744321 |
| Mother's age at death | rs3769162 | 2:174064270_T_G | 0.210466 | 0.026298 | 0.00624 | 2.50E-05 | 0.99673 | 0.370237 |
| Mother's age at death | rs3754739 | 2:174080179_A_G | 0.803009 | -0.02694 | 0.006391 | 2.50E-05 | 0.99802 | 0.341374 |
| Mother's age at death | rs11324880 | 2:142837667_TA_T | 0.48221 | -0.0215 | 0.005096 | 2.50E-05 | 0.99694 | 0.871569 |
| Mother's age at death | rs56031910 | 7:47709038_C_G | 0.963309 | -0.05683 | 0.013476 | 2.50E-05 | 1 | 0.111818 |
| Mother's age at death | rs5750401 | 22:37654932_G_C | 0.429611 | 0.02183 | 0.005175 | 2.50E-05 | 0.98034 | 0.026897 |
| Mother's age at death | rs11664460 | 18:9377468_T_C | 0.899347 | 0.035752 | 0.008476 | 2.50E-05 | 0.99442 | 0.667667 |
| Mother's age at death | rs242390 | 14:56646809_T_G | 0.880003 | -0.03301 | 0.00783 | 2.50E-05 | 0.99791 | 0.633082 |
| Mother's age at death | rs6420244 | 9:83145706_G_A | 0.209554 | -0.02645 | 0.006273 | 2.50E-05 | 0.99006 | 0.175776 |
| Mother's age at death | rs4604529 | 9:83151804_A_G | 0.204286 | -0.02674 | 0.006341 | 2.50E-05 | 0.98775 | 0.205774 |
| Mother's age at death | rs1328501 | 9:83168111_A_C | 0.305429 | -0.02333 | 0.005538 | 2.50E-05 | 0.99155 | 0.133656 |
| Mother's age at death | rs310310 | 8:23652578_C_A | 0.702714 | -0.02354 | 0.005583 | 2.50E-05 | 0.99676 | 0.852662 |
| Mother's age at death | rs71549821 | 8:23710548_A_AAAAG | 0.673272 | -0.02342 | 0.005551 | 2.50E-05 | 0.95473 | 0.578469 |
| Mother's age at death | rs1346146 | 8:81047278_C_T | 0.574646 | -0.0219 | 0.005194 | 2.50E-05 | 0.98751 | 0.82734 |
| Mother's age at death | rs11741862 | 5:4290895_G_A | 0.846087 | -0.02978 | 0.00706 | 2.50E-05 | 0.98982 | 0.004307 |
| Mother's age at death | rs7700616 | 5:4293378_G_A | 0.845991 | -0.02974 | 0.007058 | 2.50E-05 | 0.99015 | 0.006533 |
| Mother's age at death | rs2927960 | 8:2228382_C_A | 0.426812 | -0.02554 | 0.006065 | 2.50E-05 | 0.72855 | 0.002213 |
| Mother's age at death | rs3132510 | 6:31172151_T_C | 0.861981 | 0.031107 | 0.007387 | 2.50E-05 | 1 | 0.568748 |
| Mother's age at death | rs113397158 | 6:31394218_A_T | 0.978667 | -0.07412 | 0.0176 | 2.50E-05 | 0.99827 | 0.075059 |
| Mother's age at death | rs113428559 | 6:31408338_G_C | 0.978599 | -0.07396 | 0.017553 | 2.50E-05 | 0.99978 | 0.034467 |
| Mother's age at death | rs9265910 | 6:31313722_C_T | 0.853661 | 0.030367 | 0.007212 | 2.50E-05 | 0.99903 | 0.540916 |
| Mother's age at death | rs34508373 | 20:43788087_T_C | 0.80136 | 0.026919 | 0.006381 | 2.50E-05 | 0.99675 | 0.493662 |
| Mother's age at death | rs35525210 | 20:43813203_T_G | 0.808266 | 0.027303 | 0.006474 | 2.50E-05 | 0.998 | 0.756676 |
| Mother's age at death | rs7344269 | 20:43828668_T_C | 0.808659 | 0.027306 | 0.006475 | 2.50E-05 | 0.99887 | 0.679047 |
| Mother's age at death | rs7346579 | 20:43844722_A_G | 0.814089 | 0.02761 | 0.006555 | 2.50E-05 | 0.99785 | 0.847462 |
| Mother's age at death | rs6032094 | 20:43912637_G_A | 0.765523 | 0.02542 | 0.006028 | 2.50E-05 | 0.99753 | 0.415126 |
| Mother's age at death | rs142266702 | 20:12696902_TTGGACACAGAGGCAGA_T | 0.868444 | -0.03185 | 0.007564 | 2.50E-05 | 0.9952 | 0.763361 |
| Mother's age at death | rs35869085 | 20:43805134_G_C | 0.811514 | 0.027572 | 0.006553 | 2.60E-05 | 0.98698 | 0.668621 |
| Mother's age at death | rs147686536 | 20:43805251_ACTT_A | 0.809307 | 0.027337 | 0.006504 | 2.60E-05 | 0.9933 | 0.650834 |
| Mother's age at death | rs13041304 | 20:43811697_G_A | 0.808366 | 0.027255 | 0.006474 | 2.60E-05 | 0.9987 | 0.728182 |
| Mother's age at death | rs62208424 | 20:43813341_G_A | 0.808109 | 0.027194 | 0.006471 | 2.60E-05 | 0.99844 | 0.742592 |
| Mother's age at death | rs17424751 | 20:43814622_T_C | 0.808132 | 0.027216 | 0.006471 | 2.60E-05 | 0.99848 | 0.735495 |
| Mother's age at death | rs6032042 | 20:43815949_T_C | 0.808214 | 0.027245 | 0.006473 | 2.60E-05 | 0.99822 | 0.72835 |
| Mother's age at death | rs6032055 | 20:43833336_C_T | 0.809635 | 0.027318 | 0.006492 | 2.60E-05 | 0.99809 | 0.733973 |
| Mother's age at death | rs34891702 | 20:43869849_CT_C | 0.796095 | 0.027464 | 0.006523 | 2.60E-05 | 0.9382 | 0.648052 |
| Mother's age at death | rs6888652 | 5:4291656_G_A | 0.846007 | -0.0297 | 0.007058 | 2.60E-05 | 0.99025 | 0.006528 |
| Mother's age at death | rs6890011 | 5:4291689_C_T | 0.846132 | -0.02971 | 0.007063 | 2.60E-05 | 0.98953 | 0.006276 |
| Mother's age at death | rs6870793 | 5:4291933_A_T | 0.846008 | -0.0297 | 0.007058 | 2.60E-05 | 0.99025 | 0.006528 |
| Mother's age at death | rs6890880 | 5:4292211_C_T | 0.846142 | -0.02972 | 0.007061 | 2.60E-05 | 0.9899 | 0.005864 |
| Mother's age at death | rs11743733 | 5:4293029_G_A | 0.846008 | -0.02971 | 0.007058 | 2.60E-05 | 0.99025 | 0.006528 |
| Mother's age at death | rs11742174 | 5:4293345_T_G | 0.846008 | -0.02971 | 0.007058 | 2.60E-05 | 0.99025 | 0.006528 |
| Mother's age at death | rs11351043 | 5:4293765_AT_A | 0.846007 | -0.02971 | 0.007058 | 2.60E-05 | 0.99025 | 0.006528 |
| Mother's age at death | rs531139503 | 8:81016896_A_AT | 0.586558 | -0.02267 | 0.005386 | 2.60E-05 | 0.92095 | 0.266631 |
| Mother's age at death | rs2854018 | 6:31308562_C_T | 0.853761 | 0.030378 | 0.007216 | 2.60E-05 | 0.99816 | 0.603434 |
| Mother's age at death | rs2394980 | 6:31312941_C_T | 0.853637 | 0.030366 | 0.007212 | 2.60E-05 | 0.9989 | 0.533335 |
| Mother's age at death | rs9265886 | 6:31313221_A_C | 0.853698 | 0.030324 | 0.007212 | 2.60E-05 | 0.99931 | 0.533212 |
| Mother's age at death | rs3016017 | 6:31317063_A_G | 0.854797 | 0.030517 | 0.007256 | 2.60E-05 | 0.99267 | 0.762746 |
| Mother's age at death | rs2923007 | 6:31317065_T_C | 0.854797 | 0.030517 | 0.007256 | 2.60E-05 | 0.99267 | 0.762746 |
| Mother's age at death | rs2523495 | 6:31377978_C_T | 0.855411 | 0.030548 | 0.007256 | 2.60E-05 | 0.99244 | 0.43429 |
| Mother's age at death | rs58045889 | 6:31390637_A_G | 0.978578 | -0.07384 | 0.017545 | 2.60E-05 | 1 | 0.034602 |
| Mother's age at death | rs10061967 | 5:137467433_T_A | 0.627904 | 0.022234 | 0.005285 | 2.60E-05 | 0.99386 | 0.578242 |
| Mother's age at death | rs6446722 | 4:3111103_T_C | 0.576182 | -0.02174 | 0.005173 | 2.60E-05 | 0.9954 | 0.041515 |
| Mother's age at death | rs17780797 | 4:3117579_T_C | 0.578317 | -0.02179 | 0.005177 | 2.60E-05 | 0.99481 | 0.059924 |
| Mother's age at death | rs4690072 | 4:3122507_T_G | 0.577757 | -0.02179 | 0.005179 | 2.60E-05 | 0.99381 | 0.058412 |
| Mother's age at death | rs59613878 | 4:3139152_C_T | 0.695436 | -0.02329 | 0.005541 | 2.60E-05 | 0.99541 | 0.142771 |
| Mother's age at death | rs1868544 | 2:142796449_T_C | 0.482642 | -0.02147 | 0.005103 | 2.60E-05 | 0.99434 | 0.839858 |
| Mother's age at death | rs59093452 | 2:142836447_TA_T | 0.492357 | -0.0216 | 0.005134 | 2.60E-05 | 0.98237 | 0.623936 |
| Mother's age at death | rs5838042 | 2:207612641_TC_T | 0.91552 | 0.038559 | 0.009175 | 2.60E-05 | 0.99727 | 0.940405 |
| Mother's age at death | rs56012632 | 2:207613501_A_T | 0.91552 | 0.03856 | 0.009175 | 2.60E-05 | 0.99728 | 0.940405 |
| Mother's age at death | rs56943283 | 2:207613690_G_C | 0.91552 | 0.03856 | 0.009175 | 2.60E-05 | 0.99728 | 0.940405 |
| Mother's age at death | rs2359723 | 2:207615492_T_C | 0.917046 | 0.03917 | 0.009304 | 2.60E-05 | 0.98587 | 0.879134 |
| Mother's age at death | rs61258471 | 2:207615857_G_A | 0.915526 | 0.038571 | 0.009175 | 2.60E-05 | 0.99738 | 0.940399 |
| Mother's age at death | rs6752639 | 2:207616709_T_C | 0.915526 | 0.038572 | 0.009175 | 2.60E-05 | 0.99738 | 0.940399 |
| Mother's age at death | rs6737715 | 2:207616736_G_T | 0.915533 | 0.038575 | 0.009175 | 2.60E-05 | 0.99737 | 0.940394 |
| Mother's age at death | rs6737947 | 2:207616884_G_C | 0.915526 | 0.038571 | 0.009175 | 2.60E-05 | 0.99738 | 0.940399 |
| Mother's age at death | rs112674464 | 2:207617853_G_GATCA | 0.914411 | 0.038527 | 0.00916 | 2.60E-05 | 0.98876 | 0.824546 |
| Mother's age at death | rs59136983 | 2:207626130_G_A | 0.915837 | 0.038793 | 0.009225 | 2.60E-05 | 0.99093 | 0.865908 |
| Mother's age at death | rs2043950 | 2:174066730_C_A | 0.210504 | 0.026241 | 0.00624 | 2.60E-05 | 0.99672 | 0.384415 |
| Mother's age at death | rs7601536 | 2:174072949_G_A | 0.806128 | -0.02704 | 0.006432 | 2.60E-05 | 0.99789 | 0.258518 |
| Mother's age at death | rs7567355 | 2:174078135_C_G | 0.804605 | -0.02694 | 0.006411 | 2.60E-05 | 0.99791 | 0.277428 |
| Mother's age at death | rs17373294 | 1:60820075_T_C | 0.976531 | 0.070788 | 0.016843 | 2.60E-05 | 1 | 0.066558 |
| Mother's age at death | rs853480 | 1:21949537_A_G | 0.897997 | -0.03558 | 0.008452 | 2.60E-05 | 0.98683 | 0.252459 |
| Mother's age at death | . | 4:57809064_T_TCTTTC | 0.970611 | -0.08155 | 0.019402 | 2.60E-05 | 0.60564 | 0.80045 |
| Mother's age at death | rs10735548 | 9:83153980_C_G | 0.204397 | -0.02654 | 0.006312 | 2.60E-05 | 0.99657 | 0.27286 |
| Mother's age at death | rs10735549 | 9:83154213_T_C | 0.204392 | -0.02653 | 0.006311 | 2.60E-05 | 0.99672 | 0.250317 |
| Mother's age at death | rs7038004 | 9:83154502_A_T | 0.204253 | -0.02654 | 0.006311 | 2.60E-05 | 0.99732 | 0.272634 |
| Mother's age at death | rs1328500 | 9:83172180_T_G | 0.305254 | -0.02328 | 0.005541 | 2.60E-05 | 0.9909 | 0.138883 |
| Mother's age at death | rs181413527 | 16:30182405_A_G | 0.983504 | -0.08767 | 0.020831 | 2.60E-05 | 0.92141 | 0.860954 |
| Mother's age at death | rs141465770 | 16:30349649_C_T | 0.983688 | -0.08929 | 0.021226 | 2.60E-05 | 0.89812 | 0.790824 |
| Mother's age at death | rs139978 | 22:36856503_T_C | 0.905457 | 0.036882 | 0.008769 | 2.60E-05 | 0.9877 | 0.177566 |
| Mother's age at death | rs12002815 | 9:83160181_G_A | 0.206338 | -0.02644 | 0.006299 | 2.70E-05 | 0.99436 | 0.365704 |
| Mother's age at death | rs13332651 | 16:73446644_G_A | 0.57841 | 0.021871 | 0.005216 | 2.70E-05 | 0.97659 | 0.713773 |
| Mother's age at death | rs370883 | 5:137472454_G_C | 0.624717 | 0.022124 | 0.005276 | 2.70E-05 | 0.99487 | 0.475182 |
| Mother's age at death | rs4835669 | 5:137473391_G_A | 0.624907 | 0.022116 | 0.00527 | 2.70E-05 | 1 | 0.098924 |
| Mother's age at death | rs10068340 | 5:137479315_G_C | 0.631472 | 0.02227 | 0.005311 | 2.70E-05 | 0.98897 | 0.43456 |
| Mother's age at death | rs3130455 | 6:31125978_A_T | 0.753791 | 0.024736 | 0.005889 | 2.70E-05 | 1 | 0.025781 |
| Mother's age at death | rs2854019 | 6:31308476_T_A | 0.853641 | 0.030279 | 0.00721 | 2.70E-05 | 0.99937 | 0.579789 |
| Mother's age at death | rs2394979 | 6:31312729_G_A | 0.853618 | 0.030296 | 0.007213 | 2.70E-05 | 0.99865 | 0.533409 |
| Mother's age at death | rs7450305 | 6:31313367_T_C | 0.853551 | 0.030265 | 0.007213 | 2.70E-05 | 0.99831 | 0.533544 |
| Mother's age at death | rs9265937 | 6:31314938_C_T | 0.853761 | 0.03025 | 0.007211 | 2.70E-05 | 0.99972 | 0.611533 |
| Mother's age at death | rs9265990 | 6:31316448_T_A | 0.854813 | 0.030368 | 0.00723 | 2.70E-05 | 0.99804 | 0.798314 |
| Mother's age at death | rs36057735 | 6:31319923_C_G | 0.800082 | 0.026856 | 0.006404 | 2.70E-05 | 0.97584 | 0.000119 |
| Mother's age at death | rs8365 | 6:32148403_G_C | 0.799614 | 0.026652 | 0.006349 | 2.70E-05 | 0.99917 | 0.558149 |
| Mother's age at death | rs4907356 | 8:142584729_C_T | 0.180473 | 0.027827 | 0.006629 | 2.70E-05 | 0.9921 | 0.323735 |
| Mother's age at death | rs6017503 | 20:43815103_G_A | 0.808202 | 0.02719 | 0.006472 | 2.70E-05 | 0.99841 | 0.721323 |
| Mother's age at death | rs6032095 | 20:43913255_A_T | 0.766312 | 0.025374 | 0.006044 | 2.70E-05 | 0.99489 | 0.391309 |
| Mother's age at death | rs11699066 | 20:12693957_T_A | 0.868649 | -0.03176 | 0.007569 | 2.70E-05 | 0.99524 | 0.772663 |
| Mother's age at death | rs192843 | 8:23687170_G_A | 0.703164 | -0.02354 | 0.005602 | 2.70E-05 | 0.99097 | 0.684558 |
| Mother's age at death | rs17718919 | 8:117235249_T_C | 0.989732 | -0.12033 | 0.028665 | 2.70E-05 | 0.75915 | 0.000201 |
| Mother's age at death | rs12092297 | 1:83469974_A_G | 0.821132 | -0.02833 | 0.006751 | 2.70E-05 | 0.96452 | 0.722121 |
| Mother's age at death | rs556703887 | 1:83481511_A_AT | 0.825925 | -0.02834 | 0.006756 | 2.70E-05 | 0.9838 | 0.642599 |
| Mother's age at death | rs7556518 | 1:7847846_G_A | 0.841518 | -0.02944 | 0.007009 | 2.70E-05 | 0.98953 | 0.770531 |
| Mother's age at death | rs143164160 | 4:3139792_GTTCC_G | 0.583078 | -0.02174 | 0.005181 | 2.70E-05 | 0.99612 | 0.050176 |
| Mother's age at death | rs4690010 | 4:3159719_G_T | 0.5826 | -0.02184 | 0.005201 | 2.70E-05 | 0.98765 | 0.088634 |
| Mother's age at death | rs363092 | 4:3196029_A_C | 0.41982 | 0.021724 | 0.005171 | 2.70E-05 | 0.99732 | 0.129399 |
| Mother's age at death | rs762847 | 4:2912063_A_G | 0.503528 | -0.02137 | 0.005088 | 2.70E-05 | 1 | 0.813085 |
| Mother's age at death | rs13036224 | 2:142804790_G_T | 0.483602 | -0.02143 | 0.00511 | 2.70E-05 | 0.99172 | 0.799494 |
| Mother's age at death | rs11692364 | 2:142809287_A_G | 0.48327 | -0.02146 | 0.005108 | 2.70E-05 | 0.99234 | 0.835361 |
| Mother's age at death | rs4662372 | 2:142841917_C_T | 0.48367 | -0.02138 | 0.005089 | 2.70E-05 | 0.9992 | 0.908084 |
| Mother's age at death | rs10496914 | 2:142881540_A_G | 0.492091 | -0.02156 | 0.005137 | 2.70E-05 | 0.98019 | 0.912734 |
| Mother's age at death | rs6435347 | 2:207612879_T_C | 0.915576 | 0.03856 | 0.009182 | 2.70E-05 | 0.99609 | 0.955241 |
| Mother's age at death | rs6435348 | 2:207612972_A_G | 0.915799 | 0.038603 | 0.0092 | 2.70E-05 | 0.99493 | 0.89559 |
| Mother's age at death | rs73068052 | 2:207615119_T_A | 0.915368 | 0.038488 | 0.009174 | 2.70E-05 | 0.99581 | 0.95536 |
| Mother's age at death | rs7583037 | 2:207612433_T_A | 0.915906 | 0.038615 | 0.009214 | 2.80E-05 | 0.99298 | 0.970047 |
| Mother's age at death | rs73974105 | 2:174073800_G_T | 0.805847 | -0.02693 | 0.006429 | 2.80E-05 | 0.99768 | 0.270951 |
| Mother's age at death | rs1449503 | 2:142807959_A_C | 0.483609 | -0.02137 | 0.005105 | 2.80E-05 | 0.99357 | 0.808427 |
| Mother's age at death | rs1449481 | 2:142835889_C_T | 0.483912 | -0.02132 | 0.005089 | 2.80E-05 | 1 | 0.737768 |
| Mother's age at death | rs362273 | 4:3227419_A_G | 0.683269 | -0.02294 | 0.005481 | 2.80E-05 | 0.99674 | 0.065615 |
| Mother's age at death | rs6702394 | 1:168927684_C_T | 0.648278 | 0.022326 | 0.005332 | 2.80E-05 | 0.99657 | 0.667252 |
| Mother's age at death | rs74060012 | 1:21919880_A_T | 0.857799 | -0.03083 | 0.007359 | 2.80E-05 | 0.97675 | 0.390113 |
| Mother's age at death | rs968212 | 9:83136572_A_G | 0.208942 | -0.0263 | 0.006283 | 2.80E-05 | 0.9888 | 0.161339 |
| Mother's age at death | rs7505307 | 18:14454917_C_T | 0.82667 | -0.02966 | 0.007078 | 2.80E-05 | 0.90525 | 0.450703 |
| Mother's age at death | rs3130349 | 6:32147696_G_A | 0.799482 | 0.026603 | 0.006345 | 2.80E-05 | 1 | 0.589034 |
| Mother's age at death | rs3134941 | 6:32149537_C_G | 0.799449 | 0.026557 | 0.006345 | 2.80E-05 | 0.99971 | 0.570532 |
| Mother's age at death | rs3134940 | 6:32149816_T_C | 0.799477 | 0.026593 | 0.006345 | 2.80E-05 | 0.99988 | 0.570519 |
| Mother's age at death | rs1800625 | 6:32152442_A_G | 0.799347 | 0.026605 | 0.006357 | 2.80E-05 | 0.99516 | 0.54048 |
| Mother's age at death | rs2256750 | 6:31312020_G_A | 0.853816 | 0.030181 | 0.007211 | 2.80E-05 | 0.99998 | 0.571571 |
| Mother's age at death | rs2394981 | 6:31313029_G_T | 0.851783 | 0.030122 | 0.007188 | 2.80E-05 | 0.99456 | 0.515217 |
| Mother's age at death | rs2507998 | 6:31314652_A_G | 0.853806 | 0.030193 | 0.007211 | 2.80E-05 | 0.99987 | 0.587368 |
| Mother's age at death | rs9265979 | 6:31316044_C_T | 0.853912 | 0.030255 | 0.007216 | 2.80E-05 | 0.99909 | 0.619341 |
| Mother's age at death | rs2523992 | 6:30075103_T_G | 0.819542 | 0.02788 | 0.00665 | 2.80E-05 | 0.98616 | 0.26788 |
| Mother's age at death | rs3095156 | 6:30926517_C_T | 0.848076 | 0.029767 | 0.007099 | 2.80E-05 | 0.99924 | 0.875441 |
| Mother's age at death | rs217276 | 5:137459469_T_C | 0.626481 | 0.022248 | 0.005308 | 2.80E-05 | 0.9778 | 0.152528 |
| Mother's age at death | rs217277 | 5:137460383_T_A | 0.626498 | 0.022234 | 0.005308 | 2.80E-05 | 0.97779 | 0.149022 |
| Mother's age at death | . | 20:43830737_A_ATTCT | 0.806082 | 0.027056 | 0.006452 | 2.80E-05 | 0.66639 | 0.691576 |
| Mother's age at death | . | 20:43830737_ATTCT_A | 0.806082 | 0.027056 | 0.006452 | 2.80E-05 | 0.9968 | 0.859603 |
| Mother's age at death | rs2741523 | 20:43920446_C_T | 0.765214 | 0.025272 | 0.006029 | 2.80E-05 | 0.99699 | 0.388419 |
| Mother's age at death | rs6032093 | 20:43912167_A_G | 0.765621 | 0.0252 | 0.00603 | 2.90E-05 | 0.99726 | 0.410412 |
| Mother's age at death | rs6883842 | 5:4291145_G_A | 0.845766 | -0.02955 | 0.007064 | 2.90E-05 | 0.98706 | 0.003787 |
| Mother's age at death | rs435570 | 5:137468635_T_G | 0.626746 | 0.022099 | 0.005289 | 2.90E-05 | 0.99118 | 0.600012 |
| Mother's age at death | rs402175 | 6:31308717_G_A | 0.853718 | 0.030173 | 0.007211 | 2.90E-05 | 0.99923 | 0.644392 |
| Mother's age at death | rs9265831 | 6:31311449_T_A | 0.850943 | 0.030023 | 0.007179 | 2.90E-05 | 0.99135 | 0.690892 |
| Mother's age at death | rs2256747 | 6:31311950_T_C | 0.853821 | 0.030147 | 0.007213 | 2.90E-05 | 0.99957 | 0.571505 |
| Mother's age at death | rs2844589 | 6:31312259_G_A | 0.853815 | 0.030177 | 0.007211 | 2.90E-05 | 0.99998 | 0.587368 |
| Mother's age at death | rs9265857 | 6:31312607_G_C | 0.853812 | 0.030178 | 0.007211 | 2.90E-05 | 0.99992 | 0.571571 |
| Mother's age at death | rs2394978 | 6:31312656_C_T | 0.853813 | 0.030178 | 0.007211 | 2.90E-05 | 0.99992 | 0.571571 |
| Mother's age at death | rs9265971 | 6:31315792_T_C | 0.853853 | 0.030154 | 0.007213 | 2.90E-05 | 0.99964 | 0.611364 |
| Mother's age at death | rs9265972 | 6:31315805_G_C | 0.853853 | 0.030153 | 0.007213 | 2.90E-05 | 0.99965 | 0.611364 |
| Top 1% of age-at-death distribution | rs528161076 | 7:4827514_T_TC | 0.976472 | -0.01577 | 0.002857 | 3.40E-08 | 0.97739 | 0.559345 |
| Top 1% of age-at-death distribution | rs75824829 | 9:138268602_G_A | 0.962546 | -0.01326 | 0.002415 | 4.00E-08 | 0.86746 | 0.118016 |
| Top 1% of age-at-death distribution | rs79109928 | 7:4826191_G_A | 0.977264 | -0.01545 | 0.002906 | 1.00E-07 | 0.97375 | 1 |
| Top 1% of age-at-death distribution | rs17135119 | 7:4822576_C_T | 0.976231 | -0.01468 | 0.002813 | 1.80E-07 | 0.99507 | 0.897255 |
| Top 1% of age-at-death distribution | rs116402929 | 7:4825003_T_C | 0.976412 | -0.0147 | 0.002819 | 1.80E-07 | 0.99776 | 0.948392 |
| Top 1% of age-at-death distribution | rs377310910 | 9:1940937_G_A | 0.969251 | -0.01479 | 0.002862 | 2.30E-07 | 0.74126 | 0.76454 |
| Top 1% of age-at-death distribution | rs112711895 | 7:4826649_T_C | 0.976249 | -0.01462 | 0.002829 | 2.40E-07 | 0.97427 | 0.892732 |
| Top 1% of age-at-death distribution | rs79959107 | 7:4838301_G_A | 0.976498 | -0.01451 | 0.002824 | 2.80E-07 | 0.99777 | 0.948301 |
| Top 1% of age-at-death distribution | rs112648533 | 7:4834304_G_A | 0.976375 | -0.01437 | 0.002814 | 3.30E-07 | 0.99687 | 1 |
| Top 1% of age-at-death distribution | rs80198324 | 7:4834555_G_A | 0.976327 | -0.01428 | 0.00281 | 3.70E-07 | 0.99762 | 1 |
| Top 1% of age-at-death distribution | rs148783056 | 7:4834969_T_C | 0.976327 | -0.01428 | 0.00281 | 3.70E-07 | 0.99762 | 1 |
| Top 1% of age-at-death distribution | rs113054066 | 7:4835223_C_G | 0.976682 | -0.01443 | 0.002839 | 3.70E-07 | 0.99194 | 1 |
| Top 1% of age-at-death distribution | rs116576980 | 7:4824131_A_G | 0.976434 | -0.01427 | 0.00282 | 4.20E-07 | 0.99756 | 0.948338 |
| Top 1% of age-at-death distribution | rs7861963 | 9:128178907_T_C | 0.982787 | -0.0164 | 0.003247 | 4.40E-07 | 0.99586 | 0.924175 |
| Top 1% of age-at-death distribution | rs10261754 | 7:4841188_T_C | 0.975876 | -0.01404 | 0.002789 | 4.80E-07 | 0.99678 | 0.652289 |
| Top 1% of age-at-death distribution | rs115198050 | 7:4831777_G_A | 0.975837 | -0.01396 | 0.002779 | 5.00E-07 | 0.99683 | 0.897255 |
| Top 1% of age-at-death distribution | rs144790747 | 19:14487347_G_A | 0.983941 | -0.02058 | 0.004099 | 5.10E-07 | 0.69065 | 0.928498 |
| Top 1% of age-at-death distribution | rs7871262 | 9:128177681_A_G | 0.982755 | -0.01627 | 0.003241 | 5.20E-07 | 0.99638 | 0.924255 |
| Top 1% of age-at-death distribution | rs7858017 | 9:128178007_T_C | 0.982752 | -0.01625 | 0.003241 | 5.30E-07 | 0.9962 | 0.924255 |
| Top 1% of age-at-death distribution | rs1976355 | 9:128175847_C_A | 0.982879 | -0.01641 | 0.003274 | 5.40E-07 | 0.97925 | 1 |
| Top 1% of age-at-death distribution | rs79520630 | 7:4838181_C_G | 0.976367 | -0.01411 | 0.002814 | 5.40E-07 | 0.99834 | 0.948301 |
| Top 1% of age-at-death distribution | rs60589809 | 9:92937726_C_G | 0.955652 | -0.01051 | 0.002103 | 5.80E-07 | 0.96793 | 0.39819 |
| Top 1% of age-at-death distribution | rs7784925 | 7:4833109_A_G | 0.97657 | -0.01418 | 0.002842 | 6.00E-07 | 0.98365 | 0.947153 |
| Top 1% of age-at-death distribution | rs146678646 | 7:4835534_TTTTC_T | 0.97298 | -0.01345 | 0.002708 | 6.70E-07 | 0.94356 | 0.651328 |
| Top 1% of age-at-death distribution | rs2804497 | 10:33616037_G_T | 0.986628 | -0.02042 | 0.004107 | 6.70E-07 | 0.82085 | 4.58E-05 |
| Top 1% of age-at-death distribution | rs139762862 | 7:4837486_G_A | 0.976437 | -0.01403 | 0.002827 | 6.90E-07 | 0.9933 | 0.517738 |
| Top 1% of age-at-death distribution | rs78710395 | 9:92906479_T_A | 0.971091 | -0.01278 | 0.002577 | 7.10E-07 | 0.96744 | 0.346282 |
| Top 1% of age-at-death distribution | 1:106735127_GCTGCTTTTTTA_G | 1:106735127_GCTGCTTTTTT_G | 0.984128 | -0.01715 | 0.003462 | 7.30E-07 | 0.99072 | 0.714529 |
| Top 1% of age-at-death distribution | 1:106735127_GCTGCTTTTTTA_G | 1:106735127_GCTGCTTTTTTA_G | 0.984128 | -0.01715 | 0.003462 | 7.30E-07 | 0.97293 | 0.778449 |
| Top 1% of age-at-death distribution | rs11999166 | 9:128176529_C_T | 0.983013 | -0.01621 | 0.003288 | 8.20E-07 | 0.97891 | 0.771809 |
| Top 1% of age-at-death distribution | rs12002063 | 9:128175143_A_G | 0.982772 | -0.01598 | 0.003243 | 8.30E-07 | 0.99579 | 0.924311 |
| Top 1% of age-at-death distribution | 1:9361593_TGGATAGACCTGAGTGCGCACAGGACAC_T | 1:9361593_TGGATAGACCTGAGTGCGCACAGGACAC_T | 0.989052 | -0.02399 | 0.004873 | 8.50E-07 | 0.69846 | 0.37086 |
| Top 1% of age-at-death distribution | rs1443844 | 6:160574802_A_G | 0.606429 | -0.00429 | 0.000872 | 8.90E-07 | 0.99877 | 0.645431 |
| Top 1% of age-at-death distribution | rs55863212 | 9:128180163_A_G | 0.9827 | -0.01587 | 0.003231 | 9.10E-07 | 0.99521 | 0.776784 |
| Top 1% of age-at-death distribution | rs143308878 | 7:4872154_A_G | 0.977681 | -0.0144 | 0.002932 | 9.10E-07 | 0.97619 | 1 |
| Top 1% of age-at-death distribution | rs13070407 | 3:25493178_T_C | 0.729334 | 0.004701 | 0.000959 | 9.60E-07 | 1 | 0.309165 |
| Top 1% of age-at-death distribution | rs1450818 | 1:106697841_A_G | 0.981656 | -0.01554 | 0.003181 | 1.00E-06 | 0.99902 | 0.806147 |
| Top 1% of age-at-death distribution | rs3798164 | 6:160575202_G_A | 0.608855 | -0.00426 | 0.000873 | 1.00E-06 | 1 | 0.733973 |
| Top 1% of age-at-death distribution | rs12002064 | 9:128175153_A_G | 0.982706 | -0.01585 | 0.003241 | 1.00E-06 | 0.99319 | 0.924739 |
| Top 1% of age-at-death distribution | rs2173484 | 9:128173526_C_A | 0.983016 | -0.01601 | 0.003281 | 1.10E-06 | 0.99118 | 0.9242 |
| Top 1% of age-at-death distribution | rs4709402 | 6:160574037_C_T | 0.613169 | -0.00428 | 0.000877 | 1.10E-06 | 0.99381 | 0.798281 |
| Top 1% of age-at-death distribution | 1:106735127_GCTGCTTTTTT_G | 1:106735127_GCTGCTTTTTT_G | 0.983718 | -0.01653 | 0.003389 | 1.10E-06 | 0.99072 | 0.714529 |
| Top 1% of age-at-death distribution | 1:106735127_GCTGCTTTTTT_G | 1:106735127_GCTGCTTTTTTA_G | 0.983718 | -0.01653 | 0.003389 | 1.10E-06 | 0.97293 | 0.778449 |
| Top 1% of age-at-death distribution | rs1597543 | 1:106735203_C_T | 0.983718 | -0.01653 | 0.003389 | 1.10E-06 | 0.99072 | 0.714529 |
| Top 1% of age-at-death distribution | rs112859713 | 1:106732820_G_A | 0.981839 | -0.01569 | 0.003229 | 1.20E-06 | 0.9787 | 1 |
| Top 1% of age-at-death distribution | rs200433732 | 1:106735139_AG_A | 0.983689 | -0.01644 | 0.003387 | 1.20E-06 | 0.99057 | 0.714611 |
| Top 1% of age-at-death distribution | rs4709401 | 6:160574027_C_T | 0.610869 | -0.00425 | 0.000875 | 1.20E-06 | 0.99422 | 0.860013 |
| Top 1% of age-at-death distribution | rs145558872 | 7:4835595_A_G | 0.974908 | -0.01345 | 0.002767 | 1.20E-06 | 0.97288 | 0.951544 |
| Top 1% of age-at-death distribution | rs11216843 | 11:118151329_A_T | 0.972766 | -0.0128 | 0.002637 | 1.20E-06 | 0.99463 | 0.519063 |
| Top 1% of age-at-death distribution | rs59169624 | 11:19302569_C_T | 0.805016 | -0.00535 | 0.001105 | 1.30E-06 | 0.94695 | 0.211091 |
| Top 1% of age-at-death distribution | rs117973291 | 9:92909107_G_C | 0.970336 | -0.01223 | 0.002528 | 1.30E-06 | 0.98226 | 0.358311 |
| Top 1% of age-at-death distribution | rs79391793 | 9:92909136_T_A | 0.970248 | -0.01221 | 0.002522 | 1.30E-06 | 0.98411 | 0.360255 |
| Top 1% of age-at-death distribution | 6:119996196_G_A | 6:119996196_G_A | 0.827052 | -0.00649 | 0.001341 | 1.30E-06 | 0.71248 | 0.894835 |
| Top 1% of age-at-death distribution | 6:119996196_G_A | 6:119996196_GTACACA_G | 0.827052 | -0.00649 | 0.001341 | 1.30E-06 | 0.63321 | 1 |
| Top 1% of age-at-death distribution | rs10114429 | 9:92905337_A_G | 0.970211 | -0.01216 | 0.00252 | 1.40E-06 | 0.98441 | 0.360753 |
| Top 1% of age-at-death distribution | rs12337287 | 9:92906025_A_C | 0.970213 | -0.01217 | 0.00252 | 1.40E-06 | 0.98441 | 0.360753 |
| Top 1% of age-at-death distribution | rs7030204 | 9:92907539_A_G | 0.970213 | -0.01217 | 0.00252 | 1.40E-06 | 0.98441 | 0.360753 |
| Top 1% of age-at-death distribution | rs10116951 | 9:92908392_A_T | 0.970216 | -0.01217 | 0.00252 | 1.40E-06 | 0.98458 | 0.360753 |
| Top 1% of age-at-death distribution | rs77604360 | 9:92909369_C_T | 0.970215 | -0.01217 | 0.00252 | 1.40E-06 | 0.98453 | 0.360844 |
| Top 1% of age-at-death distribution | rs564698311 | 9:131541741_GA_G | 0.021562 | 0.018217 | 0.003773 | 1.40E-06 | 0.53206 | 0.587787 |
| Top 1% of age-at-death distribution | rs12420856 | 11:35532311_C_T | 0.941284 | -0.00876 | 0.001815 | 1.40E-06 | 1 | 0.159518 |
| Top 1% of age-at-death distribution | rs75672964 | 7:131321010_C_T | 0.958252 | -0.01042 | 0.002159 | 1.40E-06 | 0.97308 | 0.854248 |
| Top 1% of age-at-death distribution | rs12047415 | 1:106710944_C_G | 0.982049 | -0.01549 | 0.003218 | 1.50E-06 | 0.99846 | 0.742557 |
| Top 1% of age-at-death distribution | rs113059985 | 1:106719854_T_C | 0.981479 | -0.01528 | 0.003175 | 1.50E-06 | 0.99366 | 0.935276 |
| Top 1% of age-at-death distribution | rs6550980 | 3:25491214_G_C | 0.260041 | -0.0047 | 0.000977 | 1.50E-06 | 0.98763 | 0.325057 |
| Top 1% of age-at-death distribution | rs80124926 | 6:92881402_GA_G | 0.703024 | 0.004533 | 0.000942 | 1.50E-06 | 0.98007 | 0.771951 |
| Top 1% of age-at-death distribution | rs78539969 | 15:39347046_A_G | 0.979617 | -0.01453 | 0.003031 | 1.60E-06 | 0.98183 | 0.93743 |
| Top 1% of age-at-death distribution | rs117991161 | 7:4849281_C_T | 0.980551 | -0.01559 | 0.003248 | 1.60E-06 | 0.91186 | 0.700922 |
| Top 1% of age-at-death distribution | rs17016851 | 1:106698906_C_T | 0.981128 | -0.01504 | 0.003137 | 1.60E-06 | 1 | 0.810588 |
| Top 1% of age-at-death distribution | rs117260820 | 10:102048643_G_T | 0.973703 | -0.01279 | 0.002672 | 1.70E-06 | 1 | 0.533053 |
| Top 1% of age-at-death distribution | rs9295122 | 6:160570013_C_T | 0.606432 | -0.00419 | 0.000874 | 1.70E-06 | 0.9944 | 0.702797 |
| Top 1% of age-at-death distribution | rs9295124 | 6:160570172_A_G | 0.606224 | -0.00418 | 0.000873 | 1.70E-06 | 0.99557 | 0.720892 |
| Top 1% of age-at-death distribution | rs3818678 | 6:160577499_G_C | 0.606282 | -0.00418 | 0.000873 | 1.70E-06 | 0.99609 | 0.615234 |
| Top 1% of age-at-death distribution | rs9295125 | 6:160578271_G_T | 0.611263 | -0.0042 | 0.000877 | 1.70E-06 | 0.99221 | 0.737989 |
| Top 1% of age-at-death distribution | rs72261809 | 10:133021836_TAAAAC_T | 0.988884 | -0.01972 | 0.004133 | 1.80E-06 | 0.96683 | 0.767786 |
| Top 1% of age-at-death distribution | rs2232063 | 19:10196350_G_A | 0.976113 | -0.01336 | 0.002804 | 1.90E-06 | 1 | 0.016181 |
| Top 1% of age-at-death distribution | rs1393698 | 11:19302570_A_G | 0.80383 | -0.00526 | 0.001102 | 1.90E-06 | 0.94648 | 0.222449 |
| Top 1% of age-at-death distribution | rs1349387 | 1:106699400_T_C | 0.981713 | -0.01515 | 0.003184 | 1.90E-06 | 1 | 0.805738 |
| Top 1% of age-at-death distribution | rs11184776 | 1:106704845_G_T | 0.981718 | -0.01517 | 0.003185 | 1.90E-06 | 0.99972 | 0.805868 |
| Top 1% of age-at-death distribution | rs12038435 | 1:106709489_G_A | 0.981709 | -0.01516 | 0.003185 | 1.90E-06 | 0.99935 | 0.806004 |
| Top 1% of age-at-death distribution | rs12036370 | 1:106710723_A_G | 0.981718 | -0.01517 | 0.003185 | 1.90E-06 | 0.99972 | 0.805868 |
| Top 1% of age-at-death distribution | rs12037138 | 1:106711067_A_C | 0.981718 | -0.01517 | 0.003185 | 1.90E-06 | 0.99972 | 0.805868 |
| Top 1% of age-at-death distribution | rs139408385 | 1:106714626_A_T | 0.981718 | -0.01517 | 0.003185 | 1.90E-06 | 0.99972 | 0.805868 |
| Top 1% of age-at-death distribution | rs72493327 | 2:5270432_T_C | 0.957162 | 0.010016 | 0.002102 | 1.90E-06 | 1 | 0.689001 |
| Top 1% of age-at-death distribution | rs77654063 | 7:4854281_G_A | 0.976441 | -0.01351 | 0.002836 | 1.90E-06 | 0.98729 | 0.794502 |
| Top 1% of age-at-death distribution | rs74450826 | 7:4854774_G_A | 0.976564 | -0.01349 | 0.002832 | 1.90E-06 | 0.99449 | 0.896322 |
| Top 1% of age-at-death distribution | rs139086474 | 9:92902494_A_AT | 0.971599 | -0.01231 | 0.00259 | 2.00E-06 | 0.9792 | 0.345287 |
| Top 1% of age-at-death distribution | rs67278738 | 6:92880929_A_G | 0.703454 | 0.004474 | 0.000941 | 2.00E-06 | 0.98166 | 0.740406 |
| Top 1% of age-at-death distribution | rs199710891 | 12:14273071_A_AAT | 0.984469 | -0.01794 | 0.003776 | 2.00E-06 | 0.83926 | 1 |
| Top 1% of age-at-death distribution | rs77601757 | 1:106723958_G_A | 0.981718 | -0.01515 | 0.003189 | 2.00E-06 | 0.99744 | 0.805935 |
| Top 1% of age-at-death distribution | rs2297374 | 6:160575985_C_T | 0.62572 | -0.00419 | 0.000883 | 2.10E-06 | 0.99405 | 0.634293 |
| Top 1% of age-at-death distribution | rs9347388 | 6:160572557_T_A | 0.624015 | -0.00417 | 0.000882 | 2.20E-06 | 0.9951 | 0.399696 |
| Top 1% of age-at-death distribution | rs10152803 | 15:39346077_A_G | 0.979772 | -0.01429 | 0.003016 | 2.20E-06 | 1 | 0.812019 |
| Top 1% of age-at-death distribution | rs6949537 | 7:4853933_C_T | 0.975974 | -0.0132 | 0.002796 | 2.30E-06 | 0.99419 | 0.846742 |
| Top 1% of age-at-death distribution | rs138218076 | 9:92911989_G_A | 0.96981 | -0.01191 | 0.002522 | 2.40E-06 | 0.97042 | 0.506058 |
| Top 1% of age-at-death distribution | rs143696562 | 9:92912024_G_A | 0.96981 | -0.01191 | 0.002522 | 2.40E-06 | 0.97042 | 0.506058 |
| Top 1% of age-at-death distribution | rs144431416 | 4:71669617_AAT_A | 0.983396 | -0.01657 | 0.003516 | 2.40E-06 | 0.8771 | 0.339938 |
| Top 1% of age-at-death distribution | rs11081417 | 18:897091_A_G | 0.838272 | -0.00557 | 0.00118 | 2.40E-06 | 0.95719 | 0.197179 |
| Top 1% of age-at-death distribution | rs809367 | 10:89741806_G_A | 0.905519 | -0.00687 | 0.001458 | 2.50E-06 | 0.99788 | 0.298875 |
| Top 1% of age-at-death distribution | rs7750592 | 6:160569782_T_C | 0.606583 | -0.00412 | 0.000874 | 2.50E-06 | 0.99455 | 0.725437 |
| Top 1% of age-at-death distribution | rs75346371 | 9:92921222_G_A | 0.969768 | -0.01186 | 0.002524 | 2.60E-06 | 0.96756 | 0.443261 |
| Top 1% of age-at-death distribution | rs113290458 | 9:128456000_G_A | 0.96759 | -0.01353 | 0.00288 | 2.60E-06 | 0.65791 | 0.647023 |
| Top 1% of age-at-death distribution | rs34968724 | 7:7724945_C_CT | 0.373393 | 0.004204 | 0.000896 | 2.70E-06 | 0.96921 | 0.800304 |
| Top 1% of age-at-death distribution | rs13212914 | 6:160570624_C_T | 0.624666 | -0.00413 | 0.000882 | 2.80E-06 | 0.99444 | 0.382464 |
| Top 1% of age-at-death distribution | rs12030622 | 1:106693681_T_A | 0.981926 | -0.01525 | 0.003256 | 2.80E-06 | 0.96737 | 0.802862 |
| Top 1% of age-at-death distribution | rs113690667 | 11:107659453_C_T | 0.953871 | -0.00977 | 0.002089 | 2.90E-06 | 0.94982 | 0.973742 |
| Top 1% of age-at-death distribution | rs200274287 | 10:101964476_GAAC_G | 0.974834 | -0.01294 | 0.002772 | 3.00E-06 | 0.96745 | 0.859819 |
| Top 1% of age-at-death distribution | rs9295123 | 6:160570114_G_A | 0.608281 | -0.00408 | 0.000875 | 3.00E-06 | 0.9945 | 0.671228 |
| Top 1% of age-at-death distribution | rs6932881 | 6:92874922_G_T | 0.698572 | 0.004372 | 0.000937 | 3.00E-06 | 0.98439 | 0.929174 |
| Top 1% of age-at-death distribution | rs199758578 | 9:92917828_G_A | 0.969665 | -0.01172 | 0.002514 | 3.10E-06 | 0.97239 | 0.476245 |
| Top 1% of age-at-death distribution | rs142803024 | 9:92917924_A_C | 0.969665 | -0.01172 | 0.002514 | 3.10E-06 | 0.97239 | 0.476245 |
| Top 1% of age-at-death distribution | rs58580824 | 9:92919191_A_T | 0.969649 | -0.01172 | 0.002513 | 3.10E-06 | 0.97202 | 0.507867 |
| Top 1% of age-at-death distribution | rs148378832 | 7:131309859_C_T | 0.951031 | -0.0098 | 0.002102 | 3.10E-06 | 0.88183 | 0.822419 |
| Top 1% of age-at-death distribution | rs114792874 | 7:131310080_A_G | 0.951028 | -0.0098 | 0.002102 | 3.10E-06 | 0.88257 | 0.822447 |
| Top 1% of age-at-death distribution | rs60685937 | 9:92919207_G_A | 0.969645 | -0.01171 | 0.002513 | 3.20E-06 | 0.97208 | 0.507925 |
| Top 1% of age-at-death distribution | rs74305899 | 9:92921665_A_G | 0.969619 | -0.0117 | 0.002513 | 3.20E-06 | 0.9714 | 0.445297 |
| Top 1% of age-at-death distribution | rs2346160 | 6:167675304_G_T | 0.368632 | 0.004203 | 0.000902 | 3.20E-06 | 0.96383 | 0.347256 |
| Top 1% of age-at-death distribution | rs73307309 | 7:4838002_A_C | 0.975184 | -0.01273 | 0.002736 | 3.20E-06 | 0.99707 | 1 |
| Top 1% of age-at-death distribution | rs77126626 | 9:92923212_A_T | 0.969607 | -0.0117 | 0.002517 | 3.30E-06 | 0.96839 | 0.445297 |
| Top 1% of age-at-death distribution | rs9347386 | 6:160571499_A_G | 0.623761 | -0.0041 | 0.000881 | 3.30E-06 | 0.99536 | 0.379463 |
| Top 1% of age-at-death distribution | rs11216846 | 11:118163916_G_A | 0.972748 | -0.01226 | 0.002638 | 3.40E-06 | 0.99347 | 0.555469 |
| Top 1% of age-at-death distribution | rs72698044 | 1:165627540_A_G | 0.92853 | -0.0077 | 0.001663 | 3.60E-06 | 0.99229 | 0.789205 |
| Top 1% of age-at-death distribution | rs149693590 | 22:31976149_C_G | 0.989221 | -0.01962 | 0.004232 | 3.60E-06 | 0.95516 | 0.788551 |
| Top 1% of age-at-death distribution | rs80267918 | 9:92926255_G_A | 0.96955 | -0.01162 | 0.002513 | 3.70E-06 | 0.96948 | 0.446272 |
| Top 1% of age-at-death distribution | rs73305396 | 7:4832521_C_T | 0.974922 | -0.01259 | 0.00272 | 3.70E-06 | 0.99632 | 0.89815 |
| Top 1% of age-at-death distribution | rs111281919 | 20:5707624_T_A | 0.986476 | -0.02354 | 0.005085 | 3.70E-06 | 0.51679 | 1 |
| Top 1% of age-at-death distribution | rs199769928 | 9:92928028_AT_A | 0.969983 | -0.01172 | 0.002535 | 3.80E-06 | 0.96561 | 0.409977 |
| Top 1% of age-at-death distribution | rs1382785 | 6:160573407_C_T | 0.626764 | -0.00407 | 0.000882 | 4.00E-06 | 0.99695 | 0.433691 |
| Top 1% of age-at-death distribution | rs73199941 | 8:18573919_T_A | 0.980604 | -0.01471 | 0.003196 | 4.20E-06 | 0.93489 | 0.061263 |
| Top 1% of age-at-death distribution | rs17135109 | 7:4816574_C_T | 0.97602 | -0.01294 | 0.002813 | 4.30E-06 | 0.98591 | 1 |
| Top 1% of age-at-death distribution | rs11944781 | 4:138539317_C_T | 0.969609 | -0.01169 | 0.002544 | 4.30E-06 | 0.94437 | 0.080501 |
| Top 1% of age-at-death distribution | rs59748091 | 11:19304435_C_T | 0.790421 | -0.00485 | 0.001056 | 4.40E-06 | 0.98167 | 0.228229 |
| Top 1% of age-at-death distribution | rs568405061 | 7:4873428_CG_C | 0.970993 | -0.01166 | 0.002539 | 4.40E-06 | 0.97936 | 0.366345 |
| Top 1% of age-at-death distribution | rs3800007 | 6:57236977_C_T | 0.969686 | -0.01239 | 0.0027 | 4.40E-06 | 0.82514 | 0.466455 |
| Top 1% of age-at-death distribution | rs150209652 | 7:4876064_G_A | 0.977961 | -0.01338 | 0.002918 | 4.50E-06 | 0.9955 | 0.946187 |
| Top 1% of age-at-death distribution | rs16849041 | 1:165627569_A_C | 0.928603 | -0.00763 | 0.001664 | 4.50E-06 | 0.99251 | 0.80636 |
| Top 1% of age-at-death distribution | rs200224959 | 7:101143523_AG_A | 0.982843 | -0.01516 | 0.003313 | 4.80E-06 | 0.98838 | 0.523805 |
| Top 1% of age-at-death distribution | rs58044828 | 11:19304367_C_T | 0.790768 | -0.00483 | 0.001057 | 4.80E-06 | 0.98135 | 0.201995 |
| Top 1% of age-at-death distribution | rs73314502 | 7:4873205_A_T | 0.970668 | -0.01151 | 0.002517 | 4.80E-06 | 0.97851 | 0.366606 |
| Top 1% of age-at-death distribution | rs71752266 | 6:92876448_TGTTGATGAGA_T | 0.69838 | 0.004279 | 0.000936 | 4.90E-06 | 0.98424 | 0.789787 |
| Top 1% of age-at-death distribution | rs75511781 | 7:131323710_A_G | 0.956922 | -0.00957 | 0.002096 | 4.90E-06 | 1 | 0.830376 |
[truncated: 115,237 more chars]
