# Supplementary material for: Human longevity is influenced by many genetic variants: evidence from 75,000 UK Biobank participants
Source: Aging (Albany NY). 2016 Mar 23;8(3):547–60. doi: 10.18632/aging.100930 (PMC4833145; doi:10.18632/aging.100930)
Supplement: Supplementary file 5 [file aging-08-547-s005.docx]

**Supplementary Table 3**

Model results from analyses testing each GRS against parental longevity phenotypes. Models are linear or logistic regression depending on the outcome (whether continuous or binary, respectively). Effect sizes are therefore coefficients or odds ratios, respectively. Benjamini-Hochberg-corrected p-values included

| **GRS (n SNPs)** | **Outcome** | **Model** | **effect** | **ciLower** | **ciUpper** | **p** | **p_BH** | **n** | **n_cases** |
| --- | --- | --- | --- | --- | --- | --- | --- | --- | --- |
| Alzheimer's Disease (8) | Fathers age at death | linear | -0.001 | -0.067 | 0.066 | 9.88E-01 | 9.88E-01 | 63775 |  |
| Alzheimer's Disease (8) | Mothers age at death | linear | -0.111 | -0.173 | -0.050 | 3.90E-04 | 2.12E-03 | 52776 |  |
| Alzheimer's Disease (8) | Combined parent age death (z-score) | linear | -0.013 | -0.024 | -0.003 | 1.39E-02 | 4.59E-02 | 45627 |  |
| Alzheimer's Disease (8) | At least 1 parent in top 1% of age at death range | logistic | 0.954 | 0.914 | 0.996 | 3.12E-02 | 8.78E-02 | 42273 | 1339 |
| Body Mass Index (69) | Fathers age at death | linear | -2.409 | -3.563 | -1.254 | 4.37E-05 | 3.32E-04 | 63775 |  |
| Body Mass Index (69) | Mothers age at death | linear | -0.940 | -2.020 | 0.140 | 8.81E-02 | 1.72E-01 | 52776 |  |
| Body Mass Index (69) | Combined parent age death (z-score) | linear | -0.318 | -0.504 | -0.132 | 8.16E-04 | 4.13E-03 | 45627 |  |
| Body Mass Index (69) | At least 1 parent in top 1% of age at death range | logistic | 0.624 | 0.299 | 1.304 | 2.10E-01 | 3.34E-01 | 42273 | 1339 |
| Breast Cancer (65) | Fathers age at death | linear | -0.028 | -0.059 | 0.003 | 7.99E-02 | 1.69E-01 | 63775 |  |
| Breast Cancer (65) | Mothers age at death | linear | -0.025 | -0.055 | 0.004 | 8.79E-02 | 1.72E-01 | 52776 |  |
| Breast Cancer (65) | Combined parent age death (z-score) | linear | -0.005 | -0.010 | 0.000 | 3.52E-02 | 9.55E-02 | 45627 |  |
| Breast Cancer (65) | At least 1 parent in top 1% of age at death range | logistic | 1.012 | 0.992 | 1.032 | 2.60E-01 | 3.79E-01 | 42273 | 1339 |
| Coronary Artery Disease (CAD) (42) | Fathers age at death | linear | -0.133 | -0.176 | -0.090 | 1.16E-09 | 4.41E-08 | 63775 |  |
| Coronary Artery Disease (CAD) (42) | Mothers age at death | linear | -0.093 | -0.133 | -0.054 | 4.19E-06 | 4.55E-05 | 52776 |  |
| Coronary Artery Disease (CAD) (42) | Combined parent age death (z-score) | linear | -0.024 | -0.031 | -0.017 | 1.52E-11 | 1.16E-09 | 45627 |  |
| Coronary Artery Disease (CAD) (42) | At least 1 parent in top 1% of age at death range | logistic | 0.940 | 0.914 | 0.966 | 9.40E-06 | 8.93E-05 | 42273 | 1339 |
| CAD (no LDL, HDL or TG SNPs) (23) | Fathers age at death | linear | -0.148 | -0.205 | -0.091 | 3.34E-07 | 4.23E-06 | 63775 |  |
| CAD (no LDL, HDL or TG SNPs) (23) | Mothers age at death | linear | -0.099 | -0.151 | -0.046 | 2.53E-04 | 1.48E-03 | 52776 |  |
| CAD (no LDL, HDL or TG SNPs) (23) | Combined parent age death (z-score) | linear | -0.024 | -0.033 | -0.015 | 1.81E-07 | 2.75E-06 | 45627 |  |
| CAD (no LDL, HDL or TG SNPs) (23) | At least 1 parent in top 1% of age at death range | logistic | 0.942 | 0.908 | 0.976 | 1.11E-03 | 4.96E-03 | 42273 | 1339 |
| Crohn's disease (139) | Fathers age at death | linear | -0.019 | -0.047 | 0.009 | 1.86E-01 | 3.14E-01 | 63775 |  |
| Crohn's disease (139) | Mothers age at death | linear | -0.017 | -0.043 | 0.009 | 2.11E-01 | 3.34E-01 | 52776 |  |
| Crohn's disease (139) | Combined parent age death (z-score) | linear | -0.005 | -0.010 | -0.001 | 2.36E-02 | 6.90E-02 | 45627 |  |
| Crohn's disease (139) | At least 1 parent in top 1% of age at death range | logistic | 0.982 | 0.965 | 1.000 | 4.88E-02 | 1.20E-01 | 42273 | 1339 |
| Forced Vital Capacity (6) | Fathers age at death | linear | -0.001 | -0.007 | 0.004 | 6.20E-01 | 6.93E-01 | 63775 |  |
| Forced Vital Capacity (6) | Mothers age at death | linear | -0.002 | -0.007 | 0.003 | 3.97E-01 | 5.20E-01 | 52776 |  |
| Forced Vital Capacity (6) | Combined parent age death (z-score) | linear | 0.000 | -0.001 | 0.000 | 2.80E-01 | 3.87E-01 | 45627 |  |
| Forced Vital Capacity (6) | At least 1 parent in top 1% of age at death range | logistic | 1.000 | 0.997 | 1.003 | 9.69E-01 | 9.88E-01 | 42273 | 1339 |
| High Density Lipoprotein (67) | Fathers age at death | linear | 0.612 | -0.046 | 1.270 | 6.82E-02 | 1.55E-01 | 63775 |  |
| High Density Lipoprotein (67) | Mothers age at death | linear | 0.184 | -0.431 | 0.799 | 5.58E-01 | 6.63E-01 | 52776 |  |
| High Density Lipoprotein (67) | Combined parent age death (z-score) | linear | 0.075 | -0.031 | 0.181 | 1.63E-01 | 2.82E-01 | 45627 |  |
| High Density Lipoprotein (67) | At least 1 parent in top 1% of age at death range | logistic | 1.804 | 1.187 | 2.743 | 5.74E-03 | 2.42E-02 | 42273 | 1339 |
| Inflammatory Bowel Disease (156) | Fathers age at death | linear | -0.033 | -0.060 | -0.006 | 1.73E-02 | 5.48E-02 | 63775 |  |
| Inflammatory Bowel Disease (156) | Mothers age at death | linear | -0.013 | -0.038 | 0.012 | 3.17E-01 | 4.30E-01 | 52776 |  |
| Inflammatory Bowel Disease (156) | Combined parent age death (z-score) | linear | -0.006 | -0.010 | -0.002 | 7.00E-03 | 2.80E-02 | 45627 |  |
| Inflammatory Bowel Disease (156) | At least 1 parent in top 1% of age at death range | logistic | 0.987 | 0.970 | 1.004 | 1.44E-01 | 2.61E-01 | 42273 | 1339 |
| Low Density Lipoprotein (49) | Fathers age at death | linear | -1.297 | -1.924 | -0.670 | 5.06E-05 | 3.50E-04 | 63775 |  |
| Low Density Lipoprotein (49) | Mothers age at death | linear | -0.758 | -1.342 | -0.173 | 1.11E-02 | 3.83E-02 | 52776 |  |
| Low Density Lipoprotein (49) | Combined parent age death (z-score) | linear | -0.269 | -0.370 | -0.168 | 1.76E-07 | 2.75E-06 | 45627 |  |
| Low Density Lipoprotein (49) | At least 1 parent in top 1% of age at death range | logistic | 0.433 | 0.290 | 0.647 | 4.34E-05 | 3.32E-04 | 42273 | 1339 |
| Menopause age (52) | Fathers age at death | linear | -0.129 | -0.326 | 0.067 | 1.98E-01 | 3.27E-01 | 63775 |  |
| Menopause age (52) | Mothers age at death | linear | 0.135 | -0.048 | 0.318 | 1.49E-01 | 2.63E-01 | 52776 |  |
| Menopause age (52) | Combined parent age death (z-score) | linear | 0.010 | -0.022 | 0.041 | 5.56E-01 | 6.63E-01 | 45627 |  |
| Menopause age (52) | At least 1 parent in top 1% of age at death range | logistic | 1.022 | 0.901 | 1.158 | 7.38E-01 | 8.01E-01 | 42273 | 1339 |
| Prostate Cancer (85) | Fathers age at death | linear | -0.007 | -0.034 | 0.020 | 6.07E-01 | 6.92E-01 | 63775 |  |
| Prostate Cancer (85) | Mothers age at death | linear | 0.000 | -0.025 | 0.025 | 9.85E-01 | 9.88E-01 | 52776 |  |
| Prostate Cancer (85) | Combined parent age death (z-score) | linear | -0.001 | -0.006 | 0.003 | 5.34E-01 | 6.55E-01 | 45627 |  |
| Prostate Cancer (85) | At least 1 parent in top 1% of age at death range | logistic | 0.989 | 0.973 | 1.006 | 2.22E-01 | 3.44E-01 | 42273 | 1339 |
| Stroke (4) | Fathers age at death | linear | -1.242 | -2.495 | 0.012 | 5.22E-02 | 1.24E-01 | 63775 |  |
| Stroke (4) | Mothers age at death | linear | -0.430 | -1.597 | 0.738 | 4.71E-01 | 5.87E-01 | 52776 |  |
| Stroke (4) | Combined parent age death (z-score) | linear | -0.167 | -0.368 | 0.035 | 1.05E-01 | 1.95E-01 | 45627 |  |
| Stroke (4) | At least 1 parent in top 1% of age at death range | logistic | 0.434 | 0.195 | 0.967 | 4.10E-02 | 1.04E-01 | 42273 | 1339 |
| Systolic Blood Pressure (26) | Fathers age at death | linear | -0.279 | -0.375 | -0.182 | 1.47E-08 | 3.72E-07 | 63775 |  |
| Systolic Blood Pressure (26) | Mothers age at death | linear | -0.082 | -0.172 | 0.008 | 7.39E-02 | 1.60E-01 | 52776 |  |
| Systolic Blood Pressure (26) | Combined parent age death (z-score) | linear | -0.031 | -0.046 | -0.015 | 1.20E-04 | 7.60E-04 | 45627 |  |
| Systolic Blood Pressure (26) | At least 1 parent in top 1% of age at death range | logistic | 0.937 | 0.881 | 0.997 | 3.99E-02 | 1.04E-01 | 42273 | 1339 |
| Telomere Length (7) | Fathers age at death | linear | 0.229 | -1.326 | 1.785 | 7.73E-01 | 8.09E-01 | 63775 |  |
| Telomere Length (7) | Mothers age at death | linear | -0.856 | -2.306 | 0.594 | 2.47E-01 | 3.74E-01 | 52776 |  |
| Telomere Length (7) | Combined parent age death (z-score) | linear | -0.073 | -0.323 | 0.177 | 5.68E-01 | 6.64E-01 | 45627 |  |
| Telomere Length (7) | At least 1 parent in top 1% of age at death range | logistic | 1.504 | 0.557 | 4.059 | 4.21E-01 | 5.42E-01 | 42273 | 1339 |
| Triglycerides (37) | Fathers age at death | linear | -1.366 | -2.170 | -0.562 | 8.72E-04 | 4.14E-03 | 63775 |  |
| Triglycerides (37) | Mothers age at death | linear | -0.183 | -0.930 | 0.563 | 6.30E-01 | 6.94E-01 | 52776 |  |
| Triglycerides (37) | Combined parent age death (z-score) | linear | -0.170 | -0.299 | -0.041 | 1.00E-02 | 3.62E-02 | 45627 |  |
| Triglycerides (37) | At least 1 parent in top 1% of age at death range | logistic | 0.820 | 0.491 | 1.371 | 4.50E-01 | 5.70E-01 | 42273 | 1339 |
| Type-1 Diabetes (29) | Fathers age at death | linear | -0.049 | -0.090 | -0.007 | 2.25E-02 | 6.84E-02 | 63775 |  |
| Type-1 Diabetes (29) | Mothers age at death | linear | -0.036 | -0.075 | 0.003 | 6.93E-02 | 1.55E-01 | 52776 |  |
| Type-1 Diabetes (29) | Combined parent age death (z-score) | linear | -0.009 | -0.016 | -0.002 | 7.53E-03 | 2.86E-02 | 45627 |  |
| Type-1 Diabetes (29) | At least 1 parent in top 1% of age at death range | logistic | 1.014 | 0.987 | 1.041 | 3.25E-01 | 4.33E-01 | 42273 | 1339 |
| Type-2 Diabetes (55) | Fathers age at death | linear | -0.336 | -0.740 | 0.069 | 1.04E-01 | 1.95E-01 | 63775 |  |
| Type-2 Diabetes (55) | Mothers age at death | linear | -0.098 | -0.476 | 0.279 | 6.10E-01 | 6.92E-01 | 52776 |  |
| Type-2 Diabetes (55) | Combined parent age death (z-score) | linear | -0.036 | -0.102 | 0.029 | 2.73E-01 | 3.84E-01 | 45627 |  |
| Type-2 Diabetes (55) | At least 1 parent in top 1% of age at death range | logistic | 0.797 | 0.615 | 1.031 | 8.43E-02 | 1.72E-01 | 42273 | 1339 |
| Ulcerative Colitis (87) | Fathers age at death | linear | -0.020 | -0.055 | 0.014 | 2.51E-01 | 3.74E-01 | 63775 |  |
| Ulcerative Colitis (87) | Mothers age at death | linear | -0.005 | -0.037 | 0.027 | 7.63E-01 | 8.09E-01 | 52776 |  |
| Ulcerative Colitis (87) | Combined parent age death (z-score) | linear | -0.003 | -0.009 | 0.002 | 2.64E-01 | 3.79E-01 | 45627 |  |
| Ulcerative Colitis (87) | At least 1 parent in top 1% of age at death range | logistic | 1.003 | 0.981 | 1.026 | 7.77E-01 | 8.09E-01 | 42273 | 1339 |
