# Supplementary material for: Human longevity is influenced by many genetic variants: evidence from 75,000 UK Biobank participants
Source: Aging (Albany NY). 2016 Mar 23;8(3):547–60. doi: 10.18632/aging.100930 (PMC4833145; doi:10.18632/aging.100930)
Supplement: Supplementary file 6 [file aging-08-547-s006.docx]

**Supplementary Table 4**

Specific a priori SNPs associated with longevity or related phenotypes and their 4 associations from the GWAS of four parental-age-at-death longevity traits in UK Biobank.

[Split over two pages.]

|  |  |  |  | Combined parents age at death | | | Father's age at death | | |  |  |  |
| --- | --- | --- | --- | --- | --- | --- | --- | --- | --- | --- | --- | --- |
| **SNP** | **POS_build37** | **gene** | **link** | **BETA_parents** | **SE_parents** | **P_parents** | **BETA_fathers** | **SE_fathers** | **P_fathers** | **A1** | **A0** | **A1FREQ** |
| rs2802292 | 6:108908518 | FOXO3A | Broer 2014. EU_longevity, cases: 90+, controls <65. | -0.0054 | 0.0078 | 4.90E-01 | 0.0058 | 0.0048 | 2.30E-01 | G | T | 0.37 |
| rs10457180 | 6:108965039 | FOXO3A | Broer 2014. EU_longevity, cases: 90+, controls <65. | -0.0033 | 0.0083 | 6.90E-01 | 0.0035 | 0.0050 | 4.90E-01 | G | A | 0.29 |
| rs13217795 | 6:108974098 | FOXO3A | Soerensen, 2010. | -0.0030 | 0.0083 | 7.20E-01 | 0.0027 | 0.0051 | 5.90E-01 | C | T | 0.29 |
| rs2764264 | 6:108934461 | FOXO3A | Soerensen, 2010. | -0.0065 | 0.0083 | 4.30E-01 | 0.0030 | 0.0050 | 5.50E-01 | C | T | 0.30 |
| rs479744 | 6:109020032 | FOXO3A | Soerensen, 2010. | -0.0046 | 0.0095 | 6.30E-01 | -0.0035 | 0.0058 | 5.40E-01 | G | T | 0.79 |
| rs9400239 | 6:108977663 | FOXO3A | Soerensen, 2010. | -0.0032 | 0.0083 | 7.00E-01 | 0.0036 | 0.0051 | 4.70E-01 | T | C | 0.29 |
| rs12206094 | 6:108906200 | FOXO3A | Soerensen, 2010. | 0.0027 | 0.0084 | 7.50E-01 | -0.0070 | 0.0051 | 1.70E-01 | C | T | 0.72 |
| rs13220810 | 6:108913201 | FOXO3A | Soerensen, 2010. | -0.0098 | 0.0087 | 2.60E-01 | 0.0100 | 0.0053 | 5.80E-02 | T | C | 0.74 |
| rs7762395 | 6:108945107 | FOXO3A | Soerensen, 2010. | -0.0037 | 0.0104 | 7.20E-01 | -0.0028 | 0.0064 | 6.60E-01 | G | A | 0.85 |
| rs9486902 | 6:108878052 | FOXO3A | Soerensen, 2010. | -0.0033 | 0.0103 | 7.40E-01 | -0.0041 | 0.0063 | 5.20E-01 | C | T | 0.84 |
| rs1935949 | 6:108999287 | FOXO3A | Bao, 2014. | -0.0005 | 0.0084 | 9.50E-01 | 0.0052 | 0.0051 | 3.10E-01 | A | G | 0.28 |
| rs2721069 | 13:41143720 | FOXO1A | FOXO longevity patent 2015 | -0.0045 | 0.0082 | 5.80E-01 | -0.0055 | 0.0050 | 2.70E-01 | C | T | 0.69 |
| rs2075650 | 19:45395619 | APOE | Broer 2014. EU_longevity, cases: 90+, controls <65. | 0.0391 | 0.0102 | 1.30E-04 | 0.0107 | 0.0062 | 8.60E-02 | A | G | 0.87 |
| rs429358 | 19:45411941 | APOE | Deelen 2011. Part of E4 definition | 0.0521 | 0.0105 | 7.60E-07 | 0.0171 | 0.0064 | 7.50E-03 | T | C | 0.86 |
| rs7412 | 19:45412079 | APOE | Part of E4 definition | -0.0315 | 0.0135 | 2.00E-02 | -0.0208 | 0.0083 | 1.20E-02 | C | T | 0.92 |
| rs405509 | 19:45408836 | APOE | Soerensen 2013 | -0.0141 | 0.0076 | 6.10E-02 | -0.0075 | 0.0046 | 1.00E-01 | T | G | 0.49 |
| rs4420638 | 19:45422946 | APOE | Bertram 2007. Coinherited with APOE E4, exacerbates AD phenotype | 0.0401 | 0.0096 | 2.70E-05 | 0.0129 | 0.0058 | 2.70E-02 | A | G | 0.81 |
| rs2811712 | 9:21998035 | CDKN2A | Melzer 2007. Physical activity in older people | -0.0068 | 0.0123 | 5.80E-01 | -0.0122 | 0.0075 | 1.00E-01 | G | A | 0.10 |
| rs1333049 | 9:22125503 | CDKN2A | Dichgans 2013. CHD | 0.0189 | 0.0075 | 1.20E-02 | 0.0146 | 0.0046 | 1.50E-03 | G | C | 0.52 |
| rs4977574 | 9:22098574 | CDKN2A | Schunkert 2013. CHD | 0.0168 | 0.0075 | 2.60E-02 | 0.0143 | 0.0046 | 1.80E-03 | A | G | 0.52 |
| rs17694493 | 9:22041998 | CDKN2B-AS1 | Al Olama 2014. Prostate cancer | 0.0144 | 0.0111 | 1.90E-01 | 0.0078 | 0.0067 | 2.50E-01 | C | G | 0.86 |
| rs1011970 | 9:22062134 | CDKN2B-AS1 | Michailidou 2013. Breast Cancer | 0.0117 | 0.0101 | 2.50E-01 | 0.0108 | 0.0061 | 7.70E-02 | G | T | 0.83 |
| rs2151280 | 9:22034719 | CDKN2B-AS1 | Stacey 2015. Basal cell carcinoma | 0.0105 | 0.0076 | 1.70E-01 | 0.0081 | 0.0046 | 7.80E-02 | G | A | 0.54 |
| rs10811661 | 9:22134094 | CDKN2A | Mahajan 2014. T2D | -0.0110 | 0.0099 | 2.70E-01 | 0.0003 | 0.0060 | 9.60E-01 | T | C | 0.83 |
| rs4977756 | 19:45412079 | CDKN2B/ANRIL | Fortney 2015. 90+ survival | 0.0171 | 0.0077 | 2.50E-02 | 0.0153 | 0.0047 | 1.10E-03 | G | A | 0.41 |
| rs3184504 | 19:45408836 | SH2B3/ATXN2 | Fortney 2015. 90+ survival | -0.0176 | 0.0075 | 1.90E-02 | -0.0115 | 0.0046 | 1.20E-02 | T | C | 0.48 |
| rs514659 | 19:45422946 | ABO | Fortney 2015. 90+ survival | 0.0098 | 0.0080 | 2.20E-01 | 0.0081 | 0.0049 | 9.90E-02 | A | C | 0.69 |
| rs1051730 | 15:78894339 | CHRNA3 | Furberg 2010. Association with number of cigarettes smoked | 0.0295 | 0.0080 | 2.50E-04 | 0.0269 | 0.0049 | 3.00E-08 | G | A | 0.67 |
| rs1329650 | 10:93348120 | LOC100188947 | Furberg 2010. Association with number of cigarettes smoked | -0.0111 | 0.0085 | 1.90E-01 | 0.0026 | 0.0052 | 6.20E-01 | G | T | 0.73 |
| rs3733829 | 19:41310571 | EGLN2, near CYP2A6 | Furberg 2010. Association with number of cigarettes smoked | 0.0002 | 0.0078 | 9.80E-01 | 0.0001 | 0.0048 | 9.80E-01 | A | G | 0.64 |
| rs671 | 12:112241766 | ALDH2 | Quillen 2013. Alcohol dependence. Effect=unit decrease | -0.3447 | 0.4454 | 4.40E-01 | -0.0200 | 0.2942 | 9.50E-01 | G | A | 1.00 |
| rs11933661 | 4:131013140 | intergenic | Quillen 2013. Alcohol dependence. Effect=unit decrease | -0.0007 | 0.0076 | 9.20E-01 | -0.0043 | 0.0046 | 3.50E-01 | C | T | 0.55 |
| rs4478858 | 1:31883925 | SERINC2 | Zuo 2013. Alcohol dependence | 0.0048 | 0.0076 | 5.30E-01 | -0.0001 | 0.0046 | 9.80E-01 | T | C | 0.57 |
| rs2149954 | 5:157820602 | lncRNA 5q33.3 | Deelen 2014. Survival 90+ (controls <65 years) | -0.0218 | 0.0078 | 5.20E-03 | -0.0120 | 0.0048 | 1.20E-02 | C | T | 0.64 |
| rs6025 | 1:169519049 | F5 | Leiden F5. Deep vein thrombosis | 0.0260 | 0.0250 | 3.00E-01 | -0.0015 | 0.0153 | 9.20E-01 | T | C | 0.02 |

|  |  |  |  | Mother's age at death | | | Top 1% of age at death | | |  |  |  |
| --- | --- | --- | --- | --- | --- | --- | --- | --- | --- | --- | --- | --- |
| **SNP** | **POS_build37** | **gene** | **link** | **BETA_mothers** | **SE_mothers** | **P_mother** | **BETA_top1%** | **SE_top1%** | **P_top1%** | **A1** | **A0** | **A1FREQ** |
| rs2802292 | 6:108908518 | FOXO3A | Broer 2014. EU_longevity, cases: 90+, controls <65. | -0.0024 | 0.0053 | 6.60E-01 | -0.0001 | 0.0009 | 9.20E-01 | G | T | 0.37 |
| rs10457180 | 6:108965039 | FOXO3A | Broer 2014. EU_longevity, cases: 90+, controls <65. | 0.0008 | 0.0056 | 8.90E-01 | 0.0007 | 0.0009 | 4.30E-01 | G | A | 0.29 |
| rs13217795 | 6:108974098 | FOXO3A | Soerensen, 2010. | 0.0009 | 0.0056 | 8.70E-01 | 0.0011 | 0.0009 | 2.60E-01 | C | T | 0.29 |
| rs2764264 | 6:108934461 | FOXO3A | Soerensen, 2010. | 0.0004 | 0.0056 | 9.40E-01 | 0.0010 | 0.0009 | 2.60E-01 | C | T | 0.30 |
| rs479744 | 6:109020032 | FOXO3A | Soerensen, 2010. | -0.0028 | 0.0064 | 6.60E-01 | 0.0004 | 0.0011 | 6.80E-01 | G | T | 0.79 |
| rs9400239 | 6:108977663 | FOXO3A | Soerensen, 2010. | 0.0007 | 0.0056 | 9.00E-01 | 0.0010 | 0.0009 | 2.80E-01 | T | C | 0.29 |
| rs12206094 | 6:108906200 | FOXO3A | Soerensen, 2010. | 0.0006 | 0.0057 | 9.20E-01 | -0.0008 | 0.0010 | 3.80E-01 | C | T | 0.72 |
| rs13220810 | 6:108913201 | FOXO3A | Soerensen, 2010. | -0.0048 | 0.0059 | 4.20E-01 | 0.0002 | 0.0010 | 8.60E-01 | T | C | 0.74 |
| rs7762395 | 6:108945107 | FOXO3A | Soerensen, 2010. | -0.0056 | 0.0070 | 4.30E-01 | 0.0007 | 0.0012 | 5.70E-01 | G | A | 0.85 |
| rs9486902 | 6:108878052 | FOXO3A | Soerensen, 2010. | -0.0088 | 0.0069 | 2.00E-01 | 0.0010 | 0.0012 | 4.20E-01 | C | T | 0.84 |
| rs1935949 | 6:108999287 | FOXO3A | Bao, 2014. | -0.0004 | 0.0057 | 9.40E-01 | 0.0008 | 0.0009 | 3.70E-01 | A | G | 0.28 |
| rs2721069 | 13:41143720 | FOXO1A | FOXO longevity patent 2015 | 0.0033 | 0.0055 | 5.50E-01 | -0.0001 | 0.0009 | 9.10E-01 | C | T | 0.69 |
| rs2075650 | 19:45395619 | APOE | Broer 2014. EU_longevity, cases: 90+, controls <65. | 0.0079 | 0.0069 | 2.50E-01 | 0.0016 | 0.0012 | 1.60E-01 | A | G | 0.87 |
| rs429358 | 19:45411941 | APOE | Deelen 2011. Part of E4 definition | 0.0183 | 0.0071 | 1.00E-02 | 0.0023 | 0.0012 | 5.30E-02 | T | C | 0.86 |
| rs7412 | 19:45412079 | APOE | Part of E4 definition | -0.0019 | 0.0093 | 8.40E-01 | -0.0019 | 0.0016 | 2.20E-01 | C | T | 0.92 |
| rs405509 | 19:45408836 | APOE | Soerensen 2013 | 0.0009 | 0.0051 | 8.60E-01 | -0.0002 | 0.0009 | 8.40E-01 | T | G | 0.49 |
| rs4420638 | 19:45422946 | APOE | Bertram 2007. Coinherited with APOE E4, exacerbates AD phenotype | 0.0232 | 0.0065 | 3.30E-04 | 0.0025 | 0.0011 | 2.00E-02 | A | G | 0.81 |
| rs2811712 | 9:21998035 | CDKN2A | Melzer 2007. Physical activity in older people | 0.0044 | 0.0083 | 6.00E-01 | -0.0002 | 0.0014 | 8.90E-01 | G | A | 0.10 |
| rs1333049 | 9:22125503 | CDKN2A | Dichgans 2013. CHD | 0.0051 | 0.0051 | 3.20E-01 | 0.0018 | 0.0009 | 3.80E-02 | G | C | 0.52 |
| rs4977574 | 9:22098574 | CDKN2A | Schunkert 2013. CHD | 0.0053 | 0.0051 | 3.00E-01 | 0.0011 | 0.0009 | 2.00E-01 | A | G | 0.52 |
| rs17694493 | 9:22041998 | CDKN2B-AS1 | Al Olama 2014. Prostate cancer | 0.0059 | 0.0075 | 4.30E-01 | 0.0023 | 0.0013 | 6.80E-02 | C | G | 0.86 |
| rs1011970 | 9:22062134 | CDKN2B-AS1 | Michailidou 2013. Breast Cancer | 0.0038 | 0.0068 | 5.70E-01 | -0.0012 | 0.0011 | 2.70E-01 | G | T | 0.83 |
| rs2151280 | 9:22034719 | CDKN2B-AS1 | Stacey 2015. Basal cell carcinoma | 0.0048 | 0.0051 | 3.50E-01 | -0.0012 | 0.0009 | 1.60E-01 | G | A | 0.54 |
| rs10811661 | 9:22134094 | CDKN2A | Mahajan 2014. T2D | -0.0053 | 0.0067 | 4.30E-01 | 0.0020 | 0.0011 | 8.00E-02 | T | C | 0.83 |
| rs4977756 | 19:45412079 | CDKN2B/ANRIL | Fortney 2015. 90+ survival | 0.0060 | 0.0052 | 2.40E-01 | -0.0003 | 0.0009 | 7.20E-01 | G | A | 0.41 |
| rs3184504 | 19:45408836 | SH2B3/ATXN2 | Fortney 2015. 90+ survival | -0.0167 | 0.0051 | 1.00E-03 | -0.0019 | 0.0008 | 2.40E-02 | T | C | 0.48 |
| rs514659 | 19:45422946 | ABO | Fortney 2015. 90+ survival | 0.0057 | 0.0055 | 2.90E-01 | -0.0009 | 0.0009 | 3.30E-01 | A | C | 0.69 |
| rs1051730 | 15:78894339 | CHRNA3 | Furberg 2010. Association with number of cigarettes smoked | 0.0171 | 0.0054 | 1.60E-03 | 0.0013 | 0.0009 | 1.40E-01 | G | A | 0.67 |
| rs1329650 | 10:93348120 | LOC100188947 | Furberg 2010. Association with number of cigarettes smoked | -0.0097 | 0.0058 | 9.20E-02 | -0.0011 | 0.0010 | 2.50E-01 | G | T | 0.73 |
| rs3733829 | 19:41310571 | EGLN2, near CYP2A6 | Furberg 2010. Association with number of cigarettes smoked | -0.0032 | 0.0053 | 5.50E-01 | 0.0013 | 0.0009 | 1.30E-01 | A | G | 0.64 |
| rs671 | 12:112241766 | ALDH2 | Quillen 2013. Alcohol dependence. Effect=unit decrease | -0.0451 | 0.2988 | 8.80E-01 | 0.0002 | 0.0155 | 9.90E-01 | G | A | 1.00 |
| rs11933661 | 4:131013140 | intergenic | Quillen 2013. Alcohol dependence. Effect=unit decrease | 0.0030 | 0.0051 | 5.60E-01 | -0.0015 | 0.0009 | 7.50E-02 | C | T | 0.55 |
| rs4478858 | 1:31883925 | SERINC2 | Zuo 2013. Alcohol dependence | -0.0017 | 0.0052 | 7.40E-01 | 0.0007 | 0.0009 | 4.00E-01 | T | C | 0.57 |
| rs2149954 | 5:157820602 | lncRNA 5q33.3 | Deelen 2014. Survival 90+ (controls <65 years) | -0.0082 | 0.0053 | 1.20E-01 | -0.0004 | 0.0009 | 6.40E-01 | C | T | 0.64 |
| rs6025 | 1:169519049 | F5 | Leiden F5. Deep vein thrombosis | 0.0081 | 0.0170 | 6.30E-01 | 0.0018 | 0.0029 | 5.30E-01 | T | C | 0.02 |
